# Design, Synthesis, and Evaluation of Antinociceptive Properties of Novel CBD-Based Terpene-Cinnamoyl-Acyl-Hydrazone Analogues

**DOI:** 10.3390/ph18050755

**Published:** 2025-05-20

**Authors:** Mikaela Lucinda de Souza, João Pedro Barros de Paiva, Graziella dos Reis Rosa Franco, Vanessa Silva Gontijo, Marina Amaral Alves, Hygor Marcos Ribeiro de Souza, Anna Carolina Pereira Lontra, Eduardo Araújo de Oliveira, Thaís Biondino Sardella Giorno, Isabella Alvim Guedes, Laurent Emmanuel Dardenne, Patrícia Dias Fernandes, Claudio Viegas Jr.

**Affiliations:** 1 PeQuiM-Laboratory of Research in Medicinal Chemistry, Institute of Chemistry, Federal University of Alfenas, Alfenas 37133-840, MG, Brazil; mikaela.lucinda@sou.unifal-mg.edu.br (M.L.d.S.); grazireisfranco@yahoo.com.br (G.d.R.R.F.); vanessagontijo@yahoo.com.br (V.S.G.); 2 Laboratory of Pharmacology of Pain and Inflammation, Institute of Biomedical Sciences, Federal University of Rio de Janeiro, Rio de Janeiro 21941-577, RJ, Brazil; jbarrosdepaiva@gmail.com (J.P.B.d.P.); anna.lontra@gmail.com (A.C.P.L.); eduardoaoliveira.ff@gmail.com (E.A.d.O.); thaissardella.ufrj@gmail.com (T.B.S.G.); 3 Laboratory of Metabolomics—LabMeta/LADETEC, Institute of Chemistry, Federal University of Rio de Janeiro, Rio de Janeiro 21941-598, RJ, Brazil; marina.amaral@iq.ufrj.br (M.A.A.); ribeirohygor@ufrj.br (H.M.R.d.S.); 4 National Laboratory for Scientific Computing, Molecular Modeling in Biological Systems Group, Petrópolis 25651-075, RJ, Brazil; isabella.alvimg@gmail.com (I.A.G.); dardenne@lncc.br (L.E.D.)

**Keywords:** CBD analogues, antinociceptive activity, cannabidiol-based analogues, acute pain, bioactive acyl-hydrazone derivatives

## Abstract

**Background/Objectives**: Cannabidiol (CBD) has been reported for its antinociceptive, anti-inflammatory, and neuroprotective activities. However, several legal restrictions on its medicinal uses and even research have contributed to the development of synthetic analogues. Therefore, the aim of this study was the design and synthesis of a novel series of CBD-based structural analogues, and the in vivo evaluation of their potential antinociceptive activity. **Methods**: Using a two-step synthetic route, 26 new terpene-cinnamoyl acyl-hydrazone analogues were obtained and were submitted to in vivo screening in the classical formalin-induced paw edema and hot plate assays. **Results**: The compounds PQM-292, PQM-293, PQM-295, PQM-307, PQM-308, and PQM-309 exhibited the best results in the neurogenic phase (first phase) of the formalin-induced licking response, showing comparable results to morphine. Notably, in the inflammatory phase (second phase), compound PQM-292 exhibited the best anti-inflammatory activity. Interestingly, in the hot plate model, six other compounds (PQM-274, PQM-291, PQM-294, PQM-304, PQM-305, and PQM-378) showed the best antinociceptive activity in comparison to morphine, especially PQM-274, which exhibited an antinociceptive effect almost equivalent to the reference drug. Interestingly, these findings suggested that these bioactive compounds, despite their structural similarity, act through different mechanisms, which were investigated by molecular docking with CB1, CB2, and TRPV1 receptors. In silico results indicated that the most active compounds should act through different mechanisms, probably involving interactions with TRPA1. **Conclusions**: Therefore, due to the promising antinociceptive activity observed for these highlighted compounds, particularly for PQM-292 and PQM-274, without apparent toxicity and psychoactive effects, and the possible involvement of diverse mechanisms of action, these compounds could be considered as promising starting points to the development of new drug candidate prototypes of clinical interest.

## 1. Introduction

**Figure 1:**
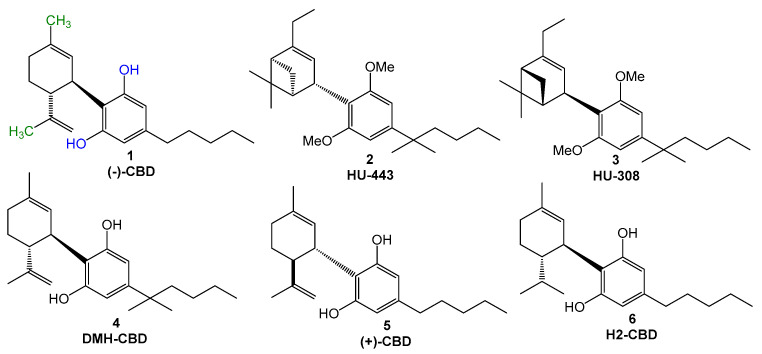
Chemical Structures of (−)-CBD (**1**), HU-443 (**2**), HU-308 (**3**), DMH-CBD (**4**), (+)-CBD (**5**), and H2-CBD (**6**).

*Cannabis* species have been used since ancient times by humans for health purposes. Among the 538 active secondary metabolites identified in *Cannabis*, more than 120 cannabinoids have been identified (ca. 22%) [1,2]. Cannabidiol ((−)-CBD, 1, Figure 1) is a non-psychoactive cannabinoid, which was first isolated from *C. sativa* in 1940. Despite its apparent pharmacological promiscuity, evidenced by its wide range of biological properties and potential interaction with multiple molecular targets, CBD is currently considered a clinically safe drug, with few and relatively smooth adverse effects, including diarrhea and appetite changes, among others. The safety doses are dependent on the route of administration, and, when administered orally, well-tolerated doses are up to 1600 mg [3]. The chemical structure of CBD is constituted by a monoterpene system linked to a 2-6-di-hydroxylated-4-*n*-pentyl-aryl ring and is naturally available only in the *trans*-relative configuration. The bioactivity of CBD seems to be, at least in part, related to the presence of the two hydroxyl groups at the aromatic ring (Figure 1, in blue), as well as the methyl group on the terpene subunit (Figure 1, in green), given that these groups can interact by different modes with amino acid residues in different molecular targets. In addition to the hydroxyl groups, the *n*-pentyl side chain appears to be essential for the antioxidant properties and could contribute to lipophilicity [1]. Amidst the cannabinoids, it was noted that aryl-substituted meroterpenoids (a special class of natural products derived from mixed terpenoid biogenesis) [4], such as CBD, have shown diverse pharmacological properties, such as anti-inflammatory, immunomodulatory [5], antioxidant, neuroprotective, an antimicrobial, and analgesic, among others [1].

To date, over 65 molecular targets have been reported to interact with CBD, such as transient receptor potential ankyrin 1 (TRPA1), transient receptor potential cation channel family (TRP), and cannabinoid receptors type 1 (CB1) and type 2 (CB2), which are the main constituents of the endocannabinoid system (ECS) [3]. Such a wealth of related targets favors different biological activities and pharmacological effects, as mentioned above [3,6,7]. The TRP channels are located in the plasma membrane of animal cells and are related to the analgesic properties of CBD, especially TRPV1. CBD was observed to effectively act against neuropathic pain, a chronic painful condition that impacts 20–25% of individuals worldwide. In addition, CBD can modulate the uptake of neurotransmitters, such as dopamine, noradrenaline, GABA, and serotonin, reinforcing its antinociceptive properties [2].

Regarding the anti-inflammatory effects of CBD, it was discovered that it plays a role in the reduction of levels of pro-inflammatory markers, such as interleukin (IL) IL-1β [1] and IL-6, and, in turn, contributes to counteracting chronic pain conditions [2]. Moreover, CBD interacts directly and indirectly with the ECS, showing lower affinity for CB1, mainly expressed in the brain, than for CB2 receptors, which are most abundant in immune cells. Other pathways of action attributed to CBD involve interactions with the G protein-coupled receptors (GPCRs) and inhibition of arachidonic acid metabolites [2,8]. All these findings corroborate the difficulties underlying the comprehension of CBD’s pharmacokinetics, reinforcing the need for more detailed studies about all potential molecular targets and possible modifications to the structure of CBD, which could result in an enhanced pharmacological profile.

As the natural cannabinoids, their synthetic derivatives have shown interesting biological activities [9], as exemplified by compounds HU-443 (**2**, Figure 1) and HU-308 (**3**), which exhibit significant anti-inflammatory and analgesic activities, acting as selective CB2 ligands, and the anti-inflammatory derivative DMH-CBD (**4**, Figure 1) [10]. It is important to note that the poor bioavailability of CBD is a major problem in evaluating its therapeutic effectiveness [1], stimulating the search for novel structurally CBD-based analogues. The literature data show that the most usual modifications proposed by authors were related to the *n*-pentyl sidechain, in the hydroxy-substituents in the aromatic ring, or the methyl groups in the terpene subunit. Structural changes in (−)-CBD also included stereochemical aspects, including the synthesis of its enantiomer (+)-CBD (**5**, Figure 1), which showed a better affinity for cannabinoid receptors, with a slight affinity for CB1. Hydrogenation of (−)-CBD at the isopropenyl functionality led to H2-CBD (**6**, Figure 1), which exhibited significant anti-inflammatory activity against ROS, nitric oxide, and tumor necrosis factor alfa (TNF-α), and higher affinity for CB1 [8]. The literature shows that alkylamides can bind to the CB2 receptor, with a stronger interaction than that of the endogenous cannabinoids [5].

However, due to intolerance after addiction to opium, most governments prohibit the use of products derived from cannabis, including CBD [11], which affects research and scientific advances [10]. The legislation of cannabinoids remained outdated, since it dates from 1906, and only in the last decade was it fully revised for medicinal and research uses in many countries. In the USA, cannabis is categorized as marijuana (or “marihuana”), in reference to the fully plant *C. sativa*, encompassing its seeds, leaf, flowers, constituents, and derivatives; and hemp, which refers to the plant *C. sativa* when it has a 0.3% concentration of Δ^9^-tetrahydrocannabinol (Δ^9^-THC), its main psychoactive constituent. In 1937, the USA Congress created the Marihuana Tax Act (Tax Act), regulating and taxing all cannabis analogues, which highly impacted scientific and clinical research, and, in 1961, the United Nations Single Convention on Narcotic Drugs determined CBD as a ‘liable to abuse’ substance [11]. It was only in 2018, with the ‘2018 Farm Bill’, that the USA Congress accepted the classification of hemp with 0.3% of Δ^9^-THC and, therefore, CBD derived from hemp became not classified as a controlled substance, leading to the approval of Epidiolex^®^ by the Food & Drug Administration (FDA), the first CBD-based drug for the treatment of epilepsy and convulsion [9]. Finally, in 2020, following recommendations by the World Health Organization (WHO), the USA removed cannabis from the most restrictive schedule, but its medicinal use remains illegal in many American regions and in other countries worldwide [11].

Therefore, given the controversial current global legislation about cannabis versus the promising therapeutic benefits of CBD and its analogues, many research groups have dedicated efforts to the discovery of new synthetic cannabinoids and CBD-based analogues as drug candidates. Herein, we report for the first time the synthesis and evaluation of a series of terpene-cinnamoyl-acyl-hydrazones, designed as CBD-based structural analogues with potential antinociceptive and anti-inflammatory activity.

## 2. Results

**Figure 2:**
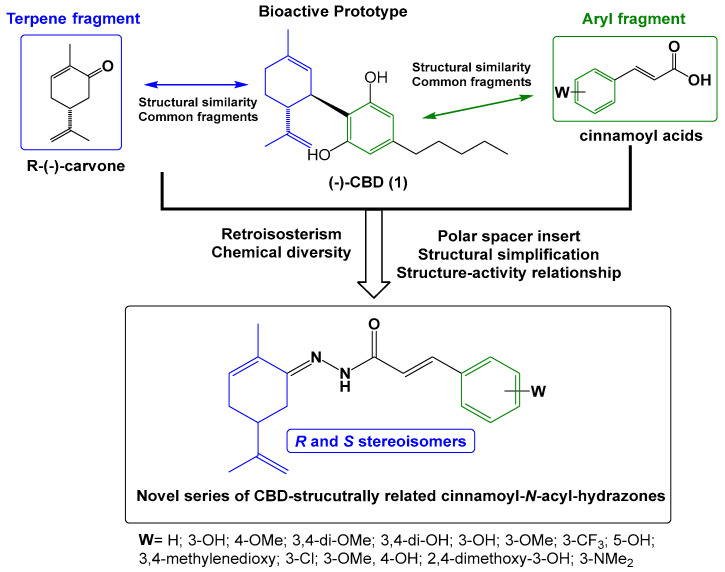
Rational structural design of a new series of the CBD-based terpene-cinnamoyl-*N*-acyl-hydrazone analogues.

The series of new series of terpene-cinnamoyl-acyl-hydrazones **10a**–**m**/**11a**–**m** was designed from the molecular architecture of CBD (**1**, Figure 2), with the carvone structure representing the monoterpene moiety linked to a functionalized aromatic subunit by the introduction of an *N*-acyl-hydrazone spacer. The rationale for the introduction of an *N*-acyl-hydrazone function was based on its potential contribution to the modulation of physical–chemical properties, such as solubility, acidity, and the ability to perform polar interactions, with a crucial impact on pharmacokinetics [12,13,14]. In addition, *N*-acyl-hydrazone is considered a privileged structure in drug discovery, acting as an important biophore in ligands with anti-inflammatory and antinociceptive activity [15,16,17]. These structural changes aimed to preserve a similar structural pattern to CBD (**1**) and allow researchers to study how the structural changes proposed could impact the pharmacological activity regarding the position of the endocyclic double bond, changes in the stereochemistry of the *iso*-propenyl group, diverse functionalization on the aromatic ring, removing the alkyl side-chain, and introduction of the *N*-acyl-hydrazone subunit.

**Figure 3:**
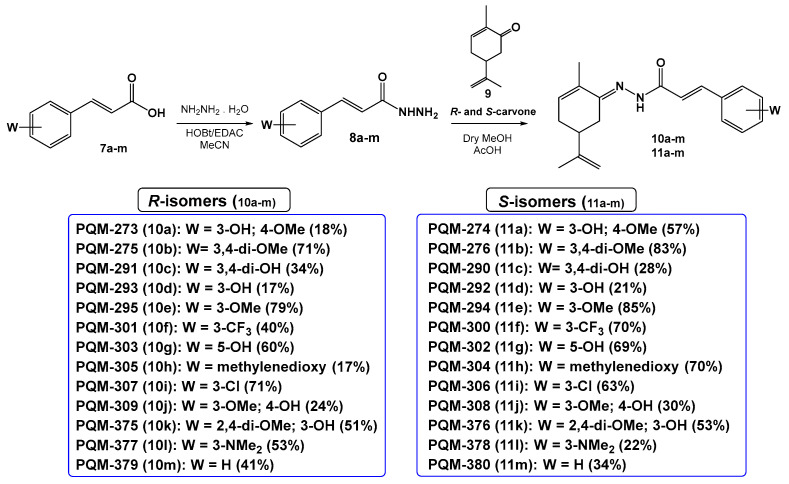
Synthetic route for the preparation of the terpene-cinnamoyl-*N*-acyl-hydrazones **10a**–**m** and **11a**–**m**.

The synthesis of the target compounds was performed in a linear two-step route (Figure 3). In the first step, a series of commercially available functionalized cinnamic acids (**7a**–**m**) was converted into the corresponding hydrazides (**8a**–**m**) by a hydrazinolysis reaction with hydrazine hydrate, catalyzed by EDAC/HOBt [18]. Next, hydrazides **8a**–**m** were acid-catalyzed reacted with *R*- or *S*-carvone, leading to the desired series of *R-*(**10a**–**m**) and *S-N-*acyl-hydrazones (**11a**–**m**), respectively. As a result, 26 compounds were obtained as pure solids in 17–83% yields (analytical and spectroscopic data available in the Supplementary Materials).

### 2.1. Biological Evaluation

The first step of the biological evaluation was focused on the in vivo toxicity of orally administered compounds. After oral administration of a dose of 10 µmol/kg, the animals were observed for possible behavioral changes for a period of 24 h. Next, blood samples were obtained for hemogram analysis. It was observed that none of the compounds caused behavioral changes (e.g., irritation, drowsiness, convulsions, raised fur, sedation, constipation, and diarrhea) or changes in the amounts of water and food intake. I addition, the evaluation of hematological parameters did not indicate any alteration in total and differential cell counts or hemoglobin, hematocrit, and platelet numbers

**Figure 4:**
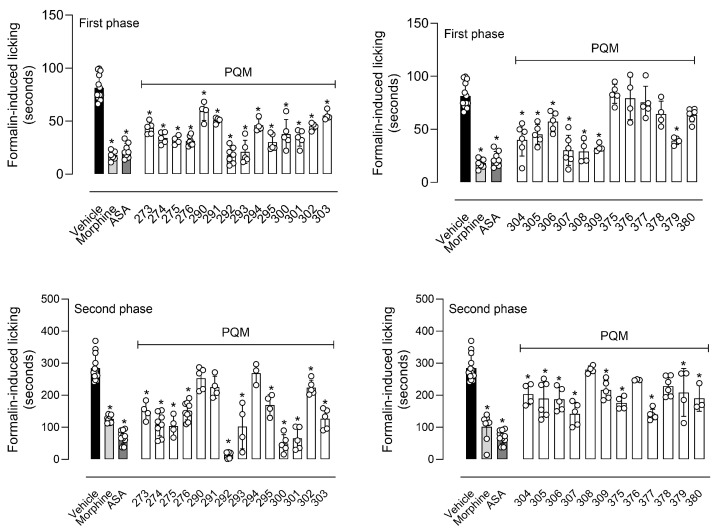
Antinociceptive effect of compounds on the licking response induced by formalin in mice. Animals were pretreated with different doses of vehicle, morphine (5 µmol/kg), acetylsalicylic acid (ASA, 1100 µmol/kg), or compounds (10 µmol/kg), 60 min before the injection of formalin (2.5%/paw). The results are presented as mean ± SD (*n* = 5 per group) of the time that the animal spent licking the capsaicin-injected paw. One-way or two-way analysis of variance (ANOVA) followed by Tukey’s post hoc test was used for unpaired data when more than two groups were compared to the same control. The post hoc tests were run only if F achieved the necessary level of statistical significance. * When *p* was lower than 0.05, group differences were considered significant.

Figure 4 demonstrates that pre-treatment of mice with a single dose of 10 µmol/kg of each compound resulted in a significant reduction in the licking behavior in the neurogenic (first phase) of the formalin assay. Except for PQM-375 (**10k**) to PQM-378 (**11l**), and PQM-380 (**11m**), all other compounds caused a reduction in the mice response, particularly for PQM-292 (**11d**), PQM-293 (**10d**), PQM-295 (**10e**), PQM-307 (**10i**), PQM-308 (**11j**), and PQM-309 (**10j**), which exhibited antinociceptive activity comparable to morphine, which was used as the reference drug. Regarding the inflammatory phase (second phase) of the model, it was observed that PQM-290 (**11c**), PQM-291 (**10c**), PQM-294 (**11e**), PQM-308 (**11j**), PQM-376 (**11k**), and PQM-378 (**11l**) did not show significant ability to reduce formalin-induced inflammatory response. In contrast, PQM-292 (**11d**) stood out among all other compounds for exhibiting the highest antinociceptive effect, almost completely abolishing the response to painful stimuli at a dose of 10 μmol/Kg, even when compared to the positive control groups of morphine and ASA.

The main differences between the two response phases in the formalin-induced paw-licking model lie in the fact that the first neurogenic phase is mainly associated with direct activation of nociceptors, and the painful stimuli are transmitted to the central nervous system (CNS) by afferent C and Aδ-fibers. On the other hand, in the second phase, also called the inflammatory phase, the nociceptive effects result from the synthesis and release of inflammatory mediators at the site of formalin injection [19,20,21]. Thus, the experimental data observed for series **10a**–**m**/**11a**–**m** can lead us to infer that some of the compounds may be acting, in some way, to reduce or inhibit the neurogenic response. It could occur due to the inhibition of nociceptors or other receptors responsible for the nociceptive response, such as opioid, substance P, and kinin receptors. We can also suggest that some of the compounds can present anti-inflammatory effects since they significantly reduced the second phase of the model, particularly PQM-292 (**11d**). This effect could occur through reduction or inhibition in the formation and/or liberation of a diversity of inflammatory mediators (i.e., prostaglandins and leukotrienes or other inflammatory mediators, such as bradykinin, histamine, and serotonin, as well as cytokines, kinins, glutamate, and nitric oxide) [21,22].

**Figure 5:**
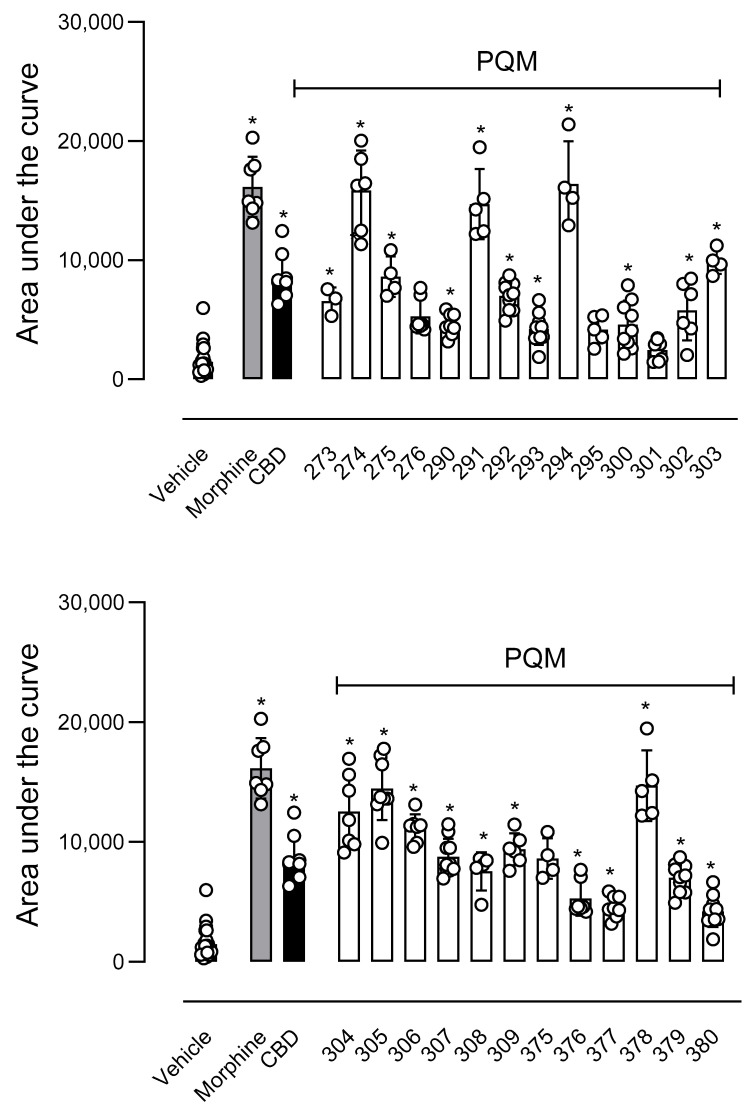
Effects of compounds in the thermal-induced nociception (hot plate model). Animals were orally pretreated with morphine (9 µmol/kg), compounds (10 µmol/kg), or vehicle. The results are presented as mean ± SD. (*n* = 7–10 per group). The area under the curve was calculated by GraphPad Prism Software 10.1.2. One-way or two-way analysis of variance (ANOVA) followed by Tukey’s post hoc test was used for unpaired data when more than two groups were compared to the same control. The post hoc tests were run only if F achieved the necessary level of statistical significance. * When *p* was lower than 0.05, group differences were considered significant.

In the next step, we evaluated the antinociceptive activity of compounds against thermally induced nociception in the hot plate model. In this assay, the animal is positioned in a warmed plate (55 °C), and the temperature activates nociceptors located in the mouse paw, transmitting acute nociceptive information to specific regions of the Central Nervous System and, in turn, producing an organized response that result in an elevation of motor response and/or paw licking [23]. As a result, only PQM-295 (**10e**) and PQM-301 (**10f**) did not show a central antinociceptive effect, as depicted by the increased area under the curve (AUC) in Figure 5. Conversely, the compounds PQM-274 (**11a**), PQM-291 (**10c**), PQM-294 (**11e**), PQM-304 (**11h**), PQM-305 (**10h**), and PQM-378 (**11l**) exhibited potent antinociceptive effects similar to those observed for the control group of morphine.

### 2.2. Molecular Docking

**Figure 6:**
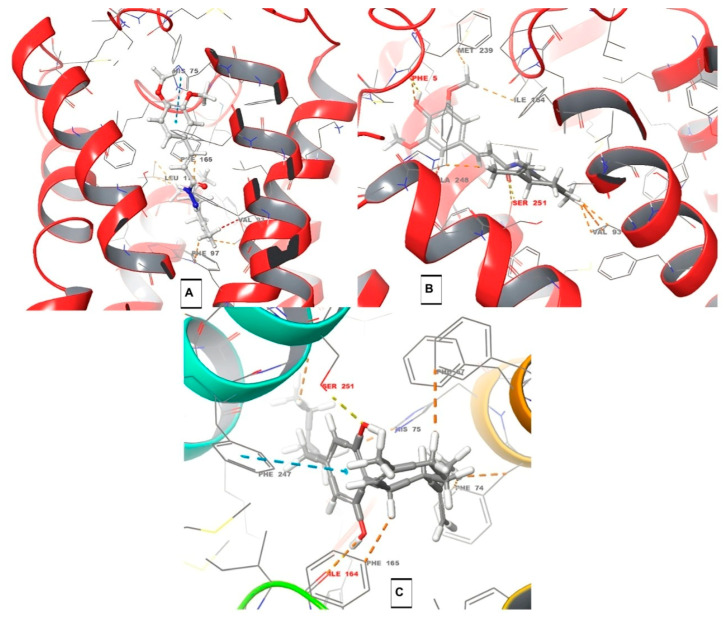
Docking results for CB1 receptor. For the PQM compounds: C atoms are represented in grey, O atoms in red, H atoms in white, and N atoms in blue. (**A**) Interactions of compound PQM-275 (**10b**) with residues of HIS-75, PHE-97, PHE-165, VAL-93, and LEU-173; (**B**) interactions of PQM-375 (**10k**) with residues of VAL-93, MET-239, ILE-164, ALA-248, SER-251 (H-bond interaction), and PHE-5 (H-bond interaction); and (**C**) interactions of CBD with residues of PHE-67, PHE-74, PHE-165, PHE-247, SER-251 (H-bond interaction), ILE-164, and HIS-75. Structural residues according to Liu et al. [24]: F170^2.57^, 174^2.61^, F177^2.64^, and H178^2.65^. Residues F200^3.36^ and W356^6.48^ also seem to play a role in the activity.

To contribute to a better understanding of possible interactions with cannabinoid receptors potentially involved in the observed antinociceptive properties of the abovementioned compounds, we performed a molecular docking study with compounds **10a**–**m/11a**–**m** towards CB1, CB2, and TRPV1 receptors (Table 1, background color highlights the best results). As depicted in Table 1, compounds PQM-275 (**10b**, ΔG_pred_ = −11.45 kcal/mol, Figure 6A) and PQM-375 (**10k**, ΔG_pred_ = −11.24 kcal/mol, Figure 6B) were predicted to have the best interaction energy and a slight selectivity for the CB1 receptor, yielding results slightly better than those obtained for CBD (ΔG_pred_ = −10.72 kcal/mol, Table 1). However, none of the compounds were found to make significant interactions with relevant amino acid residues of the protein structure, which was also observed for CBD (**C**, Figure 6), as expected, given its known low affinity for CB1 [3].

**Figure 7:**
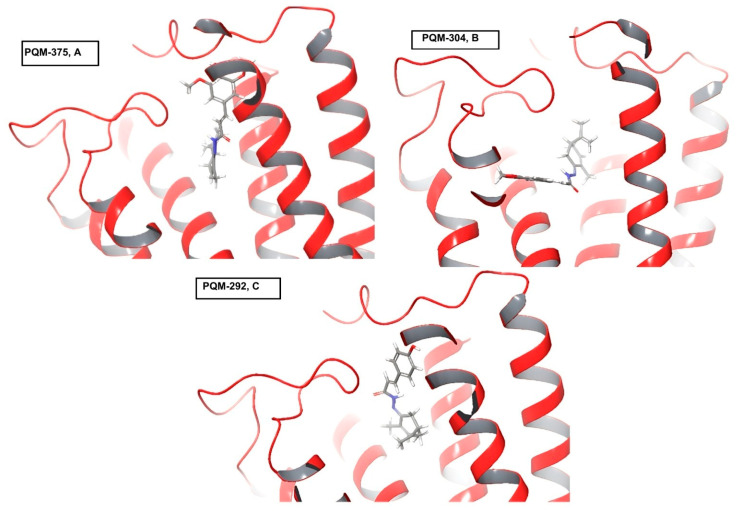
Distinct positions predicted for compounds PQM-375 (**10k**), PQM-304 (**11h**), and PQM-292 (**11d**) in the docking results for CB1. For the PQM compounds: C atoms are represented in grey, O atoms in red, H atoms in white, and N atoms in blue.

Interestingly, the analysis of a possible structure–interaction relationship revealed that most compounds substituted with one or two methoxy groups in the aromatic ring, regardless the stereochemistry at the terpene subunit, such as PQM-275, PQM-276, PQM-375, PQM-376, PQM-294, PQM-295, PQM-300, PQM-307, PQM-308, and PQM-309, as well as the two 3-dimethylamine analogues PQM-377, PQM-378, the 5-hydroxy analogue PQM-303, and PQM-379, which tend to adopt a similar orientation and establish similar interactions, with the terpene subunit facing the interior of the protein cavity, as highlighted for PQM-375 in Figure 7A. In contrast, the 3,4-methylene-dioxy analogue PQM-304 assumed a different orientation, allowing interactions with the terpene subunit facing the exterior of the cavity (Figure 7B). On the other hand, the compounds PQM-273, PQM-274, PQM-290, PQM-291, PQM-380, PQM-292, PQM-293, PQM-301, PQM-302, PQM-305, and PQM-306 were predicted to assume completely different conformation with the terpene subunit twisted to the opposite side of those previously mentioned, as highlighted for PQM-292 in Figure 7C.

**Figure 8:**
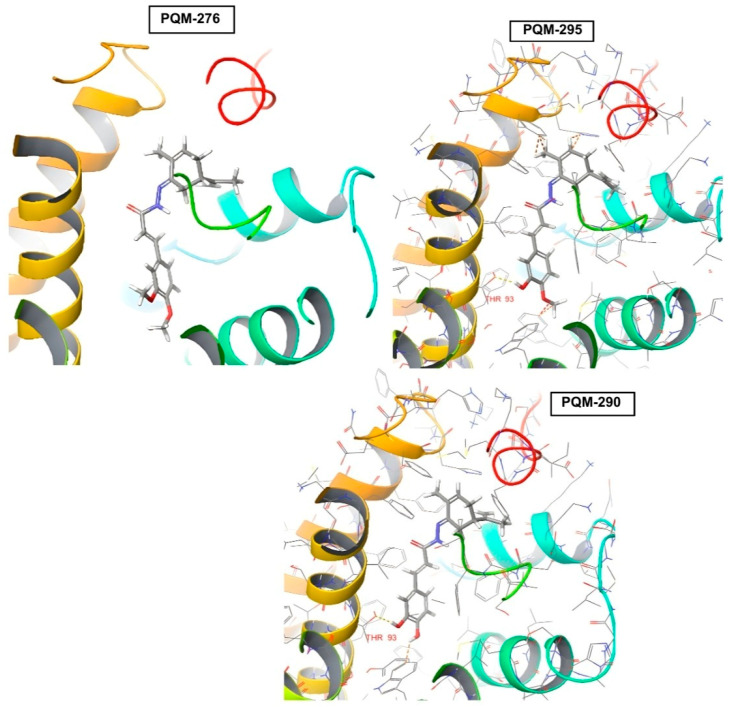
Predicted positions of PQM-276 (**11b**), PQM-295 (**10e**), and PQM-290 (**11c**) in the docking results for CB2 receptor, highlighting H-bond interactions of PQM-295 and PQM-290 with THR93 residue. For the PQM compounds: C atoms are represented in grey, O atoms in red, H atoms in white, and N atoms in blue.

Regarding the CB2 receptor, we identified that, only in the compounds PQM-273, PQM-275, PQM-301, and PQM-307, the terpene subunit was facing the interior of the protein cavity. Furthermore, although few significant interactions were predicted, we observed that the hydroxylated compounds PQM-290 (3,4-di-OH), PQM-302 (5-OH), and PQM-303 (5-OH), as well as the 3-methoxylated analogue PQM-295, were predicted to perform a H-bond interaction with the THR93 residue (Figure 8).

**Figure 9:**
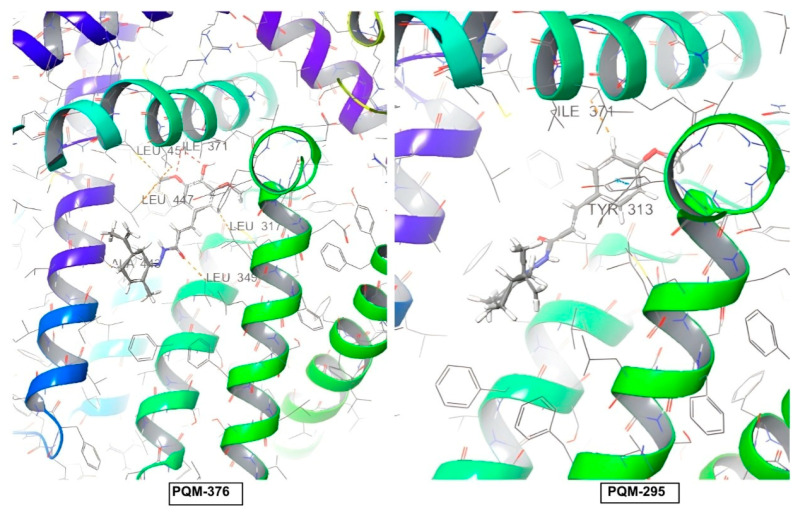
Predicted positions for compounds PQM-376 (**11k**) and PQM-295 (**10e**) in the docking results for the TRPV1 receptor. For the PQM compounds: C atoms are represented in grey, O atoms in red, H atoms in white, and N atoms in blue.

The evaluation of the binding affinity for the TRPV1 receptor revealed that all compounds were predicted with the terpene subunit facing the outside of the protein cavity, as exemplified by PQM-295 and PQM-376 in Figure 9. Once more, as observed for CB2, a few interactions were predicted for TRPV1, and none of these were predicted to interact with the structural residues THR550, SER512, ARG557, TYR511, LEU515, VAL518, MET547, ILE573, and LEU669 located in the vanilloid site, which is described as responsible for the antinociceptive activity [25].

It is worth mentioning that the molecular docking study can contribute to understanding the molecular affinity between the evaluated compounds and the selected targets. In vitro and in vivo studies are necessary for a better discussion of the observed data.

### 2.3. ADME Properties

All compounds were evaluated in silico for their ADME properties by using the QikProp V.3.5 (Schrödinger) software (Table 2, the best results are highlighted in bold). The top-five most active compounds, PQM-292, PQM-293, PQM-295, PQM-307, PQM-308, and PQM-309, in the first phase of the formalin test, in the second phase (PQM-292), as well as PQM-274, PQM-291, PQM-294, PQM-304, PQM-305, and PQM-378, which exhibited the best results in the hot plate model (all highlighted in Table 2), were predicted to have moderate blood–brain barrier (BBB) permeability, but were also predicted to be inactive in the CNS, similarly to CBD. In addition, QPPCaco values indicated good intestinal absorption, as well as moderate lipophilicity (QPlogPo/w), ability to bind human serum albumin (QPLogKHSA), and cellular permeability (MDCK cells). Despite all compounds seeming to violate Jorgensen’s rule of 3, none of them showed a violation number higher than 1 related to Lipinski’s rule, which could indicate moderate to good oral bioavailability and adequate drug-like properties for further development.

## 3. Discussion

In this work, to the best of our knowledge, we report for the first time a series of CBD-based *N*-acyl-hydrazones with potential antinociceptive and anti-inflammatory activity. Twenty-six compounds were synthesized in a two-step synthetic route from the natural monoterpene *R*- and *S*-carvones, coupled to diverse cinnamic acid-derived hydrazides, in moderate yields. All compounds were screened for their in vivo antinociceptive and anti-inflammatory effects at a fixed dose of 10 µmol/kg on the two classical formalin and hot plate models. In the neurogenic phase of the formalin test, it was evidenced that PQM-292 (**11d**), PQM-293 (**10d**), PQM-295 (**10e**), PQM-307 (**10i**), PQM-308 (**11j**), and PQM-309 (**10j**) were the most active compounds, exhibiting a comparable antinociceptive profile to morphine. The literature data suggest that treatment with CBD for chronic injury in mice or rats can be performed with doses of from 5 to 20 mg/kg [26,27,28], resulting in significant antinociceptive effects. Therefore, it seems clear that those six aforementioned compounds, especially PQM-292 and PQM-293, demonstrated promising antinociceptive activity, surpassing CBD, and being comparable to morphine at a low dose. Notably, in the anti-inflammatory phase of the formalin assay [19], PQM-292 exhibited remarkable activity, showing better results than the control groups treated with morphine and ASA. Interestingly, studies investigating CBD’s effect on inflammatory pain have demonstrated its antiallodynic effect at 2.5 mg/kg (i.p.), with no differences observed between male and female animals [29]. Thus, our results suggest that PQM-292 may act through a different mechanism of action than its analogues, exhibiting both antinociceptive and anti-inflammatory activities.

In the hot plate model, we observed that the compounds PQM-274 (**11a**), PQM-291 (**10c**), PQM-294 (**11e**), PQM-304 (**11h**), PQM-305 (**10h**), and PQM-378 (**11l**) exhibited antinociceptive effects similar to those observed for the morphine-treated group. Notably, the compound PQM-274 (**11a**) exhibited an almost equivalent effect to that of morphine. In the literature, CBD has been reported to exhibit similar antinociceptive effects at doses of 3 and 30 mg/kg. On the other hand, considering that the hot plate model is based on an acute response to intense and short-term thermal stimuli, whereas the formalin test involves induced persistent noxious stimulation, studies suggest that the type of pain model used can influence behavioral responses [30]. This could potentially explain the differences observed in the effect of the most active compounds in both animal models, and it is reasonable to consider that these compounds could act through different mechanisms related to the modulation of TRPA1 receptor in the formalin test [31], and TRPV1 in the hot plate assay [32].

Regarding the possible molecular mechanisms underlying the antinociceptive activity of the evaluated compounds, it is known that CBD interacts directly and indirectly with the ECS, but with relatively low affinity for CB1 and CB2. These two primary cannabinoid receptors play crucial roles in modulating pain. CB1 receptors are predominantly expressed in the central nervous system (CNS); their activation leads to inhibition of neurotransmitter release, particularly glutamate and substance P, thus reducing nociceptive signal transmission; and their activation is associated with centrally mediated antinociceptive effects, but also with psychotropic side effects (e.g., euphoria, cognitive impairment). CB2 receptors are mainly found in peripheral immune cells and, to a lesser extent, in the CNS; their activation results in modulation of inflammatory responses, decreasing the release of pro-inflammatory cytokines and indirectly reducing pain, and their agonists are promising for inflammatory and neuropathic pain, with a lower risk of CNS-related side effects [33,34].

Cannabinoids can act synergistically with opioids and other analgesics: co-administration with opioids may allow for dose reduction, potentially mitigating opioid-related side effects. Evidence suggests crosstalk between CB1 and mu-opioid receptors, possibly through shared intracellular signaling cascades or receptor heteromerization [35]. The principal clinical implications for using cannabinoids are for (1) chronic pain management, for example, cannabinoid-based therapies (e.g., nabiximols, dronabinol) are increasingly used for neuropathic pain, cancer-related pain, and multiple sclerosis-related spasticity; (2) there is increasing use of these substances as opioid-sparing strategies. In this situation, the incorporation of cannabinoids may reduce opioid dependence and tolerance development; (3) treatment of inflammatory conditions, for example, CB2-targeted compounds hold promise for rheumatoid arthritis, IBD, and other inflammatory diseases. However, there are some limitations, such as psychoactive effects, limiting the clinical utility of CB1 agonists; legal and regulatory constraints still impact research and therapeutic use, and limited data exist concerning long-term safety [36].

In comparison to CBD, the most active compounds in the series **10a**–**m**/**11a**–**m** were predicted to exhibit a slight affinity for CB1, even though neither of them showed significant interactions with structural residues associated with the antinociceptive effect [26]. Considering that TRPV1 channels are mainly expressed on unmyelinated C-fibers [27], which are required for the antiallodynic effect of CBD [26], docking studies were also performed against this target to evaluate the binding affinity of the synthetic analogues. Regarding the compound PQM-292 (**11d**), it showed the best antinociceptive effects on both the neurogenic and inflammatory phases of the formalin test, suggesting a promising analgesic and anti-inflammatory profile. Despite the favorable predicted affinities obtained against CB1 (−10.51 kcal/mol), CB2 (−10.37 kcal/mol), and TRPV1 (−9.17 kcal/mol), they were only moderate when compared to other analogs in the series. Notably, several compounds with stronger predicted affinities at these targets did not exhibit comparable in vivo efficacy. These findings suggest that the pharmacological effects of PQM-292 (**11d**) may also involve alternative molecular targets or synergistic mechanisms not captured by the current docking study, highlighting the importance of further experimental validation to elucidate its mode of action.

Considering the in vivo results and the computational data, a structure–activity relationship analysis suggested that the stereochemistry of the terpene moiety is not crucial for biological activity and does not influence the docking position of the ligands at the protein cavity on CB1, CB2, or TRPV1 receptors. On the other hand, the presence of methoxy or dialkylamine groups, rather than H-bond donors, such as hydroxyl substituents, on the aromatic ring, appears to induce a similar orientation of the ligands within the CB1 cavity. Additionally, hydroxy substituents at the C3, C4, or C5 positions were observed to favor a conformational change in which the terpene moiety twists to the opposite side compared to other analogues. Moreover, the hydroxyl group at the C3 position, when present as the sole substituent in the aromatic ring, was crucial for enhancing the antinociceptive activity, as seen in the most active compound PQM-292 (**11d**).

Due to the diverse in vivo pharmacological profile observed for several compounds when their effects on the formalin test and hot plate were compared, and between the two phases in the formalin test, particularly for compound PQM-292, which showed significand anti-nociceptive effects on both neurogenic and inflammatory phases, further molecular studies are being conducted by our group. Our goal is that, in the near future, we could contribute to a better understanding of the possible mechanisms of action underlying the different antinociceptive profiles observed. Moreover, our ongoing studies could also contribute to the discovery of novel, promising lead compounds, with diverse analgesic and/or anti-inflammatory properties, without central adverse effects, that could be useful to the development of improved therapeutic alternatives for chronic inflammation and neuropathic pain.

## 4. Materials and Methods

### 4.1. General Experimental Methods

NMR spectra were obtained from a BRUKER AVANCE DRX 300 MH_Z_ spectrometer. IR spectra were generated in a Nicolet iS50 FTIR (Thermo Scientific, Waltham, MA, USA) spectrometer, coupled with Pike Gladi ATR Technologies. ^1^H (300 MH_Z_) and ^13^C (75 MH_Z_) NMR chemical analyses were reported in parts per million (*δ*) relative to tetramethylsilane (0.00 ppm) or other deuterated solvent (CDCl_3_ or DMSO-*d*6) as an internal standard. Coupling constants (*J*) were reported in hertz (H_Z_) and were obtained by MestreReNova^©^ software (version 6.0.2-5475, Mestrelab Research S.L., Santiago de Compostela, Spain). Abbreviations of multiplicity were as follows: s: singlet, d: doublet, t: triplet, q: quartet, m: multiplet. Data were presented as multiplicity, integration, and coupling constant. Analytical thin layer chromatography (TLC) experiments were performed on Merck silica gel 60 F254 plates, eluted in hexane/ethyl acetate in concentration gradient, and visualized under UV light (256 nm) or chemical reaction (e.g., I_2_). Normal-phase column chromatography was performed on Sigma-Aldrich silica gel or in an Isolera automatic chromatograph (Biotage). The commercial substituted cinnamic acids, *R-* and *S*-carvone, were without further purification. The solvents were treated and distilled by conventional methods.

#### 4.1.1. Synthesis of Hydrazide Intermediates (**8a**–**m**)

To a solution of the corresponding cinnamic acid (1.83 mmol, **7a**–**m**, 1.0 eq), in 8 mL of MeCN, was added EDAC hydrochloride (1.2 eq) and HOBt (1.2 eq). The reaction mixture was stirred at room temperature for 1.5 h, with the formation of a precipitate. Next, the reaction mixture was cooled to 0 °C, followed by drop-by-drop addition of a solution of hydrazine monohydrate (10 eq) in 3 mL of MeCN (0 °C). The final reaction mixture was kept under stirring and at room temperature for an additional 45 min, when TLC analysis indicated the reaction completion. Then, the solvent was removed under pressure, followed by the addition of NaHCO_3_ 10% (2 mL) to precipitate the desired hydrazides. The crude products were filtered off and washed with distilled H_2_O (3x). The pure products were obtained as solids in 20–100% yields.
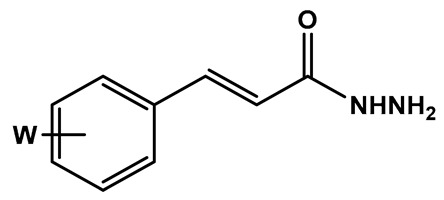

(*E*)-3-(4-hydroxy-3-methoxyphenyl) acrylohydrazide **(8a)**
MW: 208.21 g/mol. Chemical Formula: C_10_H_12_N_2_O_3_. Physical appearance: yellow solid. yield: 53%. IR (ATR, *v*_max_, cm^−1^): 3278 e 3202 (ν_as/s_ R-NH_2_), 1655 (ν RHC=CHR), 1585 (ν C=O), 1518 (*δ* NH), 1466 e 1427 (*δ*_as/s_ CH_3_), 1033 (ν Ar-O-C), 961 (*δ* RHC=CHR), 835 e 810 (*δ* C-H_ar_), 714 (*δ* NH). ^1^H NMR (300 MHz, DMSO-*d*_6_), *δ* (ppm): 9.47 (s, 1H, H11), 9.20 (s, 1H, H12), 7.34 (d, *J* = 15.7 Hz, 1H, H7), 7.11 (s, 1H, H5), 6.99 (d, *J* = 9.8 Hz, 1H, H1), 6.78 (d, *J* = 8.1 Hz, 1H, H2), 6.36 (d, *J* = 15.7 Hz, 1H, H8), 4.41 (s, 2H, H13), 3.79 (s, 3H, H10); ^13^C NMR (75 MHz, DMSO-*d*_6_) *δ* (ppm): 165.6 (C9), 148.7 (C3), 148.3 (C4), 139.1 (C7), 126.8 (C6), 121.8 (C1), 117.3 (C8), 116.1 (C2), 111.3 (C5), 56.0 (C10).
(*E*)-3-(3,4-dimethoxyphenyl)acrylohydrazide **(8b)**
MW: 222.24 g/mol. Chemical Formula: C_11_H_14_N_2_O_3_. Physical appearance: yellow solid. yield: 77%. IR (ATR, *v*_max_, cm^−1^): 3322 and 3228 (ν_as/s_ R-NH_2_), 3013 (ν =CH), 2996 (ν_as_ CH_3_), 1660 (ν C=C), 1651 (ν C=O), 1506 (*δ* NH), 1464 and 1452 (*δ* CH_3_), 1259 (ν_as_ =Ar-O-C), 1016 (ν_as_ Ar-O-C), 964 (*δ* CH). ^1^H NMR (300 MHz, DMSO-*d*_6_), *δ* (ppm): 9.27 (s, 1H), 7.38 (d, *J* = 15.8 Hz, 1H, H7), 7.14 (d, *J* = 1.9 Hz, 1H, H5), 7.11 (dd, *J* = 8.35 Hz, *J* = 1.9 Hz, 1H, H1), 6.97 (d, *J* = 15.8 Hz, 1H, H2), 6.44 (d, *J* = 15.77 Hz, 1H, H8), 4.42 (s, 2H, H13), 3.78 (s, 3H, H11), 3.77 (s, 3H, H10); ^13^C NMR (75 MHz, DMSO-*d*_6_) *δ* (ppm): 165.4 (C9), 150.2 (C4), 149.4 (C3), 138.8 (C7), 128.2 (C6), 121.7 (C8), 118.4 (C1), 112.2 (C2), 110.6 (C5), 56.0 (C10/11).
(*E*)-3-(3,4-dihydroxyphenyl)acrylohydrazide **(8c)**
MW: 194.19 g/mol. Chemical Formula: C_9_H_10_N_2_O_3_. Physical appearance: yellow solid. yield: 20%. IR (ATR, *v*_max_, cm^−1^): 3446 (ν OH), 3341 and 3312 (ν_as/s_ NH_2_), 2928 and 2867 (ν_as/s_ CH), 1688 (ν C=O), 1637 (ν C=C), 1590 (*δ* NH), 1258 (*δ* OH), 1037 (ν C-OH). ^1^H NMR (300 MHz, DMSO-*d*_6_), *δ* (ppm): 9.2 (s, 1H, H12); 7.25 (d, *J* = 15.7 Hz, 1H, H7), 6.93 (d, *J* = 1.74 Hz, 1H, H5), 6.82 (dd, *J* = 8.1 Hz, *J* = 1.75 Hz, 1H, H1), 6.73 (d, *J* = 8.1 Hz, 1H, H2), 6.24 (d, *J* = 15.7 Hz, 1H, H8), 4.37 (s, 2H, H13); ^13^C NMR (75 MHz, DMSO-*d*_6_) *δ* (ppm): 165.1 (C5), 147.3 (C11), 145.6 (C10), 138.7 (C7), 126.3 (C6), 120.4 (C8), 116.4 (C1), 115.7 (C2), 113.6 (C5).
(*E*)-3-(4-hydroxyphenyl)acrylohydrazide **(8d)**
MW: 178.19 g/mol. Chemical Formula: C_9_H_10_N_2_O_2_. Physical appearance: yellow solid. yield: 27%. IR (ATR, *v*_max_, cm^−1^): 3325 and 3269 (ν_as/s_ NH_2_), 3196 (ν CH), 1654 (ν C=C), 1608 (ν C=O), 1511 (*δ* NH), 1035 (ν C-OH), 825 (*δ* C-H ar. 1,4 dissubstituted). ^1^H NMR (300 MHz, DMSO-*d*_6_) *δ* (ppm): 9.21 (s, 1H, H11), 7.38 (d, *J* = 8.6 Hz, 2H, H1; H5), 7.31 (s, 1H, H7), 6.78 (d, *J* = 8.60 Hz 2H, H2; H4), 4.40 (s, 2H, H12); ^13^C NMR (75 MHz, DMSO-*d*_6_) *δ* (ppm): 165.2 (C9), 158.9 (C10), 138.4 (C7), 129.2 (C1/5), 126.6 (d, *J* = 15.8 Hz, 1H, H8), 116.7 (C8), 115.8 (C2/4).
(*E*)-3-(4-methoxyphenyl)acrylohydrazide **(8e)**
MW: 192.21 g/mol. Chemical Formula: C_10_H_12_N_2_O_2_. Physical appearance: white solid. yield: 59%. IR (ATR, *v*_max_, cm^−1^): 3309 and 3278 (ν_as/s_ NH_2_), 3012 (ν =CH-), 2952 e 2835 (ν_as/s_ CH_3_), 1655 (ν C=C), 1602 (ν C=O), 1521 (*δ* NH), 1462 e 1441 (δ_as/s_ CH_3_), 965 (*δ* =CH) 820 (*δ* CH_ar_). ^1^H NMR (300 MHz, CDCl_3_) *δ* (ppm): 7.64 (d, *J* = 15.6 Hz, 1H, H7), 7.50 (s, 1H, H11), 7.42–7.47 (m, 2H, H1; H5), 6.86–6.90 (m, 2H, H2; H4), 6.23 (d, *J* = 15.6 Hz, 1H, H8), 3.82 (s, 3H, H10); ^13^C NMR (75 MHz, CDCl_3_) *δ* (ppm): 167.5 (C9), 161.1(C3), 141.5 (C7), 129.5 (C1/5), 127.3 (C6), 115.3 (C8), 114.3 (C2/4), 55.4 (C10).
(*E*)-3-(4-(trifluoromethyl)phenyl)acrylohydrazide **(8f)**
MW: 230.19 g/mol. Chemical Formula: C_10_H_9_F_3_N_2_O. Physical appearance: white solid. yield: 70%. IR (ATR, *v*_max_, cm^−1^): 3441 and 3213 (ν_as/s_ NH_2_), 3316 (ν NH), 1646 (ν C=C), 1610 (ν C=O), 1531 (*δ* NH), 1317 (ν CF_3_), 836 (*δ* CH_ar_). ^1^H NMR (300 MHz, DMSO-*d*_6_) *δ* (ppm): 7.76 (s, 4H, H1; H2; H4; H5), 7.51 (d, *J* = 15.9 Hz, 1H, H7), 6.67 (d, *J* = 15.9 Hz, 1H, H8), 4.55 (s, 2H, H12); ^13^C NMR (75 MHz, DMSO-*d*_6_) *δ* (ppm): 163.8 (C9), 139.0 (C7), 136.6 (C6), 128.1 (C1/2/4/5), 125.8 (C3), 123.1 (C8).
(*E*)-3-(2-hydroxyphenyl)acrylohydrazide **(8g)**
MW: 178.19 g/mol. Chemical Formula: C_9_H_10_N_2_O_2_. Physical appearance: brown solid. yield: 73%. IR (ATR, *v*_max_, cm^−1^): 3324 and 3296 (ν_as/s_ NH_2_), 3173 (ν NH), 3059 and 2969 (ν CH), 1643 (ν C=O), 1585 (*δ* NH_2_), 1526 (*δ* NH), 1343 and 1255 (*δ* OH + ν =C-O), 760 (*δ* CH_ar_). ^1^H NMR (300 MHz, DMSO-*d*_6_) *δ* (ppm): 9.29 (s, 1H, H11), 7.64 (d, *J* = 15.9 Hz, 1H, H7), 7.40 (d, *J* = 7.55 Hz, *J* = 1.5 Hz, 1H, H1), 7.16 (t, 1H, H3), 6.88 (d, *J* = 8.1 Hz, 1H, H4), 6.88 (d, *J* = 8.1 Hz, 1H, H2), 6.59 (d, *J* = 15.9 Hz, H8), 4.41 (s, 2H, H12); **^13^C** (75 MHz, DMSO-*d*_6_) *δ* (ppm): 165.3 (C9), 156.3 (C1), 134.2 (C7), 130.4 (C4), 128.2 (C2), 121.7 (C6), 119.7 (C8), 119.3 (C5), 116.1 (C3).
(*E*)-3-(benzo[d][1,3]dioxol-5-yl)acrylohydrazide **(8h)**
MW: 206.20 g/mol. Chemical Formula: C_10_H_10_N_2_O_3_. Physical appearance: brown solid. yield: 59%. IR (ATR, *v*_max_, cm^−1^): 3314 (νNH), 3033 (ν =CH), 1661 (ν C=C), 1608 (ν C=O), 1450 (*δ*_s_ CH_2_), 1258 (ν_as_ C-O-C), 924 (*δ* CH_2_). ^1^H NMR (300 MHz, DMSO-*d*_6_) *δ* (ppm): 9.25 (s, 1H, H11), 7.35 (d, *J* = 15.75 Hz, 1H, H7), 7.13 (d, *J* = 1.4 Hz, 1H, H5), 7.06 (dd, *J* = 8.1 Hz, *J* = 1.4 Hz, 1H, H1), 6.94 (d, *J* = 8.1 Hz, 1H, H4), 6.38 (d, *J* = 15.75 Hz, 1H, H8), 6.05 (s, 2H, H10), 4.42 (s, 2H, H12); **^13^C** (75 MHz, DMSO-*d*_6_) *δ* (ppm): 164.8 (C9), 148.4 (C3), 148.0 (C2), 138.0 (C7), 129.3, (C6), 123.2 (C1), 118.4 (C8), 108.6 (C5), 106.2 (C4), 101.5 (C10).
(*E*)-3-(4-chlorophenyl)acrylohydrazide **(8i)**
MW: 196.63 g/mol. Chemical Formula: C_9_H_9_ClN_2_O. Physical appearance: white solid. yield: 100%. IR (ATR, *v*_max_, cm^−1^): 3274 (ν NH_2_), 3034 (ν =CH), 1661 (νC=C), 1629 (ν C=O), 1558 (*δ* NH), 1035 (C-Cl), 969 (*δ* HC=CH) 819 (*δ* CH_ar_). ^1^H NMR (300 MHz, DMSO-*d*_6_) *δ* (ppm): 9.52 (s, 1H, H11), 7.56–7.60 (m, 2H, H1/5), 7.45–7.48 (m, 2H, H2/4), 7.40 (s, 1H, H7), 6.55 (d, *J* = 15.9 Hz, 1H, H8); ^13^C NMR (75 MHz, DMSO-*d*_6_) *δ* (ppm): 164.2 (C9), 136.8 (C7), 134.3 (C3/C6), 129.6 (C1/5), 129.4 (C2/4), 121.1 (C8).
(*E*)-3-(3-hydroxy-4-methoxyphenyl)acrylohydrazide **(8j)**
MW: 208.21 g/mol. Chemical Formula: C_10_H_12_N_2_O_3_. Physical appearance: yellow solid. yield: 75%. IR (ATR, *v*_max_, cm^−1^): 3333 (νNH_2_), 3154 (ν NH), 1651 (ν C=O), 1594 (*δ* NH), 1495 (ν C=C_ar_), 1443 and 1364 (*δ* CH_3_), 995 (*δ* HC=CH). ^1^H NMR (300 MHz, DMSO-*d*_6_) *δ* (ppm): 9.26 (s, 1H, H10), 9.23 (s, 1H, H12), 7.30 (d, *J* = 15.74 Hz, 1H, H7), 6.98 (s, 1H, H5), 6.90–6.94 (m, 2H, H2; H1), 6.31 (d, *J* = 15.74 Hz, 1H, H8), 4.43 (s, 2H, H13), 3.78 (s, 3H, H11); ^13^C NMR (75 MHz, DMSO-*d*_6_) *δ* (ppm): 165.2 (C9), 149.4 (C10), 146.9 (C3), 138.6 (C7), 128.0 (C6), 120.6 (C1), 117.8 (C8), 113.4 (C2), 112.3 (C5), 58.2 (C11).
(*E*)-3-(4-hydroxy-3,5-dimethoxyphenyl)acrylohydrazide **(8k)**
MW: 238.24 g/mol. Chemical Formula: C_11_H_14_N_2_O_4_. Physical appearance: yellow solid. yield: 62%. IR (ATR, *v*_max_, cm^−1^): 3333 (νNH_2_), 3154 (ν NH), 1651 (ν C=O), 1594 (*δ* NH), 1495 (ν C=C_ar_), 1443 and 1364 (*δ* CH_3_), 995 (*δ* HC=CH). ^1^H NMR (300 MHz, DMSO-*d*_6_) *δ* (ppm): 9.18 (s, 1H, H13), 7.35 (d, *J* = 15.7 Hz, 1H, H7), 6.83 (s, 2H, H1; H5), 6.40 (d, *J* = 15.7 Hz, 1H, H8), 4.40 (s, 2H, H14), 3.78 (s, 6H, H10; H12); ^13^C NMR (75 MHz, DMSO-*d*_6_) *δ* (ppm): 165.1 (C9), 148.1 (C2/C4), 139.0 (C7), 137.3 (C3), 125.2 (C6), 117.3 (C8), 105.3 (C1/C5), 56.0 (C10/C12),
(*E*)-3-(4-(dimethylamino)phenyl)acrylohydrazide **(8l)**
MW: 205.26 g/mol. Chemical Formula: C_11_H_15_N_3_O. Physical appearance: yellow solid. yield: 100%. IR (ATR, *v*_max_, cm^−1^): 3333 (νNH_2_), 3154 (ν NH),1651 (ν C=O), 1594 (*δ* NH), 1495 (ν C=C_ar_), 1443 and 1364 (*δ* CH_3_), 995 (*δ* HC=CH). ^1^H NMR (300 MHz, C*D*Cl_3_) *δ* (ppm): 7.61 (d, *J* = 15.4 Hz, 1H, H7), 7.40 (d, *J* = 8.7 Hz, 2H, H1; H5), 6.99 (s, 1H, H11), 6.66 (d, *J* = 8.7 Hz, 2H, H2; H4), 6.15 (d, *J* = 15.4 Hz, 1H, H8), 3.00 (s, 6H, H10); ^13^C NMR (75 MHz, C*D*Cl_3_) *δ* (ppm): 168.2 (C9), 151.6 (C3), 142.2 (C7), 129.4 (C1/C5), 122.4 (C6), 112.4 (C8), 111.9 (C2/C4), 40.2 (C10).
Cinnamoylhydrazide **(8m)**
MW: 162.19 g/mol Chemical Formula: C_9_H_10_N_2_O Physical appearance: white solid. yield: 73%. IR (ATR, *v*_max_, cm^−1^): 3333 (νNH_2_), 3154 (ν NH),1651 (ν C=O), 1594 (*δ* NH), 1495 (ν C=C_ar_), 1443 and 1364 (*δ* CH_3_), 995 (*δ* HC=CH). ^1^H NMR (300 MHz, C*D*Cl_3_) *δ* (ppm): 8.78 (s, 1H, H10), 7.80 (d, *J* = 15.9, 1H, H7), 7.31–7.61 (m, 6H, H1, H2, H3, H4, H5 e H8); ^13^C NMR (75 MHz, C*D*Cl_3_) *δ* (ppm): 167.3 (C9), 143.4 (C7), 132.3 (C6), 129.9 (C3), 128.8 (C2/C4), 128.3 (C1/C5), 116.9 (C8).

#### 4.1.2. Synthesis of Terpene-cinnamoyl-N-acyl-hydrazones (**10a**–**m** and **11a**–**m**)

Freshly distilled enantiomeric pure *R*- or *S*-carvone (1.28 mmol) was diluted in anhydrous MeOH (5 mL), followed by the addition of glacial AcOH (10–15 drops), and stirred for 2 min. Next, a solution of the hydrazide intermediates (**8a**–**m**, 1 eq.) in dry MeOH (3 mL) was added to the reaction flask, and the reaction mixture was kept under stirring and at room temperature to completion (TLC). Then, the solvent was removed under low pressure, and the solid material was filtered off or precipitated with ice-cold MeOH, followed by washing with cold water and cold MeOH (3×, 5 mL each) to remove the unreacted carvone amount. The pure products were obtained as solids in 17–79% yields.
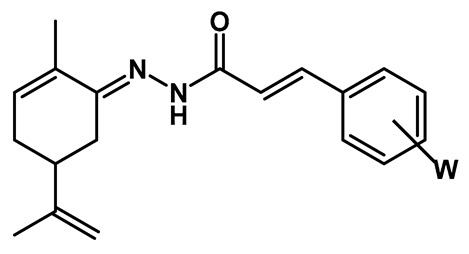

(*E*)-3-(4-hydroxy-3-methoxyphenyl)-*N*’-((*R*,*E*)-2-methyl-5-(prop-1-en-2-yl)cyclohex-2-en-1-ylidene)acrylohydrazide **(**PQM-273, **10a)**
MW: 340.42 g/mol. Chemical Formula: C_20_H_24_N_2_O_3_. Physical appearance: pale yellow solid. Melting range: 215–216 °C. Purity: 100% (HPLC). ${\left[\alpha \right]}_{D}^{25}$ = −0.07 (Yield: 18%. IR (ATR, *ν*_max_, cm^−1^): 3280 (ν NH), 3055 (ν_as_ CH_2_), 2969 (ν_s_ CH_2_ ou ν_as_ CH_3_), 2919 (ν_s_ CH_2_), 2834 (ν_s_ CH_3_), 1661 (ν C=O), 1635 (C=N), 1515 (*δ* NH), 1251 (ν_as_ Ar-O-C), 1028 (ν_s_ Ar-O-C), 978 (*δ* C-H). ^1^H NMR (300 MHz, CDCl_3_), *δ* (ppm): 8.73 (s, 1H, H12), 7.75 (d, *J* = 15.9 Hz, 1H, H7), 7.40 (d, *J* = 15.9 Hz, 1H, H8), 7.17 (d, *J* = 6.92 Hz, 1H, H16), 7.08 (s, 1H, H5), 6.92 (d, *J* = 8.14 Hz, 1H, H2), 6.16 (d, *J* = 8.14 Hz, 1H, H15), 5.92 (s, 1H, H11), 4.82 (d, *J* = 13.0 Hz, 2H, H20), 3.93 (s, 3H, H10), 2.74 (dd, *J* = 4.0 Hz, *J* = 15.3 Hz 1H, H17), 2.29–2.45 (m, 2H, H17; H16), 2.07–2.18 (m, 2H, H18; H16), 1.97 (s, 3H, H21), 1.78 (s, 3H, H22); ^13^C NMR (75 MHz, CDCl_3_), *δ* (ppm): 167.8 (C9), 149.2 (C13), 147.7 (C3), 147.3 (C4), 146.6 (C19), 143.5 (C7), 133.0 (C15), 132.7 (C14), 127.9 (C6), 122.6 (C1), 114.7 (C2), 114.2 (C8), 110.4 (C20), 110.1 (C5), 55.9 (C10), 40.6 (C17), 30.0 (C18), 28.5 (C16), 20.8 (C22), 17.8 (C21). HRMS(ESI): *m*/*z* [M + H]^+^ calcd for C_20_H_24_N_2_O_3_ 340.1787 found [M + H]^+^ 341.18571.
(*E*)-3-(4-hydroxy-3-methoxyphenyl)-*N*’-((*S*,*E*)-2-methyl-5-(prop-1-en-2-yl)cyclohex-2-en-1-ylidene)acrylohydrazide **(**PQM-274, **11a)**
MW: 340.42 g/mol. Chemical Formula: C_20_H_24_N_2_O_3_. Physical appearance: yellow solid. Melting range: 209–201 °C. Purity (HPLC): 100%. ${\left[\alpha \right]}_{D}^{25}$ = +0.08. Yield: 57%. IR (ATR, *v*_max_, cm^−1^): 3274 (ν NH), 2967 (ν_s_ =CH_2_ ou ν_as_ CH_3_), 2918 (ν_s_ =CH_2_), 1654 (ν C=O), 1616 (C=N), 1588 (*δ* NH), 1508 (ν C=C_ar_)1270 (ν_as_ A-O-C), 1030 (ν_s_ Ar-O-C). ^1^H NMR (300 MHz, CDCl_3_), *δ* (ppm): 9.24 (s, 1H, H12), 7.74 (d, *J* = 15.9 Hz, 1H, H7), 7.42 (d, *J* = 15.9 Hz, 1H, H8), 7.16 (dd, *J* = 8.2 Hz, *J* = 1.4 Hz, 1H, H16), 7.08 (d, *J* = 1.4 Hz, 1H, H5), 6.94 (d, *J* = 8.2 Hz, 1H, H2), 6.16 (d, *J* = 5.2 Hz, 1H, H15), 6.06, (s, 1H, H11), 4.83 (d, *J* = 4.3 Hz, 2H, H20), 3.93 (s, 3H, H10), 2.83 (dd, *J* = 3.4 Hz, *J* = 16.1 Hz, 1H, H18), 2.45 (dt, *J* = 4.0 Hz, *J* = 11.9 Hz 1H, H17), 2.33 (dt, *J* = 5.0 Hz, *J* = 17.0 Hz 1H, H16), 2.05–2.19 (m, 2H, H18; H16), 1.97 (s, 3H, H21), 1.79 (s, 3H, H22); ^13^C NMR (75 MHz, CDCl_3_), *δ* (ppm): 167.8 (C9), 149.5 (C13), 147.7 (C3), 147.4 (C4), 146.6 (C19), 143.4 (C7), 133.0 (C15), 132.7 (C14), 127.9 (C6), 122.6 (C1), 114.8 (C2), 114.3 (C8), 110.4 (C20), 110.1 (C5), 55.9 (C10), 40.6 (C17), 30.1 (C18), 28.7 (C16), 20.8 (C22), 17.8 (C21). HRMS(ESI): *m*/*z* [M + H]^+^ calcd for C_20_H_24_N_2_O_3_ 340.1787 found [M + H]^+^ 341.18582.
(*E*)-3-(3,4-dimethoxyphenyl)-*N*’-((*R*,*E*)-2-methyl-5-(prop-1-en-2-yl)cyclohex-2-en-1-ylidene)acrylohydrazide **(**PQM-275, **10b)**
MW: 354.45 g/mol. Chemical Formula: C_21_H_26_N_2_O_3_. Physical appearance: pale yellow solid. Melting range: 246–248 °C. Purity (HPLC): 100%. ${\left[\alpha \right]}_{D}^{25}$ = −0.12. Yield: 71%. IR (ATR, *ν*_max_, cm^−1^): 3158 (ν NH), 3063 (ν_as_ CH_2_), 2988 (ν_s_ CH_2_ ou ν_as_ CH_3_), 2907 (ν_s_ CH_2_), 2834 (ν_s_ CH_3_), 1659 (ν C=O), 1594 (*δ* NH), 1518 (ν C=C_ar_), 1463 e 1443 (*δ*_as/s_ CH_3_), 1253 (ν_as_ Ar-O-C), 1023 (ν_s_ Ar-O-C), 980 (*δ* C-H). ^1^H NMR (300 MHz, CDCl_3_), *δ* (ppm): 9.36 (s, 1H, H12), 7.76 (d, *J* = 15.9 Hz, 1H, H7), 7.46 (d, *J* = 15.9 Hz, 1H, H8), 7.18 (d, *J* = 8.3 Hz, 1H, H1), 7.13 (s, 1H, H5), 6.89 (d, *J* = 8.3 Hz, 1H, H2), 6.16 (d, *J* = 5.3 Hz, 1H, H15), 4.84 (s, 2H, H20), 3.93 (s, 6H, H11; H10), 2.87 (d, *J* = 14.1 Hz, 1H, H18), 2.41 (dt, *J* = 9.2 Hz, *J* = 7.7 Hz 1H, H17), 2.29–2.32 (m, 1H, H16), 2.11–2.18 (m, 2H, H18; H16), 1.97 (s, 3H, H21), 1.80 (s, 3H, H20); ^13^C NMR (75 MHz, CDCl_3_), *δ* (ppm): 168.0 (C9), 150.8 (C3), 149.5 (C13), 149.1 (C4), 143.1 (C7), 133.0 (C14), 132.8 (C15), 128.4 (C6), 122.3 (C1), 114.7 (C2), 111.0 (C8), 110.4 (C5), 110.0 (C20), 56.0 (C11), 55.8 (C10), 40.6 (C17), 30.1 (C18), 28.8 (C16), 20.9 (C20), 17.8 (C21). HRMS(ESI): *m*/*z* [M + H]^+^ calcd for C_21_H_26_N_2_O_3_ 354.1943 found [M + H]^+^ 355.20161.
(*E*)-3-(3,4-dimethoxyphenyl)-*N*’-((*S*,*E*)-2-methyl-5-(prop-1-en-2-yl)cyclohex-2-en-1-ylidene)acrylohydrazide **(**PQM-276, **11b)**
MW: 354.45 g/mol. Chemical Formula: C_21_H_26_N_2_O_3_. Physical appearance: pale yellow solid. Melting range: 239–240 °C. Purity (HPLC): 100%. ${\left[\alpha \right]}_{D}^{25}$ = +0.02 Yield: 83%. IR (ATR, *ν*_max_, cm^−1^): 3158 (ν NH), 3063 (ν_as_ CH_2_), 2988 (ν_s_ CH_2_ ou ν_as_ CH_3_), 2907 (ν_s_ CH_2_), 2834 (ν_s_ CH_3_), 1660 (ν C=O), 1615 (ν C=N), 1595 (*δ* NH), 1518 (ν C=C_ar_), 1463 and 1443 (*δ*_as/s_ CH_3_), 1253 (ν_as_ Ar-O-C), 1023 (ν_s_ Ar-O-C), 981 (*δ* C-H). ^1^H NMR (300 MHz, CDCl_3_), *δ* (ppm): 9.48 (s, 1H, H12), 7.75 (d, *J* = 15.9 Hz, 1H, H7), 7.46 (d, *J* = 15.9 Hz, 1H, H8), 7.18 (d, *J* = 8.2 Hz, 1H, H1), 7.13 (s, 1H, H5), 6.89 (d, *J* = 8.2 Hz, 1H, H2), 6.16 (d, *J* = 4.1 Hz, 1H, H15), 4.85 (s, 2H, H20), 3.93 (s, 6H, H10; H11), 2.88 (dd, *J* = 15.9 Hz, *J* = 3.1 Hz, 1H, H18), 2.45 (t, *J* = 11.9 Hz, 1H, H17), 2.34 (dd, *J* = 18.0 Hz, *J* = 5.0 Hz, 1H, H16), 2.12 (dd, *J* = 15.0 Hz, *J* = 13.4 Hz 2H, H18; H16), 1.97 (s, 3H, H21), 1.81 (s, 3H, H20); ^13^C NMR (75 MHz, CDCl_3_), *δ* (ppm): 168.0 (C9), 150.8 (C3), 149.6 (C13), 149.0 (C4), 143.1 (C7), 133.0 (C14), 132.8 (C15), 128.4 (C6), 122.3 (C1), 114.7 (C2), 111.0 (C8), 110.4 (C5), 110.0 (C20), 56.0 (C11), 55.8 (C10), 40.6 (C17), 30.1 (C18), 28.8 (C16), 20.9 (C20), 17.8 (C21). HRMS(ESI): *m*/*z* [M + H]^+^ calcd for C_21_H_26_N_2_O_3_ 354.1943 found [M + H]^+^ 355.20167.
(*E*)-3-(3,4-dihydroxyphenyl)-*N*’-((*S*,*E*)-2-methyl-5-(prop-1-en-2-yl)cyclohex-2-en-1-ylidene)acrylohydrazide **(**PQM-290, **11c)**
MW: 326.40 g/mol. Chemical Formula: C_19_H_22_N_2_O_3_. Physical appearance: pale yellow solid. Melting range: 195–197 °C. Purity (HPLC): 100%.${\left[\alpha \right]}_{D}^{25}$ = +0.03. Yield: 28%. IR (ATR, *v*_max_, cm^−1^): 3300 (ν OH), 3244 (ν NH), 1648 (ν C=O), 1616 (ν C=N), 1603 (*δ* NH), 1514 (ν C=C_ar_), 1371 e 1120 (*δ* OH and ν =C-O), 979 (*δ* C=CH_2_), 817 (*δ* RC=CH). ^1^H NMR (300 MHz, DMSO-*d*_6_), *δ* (ppm): 10.45–10.41 (s, 1H, H12), 7.37 (dt, *J* = 16.0 Hz *J* = 30.08 Hz, 1H, H7), 7.02 (d, *J* = 13.2 Hz, 1H, H5), 6.91 (t, *J* = 6.8 Hz 1H, H1), 6.73 (dd, *J* = 11.9 Hz and *J* = 16.0 Hz, 2H, H2 e H8), 6.15 (s, 1H, H15), 4.80 (t, *J* = 7.7 Hz, 2H, H20), 2.92 (t, *J* = 12.7 Hz, 1H, H18), 2.21–2.36 (m, 2H, H17; H16), 2.01–2.14 (m, 2H, H18; H16), 1.91–1.83 (s, 3H, H22), 1.76 (s, 3H, H21); ^13^C NMR (75 MHz, DMSO-*d*_6_), *δ* (ppm): 164.7/167.6 (C9,C9’), 149.7/153.5 (C13,C13’), 148.2 (C3), 146.1 (C4 e C19), 141.1/142.8 (C7, C7’), 133.8 (C6), 133.1 (C15), 132.7 (C14), 121.2 (C1), 117.7 (C8), 116.3 (C2), 114.5 (C5), 110.8 (C20), 18.3 (C22), 40.5 (C17), 30.1 (C18), 29.6 (C16), 20.8 (C21). HRMS(ESI): *m*/*z* [M + H]^+^ calcd for C_19_H_22_N_2_O_3_ 326.1630 found [M + H]^+^ 327.17020.
(*E*)-3-(3,4-dihydroxyphenyl)-*N*’-((*R*,*E*)-2-methyl-5-(prop-1-en-2-yl)cyclohex-2-en-1-ylidene)acrylohydrazide **(**PQM-291, **10c)**
MW: 326.40 g/mol. Chemical Formula: C_19_H_22_N_2_O_3_. Physical appearance: yellow solid. Melting range: 204–205 °C. Purity (HPLC): 100%. ${\left[\alpha \right]}_{D}^{25}$ = −0.18. Yield: 34%. IR (ATR, *v*_max_, cm^−1^): 3299 (ν OH), 3243 (ν NH), 2972 (ν_s_ CH_2_ or ν_as_ CH_3_), 1648 (ν C=O), 1616 (ν C=N), 1603 (*δ* NH), 1514 (ν C=C_ar_), 1370 and 1120 (*δ* OH and =C-O), 979 (*δ* C=CH_2_), 816 (*δ* RC=CH). ^1^H NMR (300 MHz, DMSO-*d*_6_), *δ* (ppm): 10.46–10.41 (s, 1H, H12), 9.46 (s, 1H, H11), 9.23 (s, 1H, H10), 7.37 (dt, *J* = 16.2 Hz *J* = 29.7 Hz, 1H, H7), 7.02 (d, *J* = 13.2 Hz, 1H, H5), 6.89–6.93 (m, 1H, H16), 6.74 (dd, *J* = 12.1 Hz and *J* = 16.41 Hz, 2H, H2; H8), 6.15 (s, 1H, H15), 4.80 (t, *J* = 7.2 Hz, 2H, H20), 2.93 (t, *J* = 13.2 Hz, 1H, H18), 2.21–2.36 (m, 2H, H17; H16), 2.03–2.14 (m, 2H, H18; H16), 1.91–1.83 (s, 3H, H22), 1.76 (s, 3H, H21); ^13^C NMR (75 MHz, DMSO-*d*_6_), *δ* (ppm): 162.7/ 167.6 (C9,C9’), 149.7/153.5 (C13,C13′), 148.1 (C3), 146.1 (C4), 146.0 (C19), 141.0/142.8 (C7, C7′), 133.8 (C6), 133.0 (C15), 132.7 (C14), 121.2 (C1), 117.7 (C8), 116.3 (C2), 114.1 (C5), 110.8 (C20), 40.5 (C17), 30.1 (C18), 29.6 (C16), 21.0 (C21), 18.5 (C22). HRMS(ESI): *m*/*z* [M + H]^+^ calcd for C_19_H_22_N_2_O_3_ 326.1630 found [M + H]^+^ 327.17022.
(*E*)-3-(4-hydroxyphenyl)-*N*’-((*R*,*E*)-2-methyl-5-(prop-1-en-2-yl)cyclohex-2-en-1-ylidene)acrylohydrazide **(**PQM-292, **11d)**
MW: 310.40 g/mol. Chemical Formula: C_19_H_22_N_2_O_2_. Physical appearance: pale yellow solid. Melting range: 189–190 °C. Purity: 100%. ${\left[\alpha \right]}_{D}^{25}$ = +0.08. Yield: 21%. IR (ATR, *v*_max_, cm^−1^): 3299 (ν OH), 3066 (ν_as_ CH_2_), 3014 (ν_s_ CH_2_), 2953 and 2922 (ν_as/s_ CH_3_), 1654 (ν C=O), 1622 (ν C=N), 1601 (*δ* NH), 1516 (ν C=C_ar_), 1442 (*δ*_s_ CH_2_), 1375 (*δ*_s_ CH_3_), 1274 (ν C-N), 1200 (ν C-O), 1166 (*δ* OH + =C-O), 974 (*δ* HC=CH), 887 (*δ* =CH), 827 (*δ* CH_ar_). ^1^H NMR (300 MHz, DMSO-*d*_6_), *δ* (ppm): 10.4 (s, 1H, H10), 9.98 (s, 1H, H11), 7.31–7.59 (m, 3H, H1, H5; H7), 6.80 (t, *J* = 10.4 Hz, 3H, H2/H4; H8), 6.15 (s, 1H, H14), 4.80 (t, *J* = 7.65 Hz, 2H, H19), 2.93 (t, *J* = 13.4 Hz, 1H, H15), 2.32 (dd, *J* = 9.7 Hz, *J* = 15.9 Hz, 1H, H16), 2.02–2.22 (m, 3H, H17; H15), 1.85 (s,1H, H21), 1.75 (s, 1H, H20); ^13^C NMR (75 MHz, DMSO-*d*_6_), *δ* (ppm): 167.6 (C9), 159.6 (C3), 153.5 (C12), 133.8 (C14), 133.0 (C13), 129.9 (C1, C5), 126.4 (C6), 117.8 (C8), 116.3 (C2, C4), 110.8 (C19), 40.6 (C16), 30.1 (C17), 29.6 (C15), 20.9 (C20), 18.5 (C21). HRMS(ESI): *m*/*z* [M + H]^+^ calcd for C_19_H_22_N_2_O_2_ 310.1681 found [M + H]^+^ 311.17534.
(*E*)-3-(4-hydroxyphenyl)-*N*’-((*R*,*E*)-2-methyl-5-(prop-1-en-2-yl)cyclohex-2-en-1-ylidene)acrylohydrazide **(**PQM-293, **10d)**
MW: 310.40 g/mol. Chemical Formula: C_19_H_22_N_2_O_2_. Physical appearance: pale yellow solid. Melting range: 227–230 °C. Purity (HPLC): 100%. ${\left[\alpha \right]}_{D}^{25}$ = −0.12. Yield: 17%. IR (ATR, *v*_max_, cm^−1^): 3301 (ν OH), 3067 (ν_as_ CH_2_), 3014 e 2973 (ν_s/as_ C_2_), 2953 and 2921 (ν_as/s_ CH_3_), 1653 (ν C=O), 1622 (ν C=N), 1601 (*δ* NH), 1521 (ν C=C_ar_), 1442 (*δ*_s_ CH_2_), 1375 (*δ*_s_ CH_3_), 1274 (ν C-N), 1201 (ν C-O), 1167 (*δ* OH + =C-O), 974 (*δ* HC=CH), 888 (*δ* =CH), 827 (*δ* CH_ar_). ^1^H NMR (300 MHz, DMSO-*d*_6_), *δ* (ppm): 10.4 (s, 1H, H10), 9.96 (s, 1H, H11), 7.31–7.58 (m, 3H, H1, H5; H7), 6.79 (t, *J* = 9.95 Hz, 3H, H2/H4; H8), 6.15 (s, 1H, H14), 4.80 (t, *J* = 8.05 Hz, 2H, H19), 2.93 (t, *J* = 14.3 Hz, 1H, H15), 2.28–2.36 (m, 1H, H16), 1.98–2.22 (m, 3H, H17; H15), 1.85 (s,1H, H21), 1.75 (s, 1H, H20); ^13^C NMR (75 MHz, DMSO-*d*_6_), *δ* (ppm): 167.6 (C9), 159.6 (C3), 153.5 (C12), 133.8 (C14), 133.0 (C13), 130.0 (C1, C5), 126.4 (C6), 117.8 (C8), 116.3 (C2, C4), 110.8 (C19), 40.6 (C16), 30.1 (C17), 29.6 (C15), 20.9 (C20), 18.4 (C21). HRMS(ESI): *m*/*z* [M + H]^+^ calcd for C_19_H_22_N_2_O_2_ 310.1681 found [M + H]^+^ 311.17540.
(*E*)-3-(4-methoxyphenyl)-*N*’-((*R*,*E*)-2-methyl-5-(prop-1-en-2-yl)cyclohex-2-en-1-ylidene)acrylohydrazide **(**PQM-294, **11e)**
MW: 324.42 g/mol. Chemical Formula: C_20_H_24_N_2_O_2_. Physical appearance: pale yellow solid. Melting range: 230–232 °C. Purity (HPLC): 100%. ${\left[\alpha \right]}_{D}^{25}$ = +0.04. Yield: 85%. IR (ATR, *v*_max_, cm^−1^): 3161 (ν NH), 3073 (ν_as_ CH_2_), 3031 (ν_s_ CH_2_), 2968 and 2918 (ν_as/s_ CH_3_), 2835 (ν_s_ OCH_3_), 1657 (ν C=O), 1617 (ν C=N), 1595 (*δ* NH), 1509 (ν C=C_ar_), 1463 (*δ*_s_ CH_2_), 1374 (*δ*_s_ CH_3_), 1252 (ν_as_ C-O), 1166 (ν C-N), 984 (*δ* HC=CH), 892 (*δ* =CH), 817 (*δ* CH_ar_). ^1^H NMR (300 MHz, CDCl_3_), *δ* (ppm): 1.80 (s, 3H, H20), 1.97 (S, 3H, H21), 2.07–2.17 (m, 2H, H17; H15), 2.28–2.48 (m, 2H, H16; H15), 2.89 (dd, *J* = 16.0.Hz, *J* = 3.5 Hz, 1H, H17), 3.84 (s, 3H, H10), 4.84 (s, 2H), 6.15 (d, *J* = 5.6 Hz, 1H, H14), 6.92 (d, *J* = 8.6 Hz, 2H, H2, H4), 7.48 (d, *J* = 16.0 Hz, 1H, H8), 7.54 (d, *J* = 8.6 Hz, 2H, H1; H5), 7.77 (d, *J* = 16.0 Hz, 1H, H7), 9.49 (s, 1H, H11). ^13^C NMR (75 MHz, CDCl_3_), *δ* (ppm): 168.2 (C9), 161.1 (C3), 149.6 (C18), 147.5 (C7), 142.9 (C13), 132.8 (C14), 129.8 (C1, C5), 128.1 (C6), 114.4 (C8), 114.2 (C2, C4), 110.3 (C19), 55.6 (C10), 40.6 (C16), 30.1 (C17), 28.8 (C15), 20.8 (C20), 17.9 (C21). HRMS(ESI): *m*/*z* [M + H]^+^ calcd for C_20_H_24_N_2_O_2_ 324.1838 found [M + H]^+^ 325.19118.
(*E*)-3-(4-methoxyphenyl)-*N*’-((*R*,*E*)-2-methyl-5-(prop-1-en-2-yl)cyclohex-2-en-1-ylidene)acrylohydrazide **(**PQM-295, **10e)**
MW: 324.42 g/mol. Chemical Formula: C_20_H_24_N_2_O_2_. Physical appearance: pale yellow solid. Melting range: 215–216 °C. Purity (HPLC): 100%. ${\left[\alpha \right]}_{D}^{25}$ = −0.10. Yield: 79%. IR (ATR, *v*_max_, cm^−1^): 3162 (ν NH), 3073 (ν_as_ CH_2_), 3032 (ν_s_ CH_2_), 2968 and 2918 (ν_as/s_ CH_3_), 2835 (ν_s_ OCH_3_), 1658 (ν C=O), 1617 (ν C=N), 1595 (*δ* NH), 1510 (ν C=C_ar_), 1463 (*δ*_s_ CH_2_), 1375 (*δ*_s_ CH_3_), 1252 (ν_as_ C-O), 1166 (ν C-N), 984 (*δ* HC=CH), 893 (*δ* =CH), 817 (*δ* CH_ar_). ^1^H NMR (300 MHz, CDCl_3_), *δ* (ppm): 9.50 (s, 1H, H11), 7.77 (d, *J* = 16.0 Hz, 1H, H7), 7.54 (d, *J* = 8.6 Hz, 2H, H1; H5), 7.46 (d, *J* = 16.0 Hz, 1H, H8), 6.92 (d, *J* = 8.6 Hz, 2H, H2, H4), 6.15 (d, *J* = 4.3 Hz, 1H, H14), 4.84 (s, 2H, H19), 3.84 (s, 3H, H10), 2.89 (dd, *J* = 16.1 Hz, *J* = 2.9 Hz, 1H, H17), 2.30–2.48 (m, 2H, H16; H15), 2.12 (dd, *J* = 14.4 Hz, *J* = 13.1 Hz, 2H, H17; H15), 1.98 (s, 3H, H21), 1.80 (s, 3H, H20); ^13^C NMR (75 MHz, CDCl_3_), *δ* (ppm): 168.2 (C9), 161.1 (C3), 149.6 (C18), 147.5 (C7), 142.9 (C13), 132.8 (C14), 129.8 (C1, C5), 128.1 (C6), 114.4 (C8), 114.2 (C2, C4), 110.3 (C19), 55.4 (C10), 40.6 (C16), 30.1 (C17), 28.9 (C15), 20.8 (C20), 17.9 (C21). HRMS(ESI): *m*/*z* [M + H]^+^ calcd for C_20_H_24_N_2_O_2_ 324.1838 found [M + H]^+^ 325.19113.
(*E*)-*N*’-((*S*,*E*)-2-methyl-5-(prop-1-en-2-yl)cyclohex-2-en-1-ylidene)-3-(4-(trifluoromethyl)phenyl)acrylohydrazide **(**PQM-300, **11f)**
MW: 362.40 g/mol. Chemical Formula: C_20_H_21_F_3_N_2_O. Physical appearance: white solid. Melting range: 220–221 °C. Purity (HPLC): 100%. ${\left[\alpha \right]}_{D}^{25}$ = +0.07. Yield: 70%. IR (ATR, *v*_max_, cm^−1^): 3168 (ν NH), 1688 (ν C=O), 1624 (*δ* NH), 1575 (ν C=N), 1318 and 1124 (ν CF_3_), 979 (*δ* HC=CH), 895 (*δ* =CH), 831 (*δ* CH_ar_). ^1^H NMR (300 MHz, CDCl_3_), *δ* (ppm): 9.26 (s, 1H, H11), 7.81 (d, *J* = 16.1 Hz, 1H, H7), 7.62–7.70 (m, 5H, H1; H2; H4; H5; H8), 6.19 (d, *J* = 7.1 Hz, 1H, H14), 4.84 (d, *J* = 5.6 Hz 2H, H19), 2.83 (dd, *J* = 15.7 Hz, *J* = 4.0 Hz, 1H, H17), 2.30–2.48 (m, 2H, H16; H15), 2.07–2.18 (m, H17, H15), 1.97 (s, 3H, H21), 1.80 (s, 3H, H20); ^13^C NMR (75 MHz, CDCl_3_), *δ* (ppm): 167.1 (C9), 150.2 (C3), 147.3 (C18), 141.4 (C7), 138.7 (C6), 133.5 (C14), 132.7 (C13), 128.3 (C1; C2; C4; C5), 119.4 (C8), 110.5 (C19), 40.6 (C16), 30.1 (C17), 28.8 (C15), 20.8 (C20), 17.9 (C21). HRMS(ESI): *m*/*z* [M + H]^+^ calcd for C_20_H_21_F_3_N_2_O 362.1606 found [M + H]^+^ 363.16785.
(*E*)-*N*’-((*R*,*E*)-2-methyl-5-(prop-1-en-2-yl)cyclohex-2-en-1-ylidene)-3-(4-(trifluoromethyl)phenyl)acrylohydrazide **(**PQM-301, **10f)**
MW: 362.40 g/mol. Chemical Formula: C_20_H_21_F_3_N_2_O. Physical appearance: white solid. Melting range: 102–103 °C. Purity (HPLC): 100%. ${\left[\alpha \right]}_{D}^{25}$ = −0.04. Yield: 40%. IR (ATR, *v*_max_, cm^−1^): 3167 (ν NH), 2923 (ν_s_ CH_2_), 1666 (ν C=O), 1623 (*δ* NH), 1575 (ν C=N), 1318 (ν C-F_3_), 979 (*δ* HC=CH), 895 (*δ* =CH), 830 (*δ* CH_ar_). ^1^H NMR (300 MHz, CDCl_3_), *δ* (ppm): 9.36 (s, 1H, H11), 7.80 (d, *J* = 16.1 Hz, 1H, H7), 7.61–7.69 (m, 5H, H1; H2; H4; H5; H8), 6.18 (d, *J* = 7.1 Hz, 1H, H14), 4.83 (d, *J* = 4.8 Hz 2H, H19), 2.83 (dd, *J* = 15.9 Hz, *J* = 3.8 Hz, 1H, H17), 2.31–2.49 (m, 2H, H16; H15), 2.03–2.21 (m, H17, H15), 1.96 (s, 3H, H21), 1.79 (s, 3H, H20); ^13^C NMR (75 MHz, CDCl_3_), *δ* (ppm): 167.2 (C9), 150.3 (C3), 147.3 (C18), 141.4 (C7), 138.7 (C6), 133.6 (C14), 132.7 (C13), 128.3 (C1; C2; C4; C5), 119.4 (C8), 110.4 (C19), 40.6 (C16), 30.1 (C17), 28.8 (C15), 20.8 (C20), 17.9 (C21). HRMS(ESI): *m*/*z* [M + H]^+^ calcd for C_20_H_21_F_3_N_2_O 362.1606 found [M + H]^+^ 363.16781.
(*E*)-3-(2-hydroxyphenyl)-*N*’-((*R*,*E*)-2-methyl-5-(prop-1-en-2-yl)cyclohex-2-en-1-ylidene)acrylohydrazide **(**PQM-302, **11g)**
MW: 310.39 g/mol. Chemical Formula: C_19_H_22_N_2_O_2_. Physical appearance: yellow solid. Melting range: 215–217 °C. Purity (HPLC): 100%. ${\left[\alpha \right]}_{D}^{25}$ = +0.09. Yield: 69%. IR (ATR, *v*_max_, cm^−1^): 3208 (ν OH), 1644 (ν C=O), 1599 (*δ* NH), 1451 (*δ* CH_3_), 1200 (ν C-OH), 989 (*δ* HC=CH), 888 (*δ* =CH), 754 (*δ* CH_ar_). ^1^H NMR (300 MHz, DMSO-*d*_6_), *δ* (ppm): 10.13–10.46 (s, 1H, H11; H11′), 7.82 (t, *J* = 16.9 Hz, 1H, H7), 7.47–7.66 (m, 1H, H8), 7.20 (t, *J* = 7.8 Hz, 1H, H3), 6.82–7.01 (m, 3H, H2; H4; H5), 6.16 (s, 1H, H14), 4.78–4.83 (m, 2H, H19), 2.94 (t, *J* = 12.5 Hz, 1H, H17), 2.21–2.36 (m, 2H, H16; H15), 2.03–2.11 (m, 2H, H17, H15), 1.89–1.84 (s, 3H, H21), 1.76 (s, 3H, H20). ^13^C NMR (75 MHz, DMSO-*d*_6_), *δ* (ppm): 167.9 (C9), 157.2 (C5), 153.8 (C12), 149.7 (C7), 148.3 (C18), 138.7 (C6), 138.0 (C13), 136.2 (C3), 133.1 (C14), 133.9 (C1), 122.2 (C8), 121.7 (C6), 120.6 (C2), 117.7 (C6), 116.7 (C4), 110.8 (C19), 40.6 (C16), 30.2 (C17), 29.6 (C15), 21.0 (C20), 18.5 (C21). HRMS(ESI): *m*/*z* [M + H]^+^ calcd for C_19_H_22_N_2_O_2_ 310.1681 found [M + H]^+^ 311.17541.
(*E*)-3-(2-hydroxyphenyl)-*N*’-((*R*,*E*)-2-methyl-5-(prop-1-en-2-yl)cyclohex-2-en-1-ylidene)acrylohydrazide **(**PQM-303, **10g)**
MW: 310.39 g/mol. Chemical Formula: C_19_H_22_N_2_O_2_. Physical appearance: pale yellow solid. Melting range: 231–233 °C. Purity (HPLC): 100%. ${\left[\alpha \right]}_{D}^{25}$ = −0.13. Yield: 60%. IR (ATR, *v*_max_, cm^−1^): 3208 (ν OH), 1644 (ν C=O), 1651 (ν C=N), 1599 (*δ* NH), 1451 (*δ* CH_3_), 1200 (ν C-OH), 989 (*δ* HC=CH), 889 (*δ* =CH), 754 (*δ* CH_ar_). ^1^H NMR (300 MHz, DMSO-*d*_6_), *δ* (ppm): 10.41–10.52 (s, 1H, H10), 10.13 (s, 1H, H11), 7.81 (t, *J* = 17.1 Hz, 1H, H7), 7.56 (dd, *J* = 11.2 Hz and *J* = 44.2 Hz 1H, H8), 7.20 (t, *J* = 7.75.Hz, 1H, H3), 6.84–6.92 (m, 3H, H2; H4; H1), 6.16 (s, 1H, H14), 4.78–4.83 (m, 2H, H19), 2.94 (t, *J* = 13.6 Hz, 1H, H17), 2.29 (dd, *J* = 14.7 Hz e *J* = 26.2 Hz, 2H, H16; H15), 2.09 (dd, *J* = 15.5 Hz and *J* = 28.0 Hz, 2H, H17; H15), 1.88–1.84 (s, 3H, H21; H21′), 1.76 (s, 3H, H20); ^13^C NMR (75 MHz, DMSO-*d*_6_), *δ* (ppm): 167.4 (C9), 156.4 (C5), 153.3 (C12), 149.2 (C7), 147.8 (C18), 138.7 (C6), 137.5 (C13), 135.7 (C3), 133.4 (C14), 132.6 (C1), 130.8 (C8), 121.7 (C6), 119.4 (C2), 116.2 (C4), 110.3 (C19), 40.6 (C16), 29.7 (C15, C17), 20.5 (C20), 18.0 (C21). HRMS(ESI): *m*/*z* [M + H]^+^ calcd for C_19_H_22_N_2_O_2_ 310.1681 found [M + H]^+^ 311.17543.
(*E*)-3-(benzo[*d*][1,3]dioxol-5-yl)-*N*’-((*S*,*E*)-2-methyl-5-(prop-1-en-2-yl)cyclohex-2-en-1-ylidene)acrylohydrazide **(**PQM-304, **11h)**
MW: 338.41 g/mol. Chemical Formula: C_20_H_22_N_2_O_3_. Physical appearance: pale yellow solid. Melting range: 244–245 °C. Purity (HPLC): 100%. ${\left[\alpha \right]}_{D}^{25}$ = +0.11. Yield: 70%. IR (ATR, *v*_max_, cm^−1^): 3155 (ν NH), 2916 (ν_s_ CH_2_), 1669 (ν C=O), 1629 (C=N), 1609 (*δ* NH), 1485 (*δ* CH_2_), 1361 (*δ* CH_3_), 1239 (ν_ass_ C-O-C), 1035 (ν_s_ C-O), 973 (*δ* HC=CH). ^1^H NMR (300 MHz, CDCl_3_), *δ* (ppm): 9.31 (s, 1H, H11), 7.72 (d, *J* = 15.87 Hz, 1H, H7), 7.40 (d, *J* = 15.90 Hz, 1H, H8), 7.11 (s, 1H, H1), 7.06 (d, *J* = 8.80 Hz, 1H, H5), 6.83 (d, *J* = 7.98 Hz, 1H, H4), 6.15 (d, *J* = 5.98 Hz, 1H, H14), 6.01 (s, 2H, H10), 4.83 (s, 2H, H19), 2.85 (dd, *J* = 3.74 Hz e *J* = 15.89 Hz 1H, H17), 2.,37–2.48 (m, 1H, H16), 2.28–2.36 (m, 1H, H15), 2.10–2.18 (m, 2H, H17, H15), 1.97 (s, 3H, H21), 1.80 (s, 3H, H20); ^13^C NMR (75 MHz, CDCl_3_), *δ* (ppm): 167.9 (C9), 149.6 (C3), 149.3 (C12), 148.2 (C2), 147.4 (C18), 143.0 (C7), 133.0 (C13), 132.9 (C6), 129.8 (C5), 124.5 (C8), 114.8 (C1), 110.4 (C4), 108.5 (C19), 106.6 (C14), 101.5 (C10), 40.6 (C16), 30.1 (C17), 28.8 (C15), 20.8 (C20), 17.9 (C21). HRMS(ESI): *m*/*z* [M + H]^+^ calcd for C_20_H_22_N_2_O_3_ 338.1630 found [M + H]^+^ 339.17039.
(*E*)-3-(benzo[*d*][1,3]dioxol-5-yl)-*N*’-((*R*,*E*)-2-methyl-5-(prop-1-en-2-yl)cyclohex-2-en-1-ylidene)acrylohydrazide **(**PQM-305, **10h)**
MW: 338.41 g/mol. Chemical Formula: C_20_H_22_N_2_O_3_. Physical appearance: pale yellow solid. Melting range: 237–239 °C. Purity: 100%. ${\left[\alpha \right]}_{D}^{25}$ = −0.12. Yield: 17%. IR (ATR, *v*_max_, cm^−1^): 3355 (ν NH), 1653 (ν C=O), 1610 (C=N), 1595 (*δ* NH), 1489 (*δ* CH_2_), 1374 (*δ* CH_3_), 1254 (ν_ass_ C-O-C), 1102 (ν_s_ C-O), 985 (*δ* HC=CH). ^1^H NMR (300 MHz, CDCl_3_), *δ* (ppm): 9.08 (s, 1H, H11), 7.73 (d, *J* = 15.9 Hz, 1H, H7), 7.39 (d, *J* = 15.95 Hz, 1H, H8), 7.12 (s, 1H, H1), 7.06 (dd, *J* = 8.11 Hz, *J* = 1.03 Hz, 1H, H5), 6.83 (d, *J* = 7.98 Hz, 1H, H4), 6.16 (d, *J* = 5.68 Hz, 1H, H14), 6.02 (s, 2H, H10), 4.84 (d, *J* = 5.62 Hz, 2H, H19), 2.80 (dd, *J* = 3.84 Hz e *J* = 15.28 Hz 1H, H17), 2.29–2.46 (m, 2H, H16 e H15), 2.12 (dd, *J* = 6.24 Hz e *J* = 8.34 Hz, *J* = 15.54 Hz 2H, H17, H15), 1.97 (s, 3H, H21), 1.79 (s, 3H, H20), ^13^C NMR (75 MHz, CDCl_3_), *δ* (ppm): 167.8 (C9), 149.5 (C3), 149.3 (C12), 148.2 (C2), 147.4 (C18), 143.1 (C7), 133.0 (C13), 132.8 (C6), 129.8 (C5), 124.5 (C8), 114.7 (C1), 110.4 (C4), 108.6 (C19), 106.6 (C14), 101.5 (C10), 40.6 (C16), 30.1 (C17), 28.7 (C15), 20.8 (C20), 17.9 (C21). HRMS(ESI): *m*/*z* [M + H]^+^ calcd for C_20_H_22_N_2_O_3_ 338.1630 found [M + H]^+^ 339.17044.
(*E*)-3-(4-chlorophenyl)-*N*’-((*S*,*E*)-2-methyl-5-(prop-1-en-2-yl)cyclohex-2-en-1-ylidene)acrylohydrazide **(**PQM-306, **11i)**
MW: 328.84 g/mol. Chemical Formula: C_19_H_21_ClN_2_O. Physical appearance: white solid. Melting range: 215–216 °C. Purity: 100%. Yield: 63%. IR (ATR, *v*_max_, cm^−1^): 3167 (ν NH), 2919 (ν_s_ CH_2_), 1663 (ν C=O), 1617 (C=N), 1489 (*δ* CH_2_), 1361 (*δ* CH_3_), 1088 (ν_s_ C-Cl), 976 (*δ* HC=CH), 815 (*δ* CH_ar_). ^1^H NMR (300 MHz, CDCl_3_), *δ* (ppm): 8.97 (s, 1H, H10), 7.76 (d, *J* = 16.37 Hz, 1H, H7), 7.50–7.56 (m, 3H, H2, H4, H8), 7.37 (d, *J* = 8.43, 2H, H1, H5), 6.18 (s, 1H, H13), 4.83 (d, *J* = 10.57 Hz, 2H, H18), 2.78 (dd, *J* = 3.94 Hz e *J* = 15.79 Hz 1H, H16), 2.31–2.46 (m, 2H, H14, H15), 2.11 (dd, *J* = 13.93 Hz e *J* = 25.80 Hz, 2H, H16, H14), 1.97 (s, 3H, H20), 1.79 (s, 3H, H19); ^13^C NMR (75 MHz, CDCl_3_), *δ* (ppm): 167.3 (C9), 149.7 (C11), 147.3 (C17), 141.9 (C7), 135.8 (C12), 133.8 (C13), 133.3 (C6), 132.7 (C3), 129.3 (C2 e C4), 129.1 (C1 e C5), 117.3 (C8), 110.5 (C18), 40.6 (C15), 30.1 (C16), 28.6 (C14), 20.8 (C19), 18.0 (C20). HRMS(ESI): *m*/*z* [M + H]^+^ calcd for C_19_H_21_ClN_2_O 328.1342 found [M + H]^+^ 329.14164.
(*E*)-3-(4-chlorophenyl)-*N*’-((*R*,*E*)-2-methyl-5-(prop-1-en-2-yl)cyclohex-2-en-1-ylidene)acrylohydrazide **(**PQM-307, **10i)**
MW: 328.84 g/mol. Chemical Formula: C_19_H_21_ClN_2_O. Physical appearance: pale yellow solid. Melting range: 234–236 °C. Purity: 100%. ${\left[\alpha \right]}_{D}^{25}$ = −0.68. Yield: 71%. IR (ATR, *v*_max_, cm^−1^): 3166 (ν NH), 2916 (ν_s_ CH_2_), 1663 (ν C=O), 1617 (C=N), 1489 (*δ* CH_2_), 1360 (*δ* CH_3_), 1087 (ν_s_ C-Cl), 976 (*δ* HC=CH), 815 (*δ* CH_ar_). ^1^H NMR (300 MHz, CDCl_3_), *δ* (ppm): 8.92 (s, 1H, H10), 7.76 (d, *J* = 16.05 Hz, 1H, H7), 7.51–7.55 (m, 3H, H2, H4, H8), 7.37 (d, *J* = 8.49 Hz, 2H, H1, H5), 6.18 (d, *J* = 4.84 Hz, 1H, H13), 4.82 (d, *J* = 11.28 Hz, 2H, H18), 2.76 (dd, *J* = 3.86 Hz e *J* = 15.90 Hz 1H, H16), 2.29–2.49 (m, 2H, H14, H15), 2.13 (m, *J* = 7.47 Hz, *J* = 10.063 Hz e *J* = 28.31 Hz, 2H, H16, H14), 1.97 (s, 3H, H20), 1.79 (s, 3H, H19), ^13^C NMR (75 MHz, CDCl_3_), *δ* (ppm): 167.2 (C9), 149.7 (C11), 147.3 (C17), 141.9 (C7), 135.8 (C12), 133.8 (C13), 133.2 (C6), 132.7 (C3), 129.3 (C2 e C4), 129.1 (C1 e C5), 117.3 (C8), 110.5 (C28), 40.6 (C15), 30.0 (C16), 28.6 (C14), 20.8 (C19), 17.9 (C20). HRMS(ESI): *m*/*z* [M + H]^+^ calcd for C_19_H_21_ClN_2_O 328.1342 found [M + H]^+^ 329.1418.
(*E*)-3-(3-hydroxy-4-methoxyphenyl)-*N*’-((*R*,*E*)-2-methyl-5-(prop-1-en-2-yl)cyclohex-2-en-1-ylidene)acrylohydrazide **(**PQM-308, **11j)**
MW: 340.42 g/mol. Chemical Formula: C_20_H_24_N_2_O_3_. Physical appearance: yellow solid. Melting range: 190–192 °C. Purity: 100%. ${\left[\alpha \right]}_{D}^{25}$ = +0.16. Yield: 30%. IR (ATR, *v*_max_, cm^−1^): 3362 (ν OH), 2911 (ν_s_ CH_2_), 2835 (ν_s_ CH_3_), 1647 (C=O), 1598 (*δ* NH), 1506 (*δ* C=C_ar_), 1274 (ν C-O-C), 974 (*δ* CH), 795 (*δ* CH_ar_). ^1^H NMR (300 MHz, CDCl_3_), *δ* (ppm): 9.21 (s, 1H, H12), 7.72 (d, *J* = 16.0 Hz, 1H, H7), 7.42 (d, *J* = 16.0 Hz, 1H, H8), 7.23 (s, 1H, H5), 7.08 (d, *J* = 8.8 Hz, 1H, H1), 6.86 (d, *J* = 8.2 Hz, 1H, H2), 6.15 (d, *J* = 4.9 Hz, 1H, H15), 5.85 (s, 1H, H10), 4.83 (d, *J* = 5.2 Hz, 2H, H20), 3.93 (s, 3H, H11), 2.82 (dd, *J* = 2.5 Hz, *J* =15.3 Hz, 1H, H18), 2.44 (t, *J* = 11.7 Hz 1H, H17), 2.32 (dd, *J* = 5.0 Hz, *J* = 16.9 Hz, 1H, H16), 2.11 (dd, *J* = 11.1 Hz, *J* = 26.3 Hz, 2H, H18, H16), 1.97 (s, 3H, H22), 1.79 (s, 3H, H21); ^13^C NMR (75 MHz, CDCl_3_), *δ* (ppm): 168.0 (C9), 149.5 (C3), 148.3 (C4), 147.4 (C19), 145.8 (C14), 143.2 (C7), 132.9 (C15), 129.0 (C6), 122.2 (C1), 114.8 (C8), 113.0 (C5), 110.4 (C2), 110.5 (C20), 56.0 (C11), 40.6 (C17), 30.1 (C18), 28.7 (C16), 20.8 (C21), 17.9 (C22). HRMS(ESI): *m*/*z* [M + H]^+^ calcd for C_20_H_24_N_2_O_3_ 340.1787 found [M + H]^+^ 341.18600.
(E)-3-(3-hydroxy-4-methoxyphenyl)-*N*’-((*R*,E)-2-methyl-5-(prop-1-en-2-yl)cyclohex-2-en-1-ylidene)acrylohydrazide **(**PQM-309, **10j)**
MW: 340.42 g/mol. Chemical Formula: C_20_H_24_N_2_O_3_. Physical appearance: yellow solid. Melting range: 194–195 °C. Purity: 100%. ${\left[\alpha \right]}_{D}^{25}$ = −0.06. Yield: 24%. IR (ATR, *v*_max_, cm^−1^): 3243 (ν OH), 2911 (ν_s_ CH_2_), 2836 (ν_s_ CH_3_), 1647 (C=O), 1598 (*δ* NH), 1557 (ν C=N), 1506 (*δ* C=C_ar_), 1274 (ν C-O-C), 975 (*δ* CH), 795 (*δ* CH_ar_). ^1^H NMR (300 MHz, CDCl_3_), *δ* (ppm): 9.03 (s, 1H, H12), 7.73 (d, *J* = 15.8 Hz, 1H, H7), 7.41 (d, *J* = 16.0 Hz, 1H, H8), 7.24 (d, *J* = 1.6 Hz, 1H, H5), 7.08 (dd, *J* = 8.8 Hz, *J* = 1.7 Hz, 1H, H1), 6.86 (d, *J* = 8.3 Hz, 1H, H2), 6.15 (d, *J* = 5.6 Hz, 1H, H15), 5.78 (s, 1H, H10), 4.82 (d, *J* = 8.6 Hz, 2H, H20), 3.93 (s, 3H, H11), 2.79 (dd, *J* = 3.6 Hz, *J* = 16.1 Hz, 1H, H18), 2.28–2.48 (m, 2H, H17 e H16), 2.12 (dd, *J* = 8.1 Hz, *J* = 22.0 Hz, 2H, H18, H16), 1.97 (s, 3H, H22), 1.79 (s, 3H, H21); ^13^C NMR (75 MHz, CDCl_3_), *δ* (ppm): 167.9 (C9), 149.4 (C3), 148.3 (C4), 147.4 (C19), 145.7 (C14), 143.3 (C7), 132.9 (C15), 129.0 (C6), 122.2 (C1), 114.8 (C8), 112.9 (C5), 110.4 (C2), 110.5 (C20), 56.0 (C11), 40.6 (C17), 30.1 (C18), 28.6 (C16), 20.8 (C21), 17.9 (C22). HRMS(ESI): *m*/*z* [M + H]^+^ calcd for C_20_H_24_N_2_O_3_ 340.1787 found [M + H]^+^ 341.18580.
(*E*)-3-(4-hydroxy-3,5-dimethoxyphenyl)-*N*’-((*S*,*E*)-2-methyl-5-(prop-1-en-2-yl)cyclohex-2-en-1-ylidene)acrylohydrazide **(**PQM-375, **10k)**
MW: 370.45 g/mol. Chemical Formula: C_21_H_26_N_2_O_4_. Physical appearance: pale yellow solid. Melting range: 217–219 °C. Purity: 100%. ${\left[\alpha \right]}_{D}^{25}$ = −0.10. Yield: 51%. IR (ATR, *v*_max_, cm^−1^): 1652 (C=O), 1609 (*δ* NH), 1510 (ν C=N), 1456 (*δ* C=C_ar_), 1322 (ν C-O-C), 981 (*δ* CH), 605 (*δ* CH_ar_). ^1^H NMR (300 MHz, DMSO-*d*_6_), *δ* (ppm): 10.34 (s, 1H, H13), 7.37–7.57 (m, 1H, H7), 6.69 (s, 2H, H1 e H5), 6.80 (d, *J* = 15.4 Hz, 1H, H8), 6.15 (s, 1H, H16), 4.82 (d, *J* = 10.2 Hz, 2H, H21), 3.80 (s, 6H, H10 e H12), 2.91 (d, *J* = 14.5 Hz, 1H, H19), 2.30 (dd, *J* = 14.9 Hz, *J* = 32.1 Hz, 2H, H18 e H17), 2.10 (dd, *J* = 13.2 Hz, *J* = 26.3 Hz, 2H, H19, H17), 1.84 (s, 3H, H22), 1.76 (s, 3H, H23); ^13^C NMR (75 MHz, DMSO-*d*_6_), *δ* (ppm): 167.1 e 162.1 (C9), 153.1 (C14), 148.1 (C2, C4), 147.8 (C20), 140.9 (C7), 137.6 (C3), 133.4 (C15), 132.6 (C16), 125.3 (C8), 117.8 (C6), 110.4 (C21), 105.5 (C1, C5), 56.0 (C10, C12), 40.4 (C18), 29.6 (C19), 29.1 (C17), 20.5 (C22), 18.0 (C23). HRMS(ESI): *m*/*z* [M + H]^+^ calcd for C_21_H_26_N_2_O_4_ 370.1893 found [M + H]^+^ 371.19604.
(E)-3-(4-hydroxy-3,5-dimethoxyphenyl)-*N*’-((*S*,E)-2-methyl-5-(prop-1-en-2-yl)cyclohex-2-en-1-ylidene)acrylohydrazide **(**PQM-376, **11k)**
MW: 370.45 g/mol. Chemical Formula: C_21_H_26_N_2_O_4_. Physical appearance: pale yellow solid. Melting range: 196–197 °C. Purity: 95%. ${\left[\alpha \right]}_{D}^{25}$ = +0.10. Yield: 53%. IR (ATR, *v*_max_, cm^−1^): 1652 (C=O), 1612 (*δ* NH), 1512 (ν C=N), 1456 (*δ* C=C_ar_), 1323 (ν C-O-C), 980 (*δ* CH), 604 (*δ* CH_ar_). ^1^H NMR (300 MHz, C*D*Cl_3_), *δ* (ppm): 8.85 (s, 1H, H13), 7.73 (d, *J* = 15.8 Hz, 1H, H7), 7.41 (d, *J* = 15.7 Hz, 1H, H8), 6.84 (s, 2H, H1 e H5), 6.16 (d, *J* = 7.1 Hz 1H, H16), 5.81 (s, 1H, H11), 4.82 (d, *J* = 10.9 Hz, 2H, H21), 3.93 (s, 6H, H10 e H12), *2.*76 (dd, *J* = 4.1 Hz, *J* = 15.4 Hz 1H, H19), 2.44 (dd, *J* = 8.4 Hz, *J* = 19.8 Hz, 1H, H18), 2.28–2.38 (m,1H, H17), 2.04–2.18 (m, 2H, H19, H17), 1.96 (s, 3H, H22), 1.79 (s, 3H, H23); ^13^C NMR (75 MHz, C*D*Cl_3_), *δ* (ppm): 167.7 (C9), 149.3 (C12), 147.3 (C20), 147.2 (C2, C4), 143.7 (C7), 136.9 (C3), 133.1 (C16), 132.7 (C15), 126.9 (C6), 114.7 (C8), 110.5 (C21), 105.2 (C1, C5), 56.3 (C10, C12), 40.6 (C18), 30.1 (C19), 28.6 (C17), 20.8 (C22), 17.7 (C23). HRMS(ESI): *m*/*z* [M + H]^+^ calcd for C_21_H_26_N_2_O_4_ 370.1893 found [M + H]^+^ 371.19626.
(*E*)-3-(4-(dimethylamino)phenyl)-*N*’-((*R*,*E*)-2-methyl-5-(prop-1-en-2-yl)cyclohex-2-en-1-ylidene)acrylohydrazide **(**PQM-377, **10l)**
MW: 337.46 g/mol. Chemical Formula: C_21_H_27_N_3_O. Physical appearance: pale yellow solid. Melting range: 236–237 °C. Purity: 72%. ${\left[\alpha \right]}_{D}^{25}$ = −0.06. Yield: 53%. IR (ATR, *v*_max_, cm^−1^): 3155 e 2914 (*δ* NH), 1651 (C=O), 1591 (*δ* NH), 1553 (ν C=N), 1350 (*δ* C=C_ar_), 1227 (ν C-O-C), 937 (*δ* CH), 645 (*δ* CH_ar_). ^1^H NMR (300 MHz, C*D*Cl_3_), *δ* (ppm): 9.01 (s, 1H, H12), 7.76 (d, *J* = 15.8 Hz, 1H, H7), 7.49 (d, *J* = 8.6 Hz, 2H, H1 e H5), 7.35 (d, *J* = 15.9 Hz, 1H, H8), 6.69 (d, *J* = 8.6 Hz, 2H, H2 e H4), 6.13 (d, *J* = 5.7 Hz 1H, H15), 4.82 (d, *J* = 6.4 Hz, 2H, H20), 3.0 (s, 6H, H10 e H11), 2.81 (dd, *J* = 3.9 Hz, *J* = 15.6 Hz 1H, H18), 2.43 (dd, *J* = 7.8 Hz, *J* = 19.8 Hz, 1H, H17), 2.31 (dt, *J* = 4.9 Hz, *J* = 10.3 Hz,1H, H16), 2.03–2.17 (m, 2H, H18, H16), 1.97 (s, 3H, H21), 1.79 (s, 3H, H22), ^13^C NMR (75 MHz, C*D*Cl_3_), *δ* (ppm): 168.5 (C9), 151.6 (C3), 148.8 (C13), 147.5 (C19), 143.9 (C7), 133.0 (C14), 132.5 (C15), 129.8 (C1 e C5), 123.3 (C6), 111.9 (C2 e C4), 111.3 (C8), 110.3 (C20), 40.7 (C17), 40.2 (C10 e C11), 30.1 (C18), 28.7 (C16), 20.8 (C22), 17.9 (C21). HRMS(ESI): *m*/*z* [M + H]^+^ calcd for C_21_H_27_N_3_O 337.2154 found [M + H]^+^ 338.22232.
(*E*)-3-(4-(dimethylamino)phenyl)-*N*’-((*S*,*E*)-2-methyl-5-(prop-1-en-2-yl)cyclohex-2-en-1-ylidene)acrylohydrazide **(**PQM-378, **11l)**
MW: 337.46 g/mol. Chemical Formula: C_21_H_27_N_3_O. Physical appearance: pale yellow solid. Melting range: 220–223 °C. Purity: 75%. ${\left[\alpha \right]}_{D}^{25}$ = +0.03. Yield: 22%. IR (ATR, *v*_max_, cm^−1^): 3154 e 2914 (*δ* NH), 1651 (C=O), 1590 (*δ* NH), 1552 (ν C=N), 1360 (*δ* C=C_ar_), 1227 (ν C-O-C), 987 (*δ* CH), 645 (*δ* CH_ar_). ^1^H NMR (300 MHz, C*D*Cl_3_), *δ* (ppm): 9.07 (s, 1H, H12), 7.76 (d, *J* = 15.9 Hz, 1H, H7), 7.49 (d, *J* = 8.6 Hz, 2H, H1 e H5), 7.35 (d, *J* = 15.9 Hz, 1H, H8), 6.69 (d, *J* = 8.6 Hz, 2H, H2 e H4), 6.13 (d, *J* = 6.1 Hz 1H, H15), 4.82 (d, *J* = 5.3 Hz, 2H, H20), 3.01 (s, 6H, H10 e H11), 2.82 (dd, *J* = 4.0 Hz, *J* = 15.7 Hz 1H, H18), 2.44 (dt, *J* = 4.4 Hz, *J* = 12.5 Hz, 1H, H17), 2.31 (dt, *J* = 5.50 Hz, *J* = 11.4 Hz, 1H, H16), 2.04–2.17 (m, 2H, H18, H16), 1.97 (s, 3H, H21), 1.79 (s, 3H, H22); ^13^C NMR (75 MHz, C*D*Cl_3_), *δ* (ppm): 168.5 (C9), 151.6 (C3), 148.8 (C13), 147.5 (C19), 143.9 (C7), 133.0 (C14), 132.5 (C15), 129.8 (C1 e C5), 123.3 (C6), 111.9 (C2 e C4), 111.3 (C8), 110.3 (C20), 40.7 (C17), 40.2 (C10 e C11), 30.1 (C18), 28.7 (C16), 20.8 (C22), 17.9 (C21). HRMS(ESI): *m*/*z* [M + H]^+^ calcd for C_21_H_27_N_3_O 337.2154 found [M + H]^+^ 338.22235.
*N*’-((*R*,*E*)-2-methyl-5-(prop-1-en-2-yl)cyclohex-2-en-1-ylidene)cinnamohydrazide **(**PQM-379, **10m)**
MW: 294.39 g/mol. Chemical Formula: C_19_H_22_N_2_O. Physical appearance: pale yellow solid. Melting range: 210–211 °C. Purity: 100%. ${\left[\alpha \right]}_{D}^{25}$ = −0.14. Yield: 41%. IR (ATR, *v*_max_, cm^−1^): 3168, 3025 e 2916 (*δ* NH), 1660 (C=O), 1447 (ν C=N), 1358 (*δ* C=C_ar_), 1218 (ν C-O-C), 886 (*δ* CH), 760 (*δ* CH_ar_). ^1^H NMR (300 MHz, C*D*Cl_3_), *δ* (ppm): 9.32 (s, 1H, H10), 7.81 (d, *J* = 16.0 Hz, 1H, H7), 7.55–7.60 (m, 3H, H1, H5, H3), 7.40 (d, *J* = 6.2 Hz, 3H, H2, H4, H8), 6.16 (d, *J* = 5.5 Hz 1H, H13), 4.83 (s, 2H, H19), 2.86 (dd, *J* = 4.0 Hz, *J* = 15.8 Hz 1H, H16), 2.40–2.50 (m, 1H, H15), 2.28–2.37 (m, 1H, H14), 2.06–2.18 (m, 2H, H16, H14), 1.97 (s, 3H, H18), 1.80 (s, 3H, H20); ^13^C NMR (75 MHz, C*D*Cl_3_), *δ* (ppm): 167.8 (C9), 149.7 (C11), 147.4 (C17), 143.3 (C7), 135.4 (C6), 133.1 (C13), 132.8 (C12), 129.9 (C3), 128.8 (C2, C4), 128.2 (C1 e C5), 116.9 (C8), 110.4 (C19), 40.6 (C15), 30.1 (C16), 28.8 (C14), 20.8 (C20), 17.9 (C18). HRMS(ESI): *m*/*z* [M + H]^+^ calcd for C_19_H_22_N_2_O 294.1732 found [M + H]^+^ 295.18033.
*N*’-((*S*,*E*)-2-methyl-5-(prop-1-en-2-yl)cyclohex-2-en-1-ylidene)cinnamohydrazide **(**PQM380, **11m)**
MW: 294.39 g/mol. Chemical Formula: C_19_H_22_N_2_O. Physical appearance: pale yellow solid. Melting range: 185–186 °C. Purity: 100%. ${\left[\alpha \right]}_{D}^{25}$ = +0.12. Yield: 34%. IR (ATR, *v*_max_, cm^−1^): 3171, 3057 e 2916 (*δ* NH), 1663 (C=O), 1447 (ν C=N), 1359 (*δ* C=C_ar_), 1220 (ν C-O-C), 886 (*δ* CH), 760 (*δ* CH_ar_). ^1^H NMR (300 MHz, C*D*Cl_3_), *δ* (ppm): 9.28 (s, 1H, H10), 7.81 (d, *J* = 16.0 Hz, 1H, H7), 7.55–7.60 (m, 3H, H1, H5, H3), 7.40 (d, *J* = 6.1 Hz, 3H, H2, H4, H8), 6.16 (d, *J* = 5.8 Hz 1H, H13), 4.84 (d, *J* = 3.7 Hz, 2H, H19), 2.85 (dd, *J* = 3.9 Hz, *J* = 15.8 Hz 1H, H16), 2.40–2.51 (m, 1H, H14); ^13^C NMR (75 MHz, C*D*Cl_3_), *δ* (ppm): 167.7 (C9), 149.7 (C11), 147.4 (C17), 143.3 (C7), 35.4 (C6), 133.1 (C13, C12), 129.9 (C3), 128.8 (C2, C4), 128.2 (C1 e C5), 116.9 (C8), 110.4 (C19), 40.6 (C15), 30.1 (C16), 28.8 (C14), 20.8 (C20), 17.9 (C18). HRMS(ESI): *m*/*z* [M + H]^+^ calcd for C_19_H_22_N_2_O 294.1732 found [M + H]^+^ 295.18027.

### 4.2. Molecular Docking

For this study, we evaluated the cannabinoid receptors CB1 and CB2, and TRPV1, which may be involved in the anti-inflammatory and antinociceptive activities in vivo (Table 3). The cannabinoid receptors were aligned with the super command from PyMOL™ Molecular Graphics System (version 2.5.0, Schrödinger, LLC, New York, NY, USA), using the CB1 (PDB ID 8GHV) structure as reference. Protein preparation was carried out with the Protein Preparation Wizard of Maestro 13.9.135 (Schrödinger, LLC, New York, NY, USA) with the protonation and tautomeric states predicted with PROPKA3 at neutral pH. The reference ligands and evaluated compounds (named PQM) were prepared with the LigPrep and Epik tools of Maestro to predict the protonation and tautomeric states at neutral pH [37]. Docking studies were performed with the DockThor-VS platform (freely available at www.dockthor.lncc.br) using the Standard configuration of the search algorithm and a grid size of 22 Å in each dimension [38,39]. The docking protocol adopted in this work was validated by redocking the co-crystallized compounds, with the top-energy pose of all protein-ligand complexes being successfully predicted with RMSD values lower than 2 Å.

### 4.3. Animals

Swiss Webster mice (25–30 g) were kindly donated by the Instituto Vital Brazil (Niterói, Rio de Janeiro, Brazil). Mice were maintained in the Animal Experimentation Department of the Institute of Biomedical Sciences in a room with a light–dark cycle of 12 h, 22 ± 2 °C, from 60% to 80% humidity, and with food and water ad libitum. Animals were used only once throughout the experiments. All protocols were conducted in accordance with the principles and guidelines adopted by the National Council for the Control of Animal Experimentation (CONCEA), approved by the Ethical Committee for Animal Research (protocols number96/23, approved on 18 October 2023). All experimental protocols were performed during the light phase. Animal numbers per group were kept at a minimum, and at the end of each experiment, mice were killed by a ketamine/xylazine overdose.

### 4.4. Drugs and Reagents

Acetylsalicylic acid (ASA) was purchased from Sigma-Aldrich (St. Louis, MO, USA). Ethanol and formalin were purchased from Merck Inc. (Rio de Janeiro, Brazil). Morphine sulfate was kindly provided by Cristália (São Paulo, Brazil). Drugs were dissolved in saline (NaCl 0.9%) prior to use. All drugs were diluted just before their use.

### 4.5. Administration of Compounds and Drugs

All compounds were dissolved in saline to prepare 100 µmol/mL stock solutions. Before their use, solutions were freshly prepared from each stock solution using saline. Doses of 10 µmol/kg (final volume of 0.1 mL per animal) were administered by oral gavage. Acetylsalicylic acid (ASA, 1.100 µmol/kg) and morphine (5 µmol/kg) were used as reference drugs. The dose of ASA and morphine was chosen based on previous results obtained by our group when their DE_50_ (i.e., the dose that caused a 50% reduction in the nociceptive or anti-inflammatory effect) was calculated. The control group was given the vehicle only.

### 4.6. In Vivo Toxicity Test

Mice received an oral administration of 100 µmol/kg of each compound. After 24 h, the animals were euthanized with ketamine (50 mg/kg)/xylazine (20 mg/kg). A sample of blood was collected in a heparinized tube. The femur was removed, the ends were cut, and the bone marrow from each femur was washed with 1 mL of saline with heparin and collected. Samples of blood and bone marrow were submitted to a complete blood hemogram and cell count, respectively, in an automatic cell counter (PocH-100iV Diff, Sysmex, Kobe, Japan).

### 4.7. Formalin-Induced Nociception

This assay was performed as described by Sakurada et al., and adapted by Matheus et al. [40,41]. This model is characterized by a response that occurred in two phases. The first phase (acute neurogenic pain) occurred during the first 5 min after the intraplantar injection of formalin, and the second phase (inflammatory pain) occurred during the period from 15 to 30 min post-injection. The animals (*n* = 7, per group) received 20 μL of formalin (2.5% *v*/*v*) into the dorsal surface of the left hind paw. The time that the animal spent licking the injected paw was immediately recorded. The mice were pretreated with oral doses of **10a-m and 11a**–**m**, morphine, ASA, or vehicle 60 min before the administration of formalin.

### 4.8. Thermal-Induced Nociception (Hot Plate Test)

Mice were tested according to the method described by Sahley and Berntson and adapted by Matheus et al. [41,42]. Mice (*n* = 8 per group) were placed on a hot plate (Insight Equipment, São Paulo, Brazil) set at 55 ± 1 °C. The reaction time (licking of paws or jumping) was recorded every 30 min post-oral administration of compounds, vehicle, morphine, or cannabidiol (CBD) until 180 min. The average reaction time (in seconds) obtained at 60 and 30 min before oral administration was considered to be baseline (normal reaction to the temperature). The area under the curve (AUC) graphs were calculated from the time course graphs. The following formula, which is based on the trapezoid rule, was used to calculate the AUC: AUC = 30 × IB ((min 30) + (min 60) + (min 180)/2), where IB is the increase from the baseline (in %).

### 4.9. Statistical Analysis

The number of animals per group was indicated in each experiment. The results are presented as mean ± SD calculated using Prism Software 10.1.2 (GraphPad Software, La Jolla, CA, USA). One-way or two-way analysis of variance (ANOVA) followed by Tukey’s post hoc test was used for unpaired data when more than two groups were compared to the same control. The post hoc tests were run only if F achieved the necessary level of statistical significance. When *p* < 0.05, group differences were considered significant.

## 5. Conclusions

Twenty-six CBD-based terpenyl-cinnamoyl-acyl-hydrazone analogues were successfully obtained, with overall yields of up to 64%. The antinociceptive effect of these compounds was investigated by the classical methods of the formalin and hot plate assay, which also assisted in the search for the mechanism of action.

In the formalin test, six compounds showed comparable results to morphine in the first phase (neurogenic phase), especially PQM-292 and PQM-293. In contrast, in the second phase (inflammatory phase), PQM-292 showed a better result than morphine for chronic and inflammatory pain, which suggests the possible anti-inflammatory activity of this compound. Six compounds in the hot plate assay exhibited better results than morphine, especially PQM-274. These findings led to the investigation of the possible mechanism involved in the observed activities. In the computational studies, all compounds showed a low affinity for the CB receptors, although this does not impact their antinociceptive activity. Regarding the TRPV1 channels, neither of the compounds interacted with key residues, suggesting that these CBD-based analogs can act through a different mechanism, such as TRPA1, or that they could be active after metabolization. Moreover, the position and nature of the substituents on the aromatic moiety of the structure of compounds seemed to have a significant impact in the observed results, suggesting that electronegative and small H-bond donor/acceptor substituents, such as hydroxy and methoxy groups, potentially favor acute and chronic antinociceptive activity, with possible anti-inflammatory properties.

## Figures and Tables

**Table 1:** Molecular docking results for binding affinity of the series **10a**–**m** and **11a**–**m** for CB1, CB2, and TRPV1 receptors, with their respective PDB code.

Compounds	Predicted Binding Affinity (kcal/mol)		
	CB1 (8GHV)	CB2 (8GUR)	TRPV1 (8GFA)
**PQM-273 (10a)**	−10.75	−10.71	−9.25
**PQM-274 (11a)**	−10.72	−10.77	−9.74
**PQM-275 (10b)**	−11.45	−11.04	−9.61
**PQM-276 (11b)**	−11.21	−11.12	−9.71
**PQM-290 (11c)**	−10.58	−10.37	−9.09
**PQM-291 (10c)**	−10.40	−10.41	−9.38
**PQM-292 (11d)**	−10.51	−10.37	−9.17
**PQM-293 (10d)**	−10.36	−10.40	−9.41
**PQM-294 (11e)**	−10.91	−10.88	−9.35
**PQM-295 (10e)**	−11.00	−10.89	−9.69
**PQM-300 (11f)**	−10.93	−11.02	−9.56
**PQM-301 (10f)**	−10.78	−10.88	−9.63
**PQM-302 (11g)**	−10.49	−10.35	−9.25
**PQM-303 (10g)**	−10.63	−10.33	−9.37
**PQM-304 (11h)**	−11.02	−10.82	−9.34
**PQM-305 (10h)**	−10.81	−10.83	−9.32
**PQM-306 (11i)**	−10.74	−10.62	−9.31
**PQM-307 (10i)**	−10.90	−10.58	−9.36
**PQM-308 (11j)**	−10.88	−10.75	−9.65
**PQM-309 (10j)**	−10.82	−10.73	−9.58
**PQM-375 (10k)**	−11.24	−11.12	−9.76
**PQM-376 (11k)**	−11.15	−11.10	−9.90
**PQM-377 (10l)**	−11.08	−10.77	−9.41
**PQM-378 (11l)**	−10.89	−10.82	−9.41
**PQM-379 (10m)**	−10.56	−10.36	−9.33
**PQM-380 (11m)**	−10.52	−10.32	−8.92
**CBD**	−10.72	−10.71	−8.98

**Table 2:** In silico ADME prediction data for all synthetic compounds and CBD (QikProp V.3.5, Schrödinger).

*Cpd.* *PQM*	*CNS*	*HBD*	*HBA*	*QP* *logPo/w*	*QP* *logS*	*QP* *logHERG*	*QPP* *Caco*	*Q* *logBB*	*QPPMDCK*	*QPLog* *KHSA*	*%* *HOA*	*RO5*	*RO3*
*273*	−2	2	3.5	4.563	−6.234	−6.026	755.097	−1.205	365.162	0.767	100	0	12
***274***	**−2**	**2**	**3.5**	**4.563**	**−6.234**	**−6.026**	**755.097**	**−1.205**	**365.162**	**0.767**	**100**	**0**	**12**
*275*	0	1	3.5	5.448	−6.923	−6.068	2309.278	−0.659	1222.432	1.009	100	1	12
*276*	0	1	3.5	5.448	−6.923	−6.068	2309.458	−0.659	1222.535	1.009	100	1	12
*290*	−2	3	3.5	3.656	−5.546	−6.04	249.314	−1.739	110.231	0.505	91.251	0	12
***291***	**−2**	**3**	**3.5**	**3.65**	**−5.514**	**−6.007**	**250.504**	**−1.729**	**110.8**	**0.503**	**91.251**	**0**	**12**
***292***	**−2**	**2**	**2.75**	**4.41**	**−5.979**	**−6.163**	**700.726**	**−1.157**	**336.826**	**0.734**	**100**	**0**	**12**
***293***	**−2**	**2**	**2.75**	**4.41**	**−5.979**	**−6.163**	**700.744**	**−1.157**	**336.836**	**0.734**	**100**	**0**	**12**
***294***	**0**	**1**	**2.75**	**5.33**	**−6.658**	**−6.172**	**2306.787**	**−0.582**	**1221.007**	**0.99**	**100**	**1**	**12**
***295***	**0**	**1**	**2.75**	**5.33**	**−6.658**	**−6.172**	**2306.514**	**−0.582**	**1220.851**	**0.99**	**100**	**1**	**12**
*300*	0	1	2	6.229	−7.886	−6.223	2307.579	−0.244	5393.733	1.249	100	1	12
*301*	0	1	2	6.227	−7.884	−6.225	2309.438	−0.245	5369.953	1.248	100	1	12
*302*	−2	2	2.75	4.462	−5.917	−6.168	853.249	−1.053	416.727	0.725	100	0	12
*303*	−2	2	2.75	4.456	−5.901	−6.155	854.156	−1.051	417.205	0.722	100	0	12
***304***	**0**	**1**	**3.5**	**4.754**	**−5.94**	**−5.775**	**2308.067**	**−0.484**	**1221.74**	**0.776**	**100**	**0**	**15**
***305***	**0**	**1**	**3.5**	**4.752**	**−5.934**	**−5.769**	**2306.555**	**−0.484**	**1220.874**	**0.775**	**100**	**0**	**15**
*306*	0	1	2	5.723	−7.16	−6.183	2309.901	−0.342	3014.466	1.095	100	1	12
***307***	**0**	**1**	**2**	**5.726**	**−7.168**	**−6.19**	**2308.571**	**−0.343**	**3012.579**	**1.096**	**100**	**1**	**12**
***308***	**−2**	**2**	**3.5**	**4.537**	**−6.305**	**−6.097**	**697.894**	**−1.259**	**335.355**	**0.765**	**100**	**0**	**12**
***309***	**−2**	**2**	**3.5**	**4.537**	**−6.305**	**−6.097**	**697.895**	**−1.259**	**335.356**	**0.765**	**100**	**0**	**12**
*375*	−2	2	4.25	4.733	−6.346	−5.812	936.171	−1.161	460.669	0.785	100	0	12
*376*	−2	2	4.25	4.734	−6.358	−5.819	929.932	−1.166	457.351	0.787	100	0	12
*377*	0	1	3	5.687	−7.105	−6.092	2466.783	−0.551	1312.796	1.142	100	1	12
***378***	**0**	**1**	**3**	**5.672**	**−7.122**	**−6.126**	**2473.389**	**−0.553**	**1316.596**	**1.135**	**100**	**1**	**12**
*379*	0	1	2	5.233	−6.223	−6.129	2638.193	−0.424	1411.668	0.958	100	1	12
*380*	0	1	2	5.229	−6.234	−6.146	2637.946	−0.426	1411.525	0.957	100	1	12
***CBD***	0	2	1.5	5.025	−5.519	−4.892	2695.653	−0.43	1444.93	0.883	100	1	1

CNS—Predicted central nervous system activity (−2, inactive to +2, active). HBD—Hydrogen bonding donor (0 to 6). HBA—Hydrogen bonding acceptors (2 to 20). QPlogP o/w—Predicted octanol/water partition coefficient (−2.0 to 6.5). QPlogS—Aqueous solubility (−6.5 to 0.5). QPlogHERG—Predicted IC_50_ value for blockage of HERG K^+^ channels (concern below −5). QPPCaco—Permeability on cell assay, model for intestinal absorption (<25 poor; >500 good). QPlogBB—Permeability in the blood–brain barrier (−3.0 to 1.2). QPPMDK—Predicted apparent MDCK cell permeability in nm/sec. MDCK cells are considered to be a good mimic for the blood-barrier (<25 poor; >500 good). QPLogKHSA—Prediction of binding to human serum albumin (−1.5 to 1.5). % HOA—Percentage of human absorption by oral route (<25%-low; >80%-high in Caco). RO5: rule of 5—Number of violations of Lipinski’s rule of 5; compounds that satisfy these rules are considered drug-like (Max.4). RO3: rule of 3—Number of violations of Jorgensen’s rule; compounds with fewer violations of these rules are more likely to be orally available (Max. 3).

**Table 3:** Selected structures of the cannabinoid receptors CB1 and CB2, the TRPV1 receptor, and the search space configuration used in the docking studies.

Receptor	PDB/Resolution	Grid Center	Cofactor/Water
CB1	8GHV/2.8 Å	X = 146.41, Y = 143.88, Z = 192.33	None
	5U09/2.6 Å	X = 144.21, Y = 148.71, Z = 191.95	1 water molecule
CB2	5ZTY/2.8 Å	X = 147.27, Y = 144.43, Z = 190.03	1 water molecule
	8GUR/2.8 Å	X = 148.56, Y = 144.29, Z = 190.45	None
TRPV1	8GFA/2.29 Å	X = 105.36, Y = 80.97, Z = 88.36	POV (cofactor), 1 water molecule

## Data Availability

The original contributions presented in this study are included in the article/Supplementary Materials. Further inquiries can be directed to the corresponding authors.

## Supplementary Materials

The following supporting information can be downloaded at: https://www.mdpi.com/article/10.3390/ph18050755/s1, Analytical and spectroscopic data.

## Abbreviations

The following abbreviations are used in this manuscript:

CDB	Cannabidiol
CB1	Cannabinoid receptor type 1
CB2	Cannabinoid receptor type 2
TRPA1	Transient receptor potential ankyrin 1
TRPV1	Transient receptor potential cation channel subfamily V member 1
ASA	Acetylsalicylic acid
