# Supplementary material for: Design, Synthesis, and Evaluation of Antinociceptive Properties of Novel CBD-Based Terpene-Cinnamoyl-Acyl-Hydrazone Analogues
Source: Pharmaceuticals (Basel). 2025 May 20;18(5):755. doi: 10.3390/ph18050755 (PMC12114903; doi:10.3390/ph18050755)
Supplement: Supplementary file 1 [file pharmaceuticals-18-00755-s001.zip › pharmaceuticals-3631544-supplementary.pdf]

Design, Synthesis, and Evaluation of Antinociceptive Properties of a Novel CBD-based Terpenyl-Cinnamoyl Analogues

Supplementary materials

S1. Absorption in the IR region and NMR Spectrums of Intermediates 2

S2. Absorption in the IR region, NMR Spectrums, Mass spectrum, and HPLC chromatogram of final compounds 22

S1. Absorption in the IR region and NMR Spectrums of Intermediates

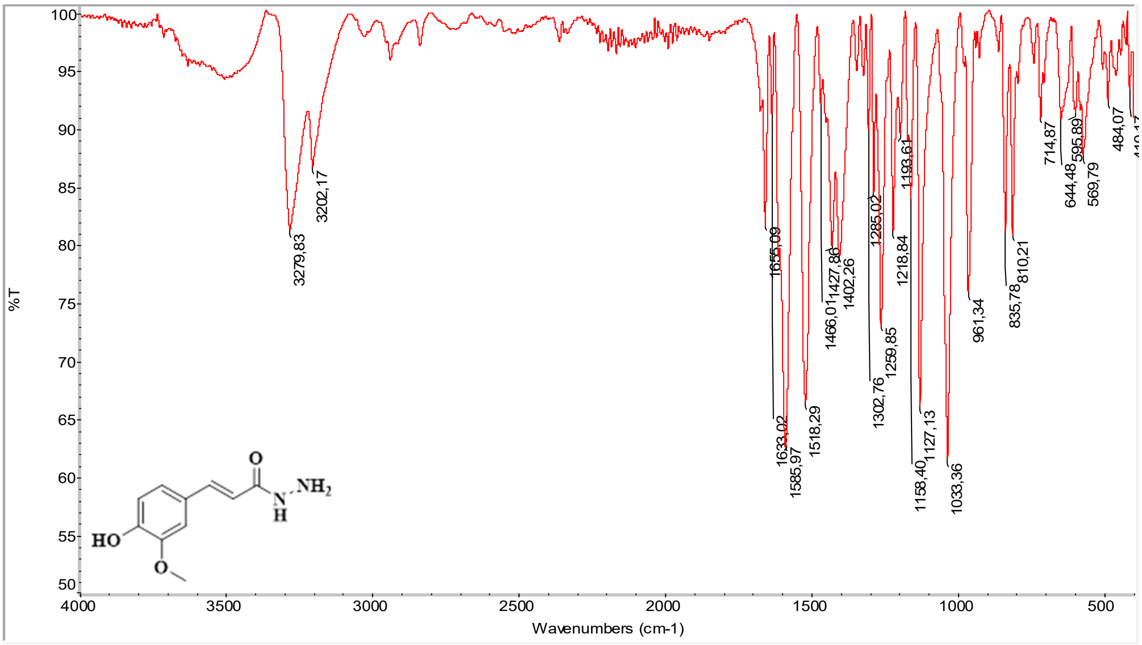

Figure S1. Absorption spectrum in the IR region (ATR) of intermediate **10a**.

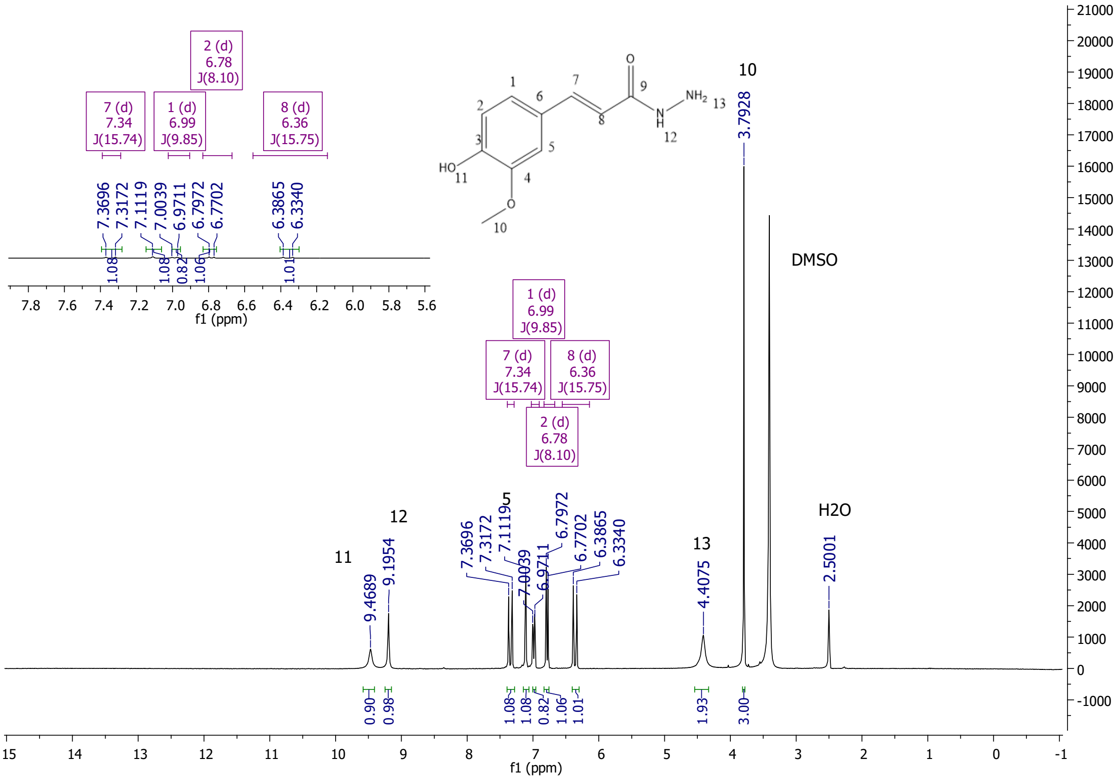

Figure S2. ^{1}H NMR spectrum (300 MHz, DMSO- d_6) of intermediate **10a**.

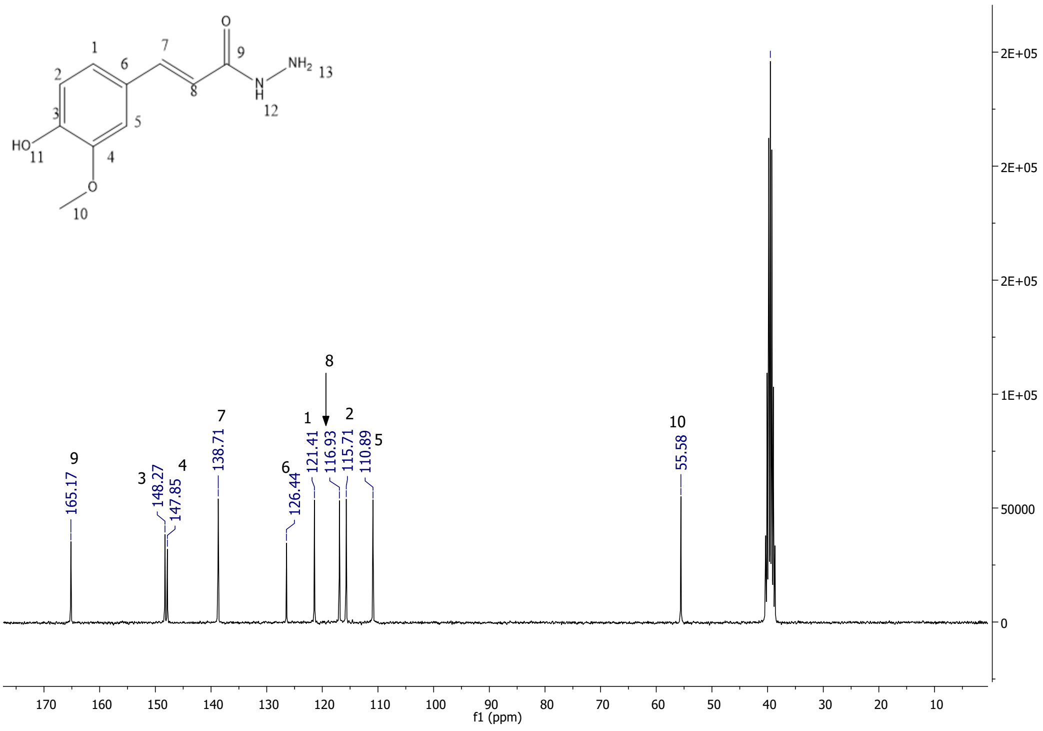

Figure S3. ^{13}C NMR spectrum (75 MHz, DMSO- d_6) of intermediate **10a**.

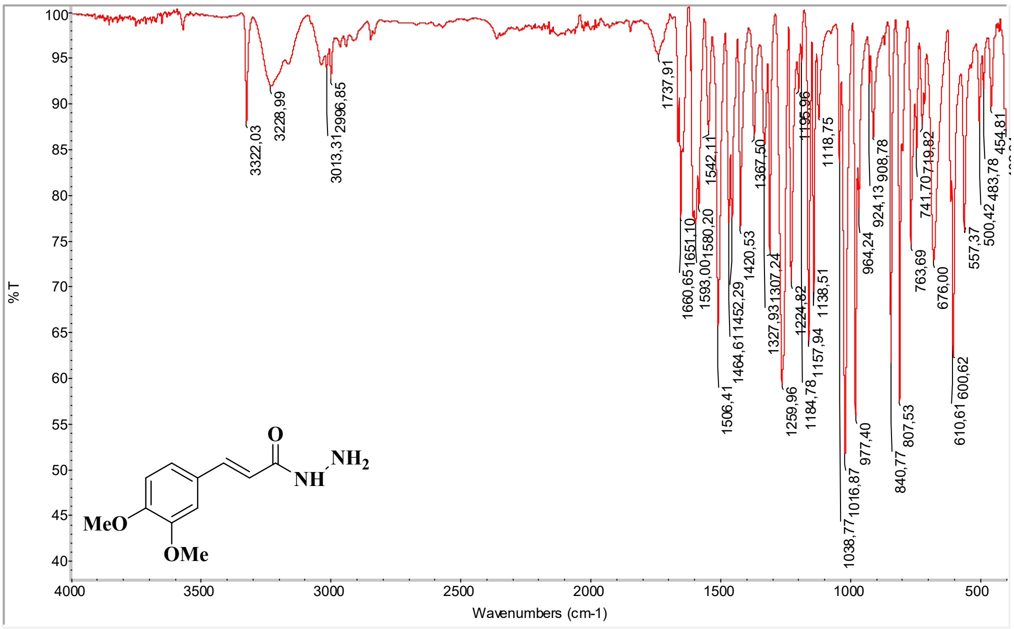

Figure S4. Absorption spectrum in the IR region (ATR) of intermediate **10b**.

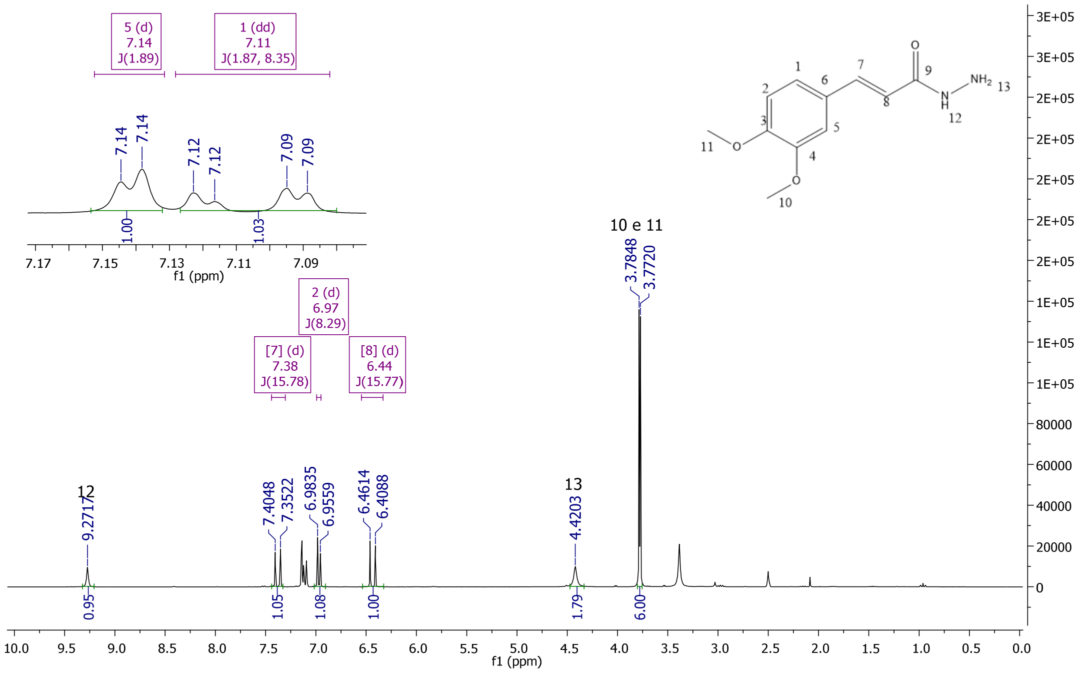

Figure S5. ^{1}H NMR spectrum (300 MHz, DMSO- d_6) of intermediate **10b**.

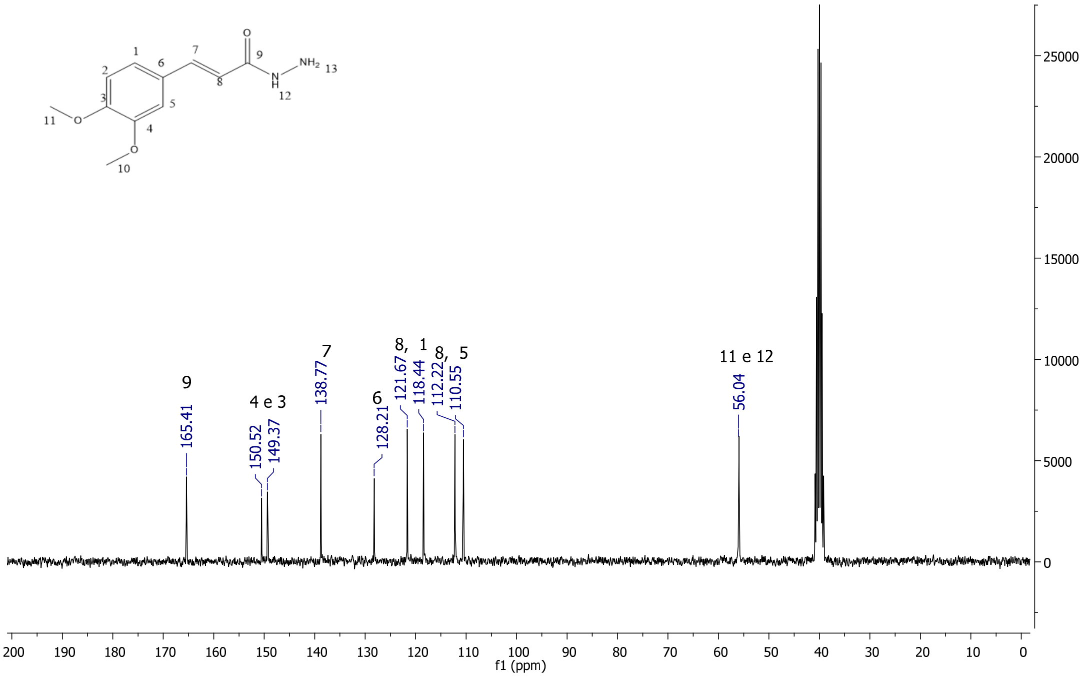

Figure S6. ^{13}C NMR spectrum (75 MHz, DMSO- d_6) of intermediate **10b**.

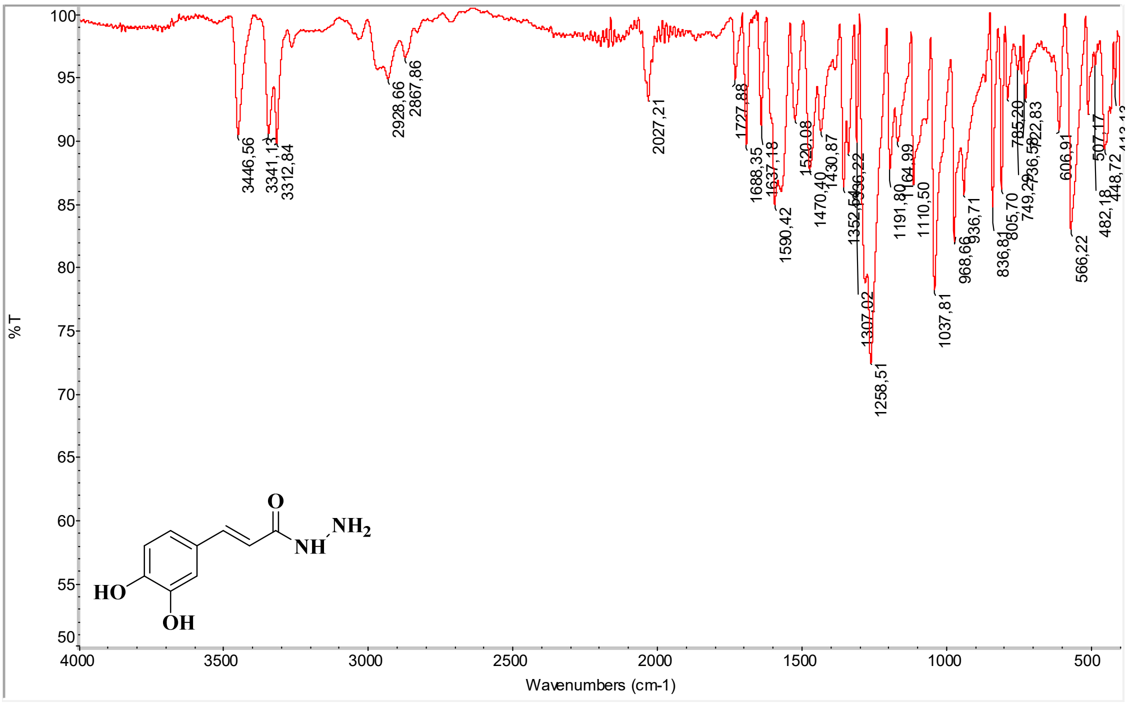

Figure S7. Absorption spectrum in the IR region (ATR) of intermediate **10c**.

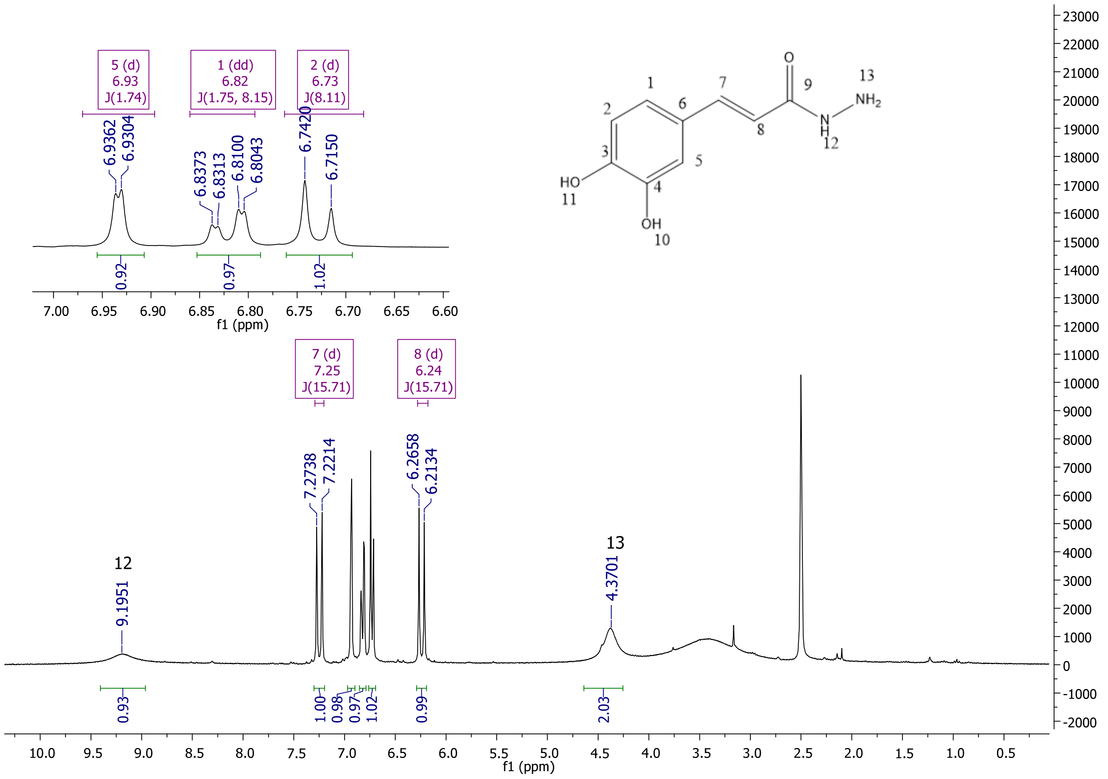

Figure S8. ^{1}H NMR spectrum (300 MHz, DMSO- d_6) of intermediate **10c**.

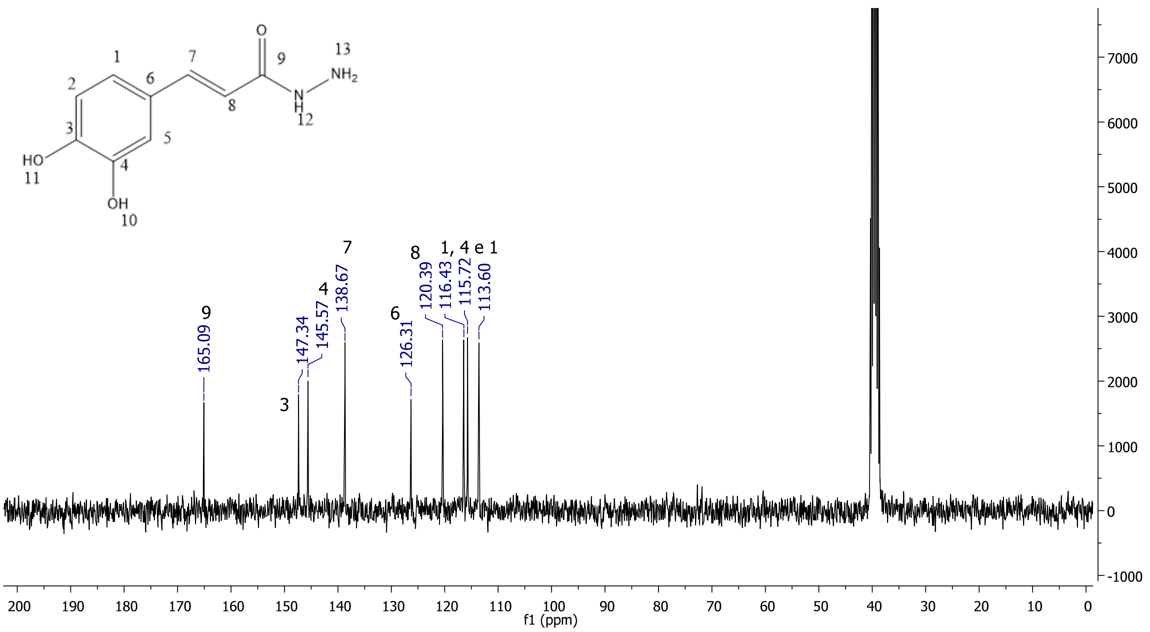

Figure S9. ^{13}C NMR spectrum (75 MHz, DMSO- d_6) of the intermediate **10c**.

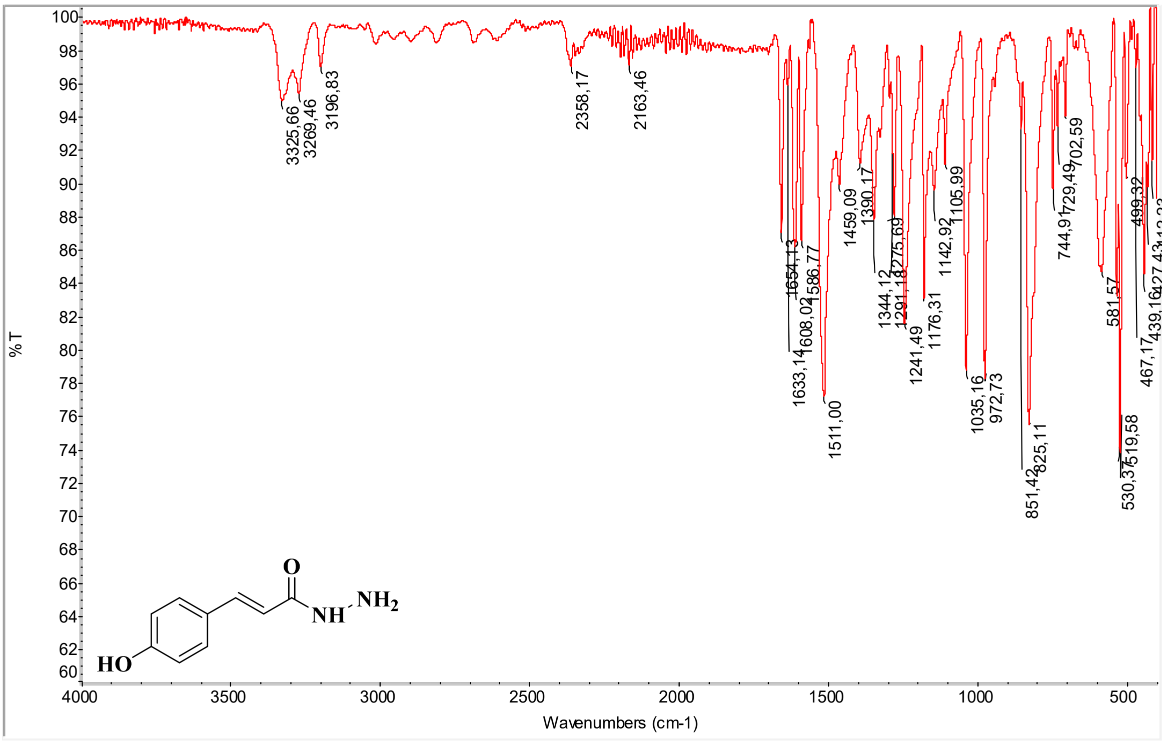

Figure S10. Absorption spectrum in the IR region (ATR) of intermediate **10d**.

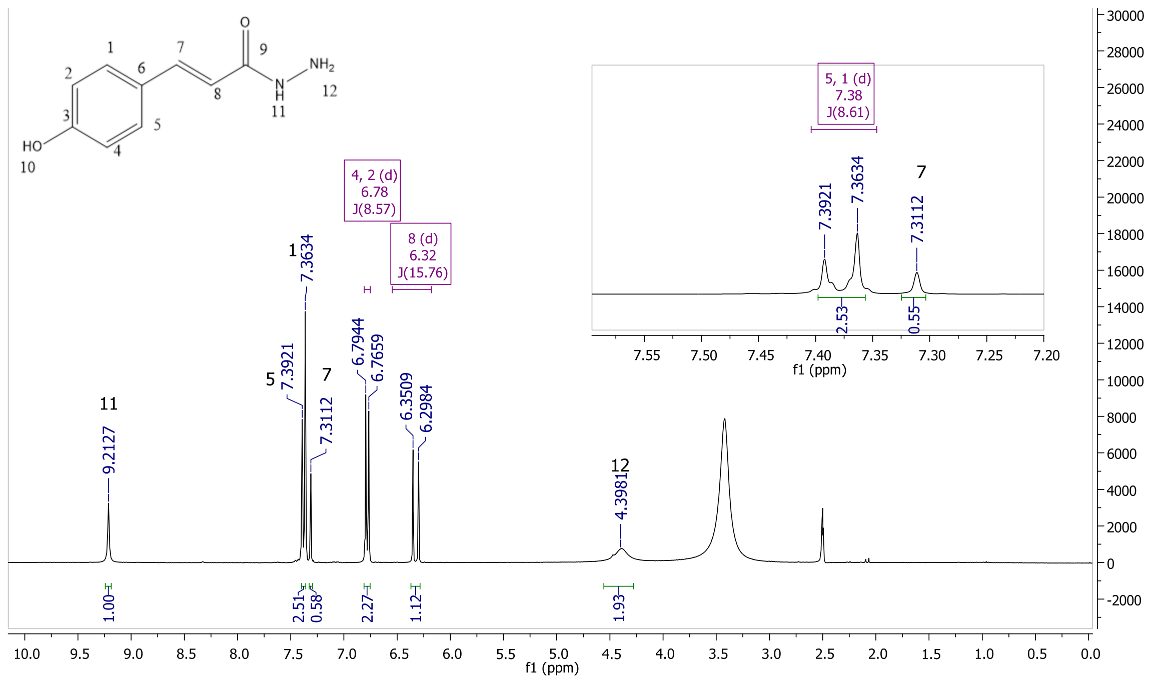

Figure S11. ^{1}H NMR spectrum (300 MHz, DMSO- d_6) of intermediate **10d**.

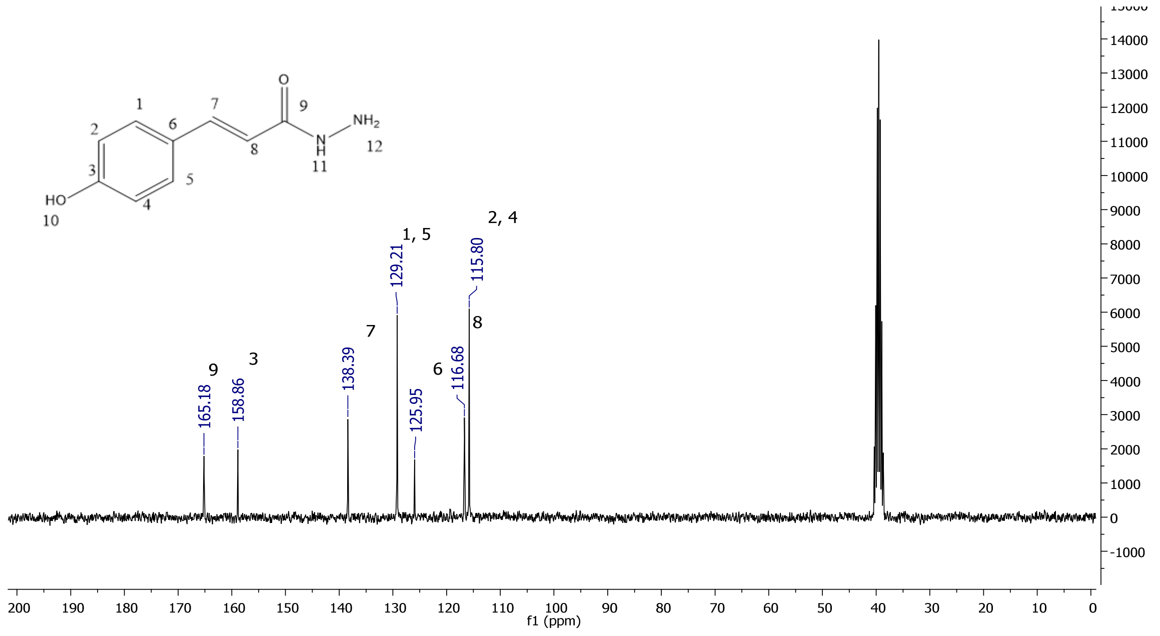

Figure S12. ^{13}C NMR spectrum (75 MHz, DMSO-d_6) of intermediate **10d**.

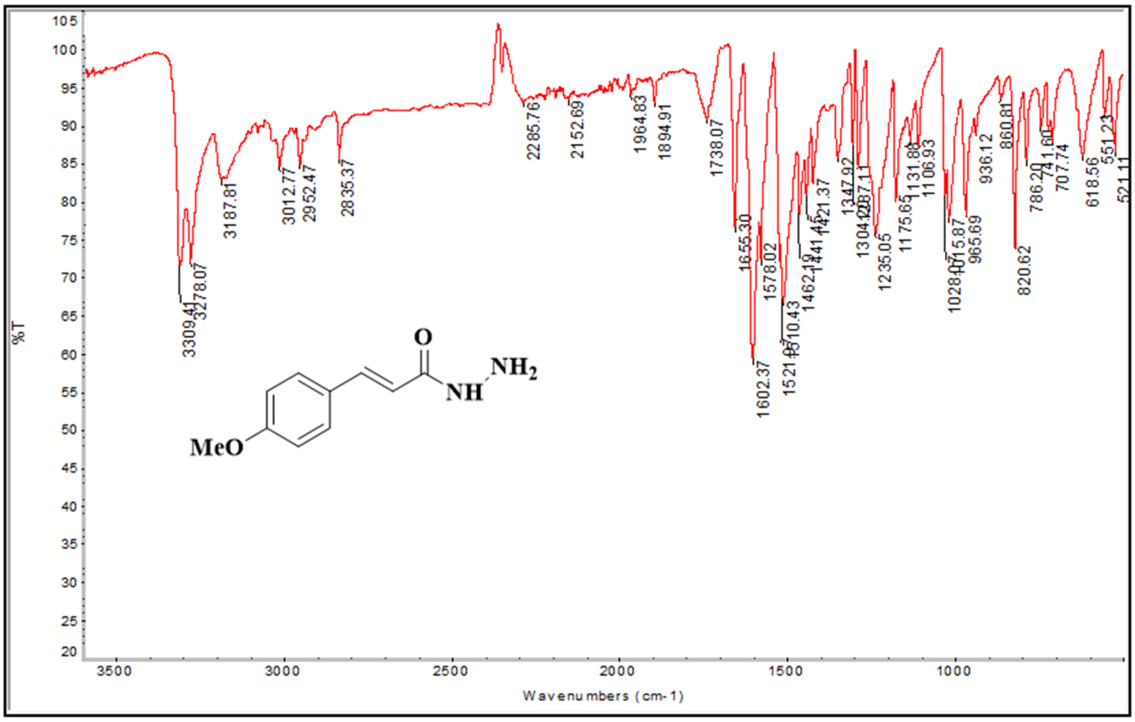

Figure S13. Absorption spectrum in the IR region (ATR) of intermediate **10e**.

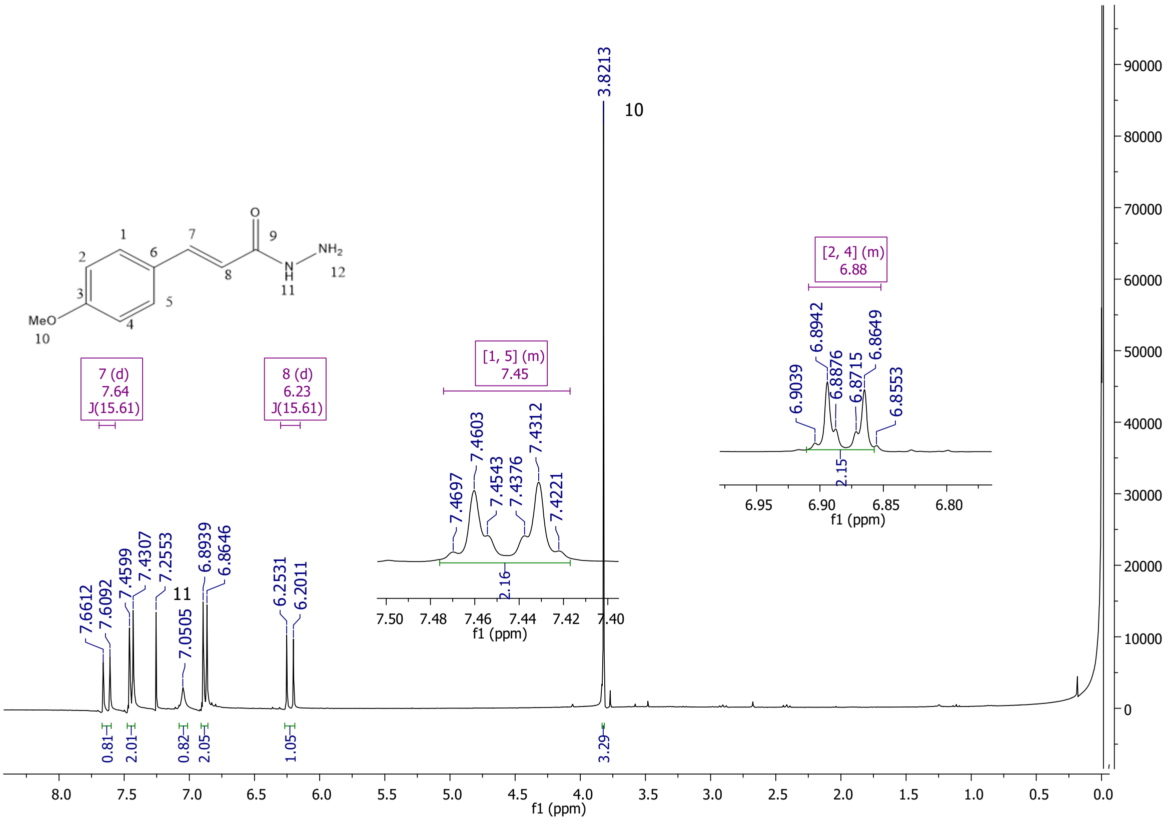

Figure S14. ^{1}H NMR spectrum (300 MHz, $CDCl_3$) of intermediate **10e.**

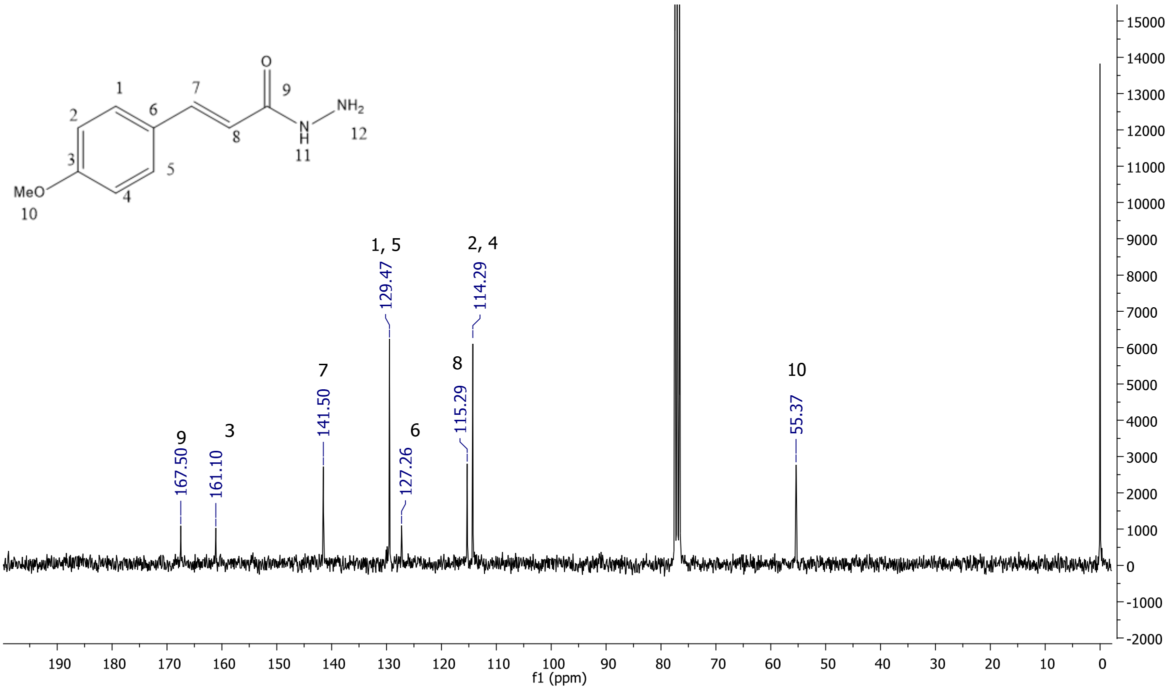

Figure S15. ^{13}C NMR spectrum (75 MHz, $CDCl_3$) of intermediate **10e**.

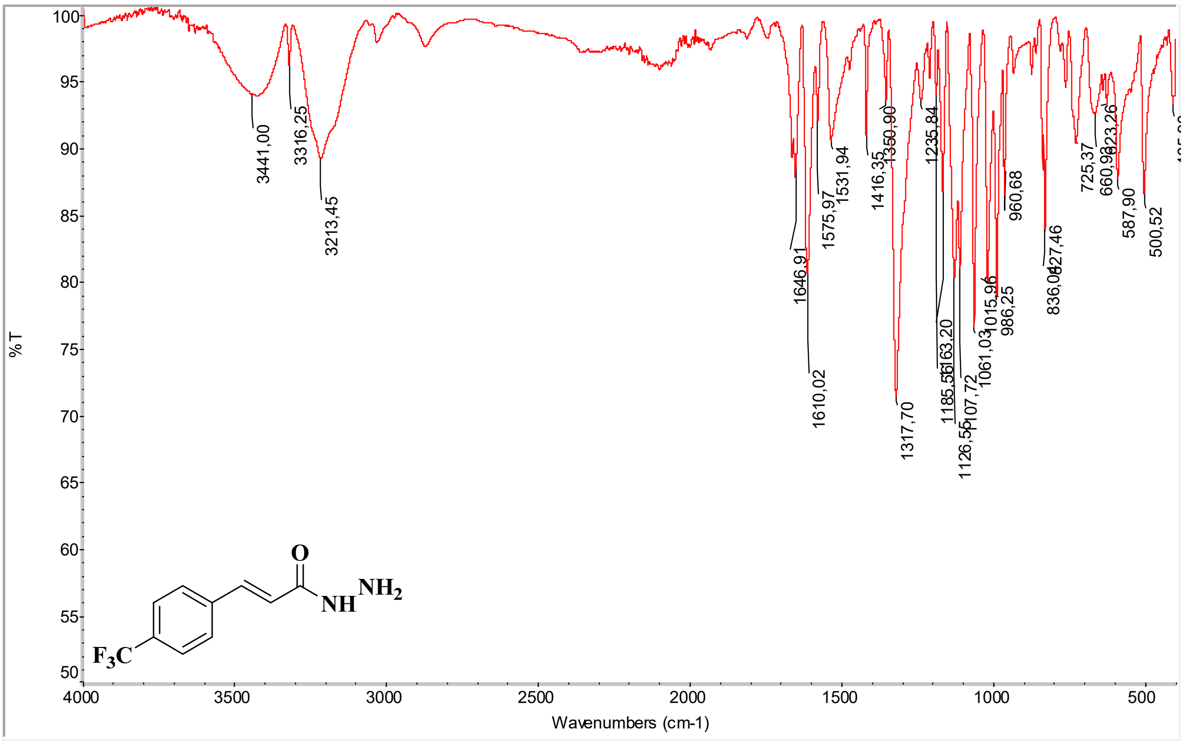

Figure S16. Absorption spectrum in the IR region (ATR) of intermediate **10f**.

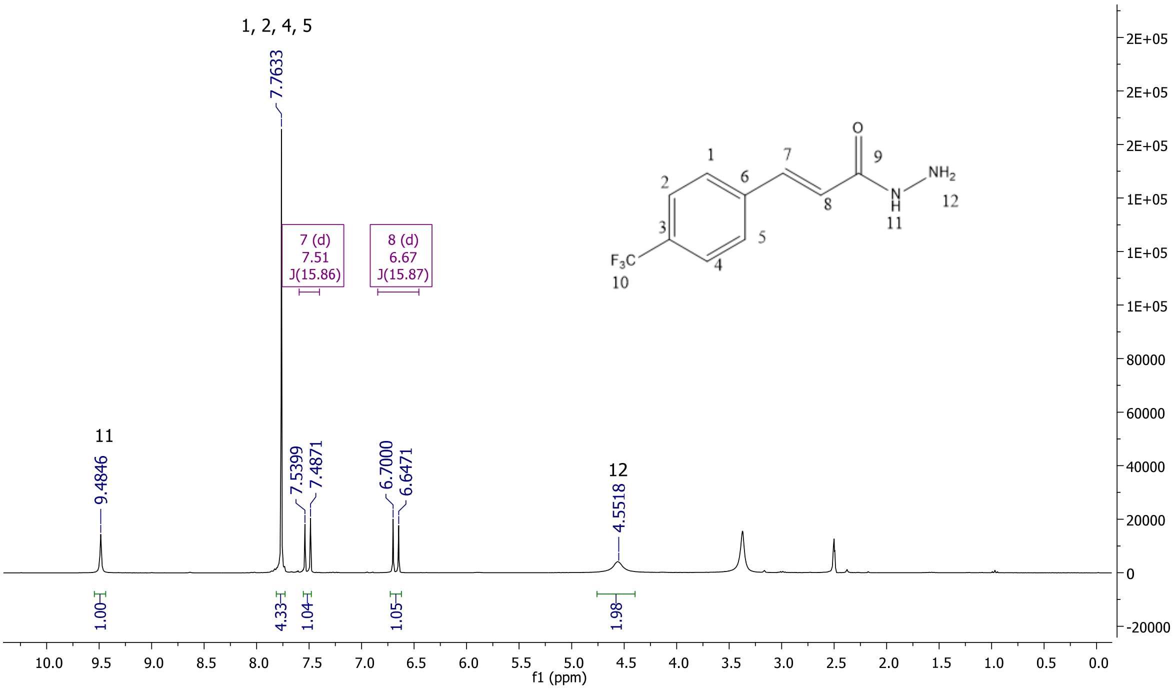

Figure S17. ^{1}H NMR spectrum (300 MHz, DMSO-d_6) of intermediate **10f.**

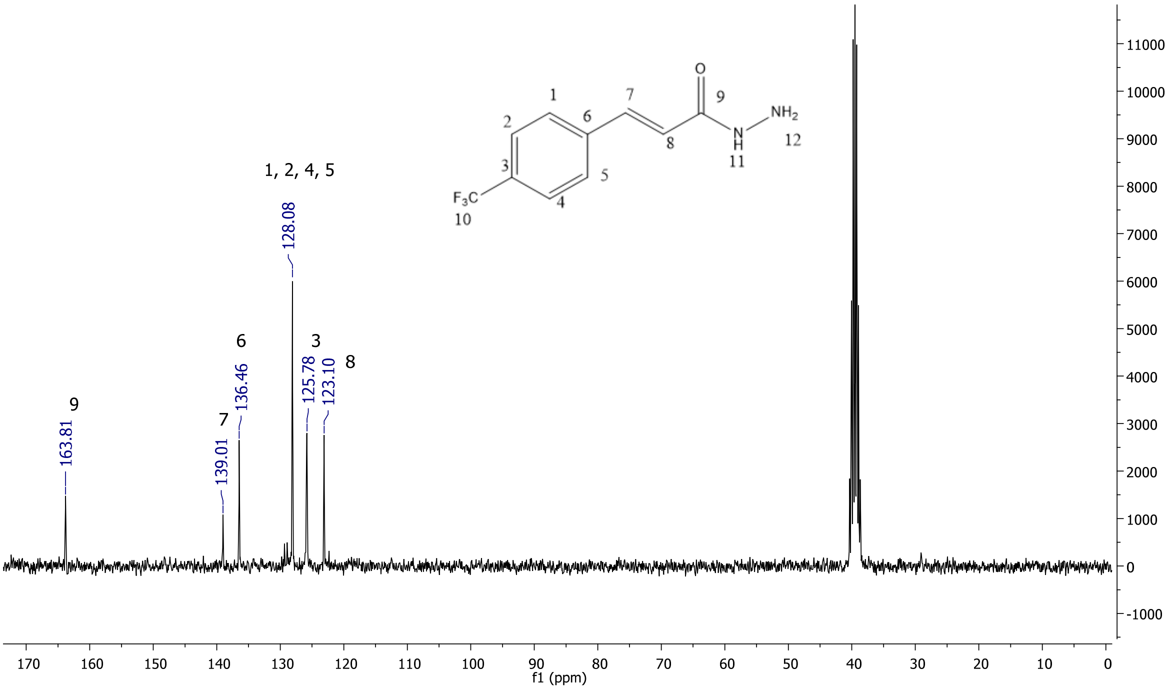

Figure S18. ^{13}C NMR spectrum (75 MHz, DMSO-d_6) of intermediate **10f**.

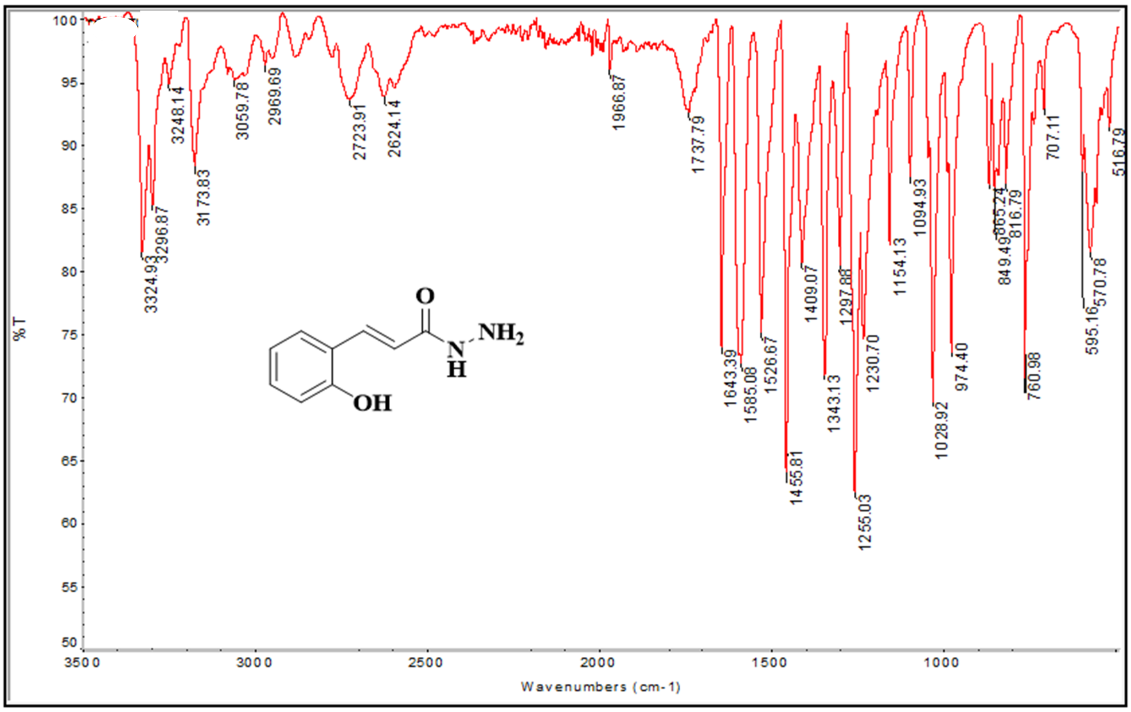

Figure S19. Absorption spectrum in the IR region (ATR) of intermediate **10g**.

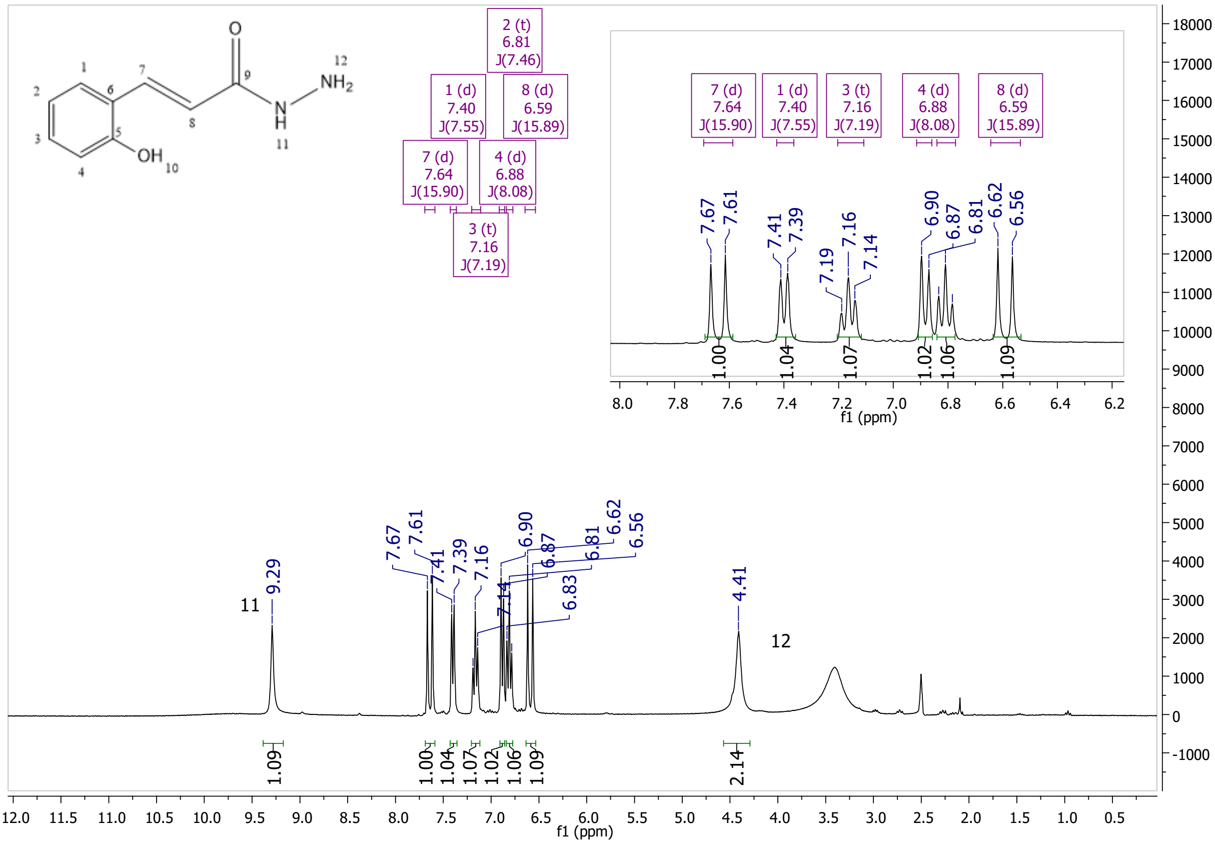

Figure S20. ^{1}H NMR spectrum (300 MHz, DMSO-d_6) of intermediate **10g**.

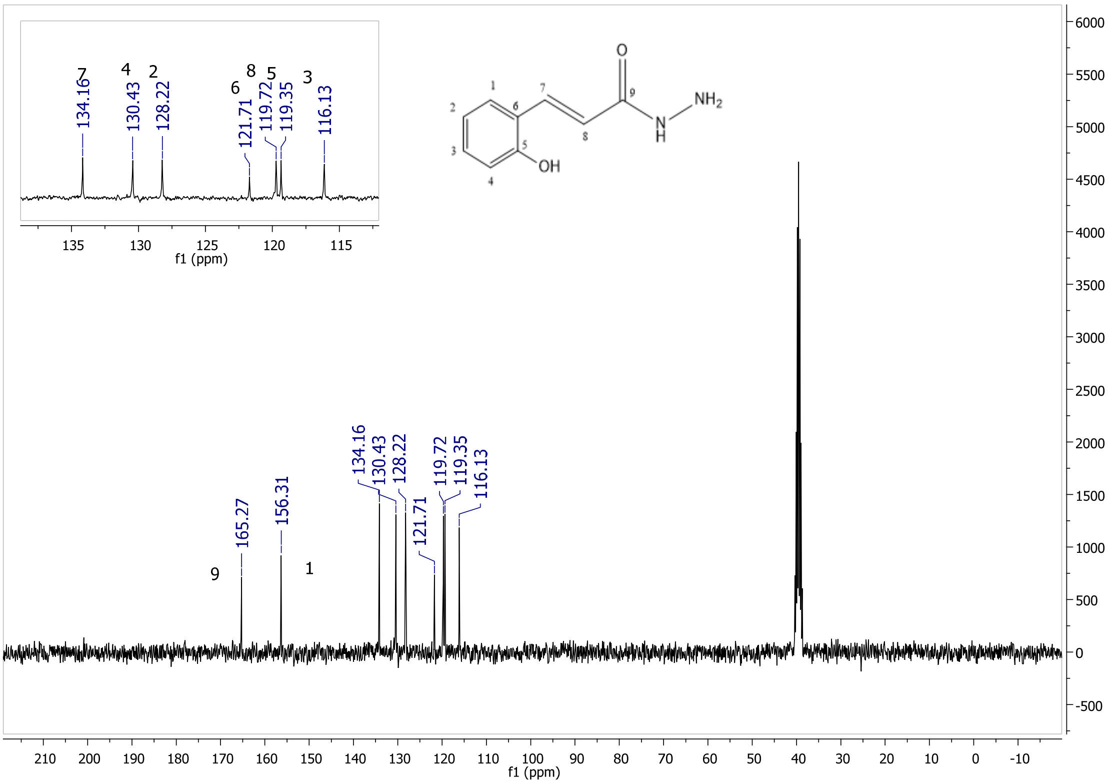

Figure S21. ^{13}C NMR spectrum (75 MHz, DMSO-d_6) of intermediate **10g**.

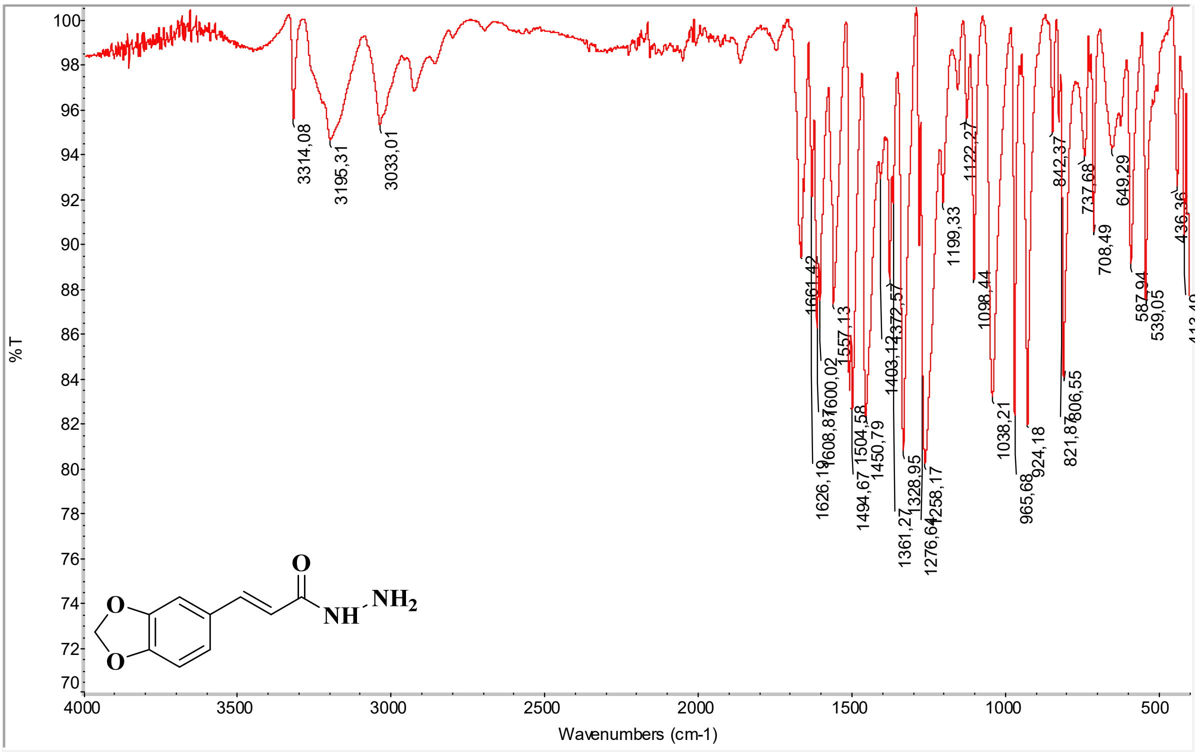

Figure S22. Absorption spectrum in the IR region (ATR) of intermediate **10h**.

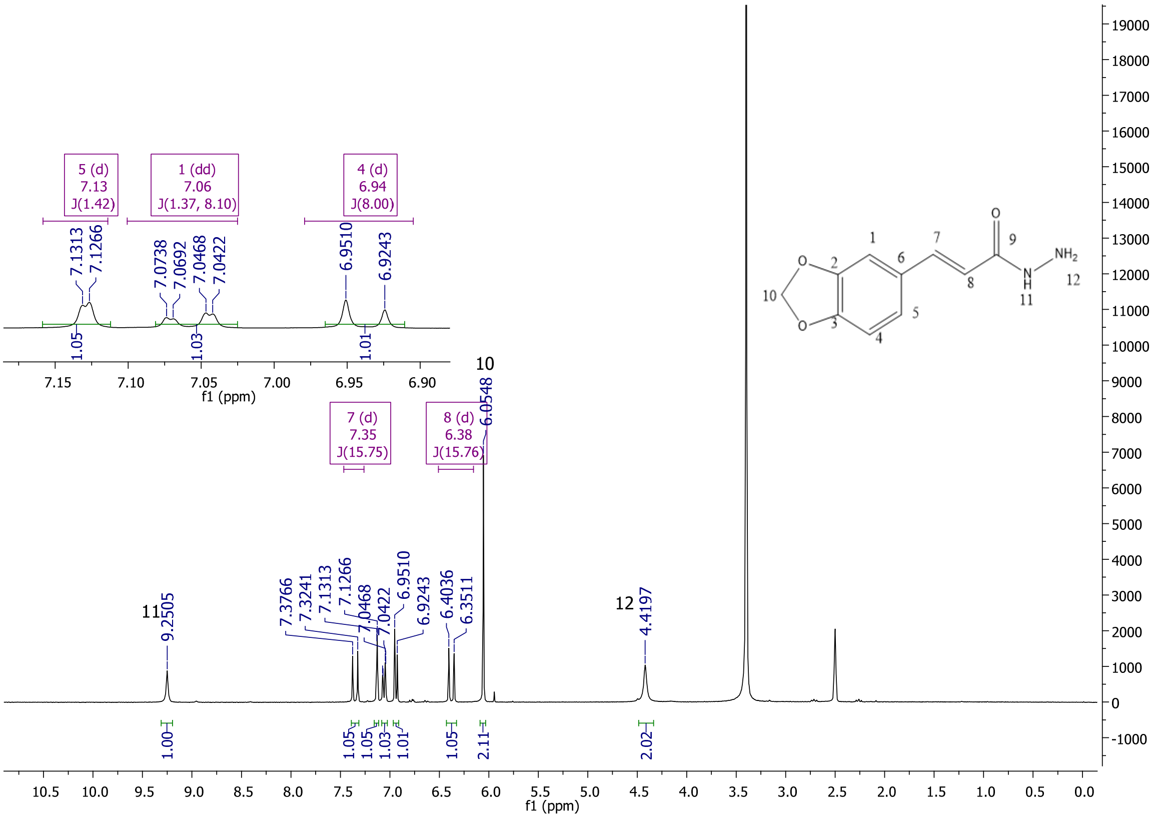

Figure S23. ^{1}H NMR spectrum (300 MHz, DMSO-d_6) of intermediate **10h**.

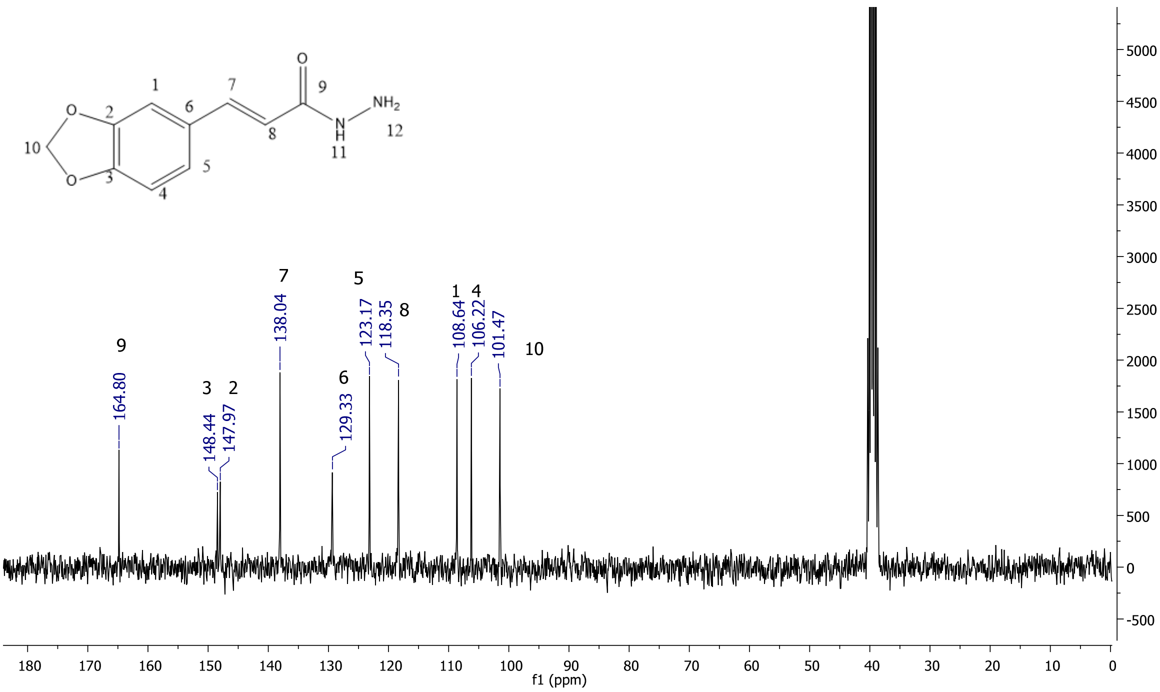

Figure S24. ^{13}C NMR spectrum (75 MHz, DMSO-d_6) of intermediate**10h**.

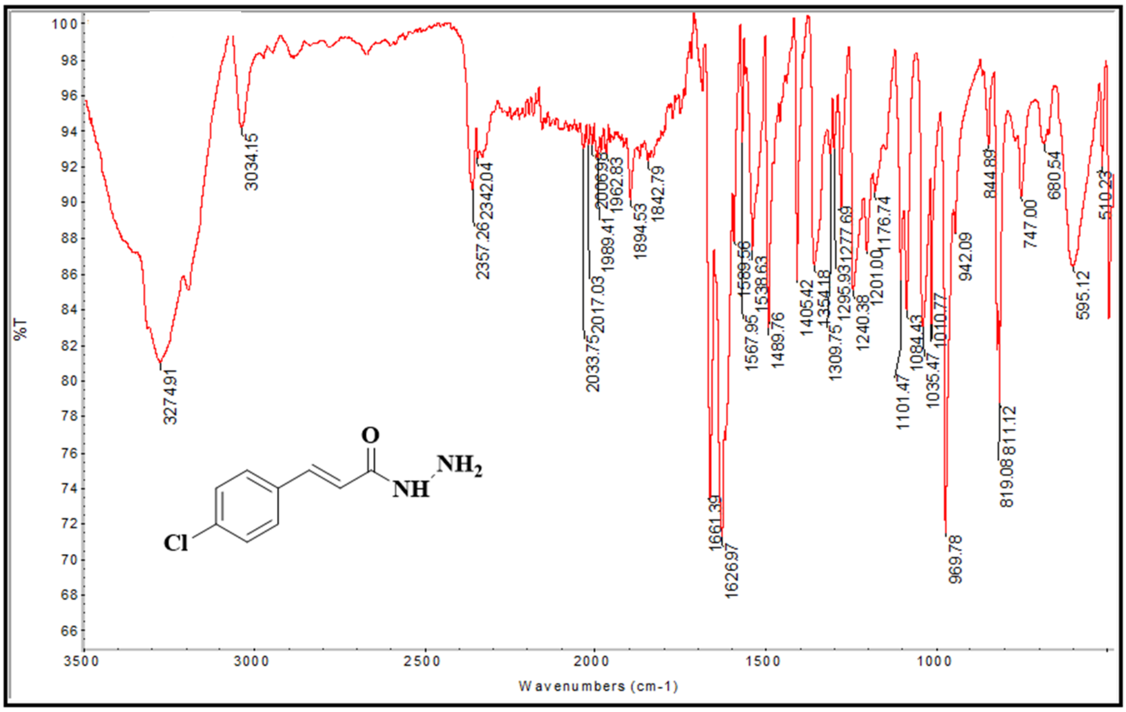

Figure S25. Absorption spectrum in the IR region (ATR) of intermediate **10i**.

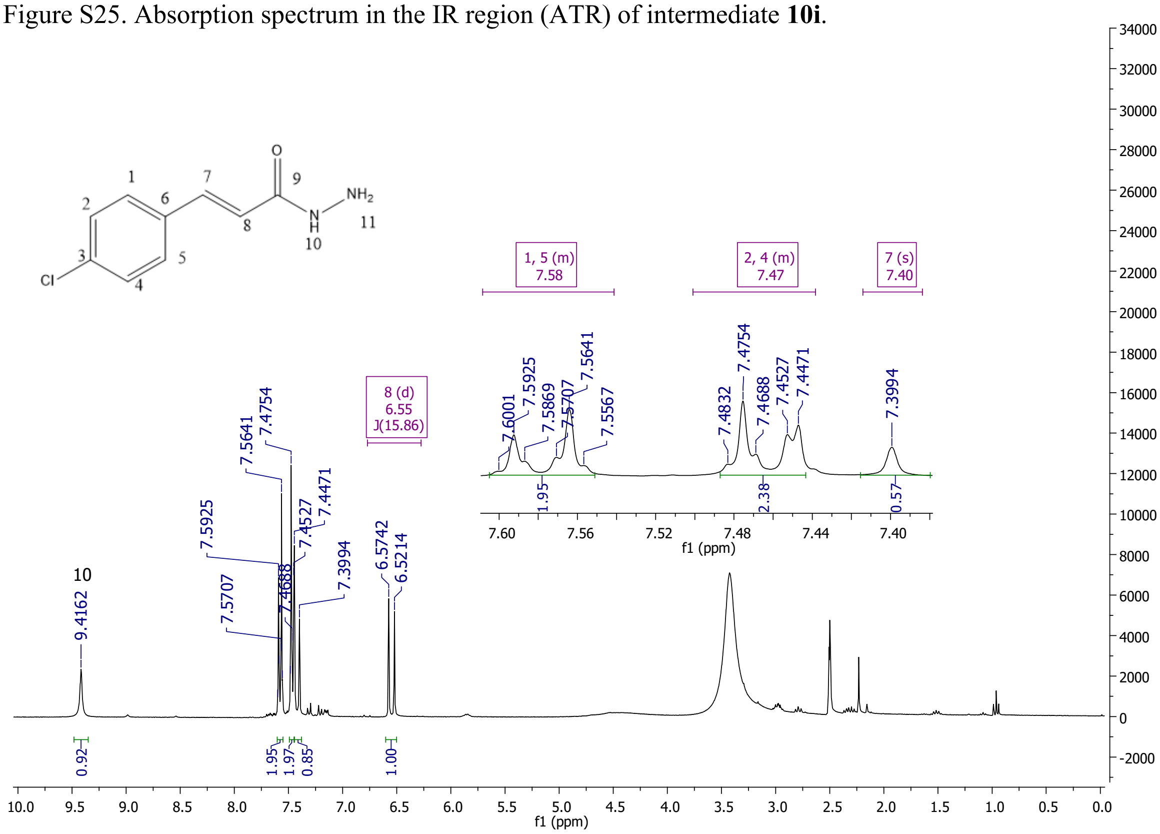

Figure S26. ^{1}H NMR spectrum (300 MHz, DMSO-d_6) of intermediate **10i**.

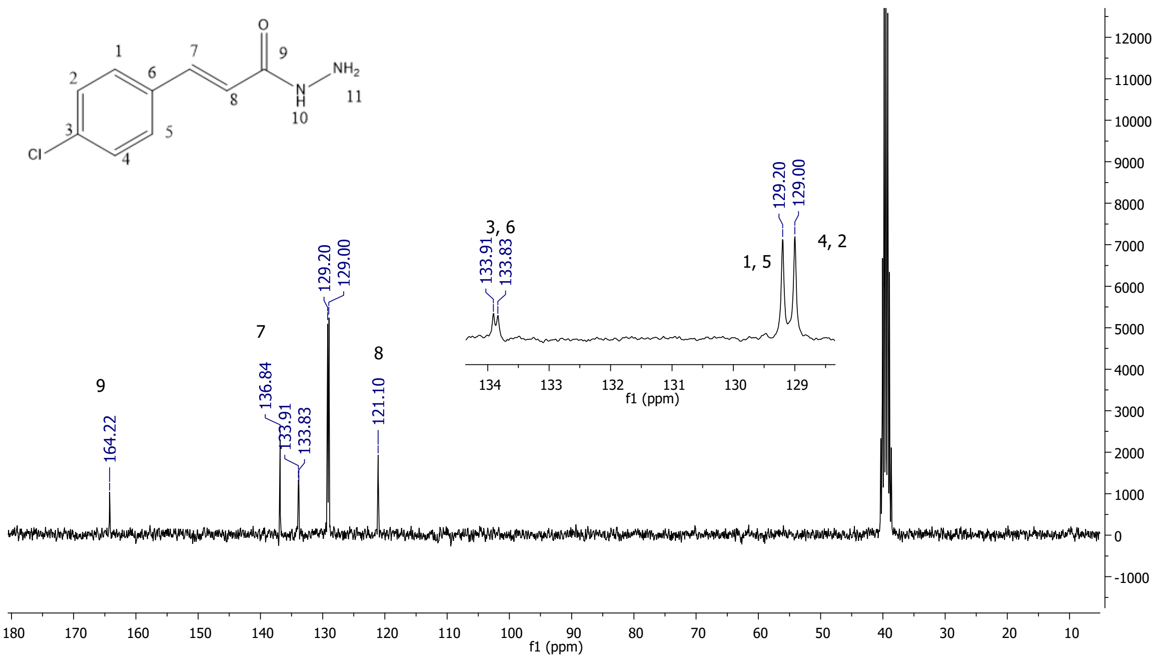

Figure S27. ^{13}C NMR spectrum (75 MHz, DMSO-d_6) of intermediate **10i.**

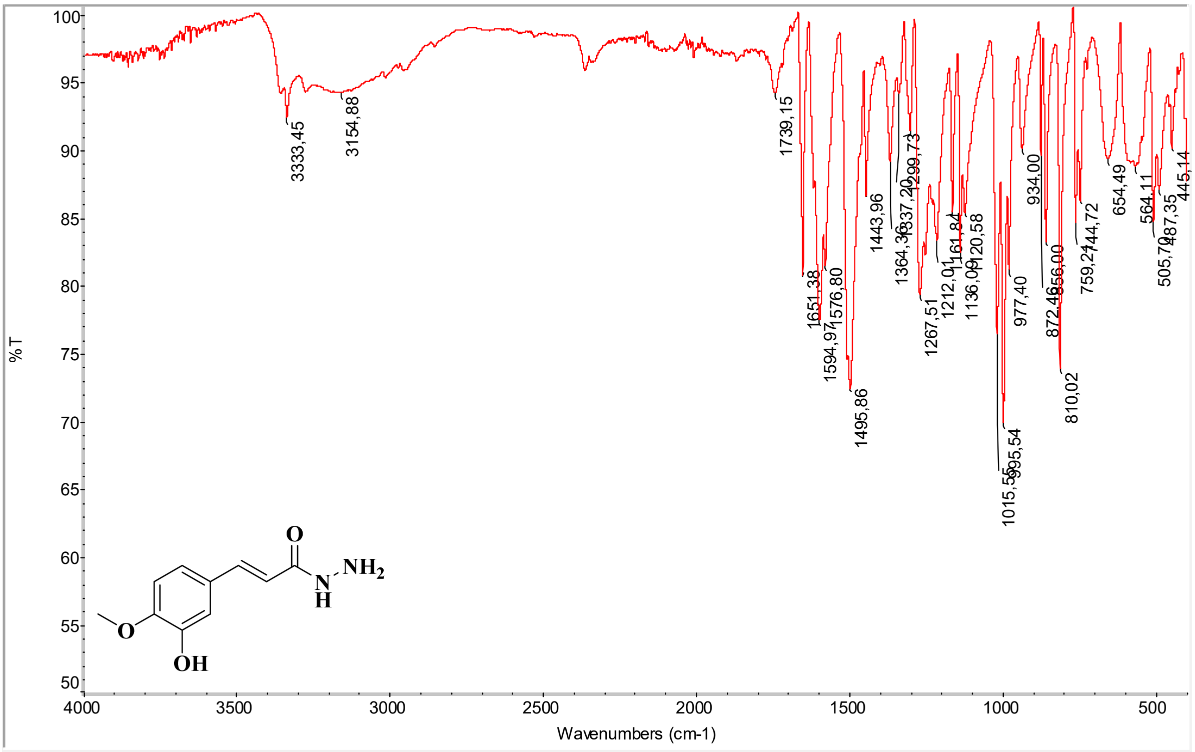

Figure S28. Absorption spectrum in the IR region (ATR) of intermediate **10j**.

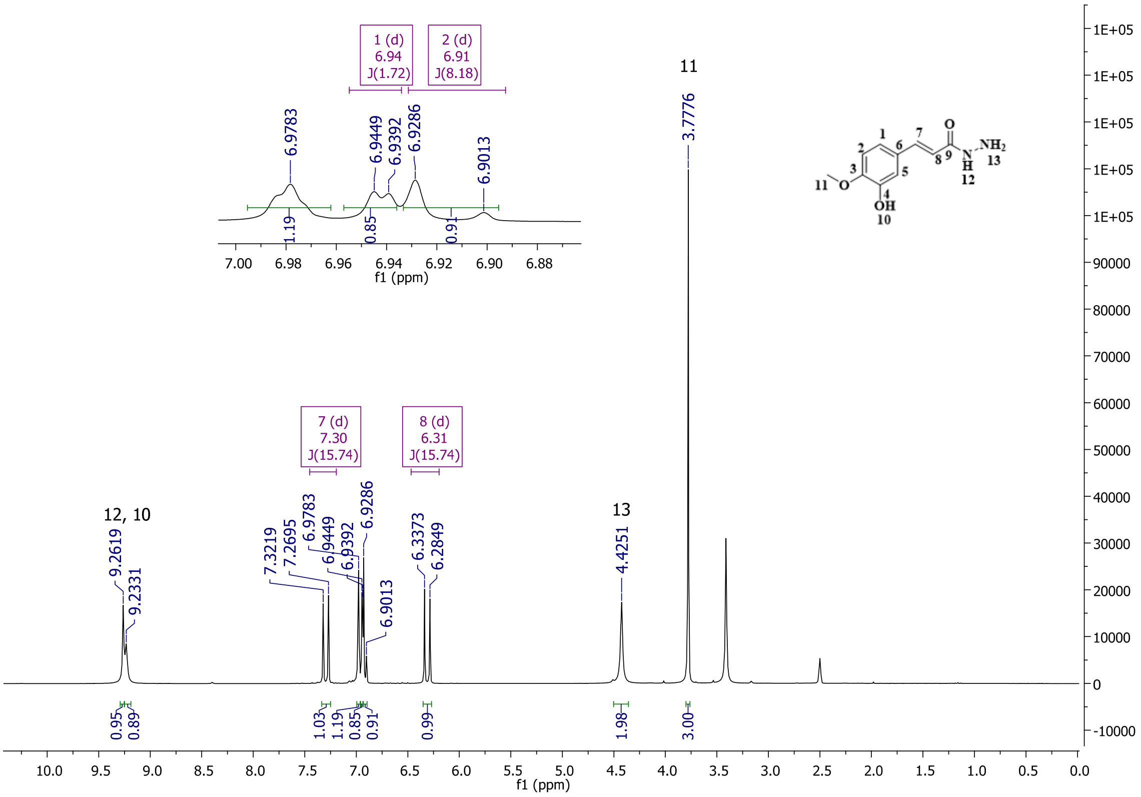

Figure S29. ^{1}H NMR spectrum (300 MHz, DMSO-d_6) of intermediate **10j.**

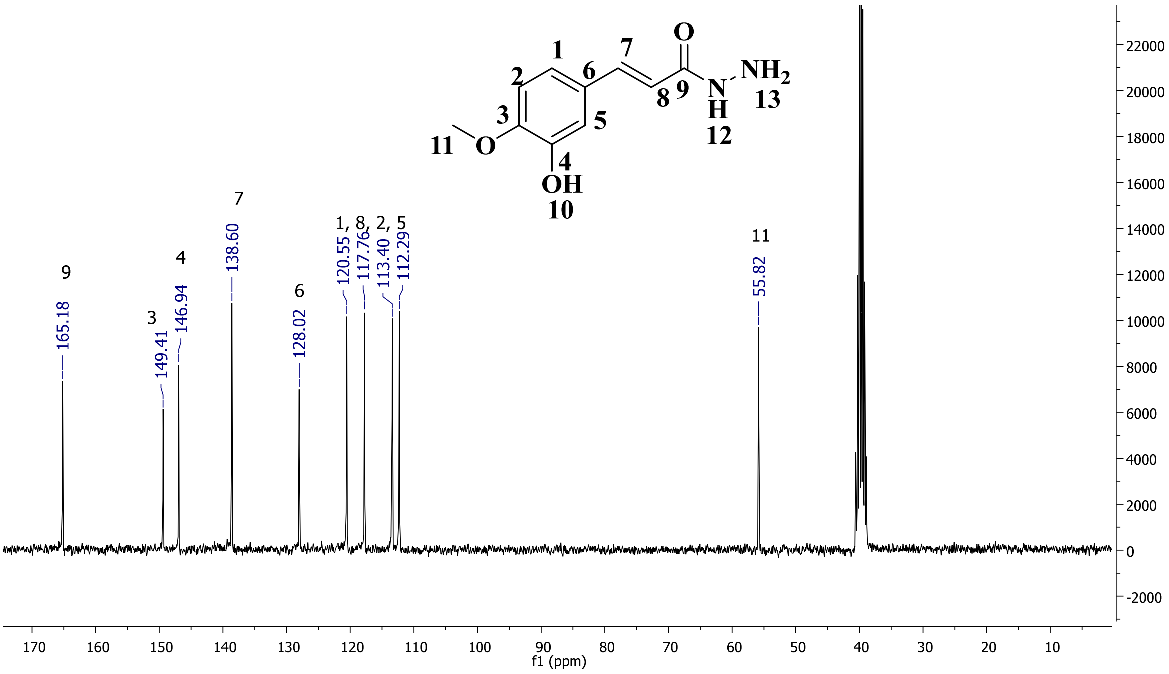

Figure S30. ^{13}C NMR spectrum (75 MHz, DMSO-d_6) of intermediate **10j**.

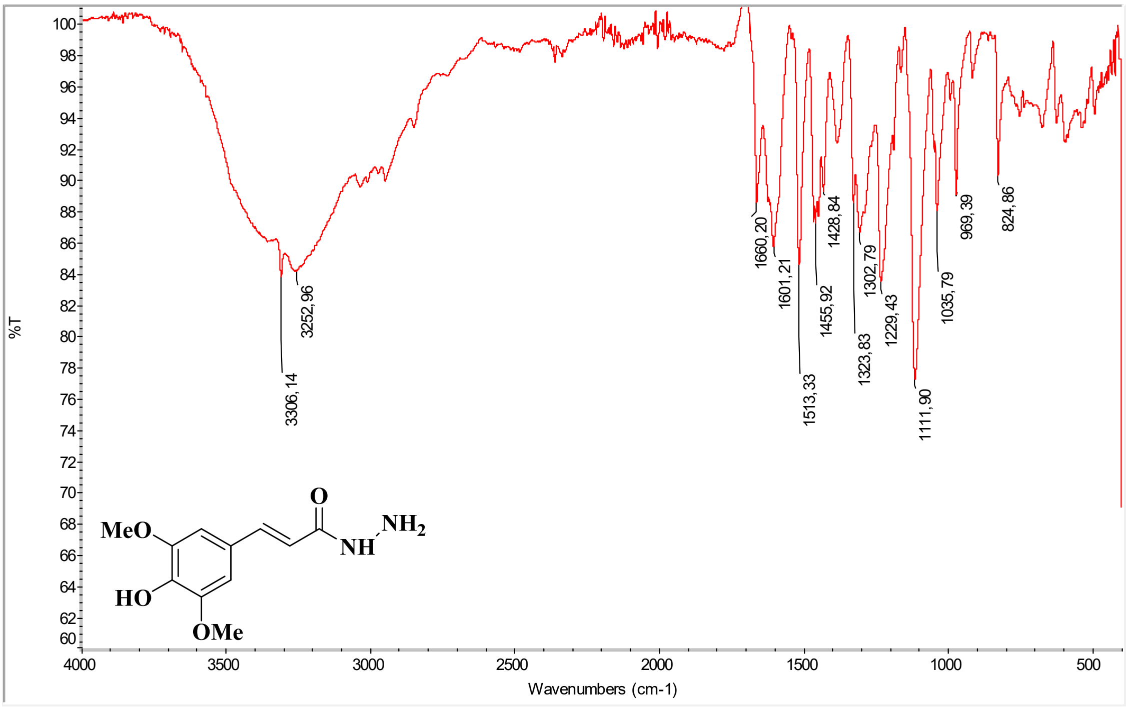

Figure S31. Absorption spectrum in the IR region (ATR) of intermediate **10k.**

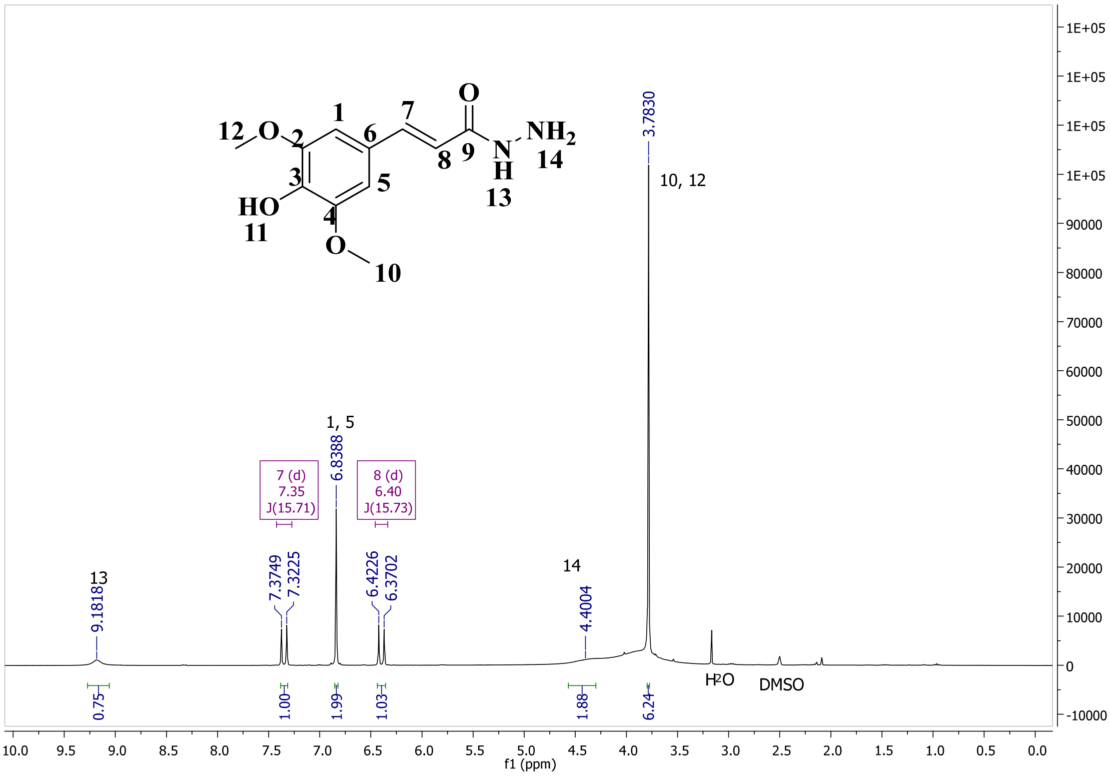

Figure S32. ^{1}H NMR spectrum (300 MHz, DMSO-d_6) of intermediate **10k**.

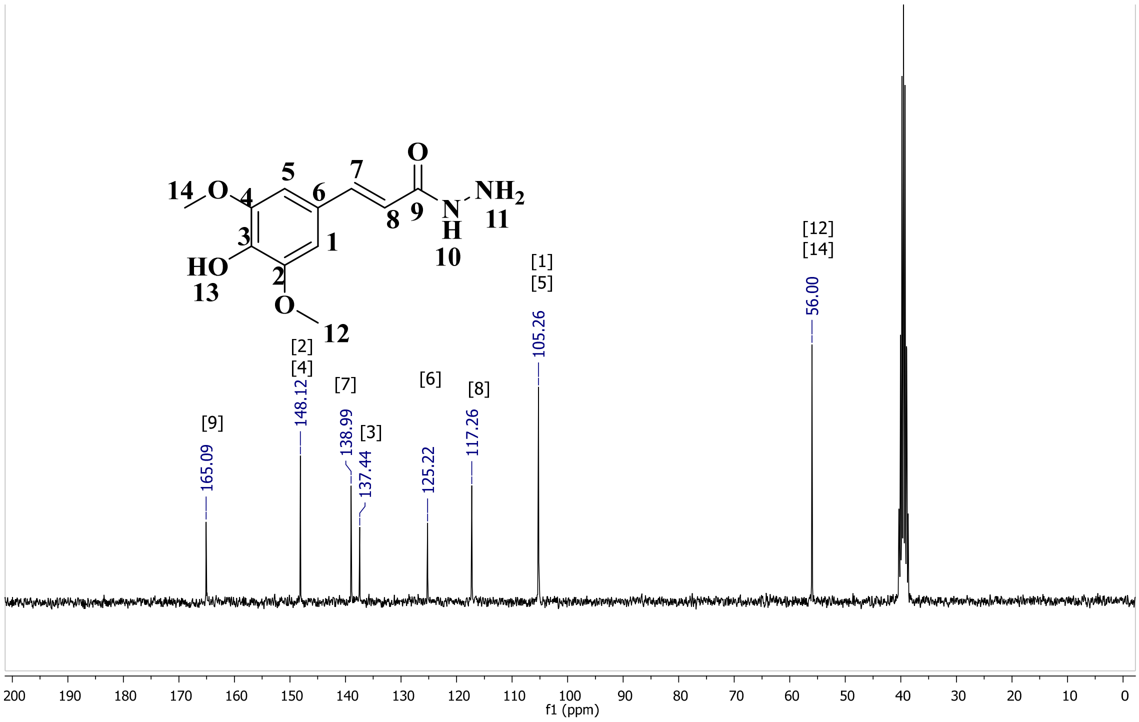

Figure S33. ^{13}C NMR spectrum (75 MHz, DMSO-d_6) of intermediate **10k**.

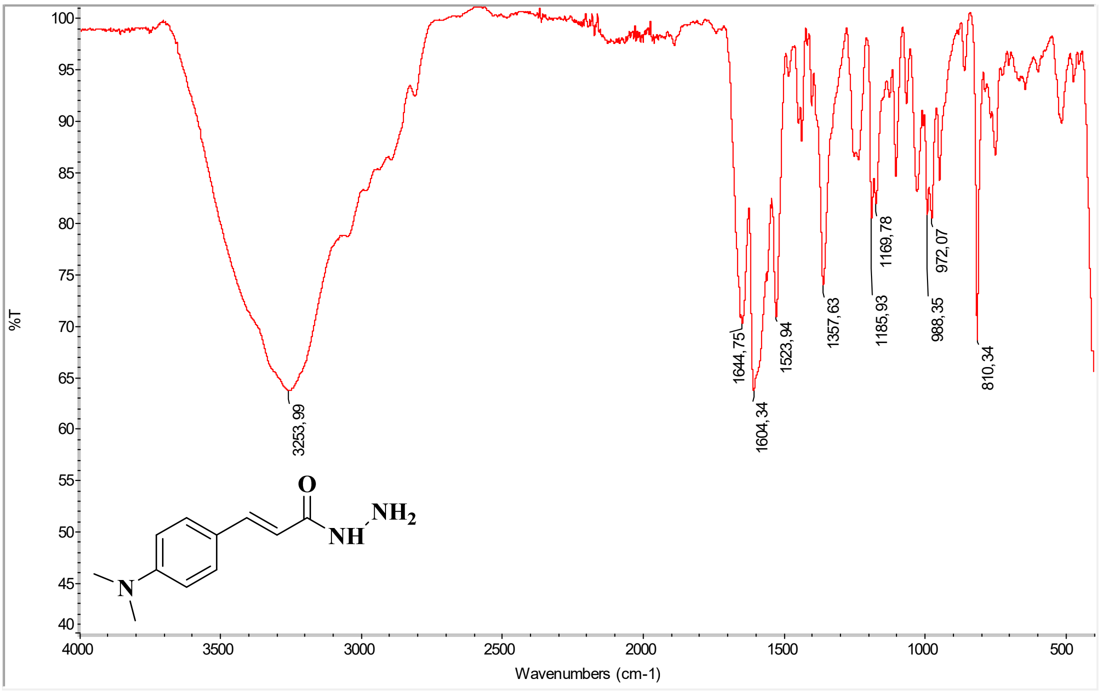

Figure S34. Absorption spectrum in the IR region (ATR) of intermediate **10l**.

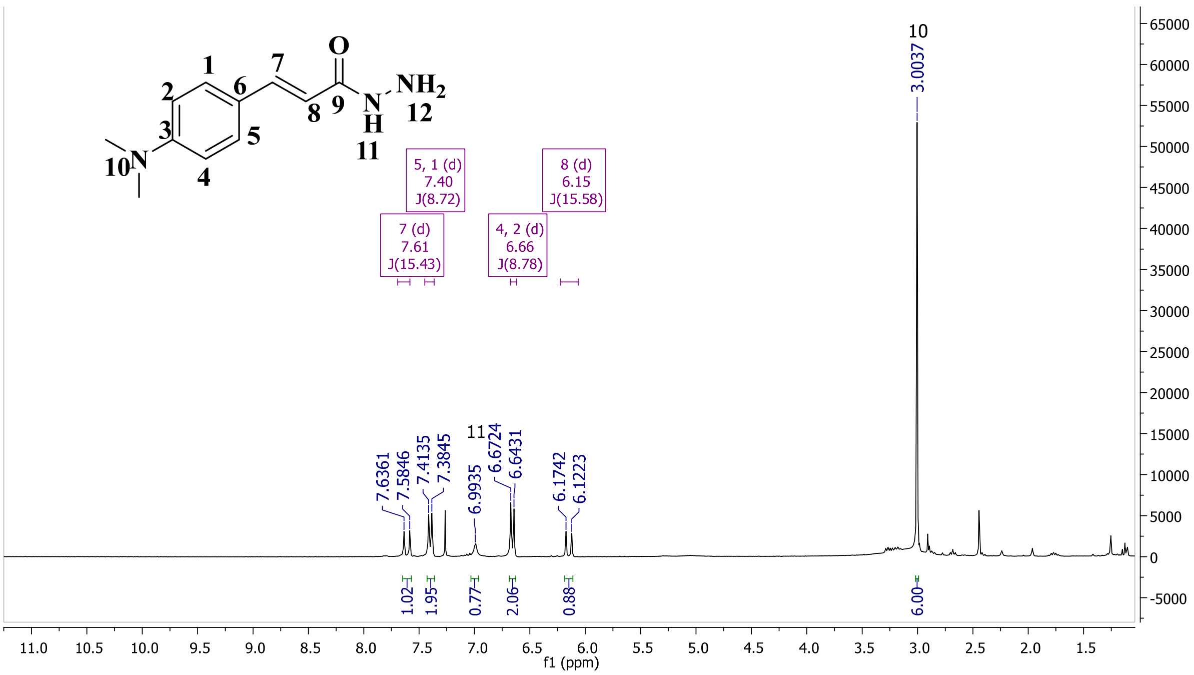

Figure S35. ^{1}H NMR spectrum (300 MHz, $CDCl_3$) of intermediate **10l**.

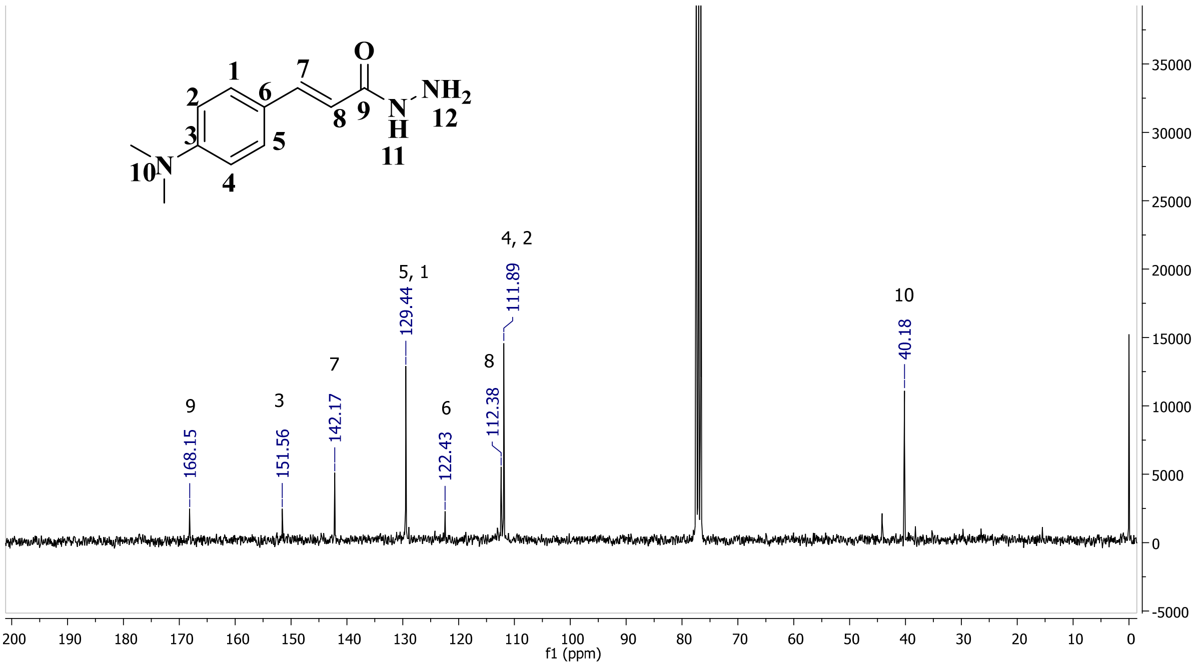

Figure S36. ^{13}C NMR spectrum (75 MHz, $CDCl_3$) of intermediate **10l**.

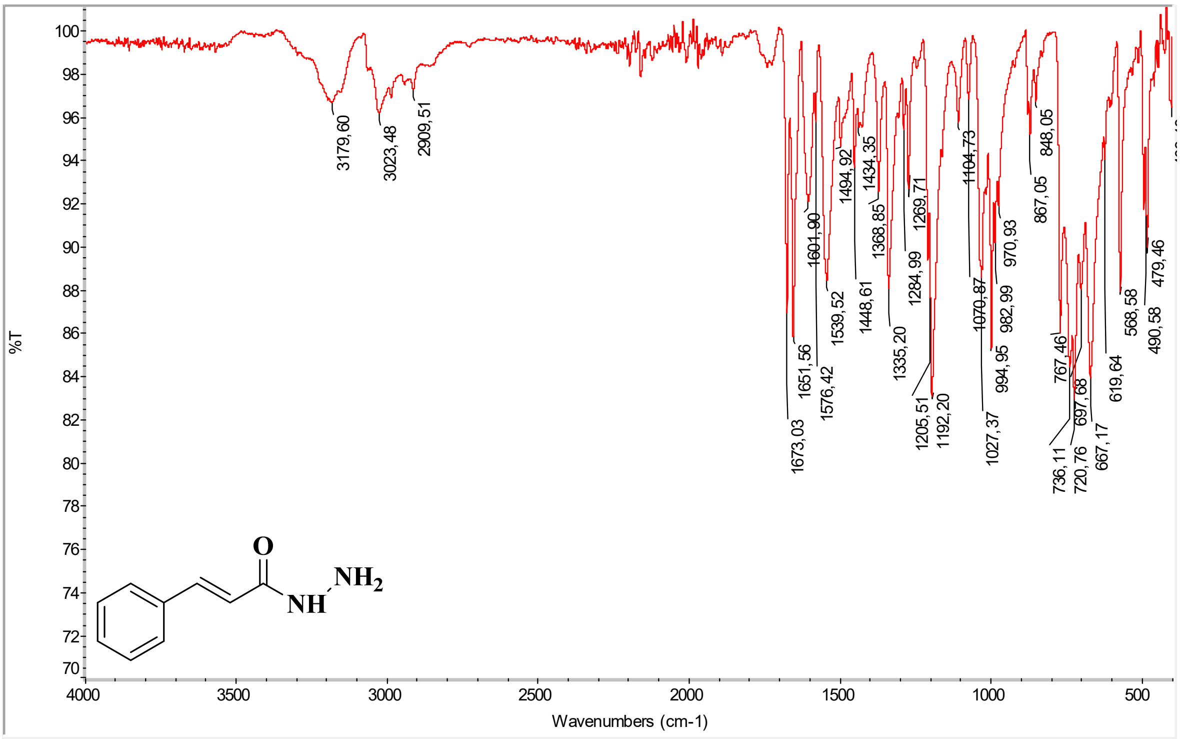

Figure S37. Absorption spectrum in the IR region (ATR) of intermediate **10m**.

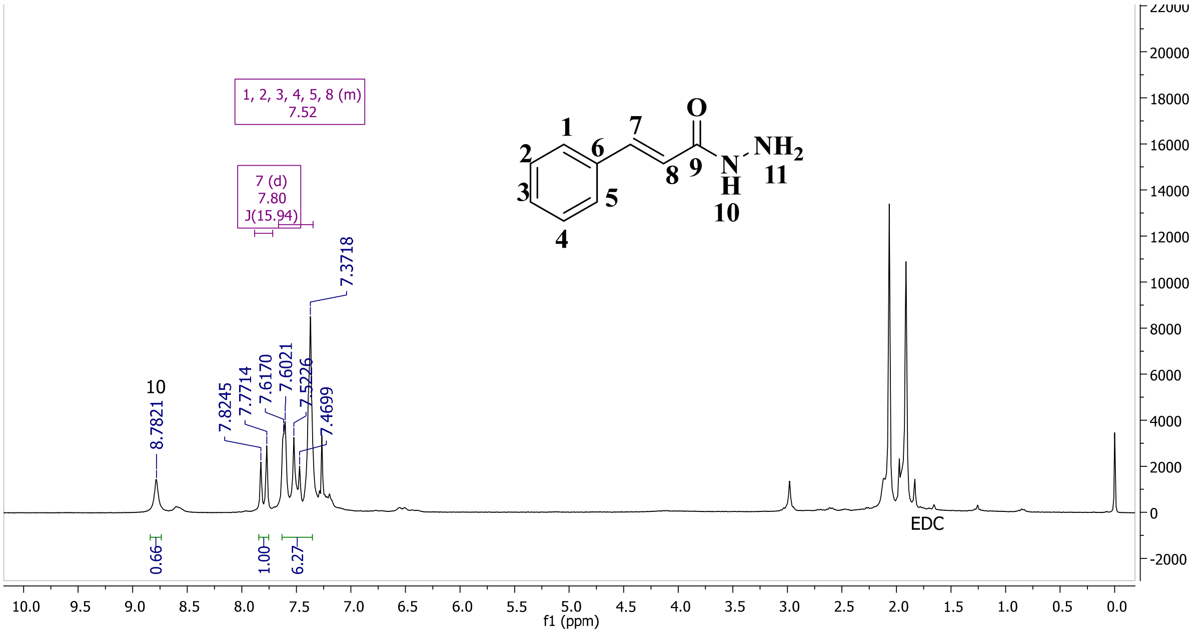

Figure S38. ^{1}H NMR spectrum (300 MHz, $CDCl_3$) of intermediate **10m**.

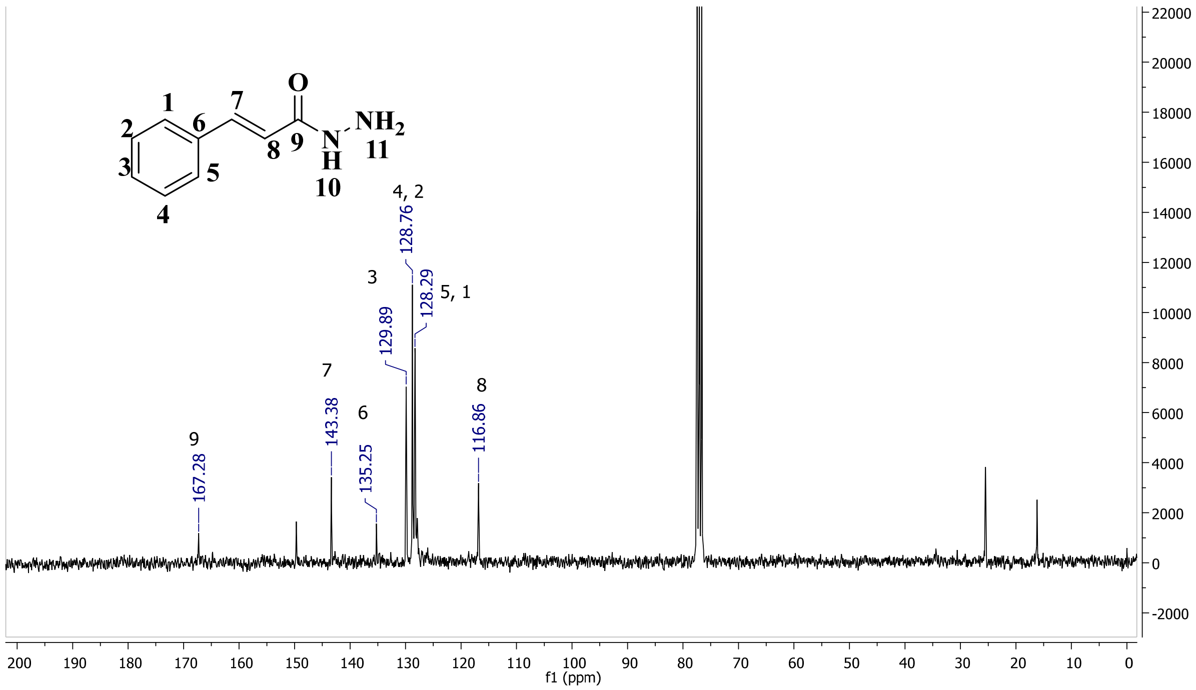

Figure S39. ^{13}C NMR spectrum (75 MHz, $CDCl_3$) of intermediate **10m**.

S2. Absorption in the IR region, NMR Spectrums, Mass spectrum, and HPLC chromatogram of final compounds

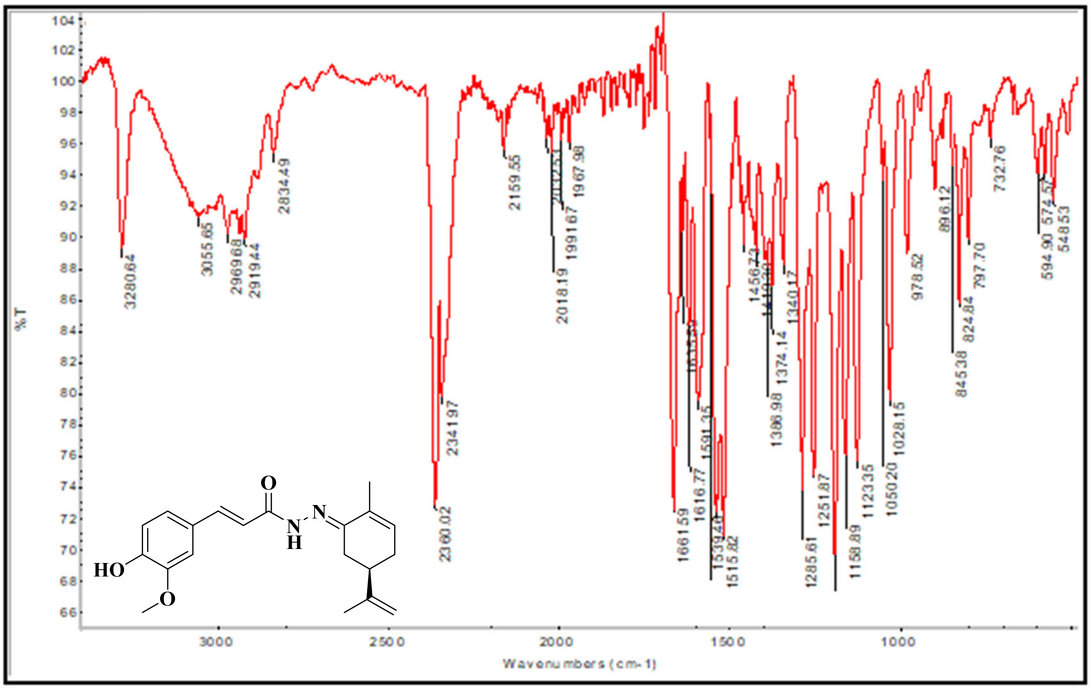

Figure S40. Absorption spectrum in the IR region (ATR) of intermediate **PQM273**.

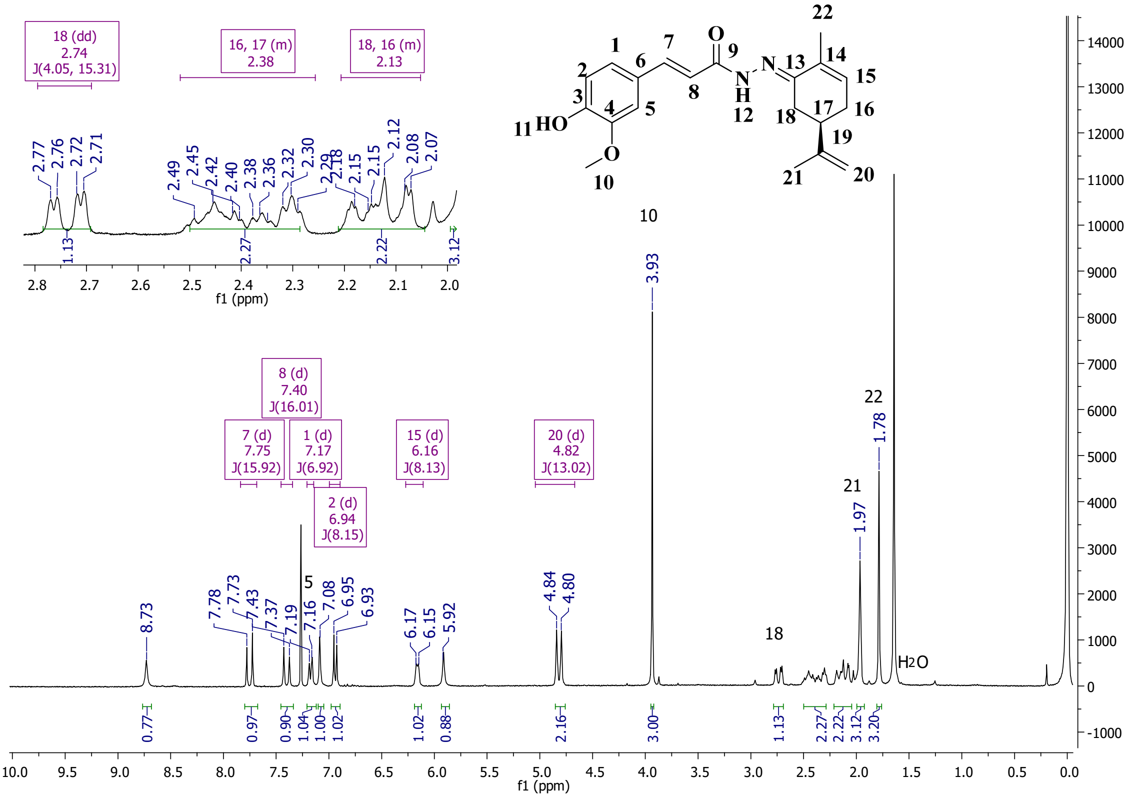

Figure S41. ^{1}H NMR spectrum (300 MHz, $CDCl_3$) of compound **PQM273**.

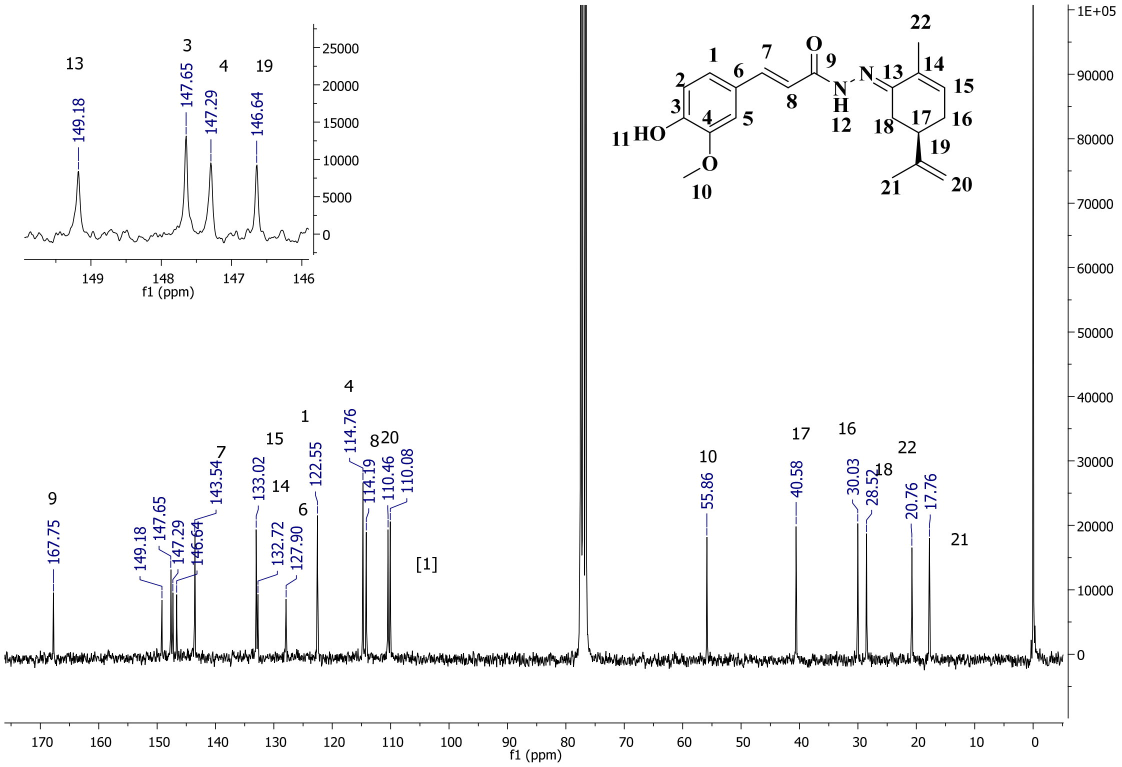

Figure S42. ^{13}C NMR spectrum (75 MHz, $CDCl_3$) of compound **PQM273.**

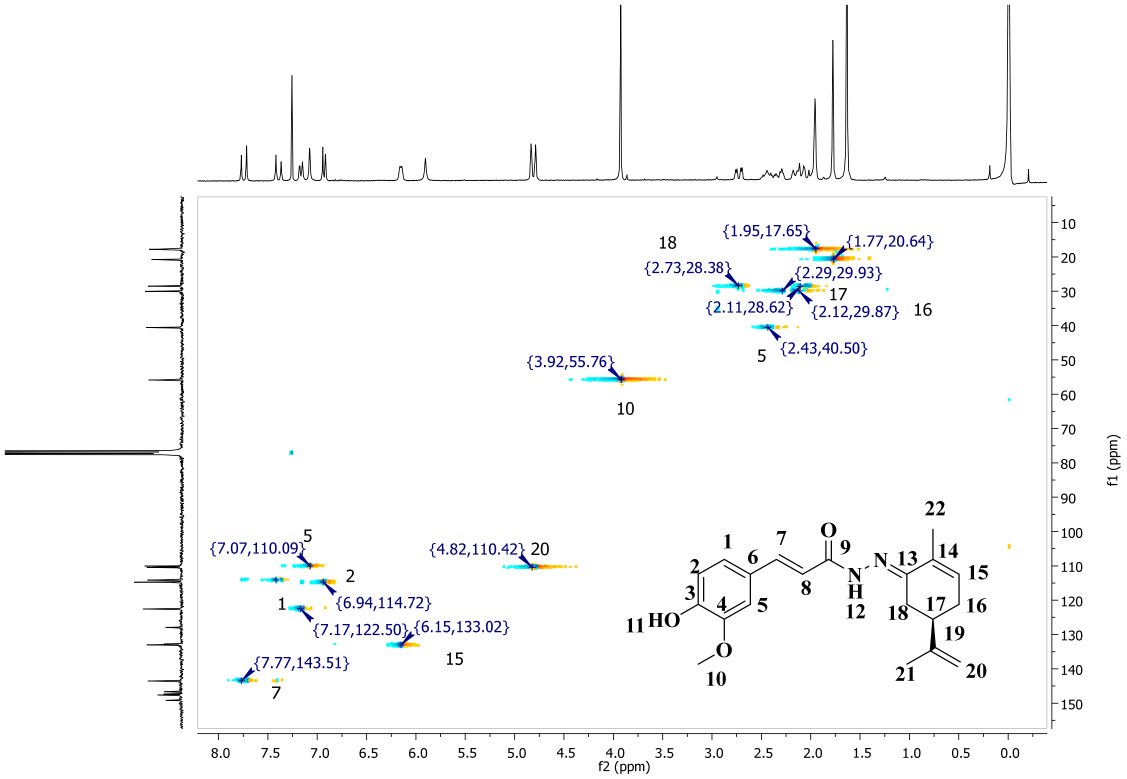

Figure S43. HSQC correlation map of compound **PQM273** (300 MHz, $CDCl_3$).

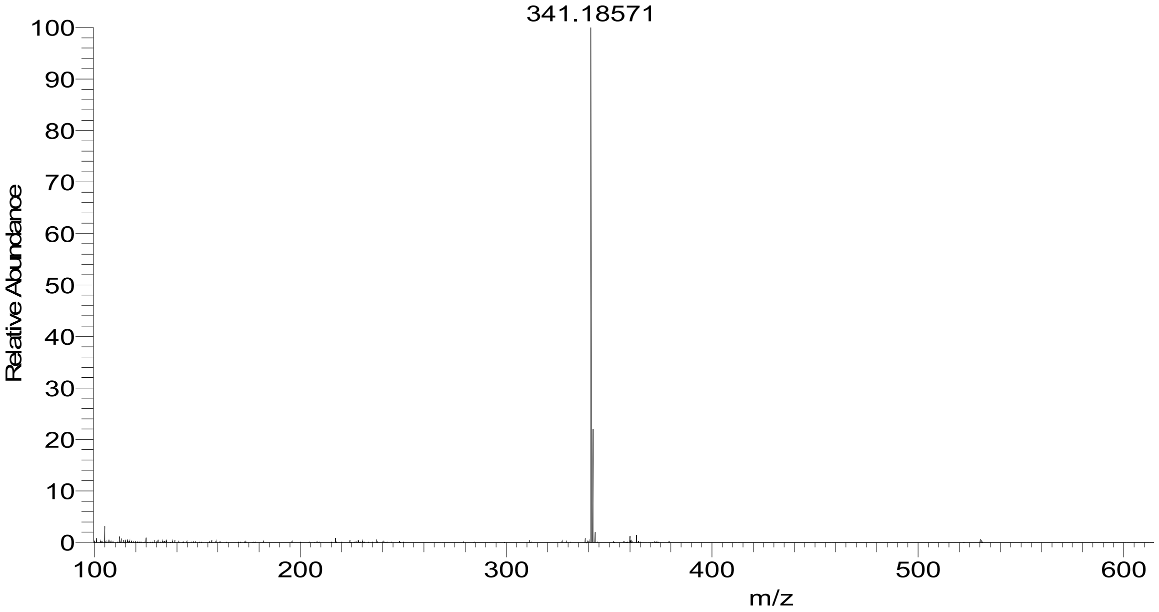

Figure S41. Mass spectrum of compound **PQM273**

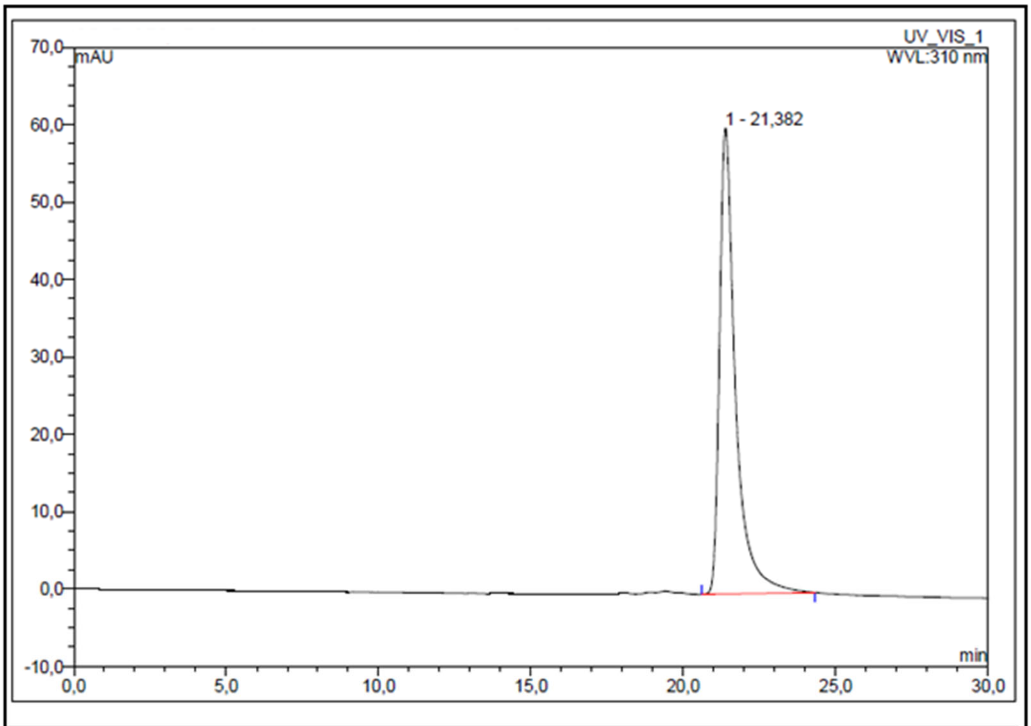

No.	Ret.Time min	Peak Name	Height mAU	Area mAU*min	Rel.Area %	Amount	Type
1	21,38	n.a.	60,202	36,737	100,00	n.a.	BMB
Total:			60,202	36,737	100,00	0,000	

Figure S42. HPLC chromatogram of compound **PQM273**.

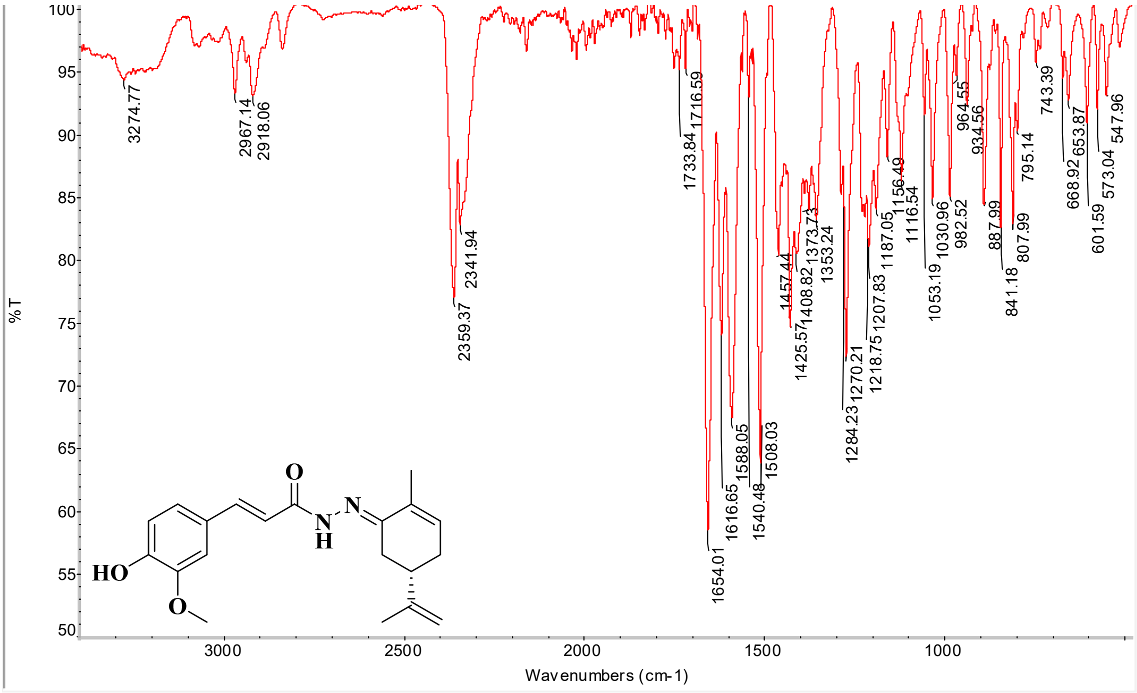

Figure S43. Absorption spectrum in the IR region (ATR) of compound **PQM274**.

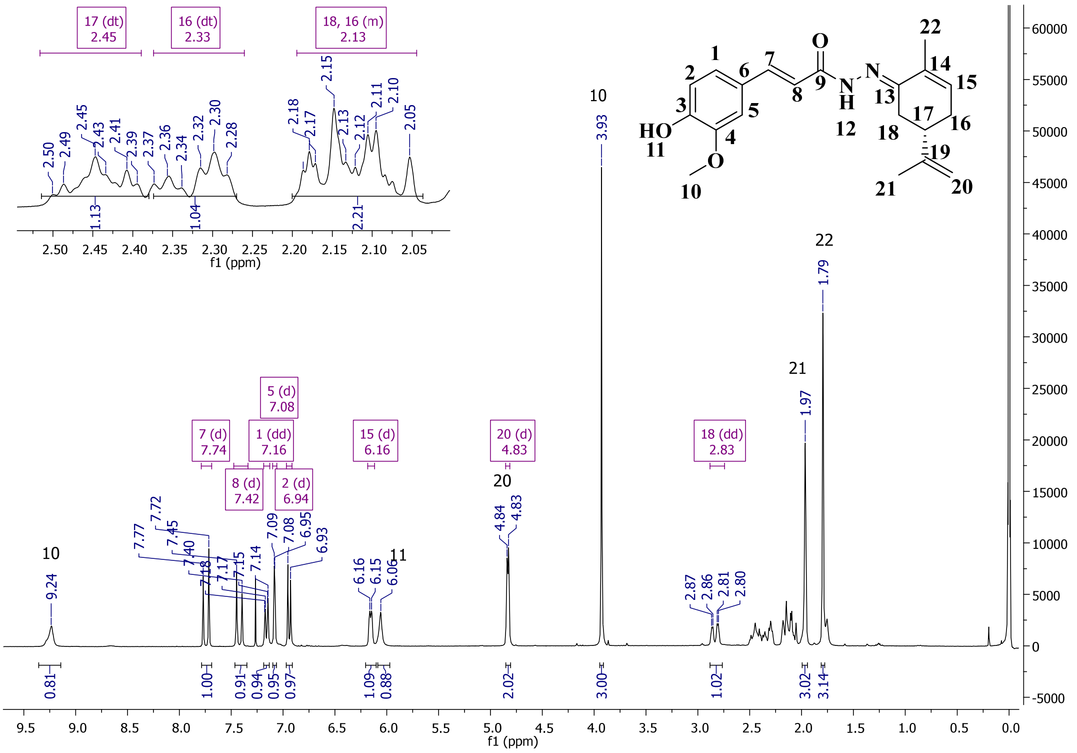

Figure S44. ^{1}H NMR spectrum (300 MHz, $CDCl_3$) of compound **PQM274**.

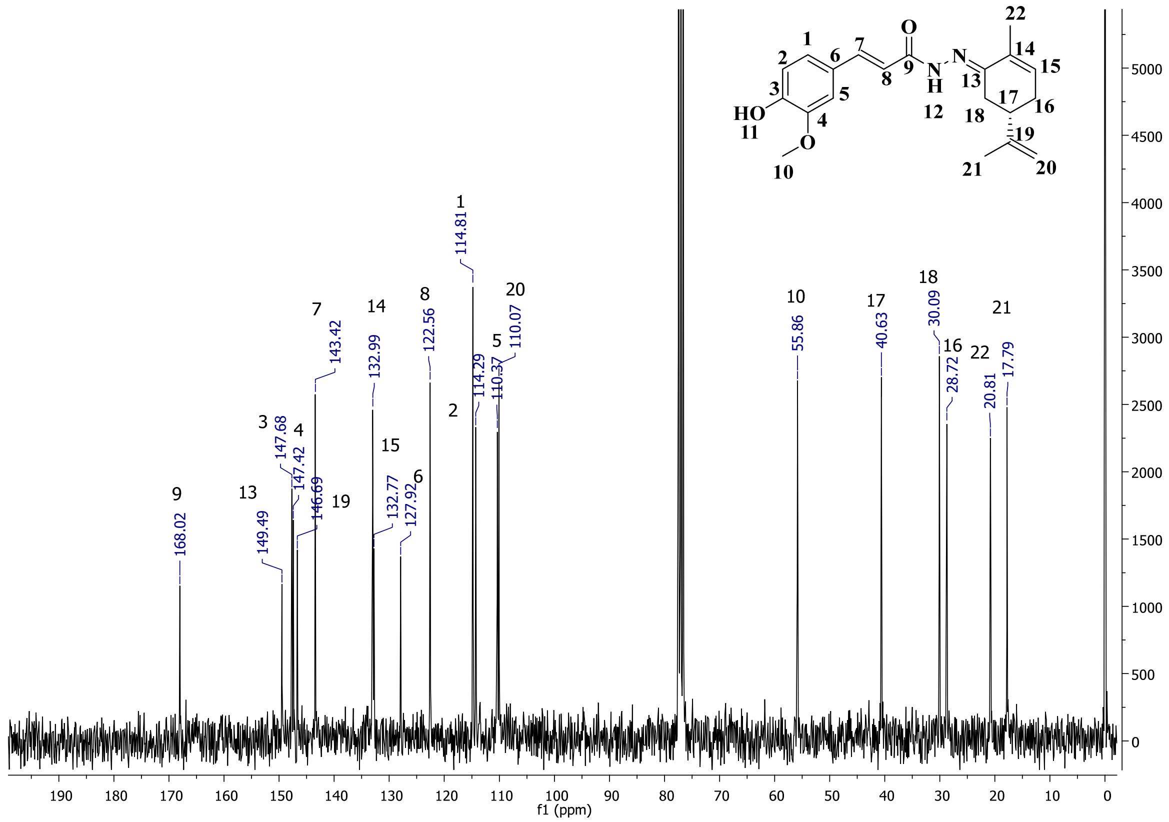

Figure S45. ^{13}C NMR spectrum (75 MHz, $CDCl_3$) of compound **PQM274.**

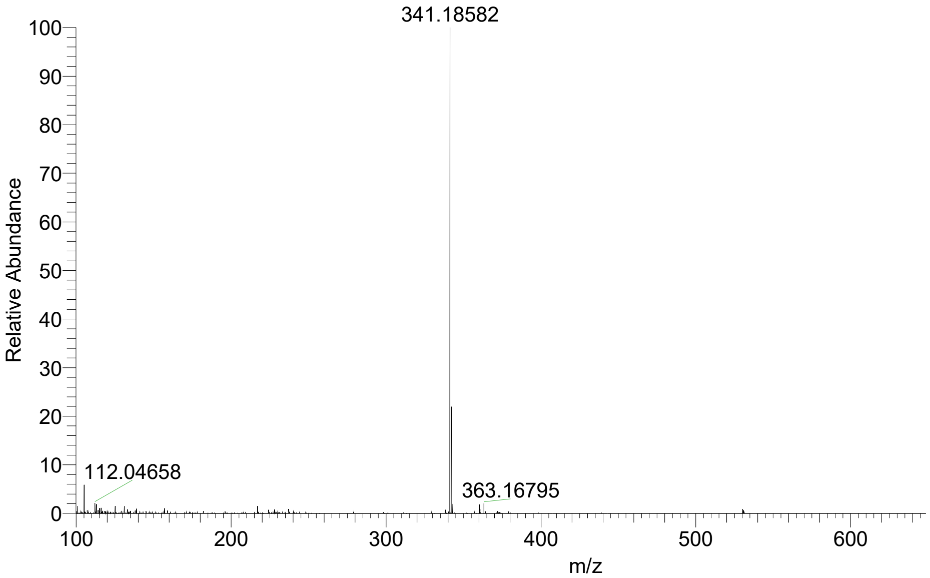

Figure S46. Mass spectrum (ESI-MS) of compound **PQM274**.

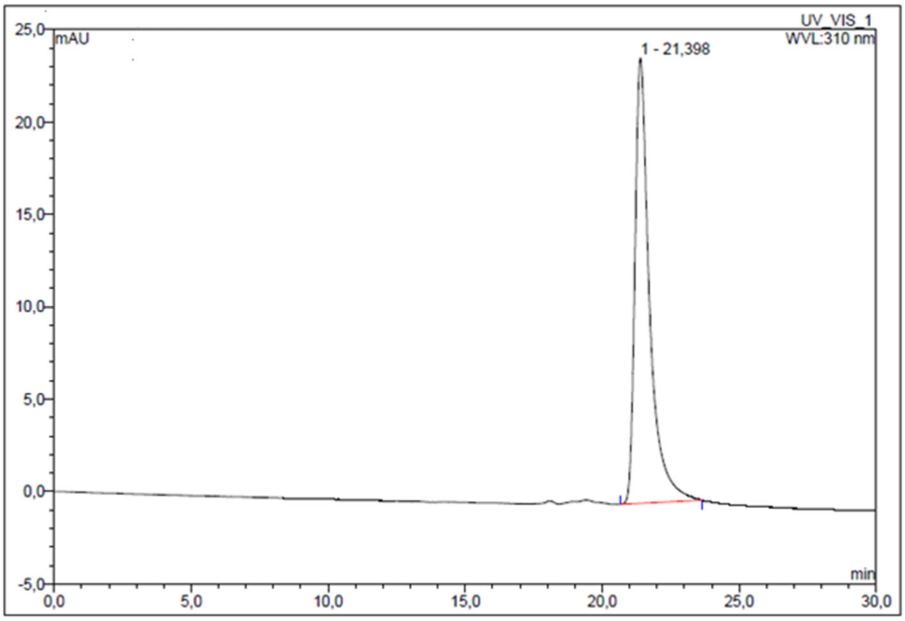

No.	Ret.Time min	Peak Name	Height mAU	Area mAU*min	Rel.Area %	Amount	Type
1	21,40	n.a.	24,115	14,935	100,00	n.a.	BMB
Total:			24,115	14,935	100,00	0,000	

Figure S47. HPLC chromatogram of compound **PQM274**.

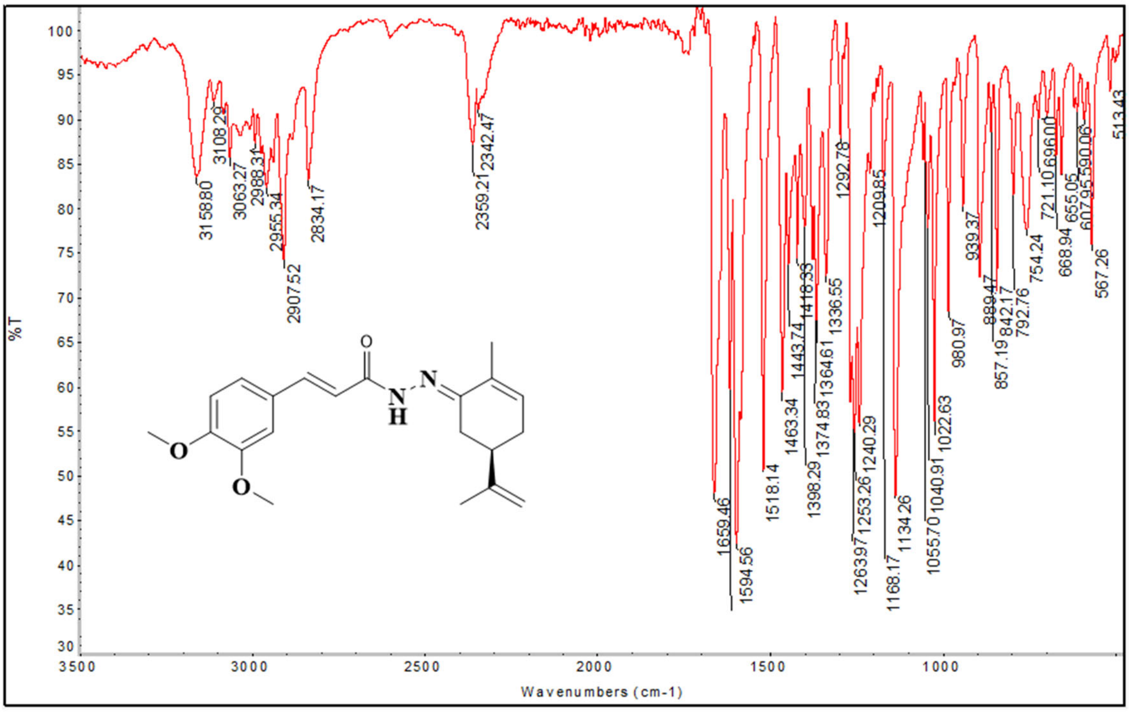

Figure S48. Absorption spectrum in the IR region (ATR) of the compound **PQM275**.

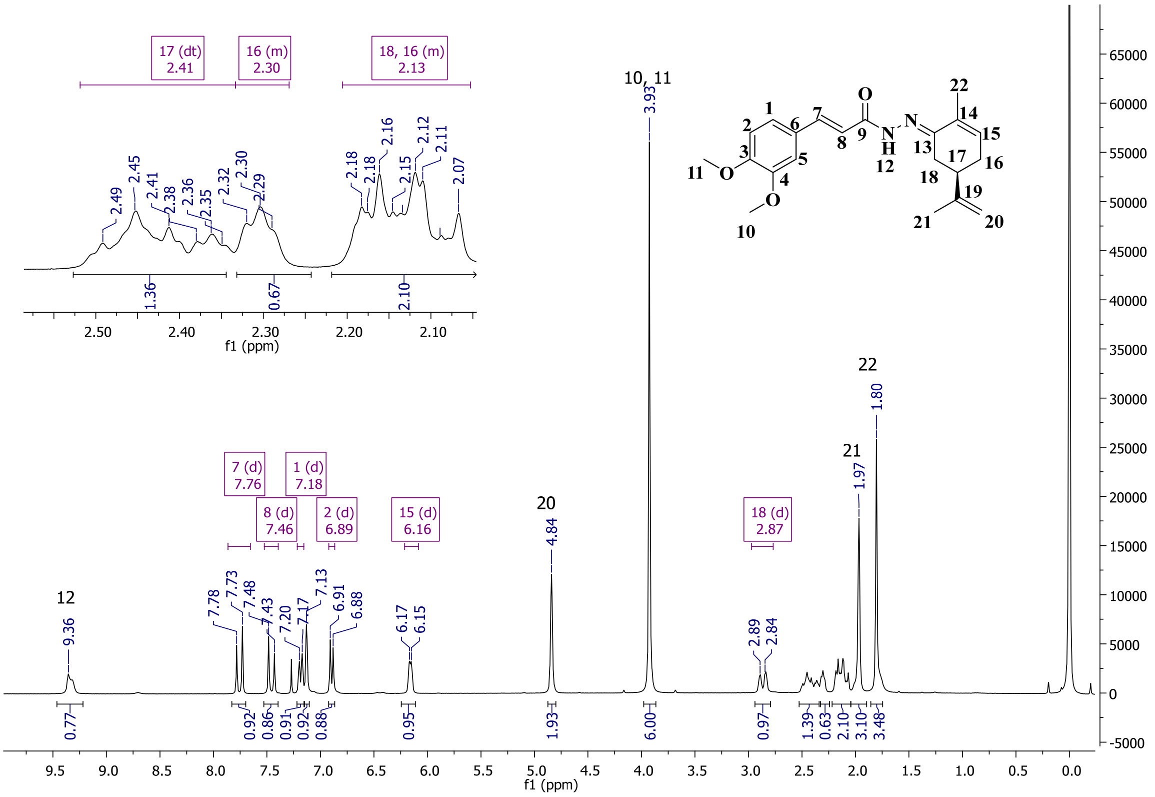

Figure S49. ^{1}H NMR spectrum (300 MHz, $CDCl_3$) of compound **PQM275**.

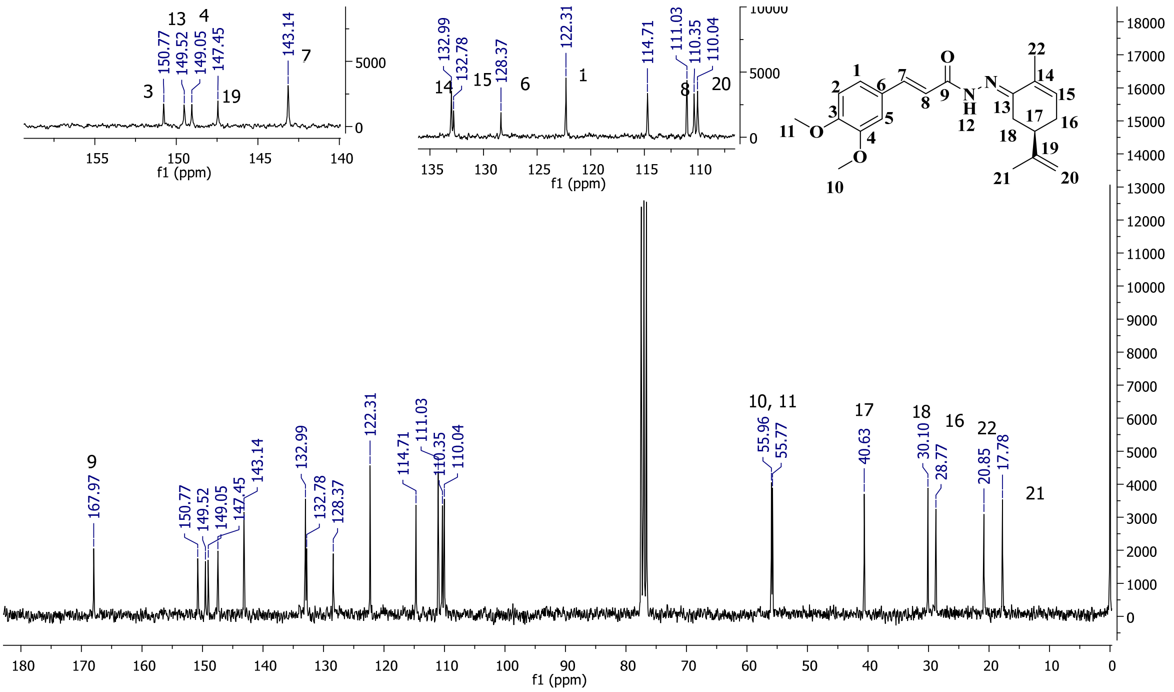

Figure S50. ^{13}C NMR spectrum (75 MHz, $CDCl_3$) of compound **PQM275**.

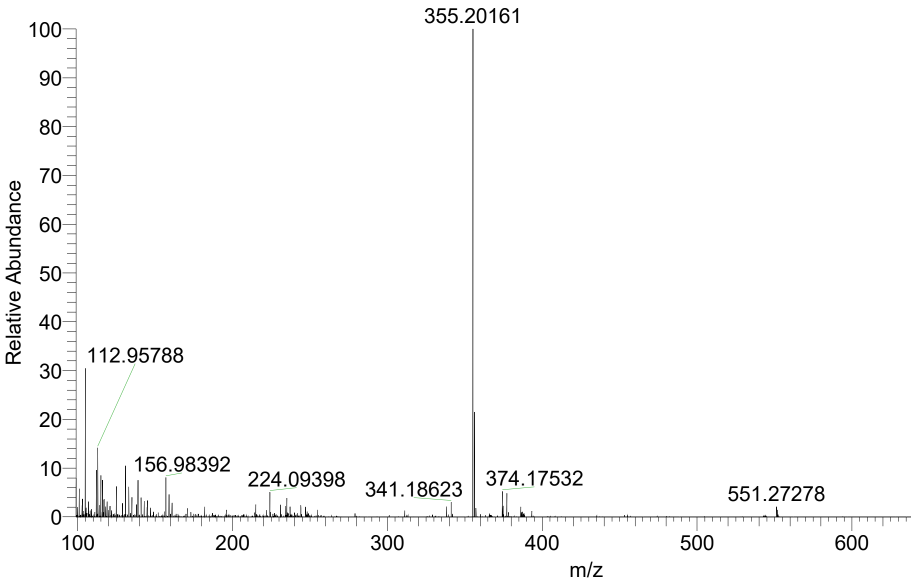

Figure S51. Mass spectrum (ESI-MS) of compound **PQM275**.

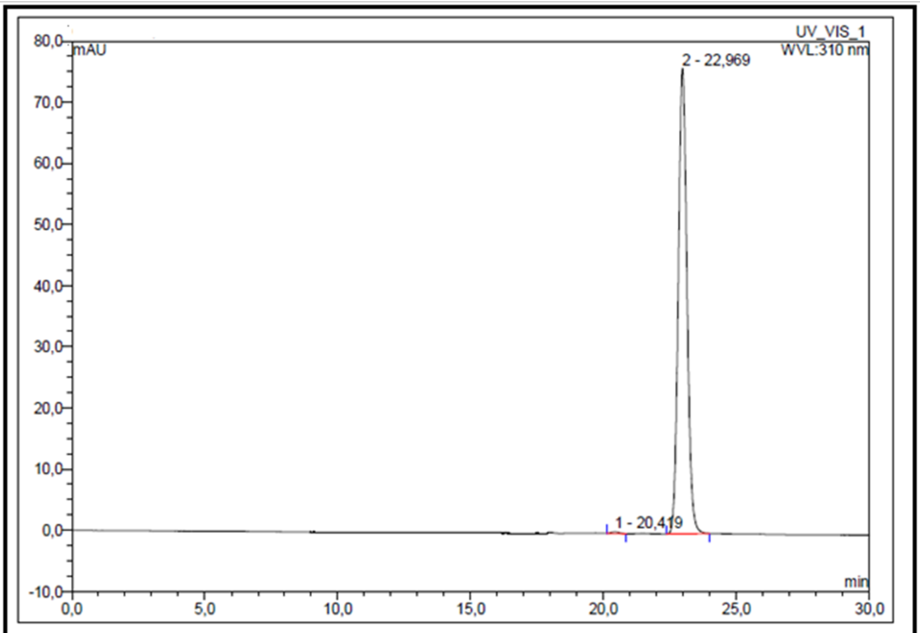

No.	Ret.Time min	Peak Name	Height mAU	Area mAU*min	Rel.Area %	Amount	Type
1	20,42	n.a.	0,253	0,081	0,28	n.a.	BMB
2	22,97	n.a.	76,114	28,930	99,72	n.a.	BMB
Total:			76,368	29,011	100,00	0,000	

Figure S52. HPLC chromatogram of compound **PQM275**.

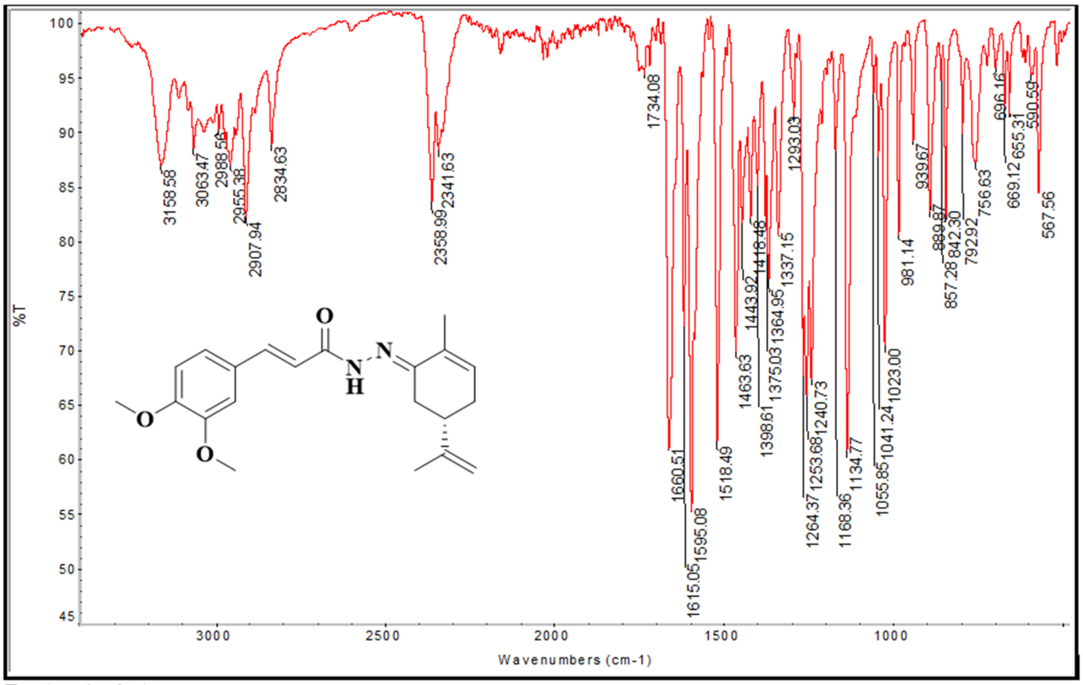

Figure S53. Absorption spectrum in the IR region (ATR) of the compound **PQM276**.

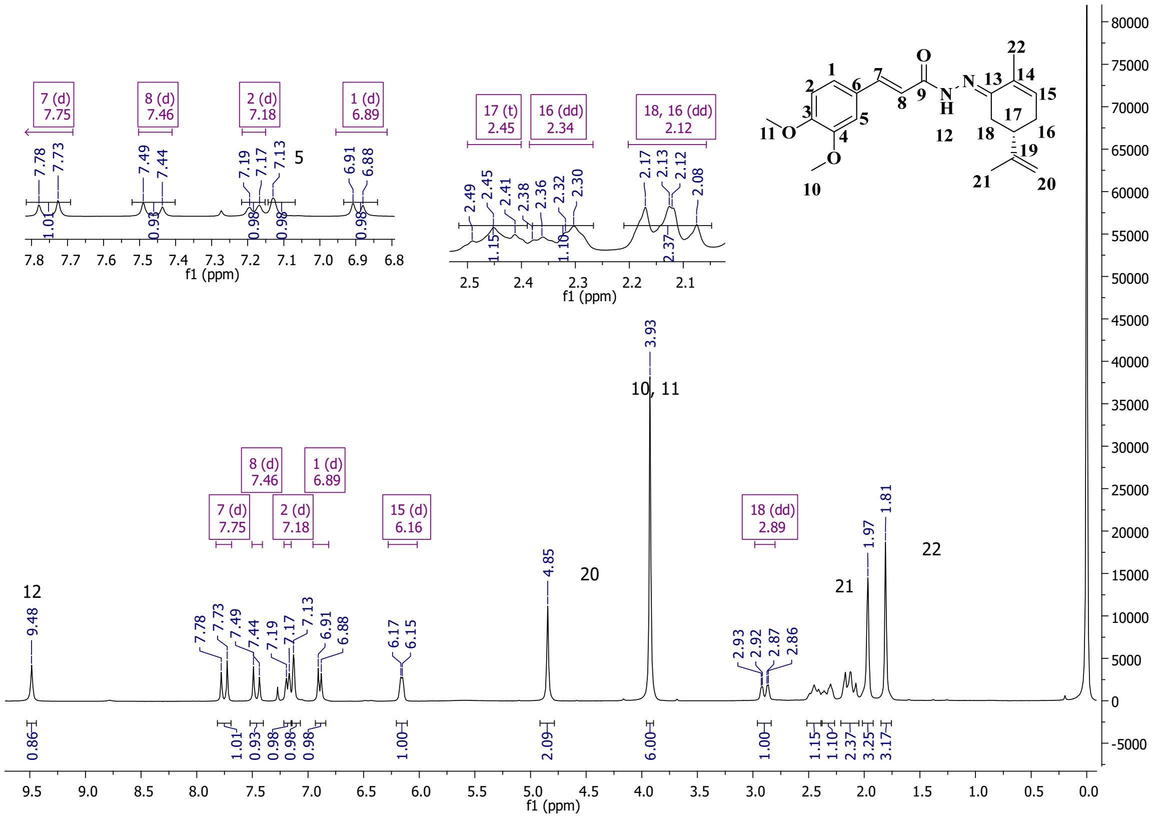

Figure S54. ^{1}H NMR spectrum (300 MHz, $CDCl_3$) of compound **PQM276**.

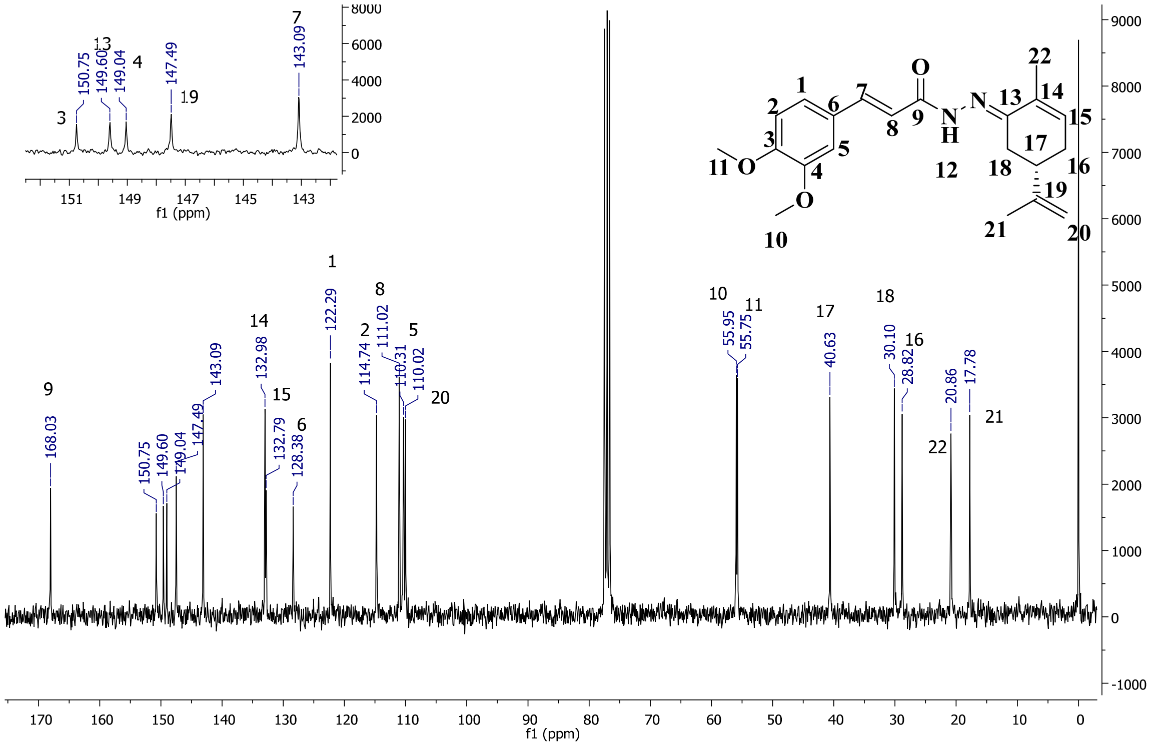

Figure S55. ^{13}C NMR spectrum (75 MHz, $CDCl_3$) of compound **PQM276**.

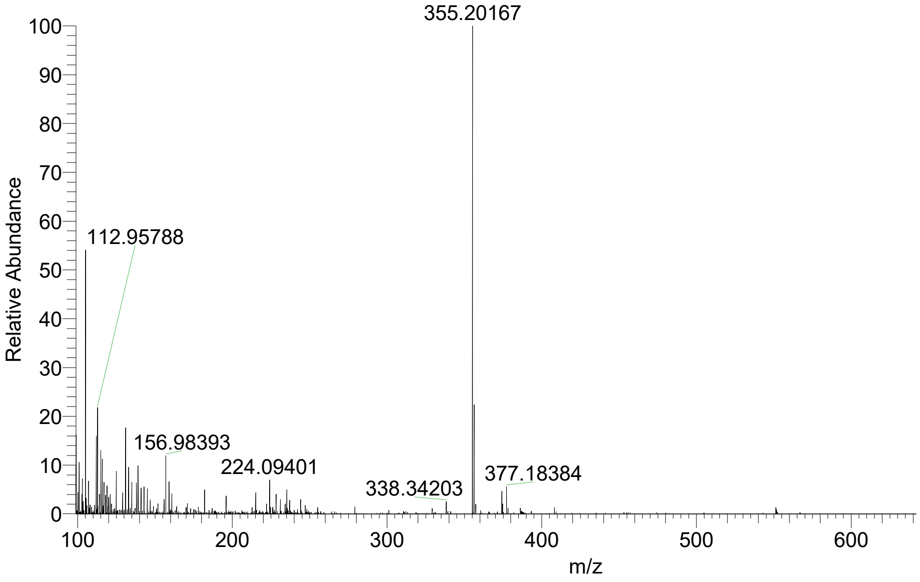

Figure S56. Mass spectrum (ESI-MS) of compound **PQM276**.

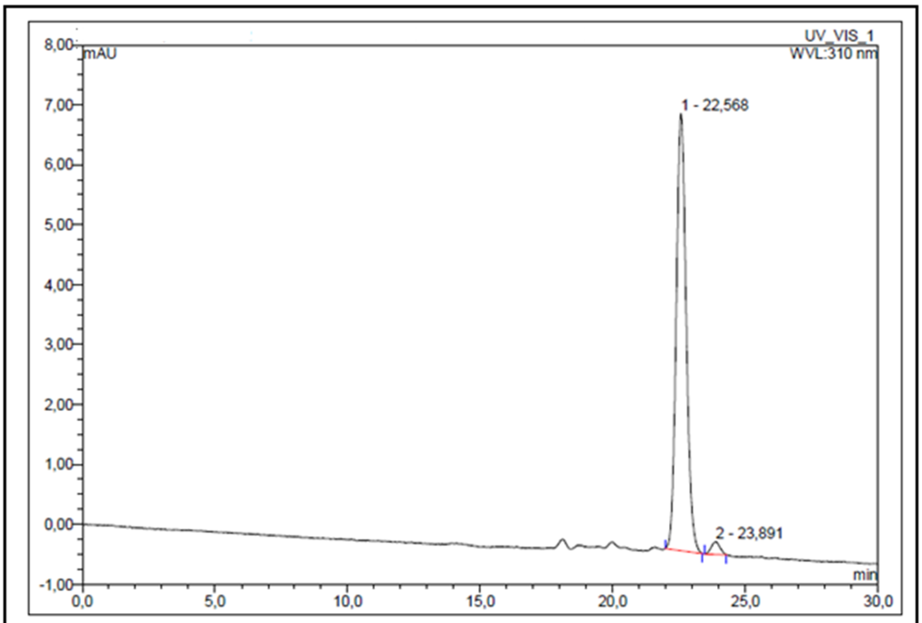

No.	Ret.Time min	Peak Name	Height mAU	Area mAU*min	Rel.Area %	Amount	Type
1	22,57	n.a.	7,294	3,164	97,44	n.a.	BMB
2	23,89	n.a.	0,218	0,083	2,56	n.a.	BMB
Total:			7,512	3,247	100,00	0,000	

Figure S57. HPLC chromatogram of compound **PQM276.**

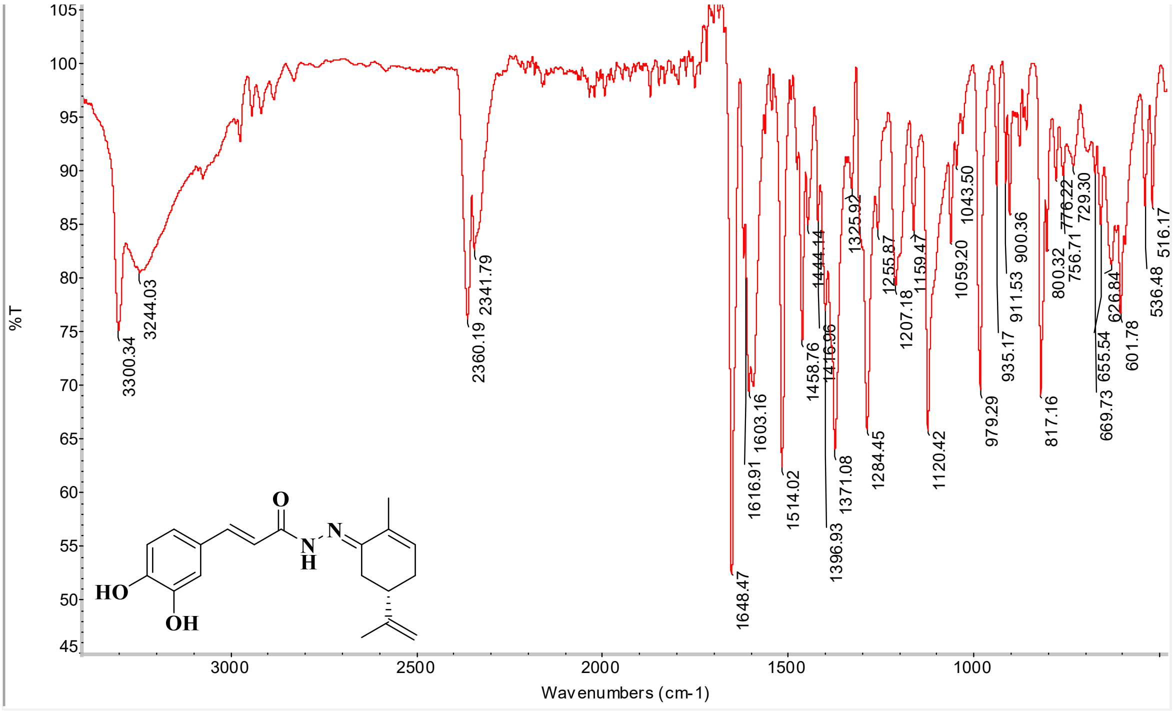

Figure S58. Absorption spectrum in the IR region (ATR) of compound **PQM290**.

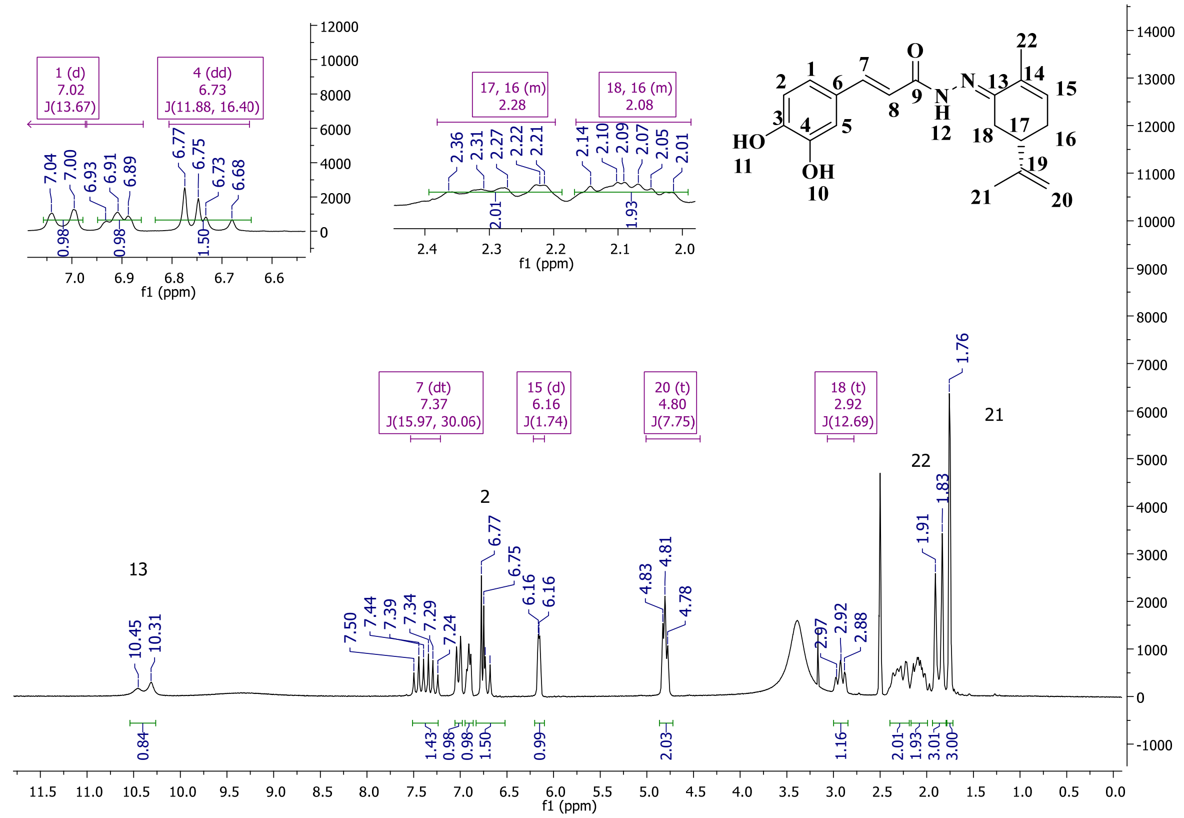

Figure S59. ^{1}H NMR spectrum (300 MHz, DMSO-d_6) of compound **PQM290.**

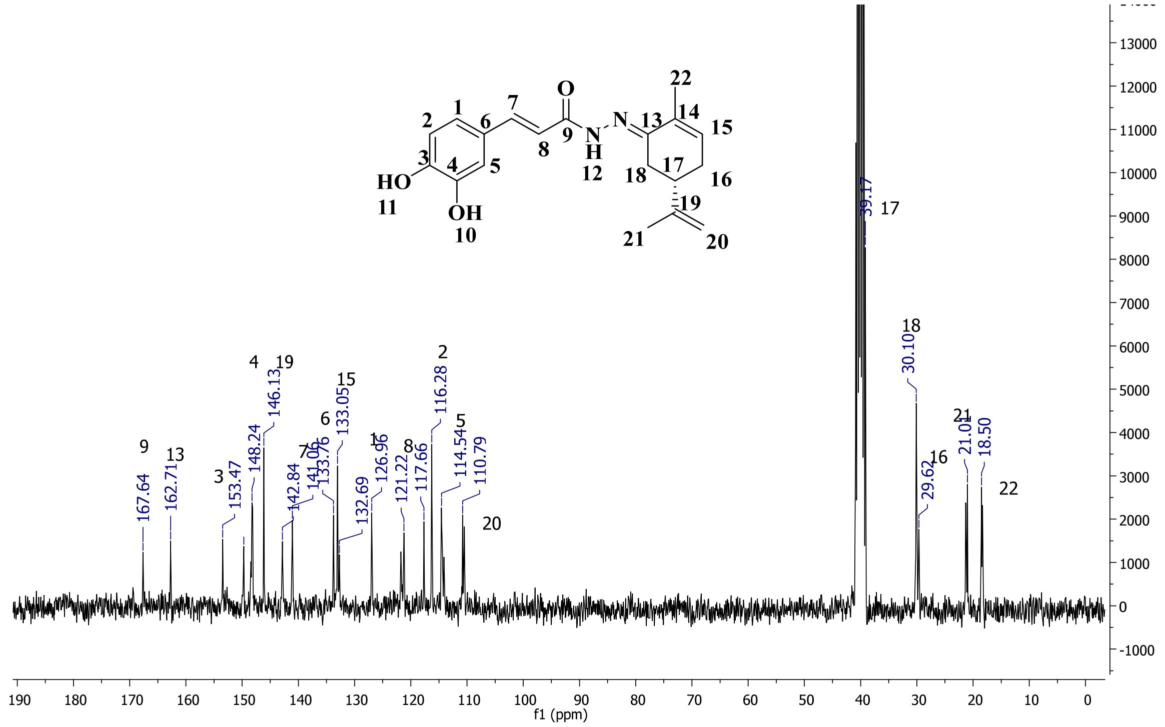

Figure S60. ^{13}C NMR spectrum (75 MHz, DMSO-d_6) of compound **PQM290**.

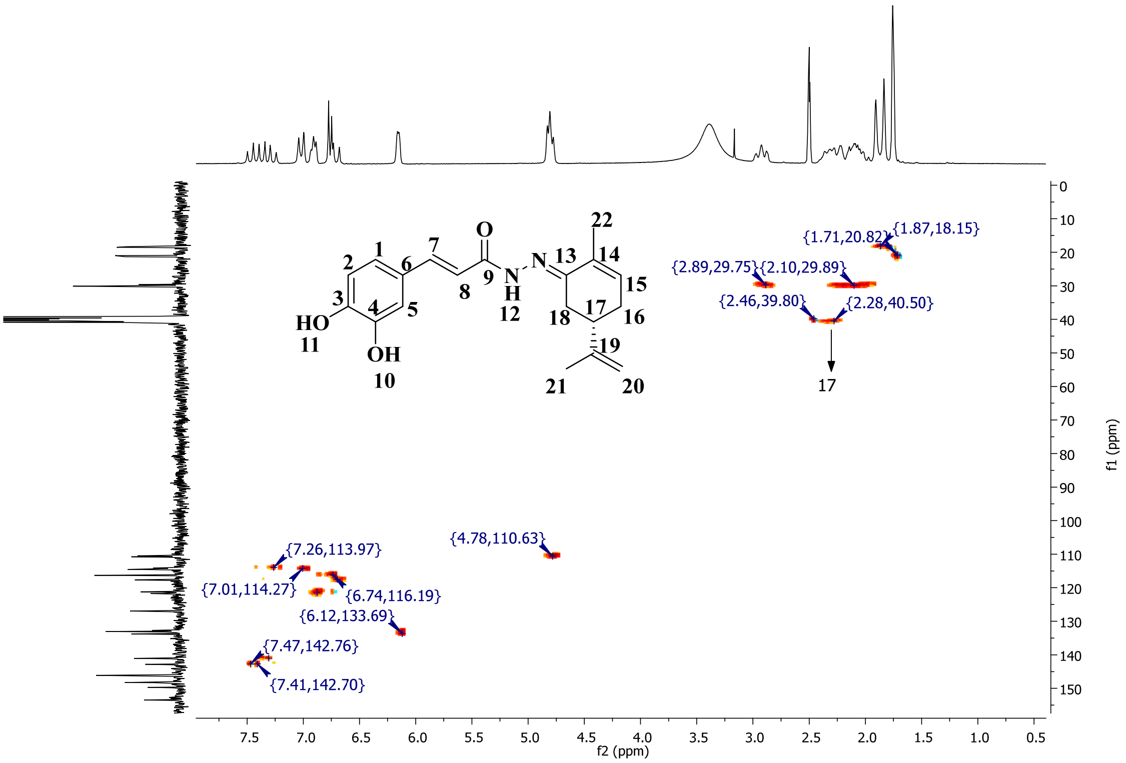

Figure S61. HSQC correlation map of compound **PQM290**.

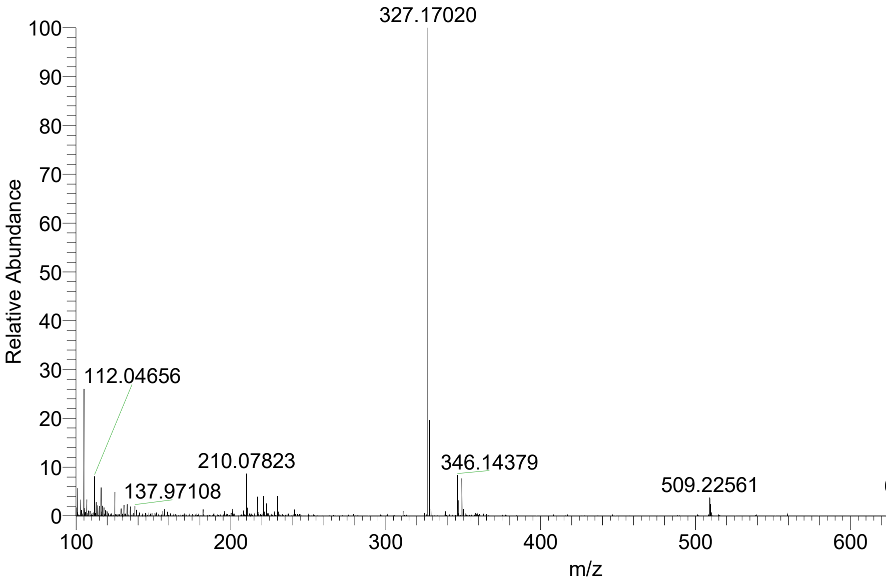

Figure S62. Mass spectrum (ESI-MS) of compound **PQM290**.

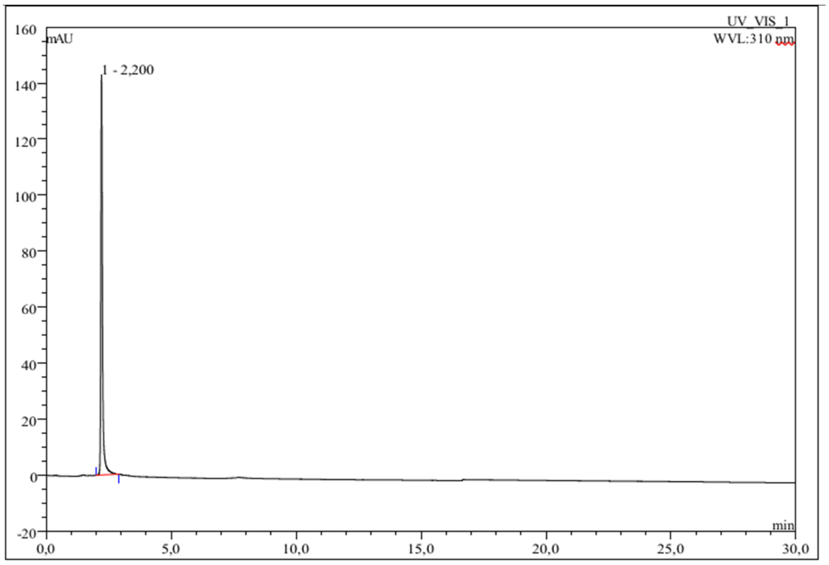

No.	Ret.Time min	Peak Name	Height mAU	Area mAU*min	Rel.Area %	Amount	Type
1	2,20	n.a.	142,980	10,936	100,00	n.a.	BMB
Total:			142,980	10,936	100,00	0,000	

Figure S63. HPLC chromatogram of compound **PQM290**

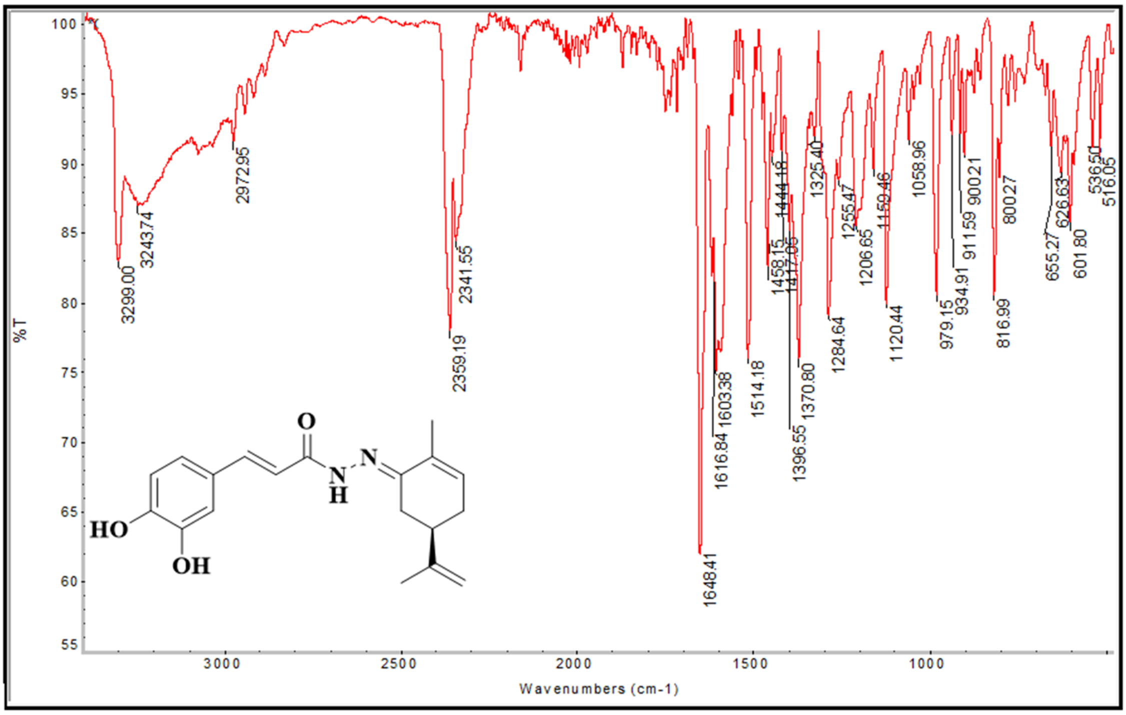

Figure S64. Absorption spectrum in the IR region (ATR) of compound **PQM291**.

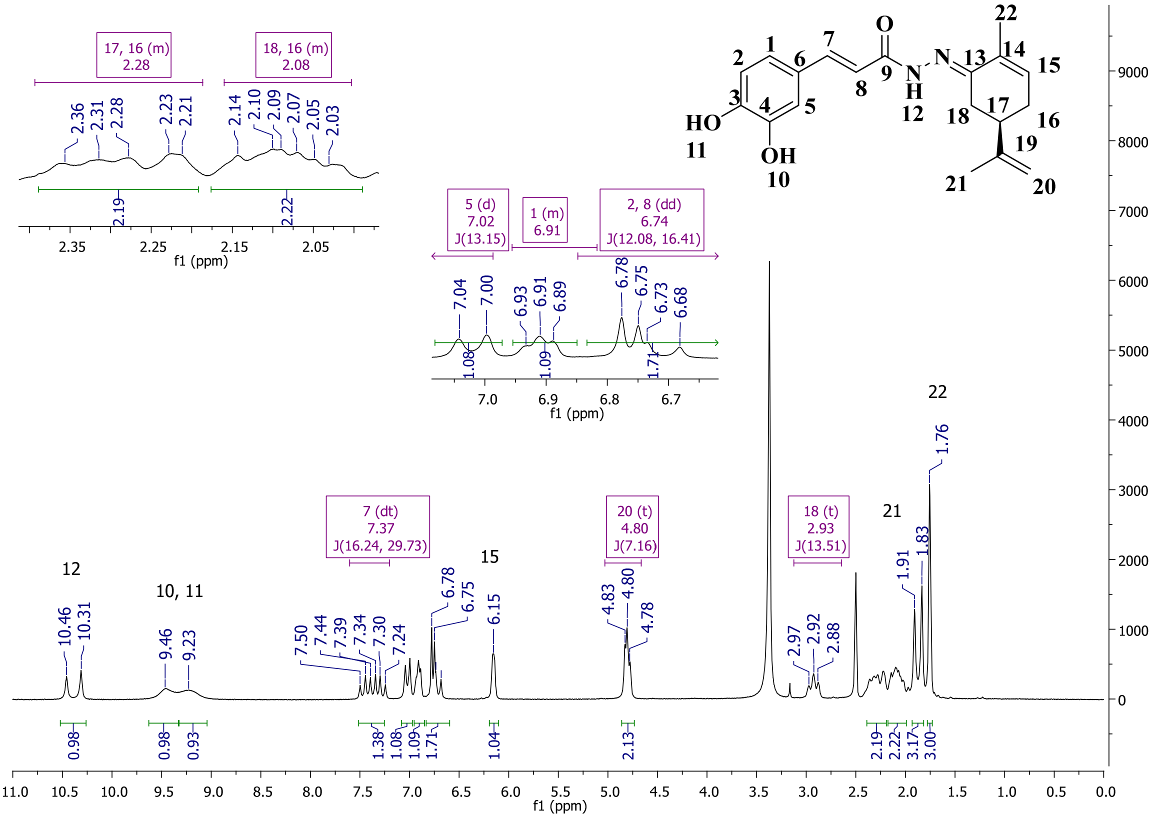

Figure S65. ^{1}H NMR spectrum (300 MHz, DMSO-d_6) of compound **PQM291**.

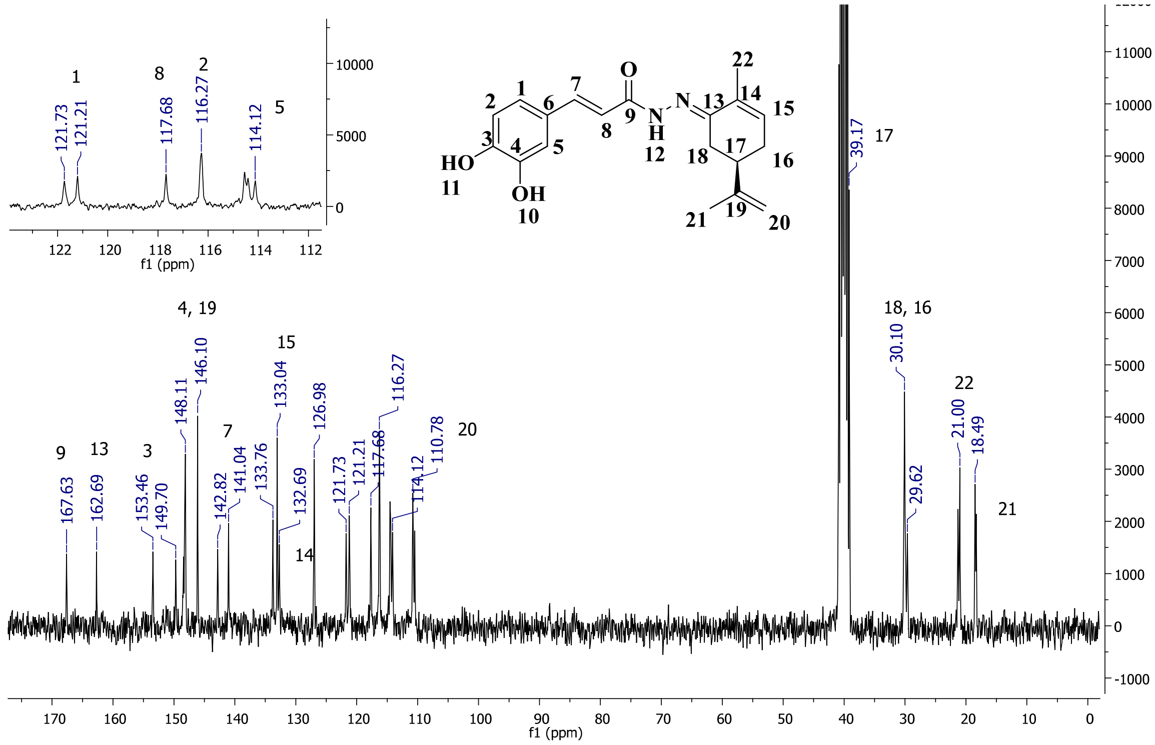

Figure S66. ^{13}C NMR spectrum (75 MHz, DMSO-d_6) of compound **PQM291**.

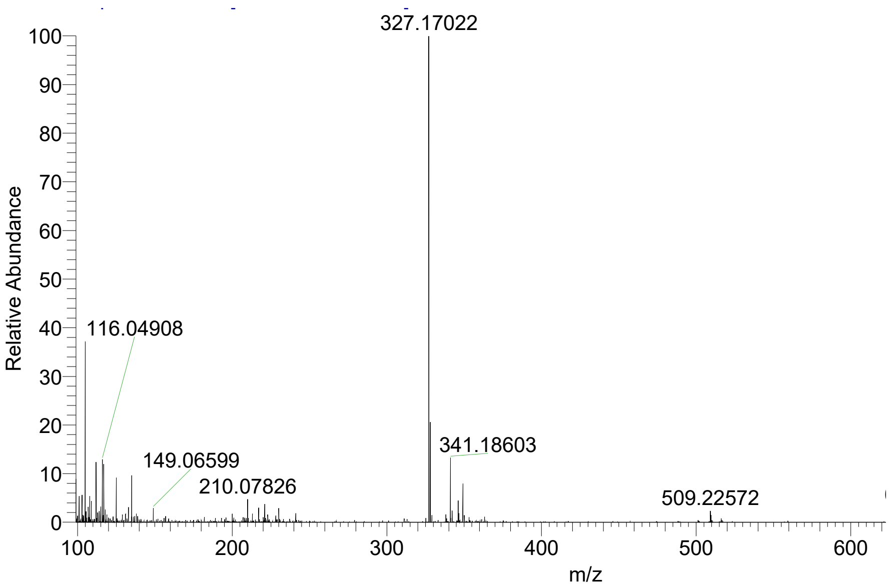

Figure S66. Mass spectrum (ESI-MS) of compound **PQM291**.

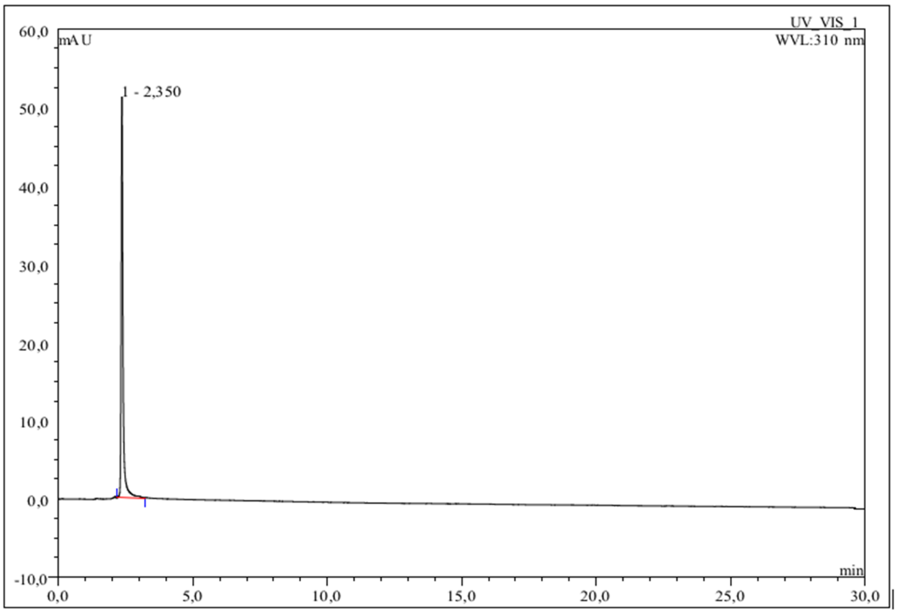

No.	Ret.Time min	Peak Name	Height mAU	Area mAU*min	Rel.Area %	Amount	Type
1	2,35	n.a.	51,110	4,224	100,00	n.a.	BMB
Total:			51,110	4,224	100,00	0,000	

Figure S67. HPLC chromatogram of compound **PQM291**.

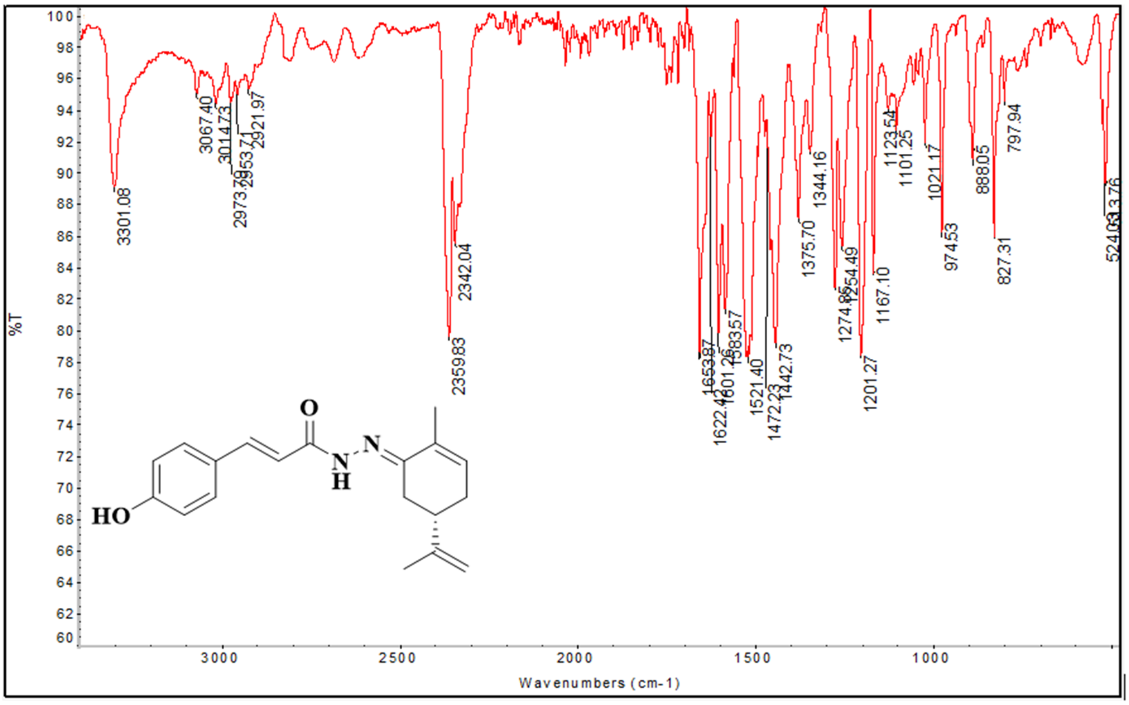

Figure S68. Absorption spectrum in the IR region (ATR) of compound **PQM292**.

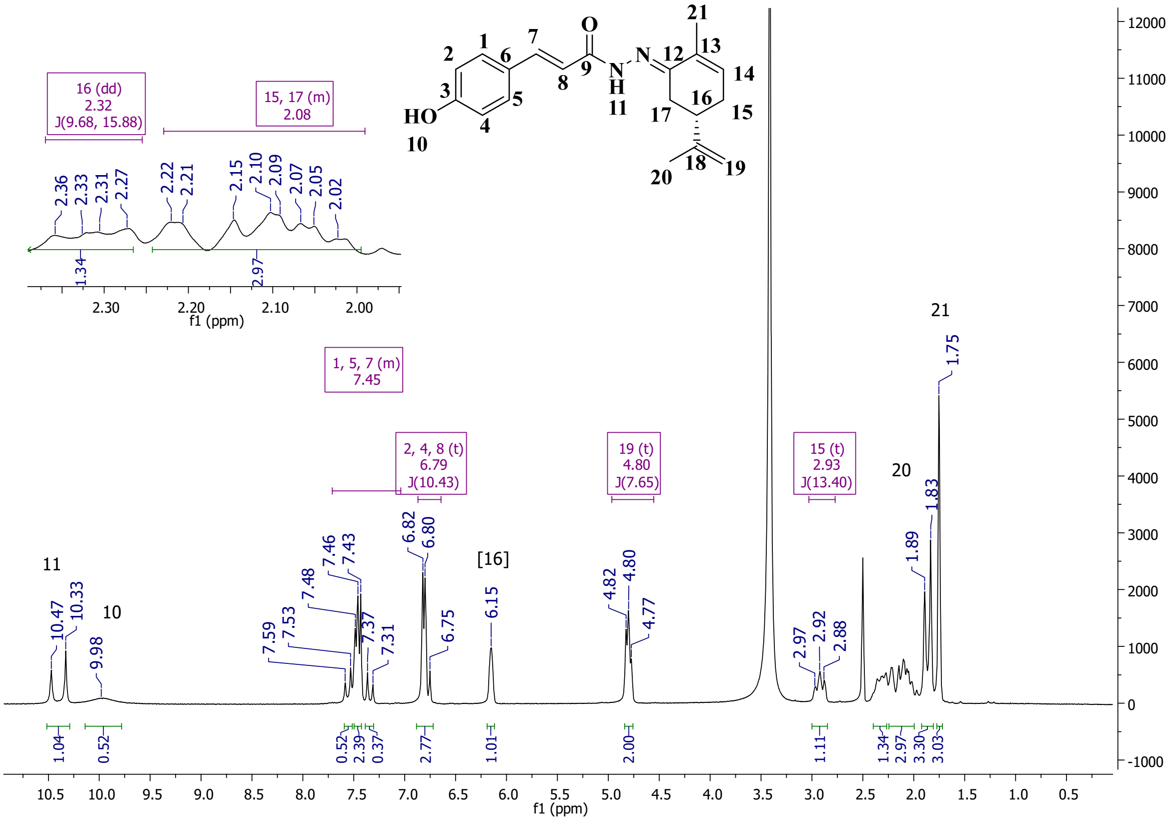

Figure S69. ^{1}H NMR spectrum (300 MHz, DMSO-d_6) of compound **PQM292**.

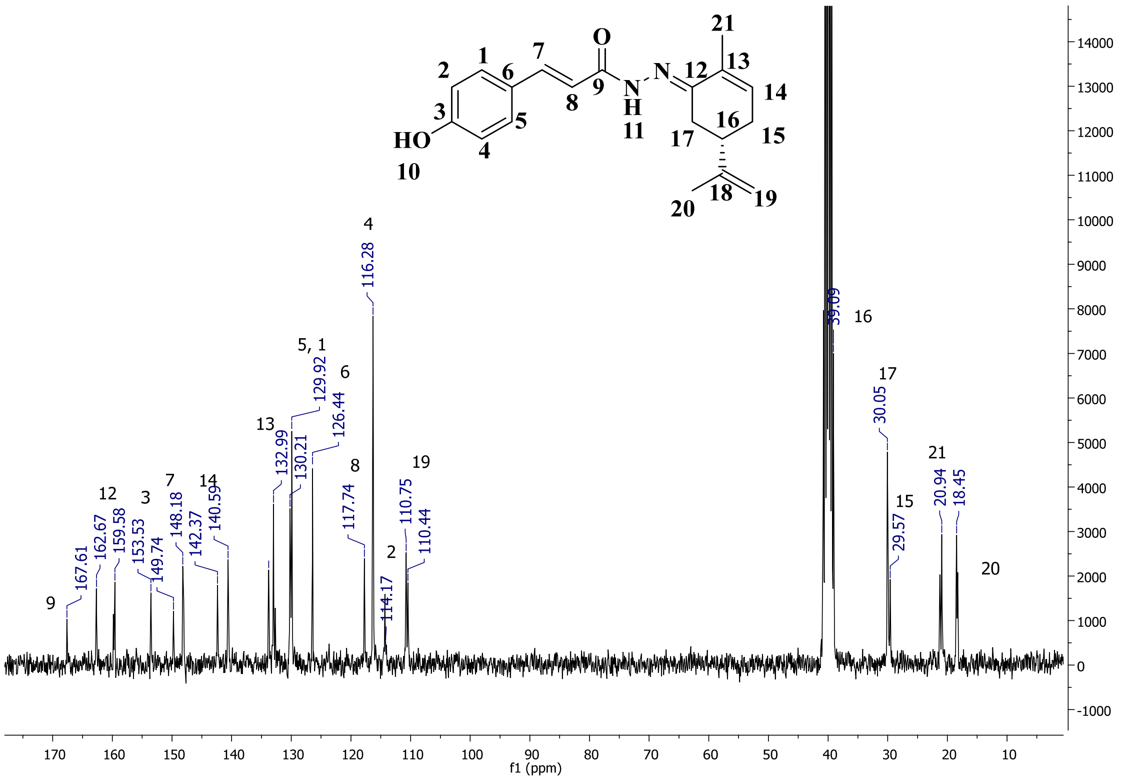

Figure S70. ^{13}C NMR spectrum (75 MHz, DMSO-d_6) of compound **PQM292**.

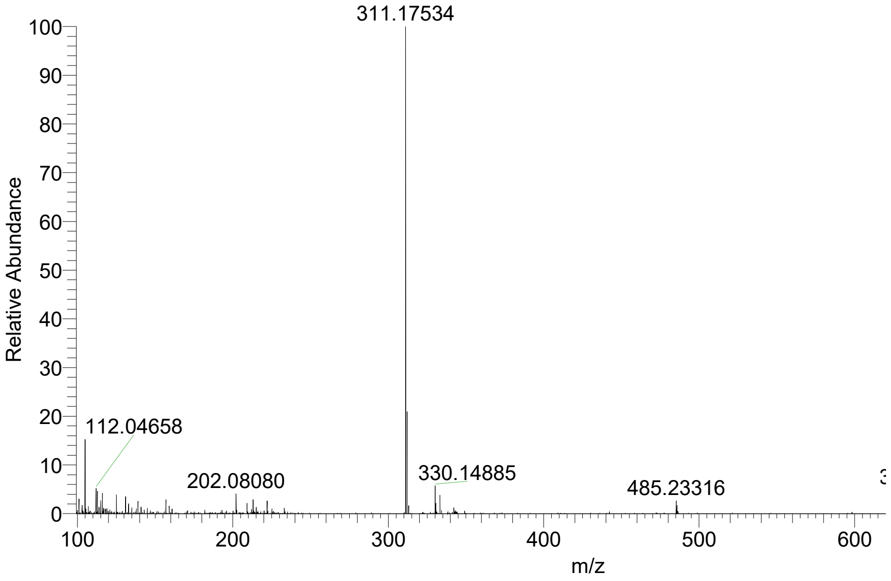

Figure S71. Mass spectrum (ESI-MS) of compound **PQM292**.

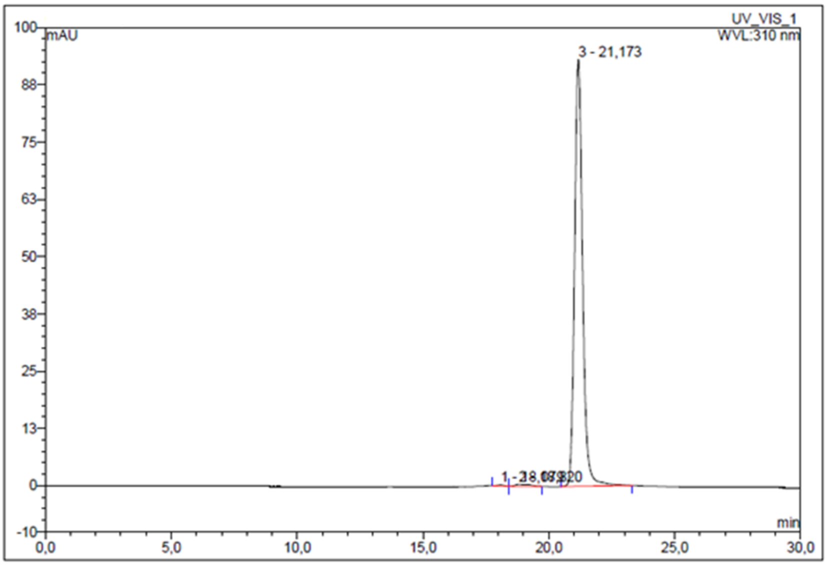

No.	Ret.Time min	Peak Name	Height mAU	Area mAU*min	Rel.Area %	Amount	Type
1	18,08	n.a.	0,242	0,070	0,20	n.a.	BMB
2	18,82	n.a.	0,461	0,315	0,90	n.a.	BMB
3	21,17	n.a.	93,103	34,490	98,90	n.a.	BMB
Total:			93,806	34,876	100,00	0,000	

Figure S72. HPLC chromatogram of compound **PQM292**.

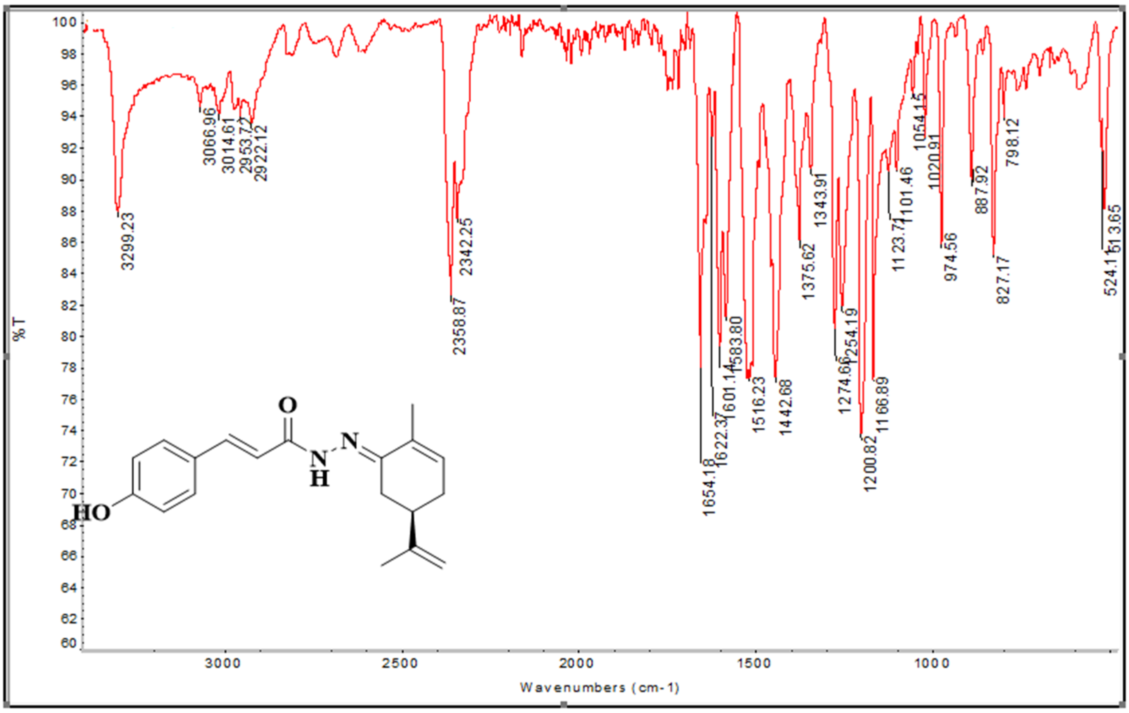

Figure S73. Absorption spectrum in the IR region (ATR) of compound **PQM293**.

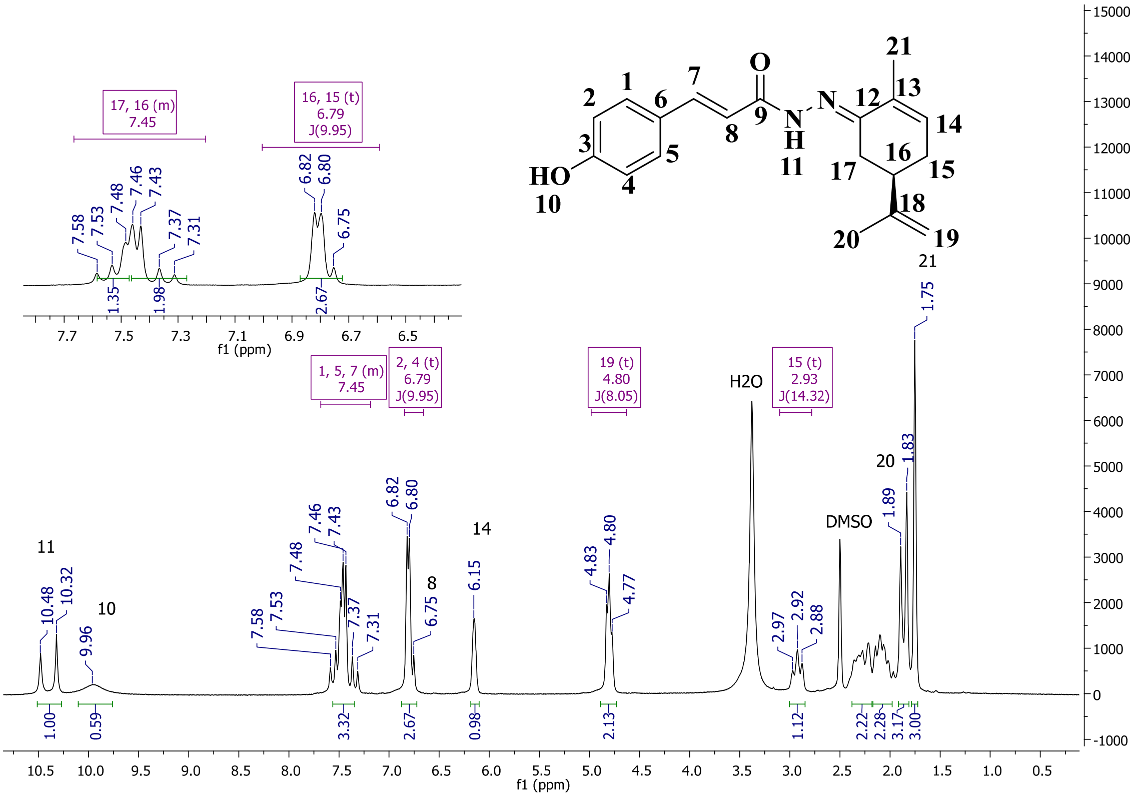

Figure S74. ^{1}H NMR spectrum (300 MHz, DMSO-d_6) of compound **PQM293**.

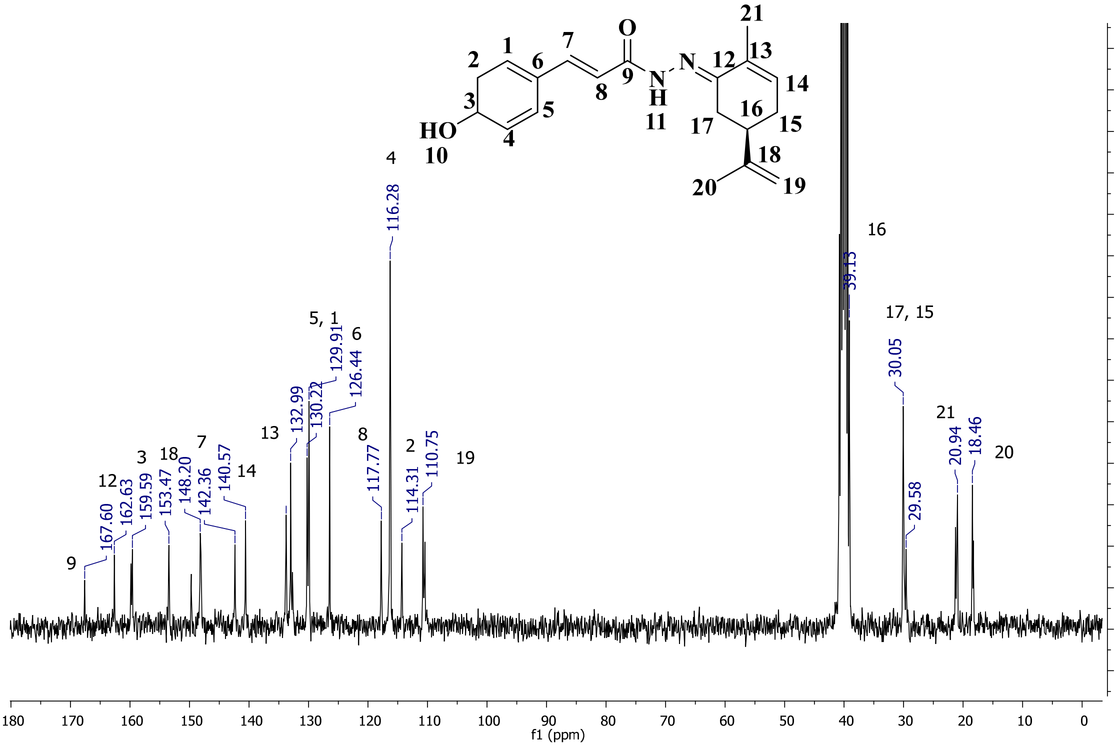

Figure S75. ^{13}C NMR spectrum (75 MHz, DMSO-d_6) of compound **PQM293**.

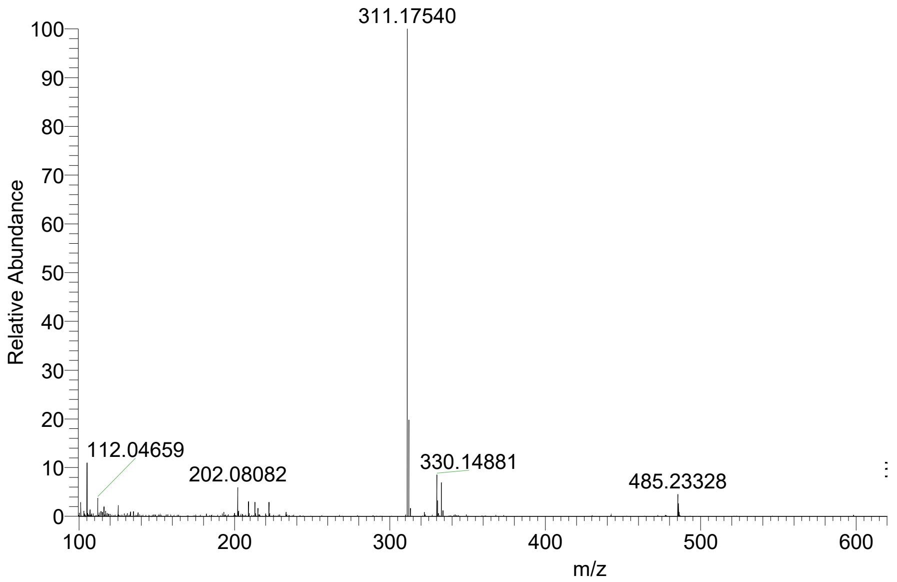

Figure S76. Mass spectrum (ESI-MS) of compound **PQM293**.

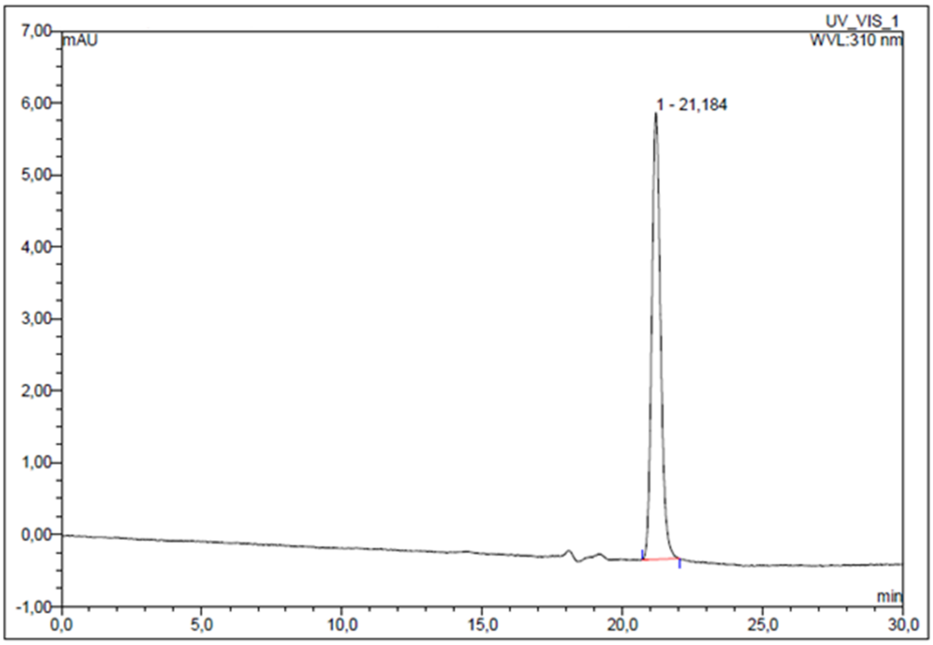

No.	Ret.Time min	Peak Name	Height mAU	Area mAU*min	Rel.Area %	Amount	Type
1	21,18	n.a.	6,205	2,261	100,00	n.a.	BMB
Total:			6,205	2,261	100,00	0,000	

Figure S77. HPLC chromatogram of compound **PQM293**.

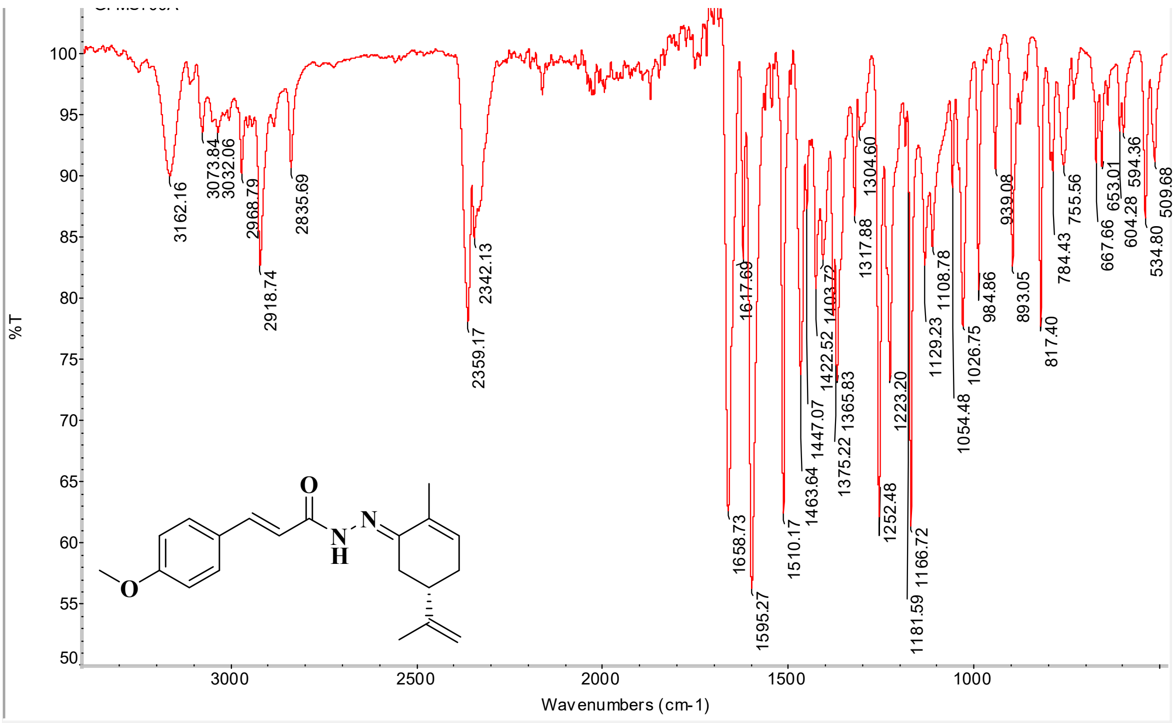

Figure S78. Absorption spectrum in the IR region (ATR) of compound **PQM294**.

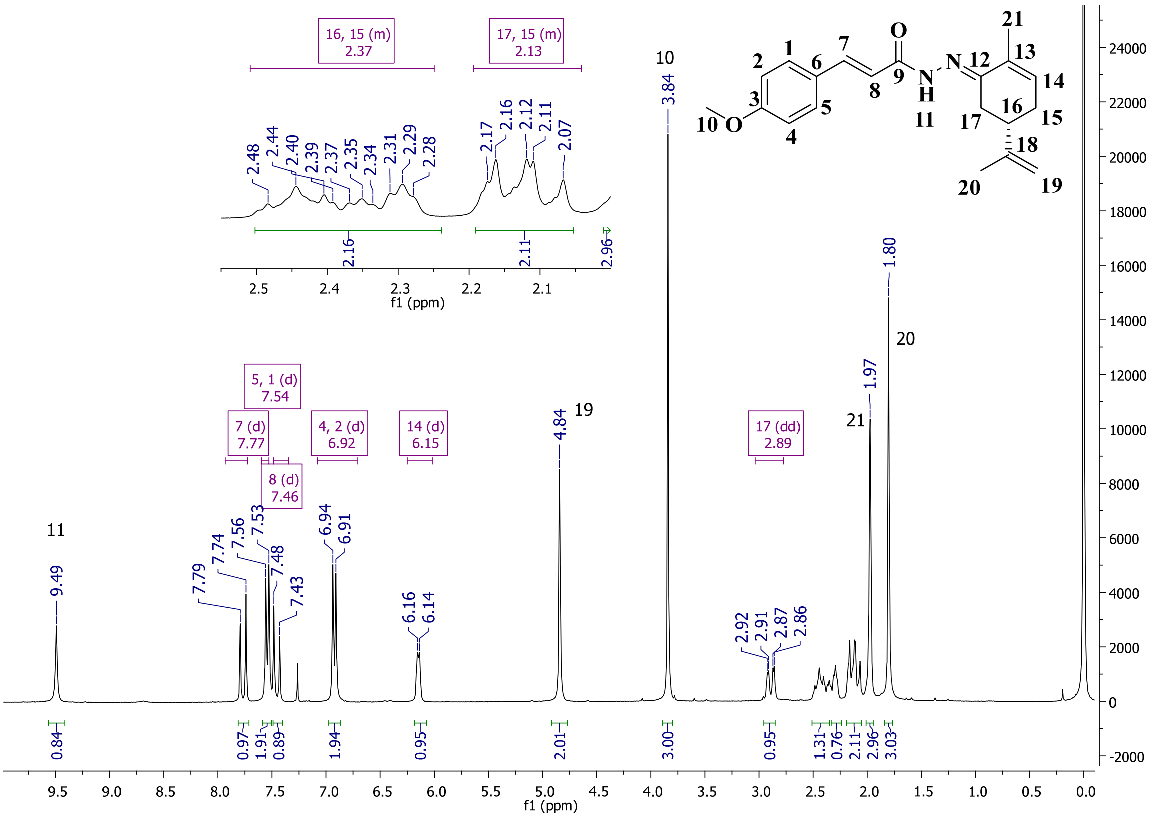

Figure S79. ^{1}H NMR spectrum (300 MHz, CDCl3) of compound **PQM294**.

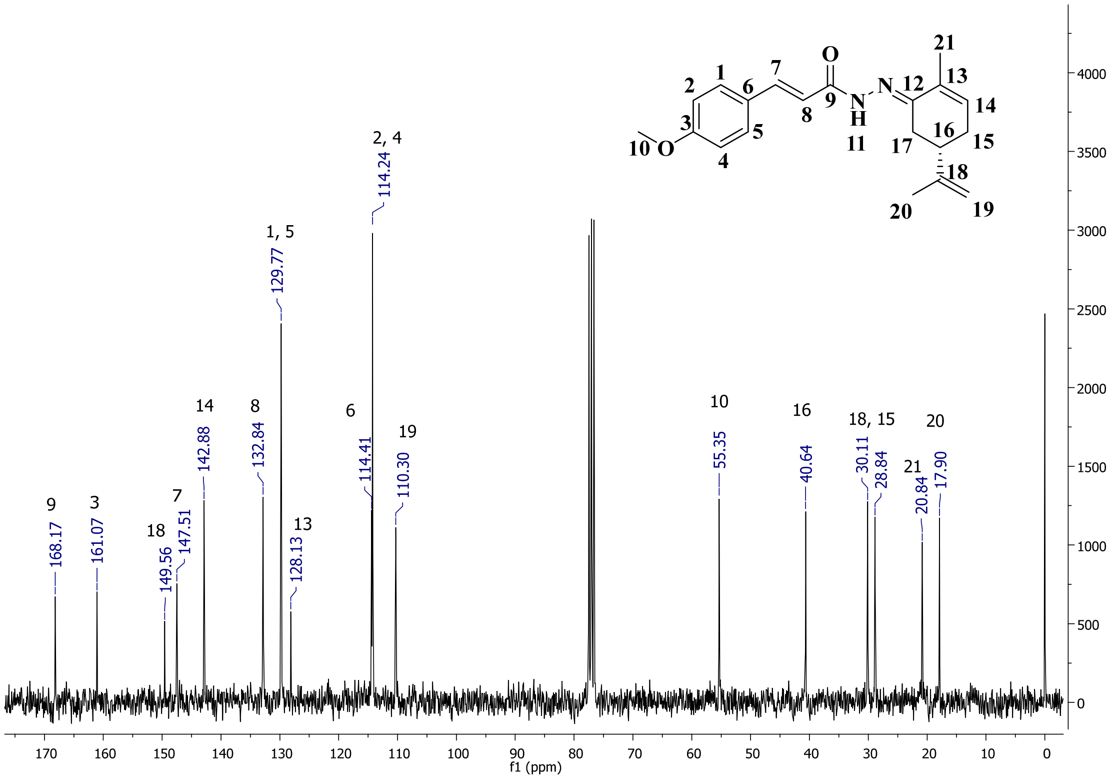

Figure S80. ^{13}C NMR spectrum (75 MHz, $CDCl_3$) of compound **PQM294**.

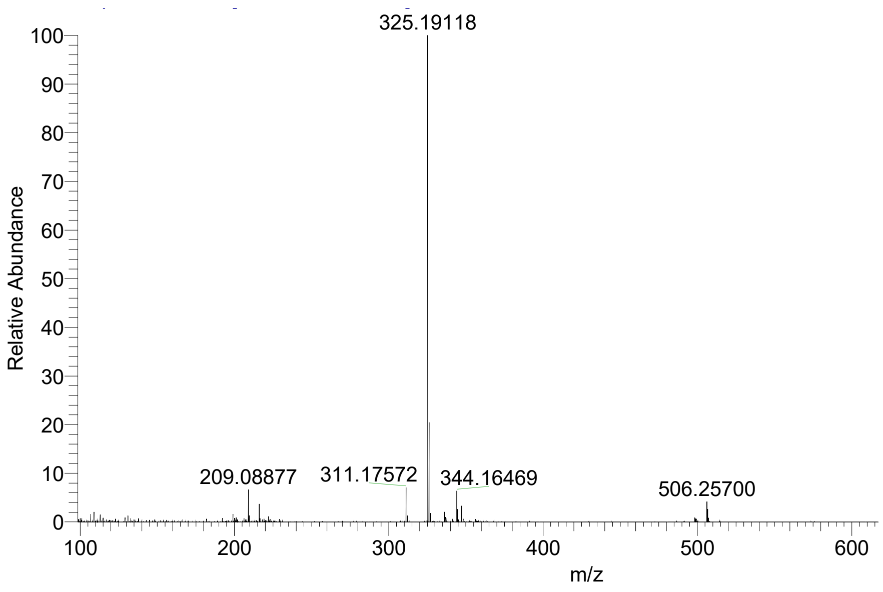

Figure S81. Mass spectrum (ESI-MS) of compound **PQM294**.

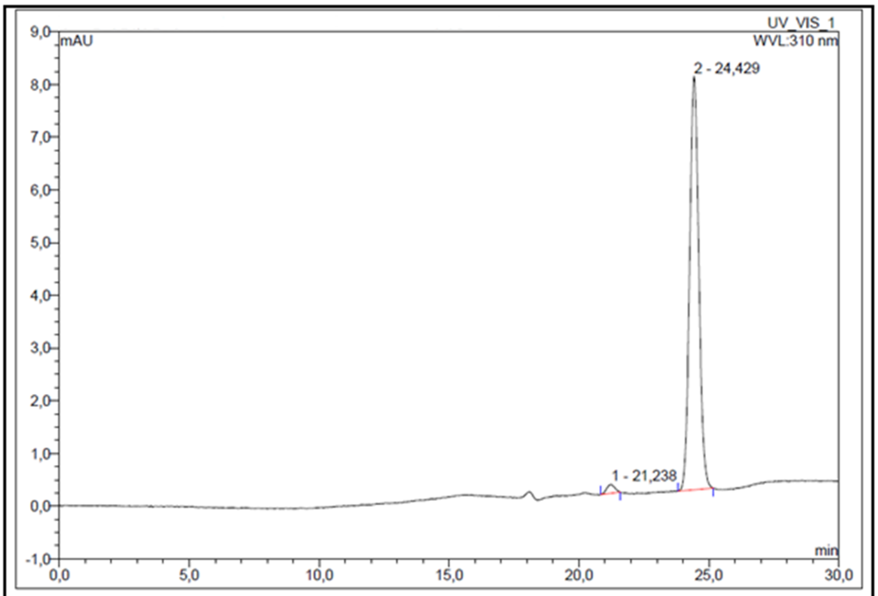

No.	Ret.Time min	Peak Name	Height mAU	Area mAU*min	Rel.Area %	Amount	Type
1	21,24	n.a.	0,171	0,061	1,85	n.a.	BMB
2	24,43	n.a.	7,852	3,219	98,15	n.a.	BMB
Total:			8,023	3,280	100,00	0,000	

Figure S82. HPLC chromatogram of compound **PQM294.**

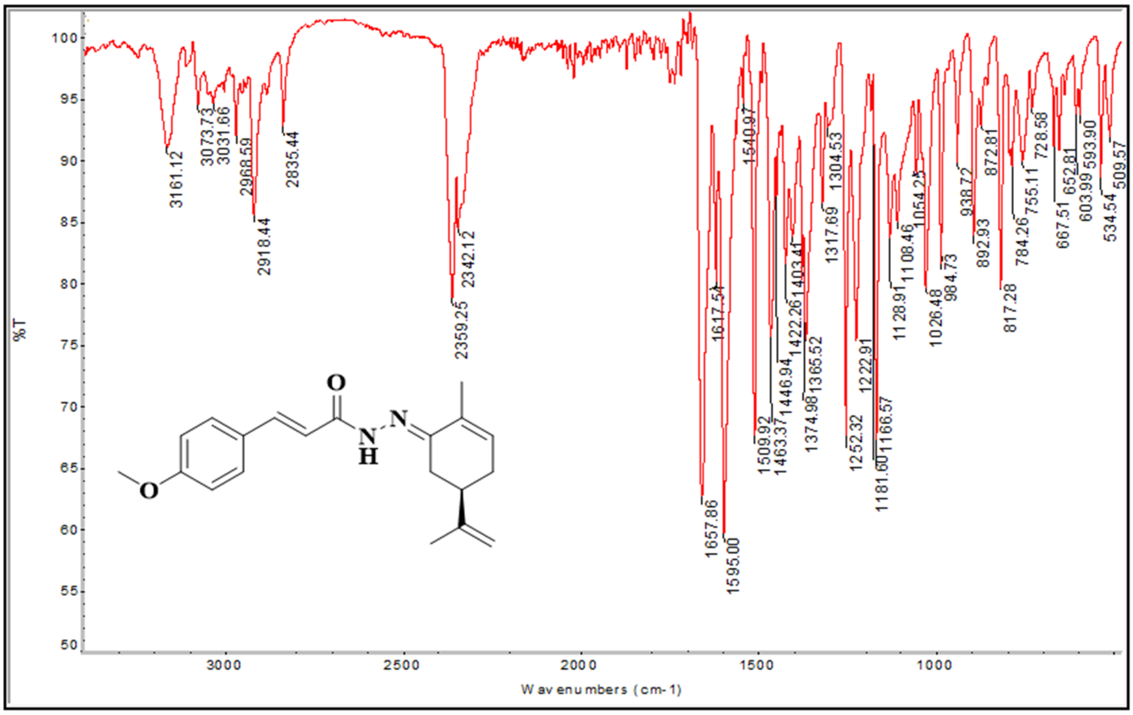

Figure S83. Absorption spectrum in the IR region (ATR) of compound **PQM295**.

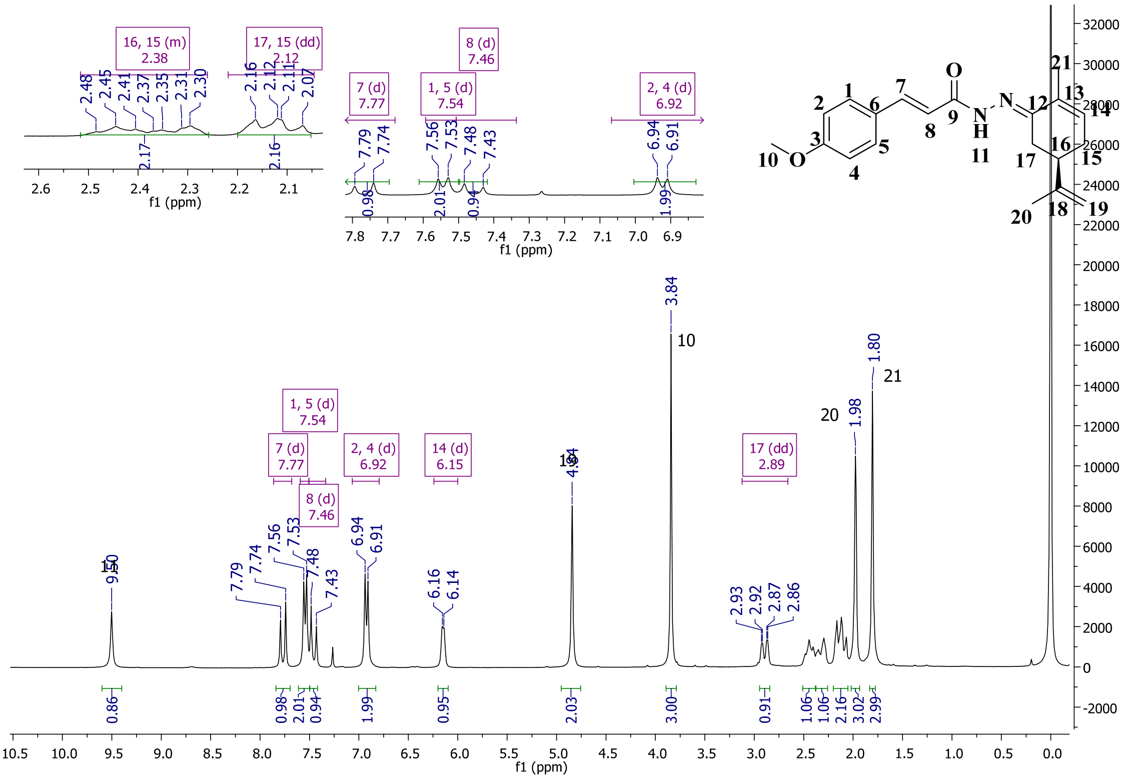

Figure S84. ^{1}H NMR spectrum (300 MHz, $CDCl_3$) of compound **PQM295**.

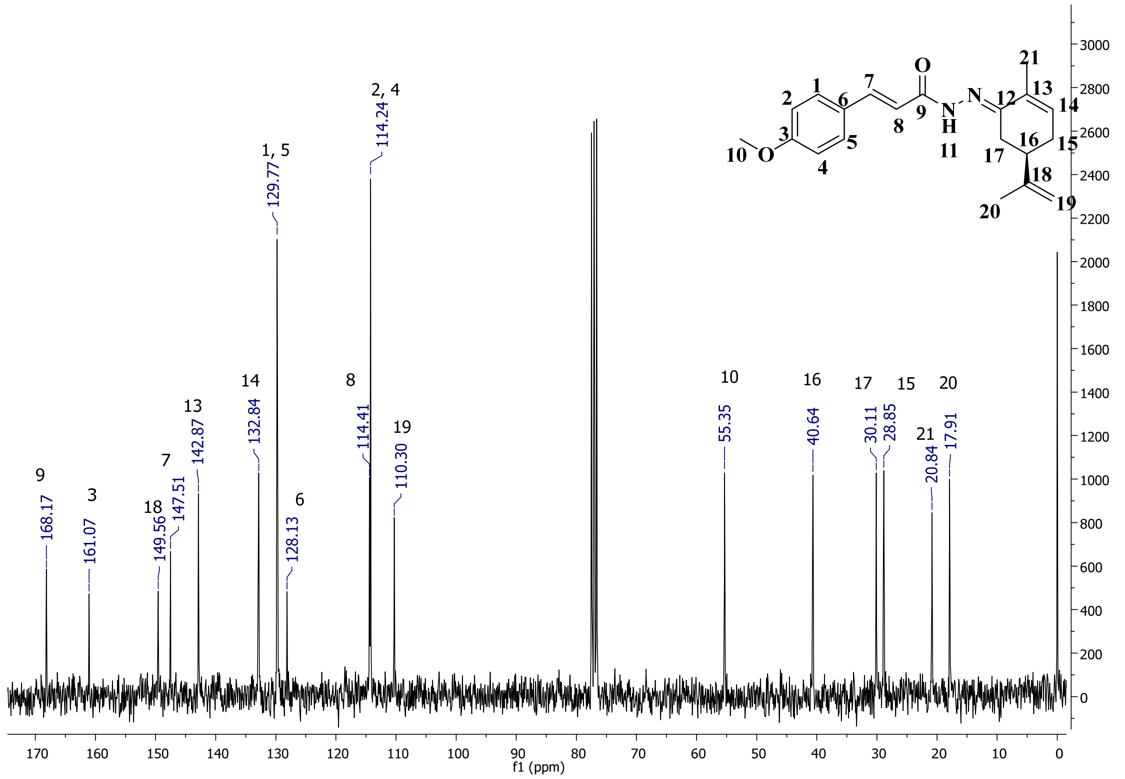

Figure S85. ^{13}C NMR spectrum (75 MHz, $CDCl_3$) of compound **PQM295**.

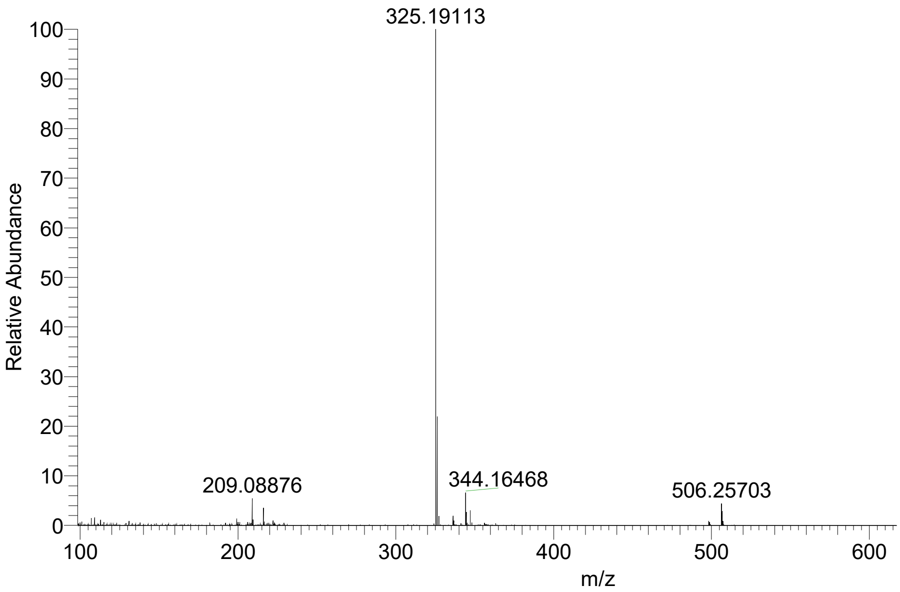

Figure S86. Mass spectrum (ESI-MS) of compound **PQM295**.

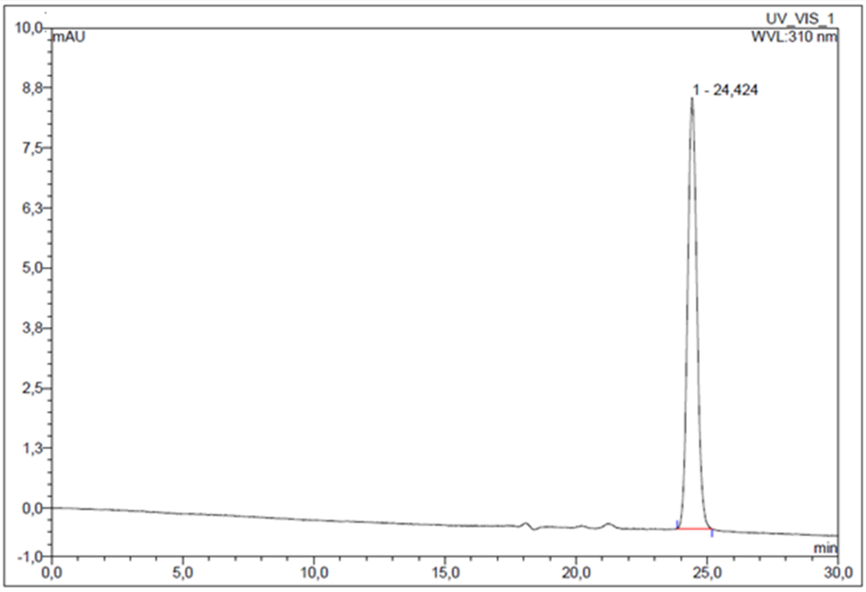

No.	Ret.Time min	Peak Name	Height mAU	Area mAU*min	Rel.Area %	Amount	Type
1	24,42	n.a.	8,977	3,686	100,00	n.a.	BMB
Total:			8,977	3,686	100,00	0,000	

Figure S87. HPLC chromatogram of compound **PQM295**.

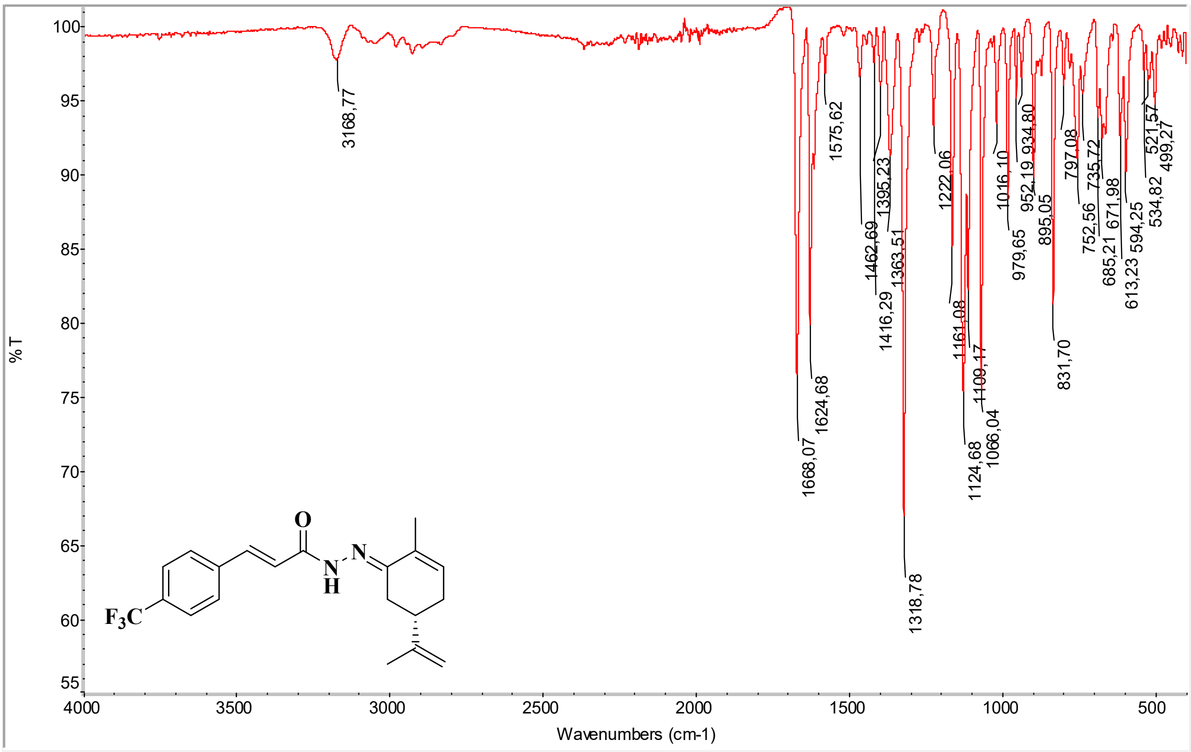

Figure S88. Absorption spectrum in the IR region (ATR) of compound **PQM300**.

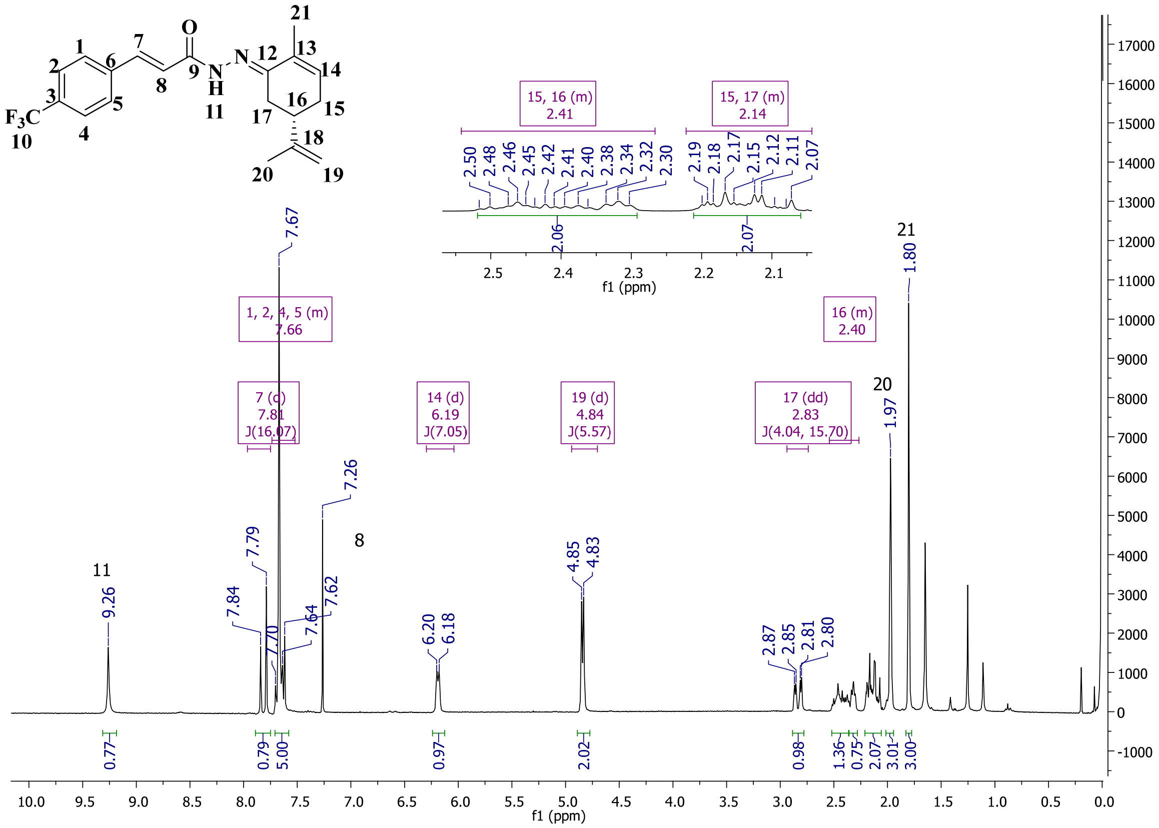

Figure S89. ^{1}H NMR spectrum (300 MHz, $CDCl_3$) of compound **PQM300**.

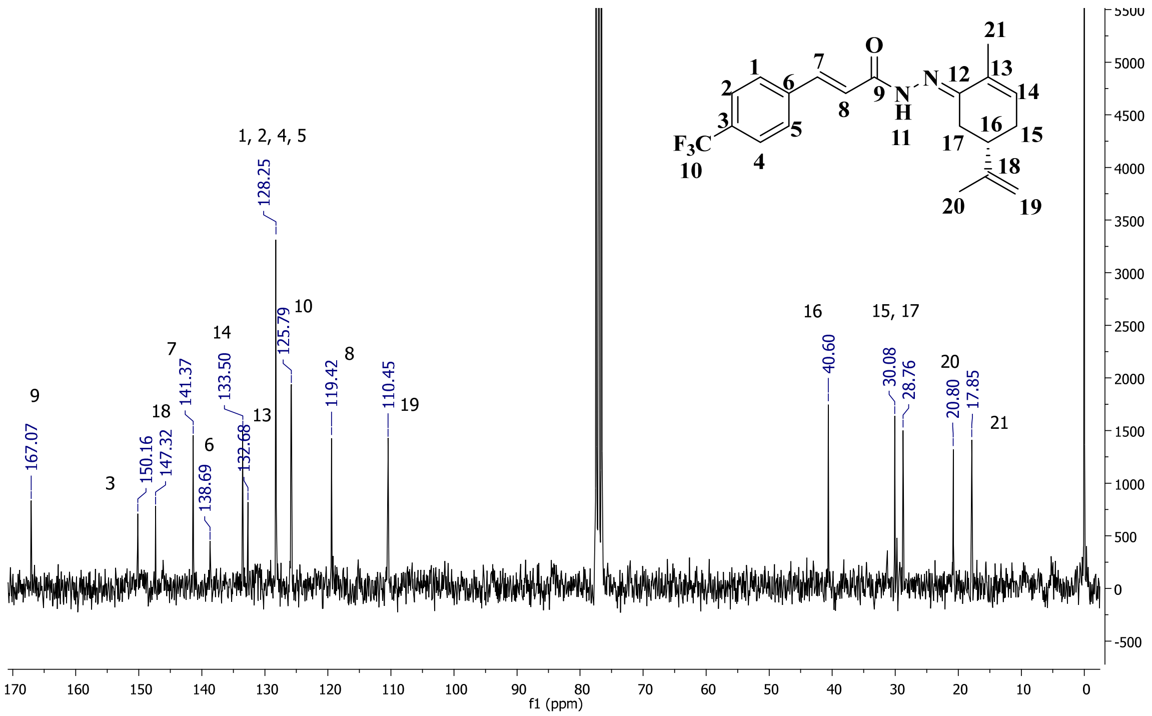

Figure S90. ^{13}C NMR spectrum (75 MHz, $CDCl_3$) of compound **PQM300.**

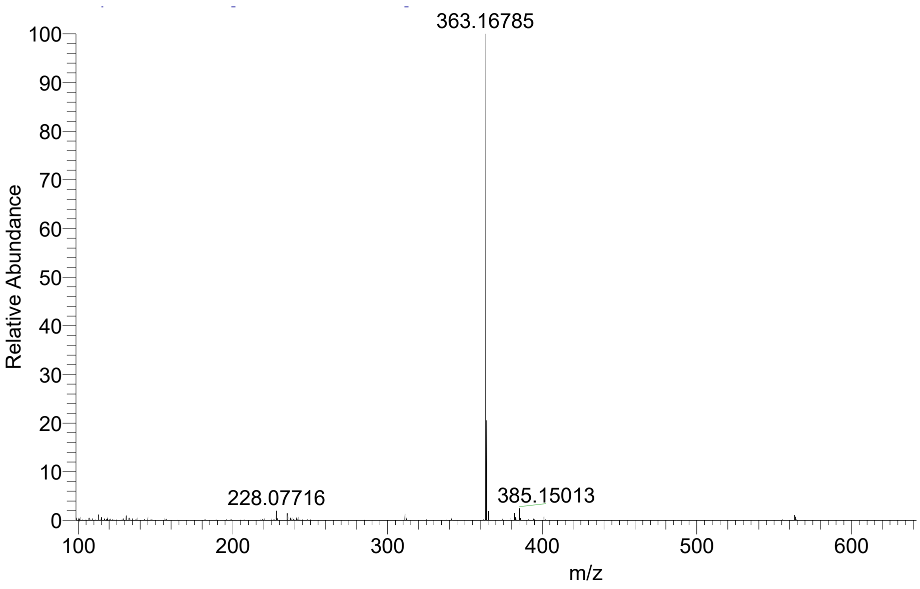

Figure S91. Mass spectrum (ESI-MS) of compound **PQM300**.

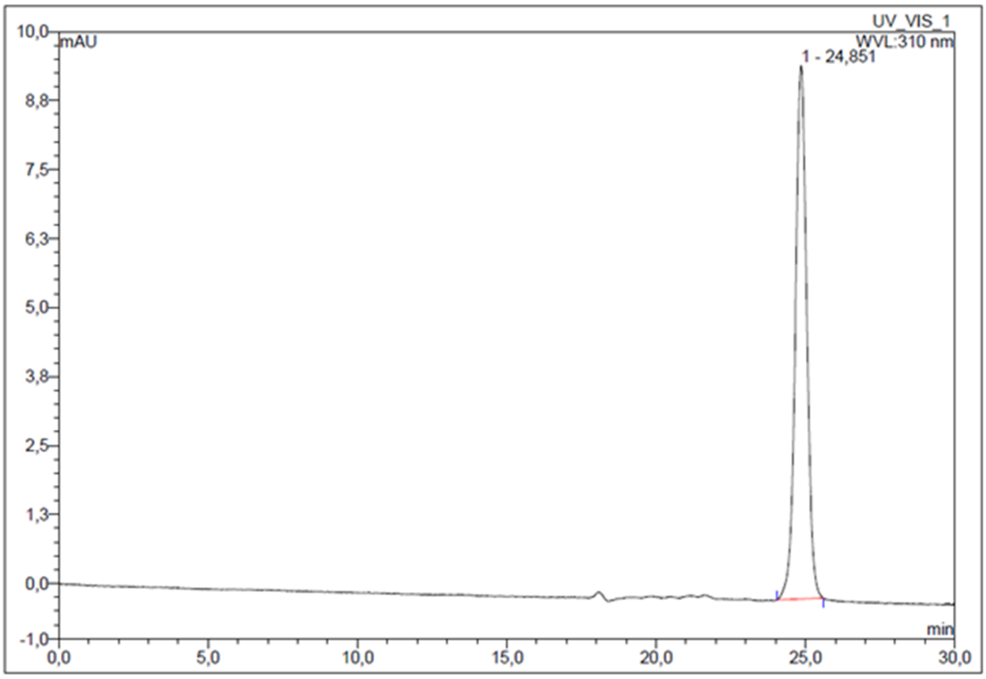

No.	Ret.Time min	Peak Name	Height mAU	Area mAU*min	Rel.Area %	Amount	Type
1	24,85	n.a.	9,664	4,255	100,00	n.a.	BMB
Total:			9,664	4,255	100,00	0,000	

Figure S92. HPLC chromatogram of compound **PQM300**.

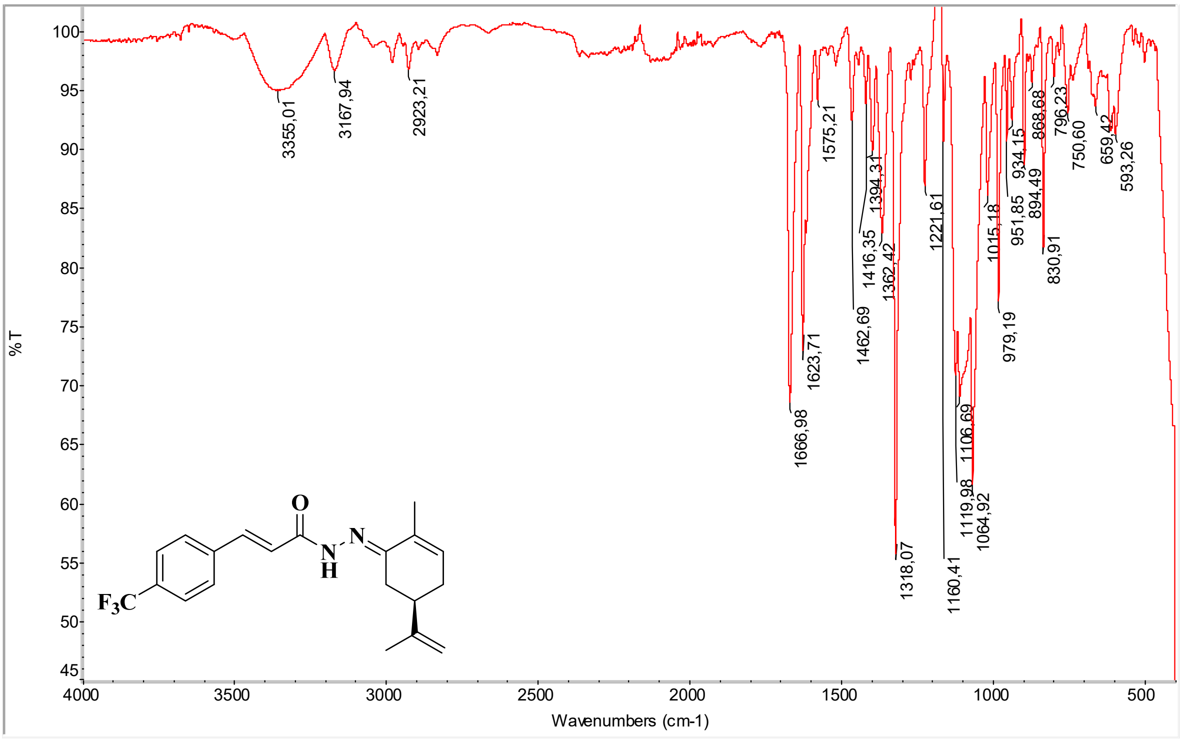

Figure S93. Figure 87. Absorption spectrum in the IR region (ATR) of compound **PQM301**.

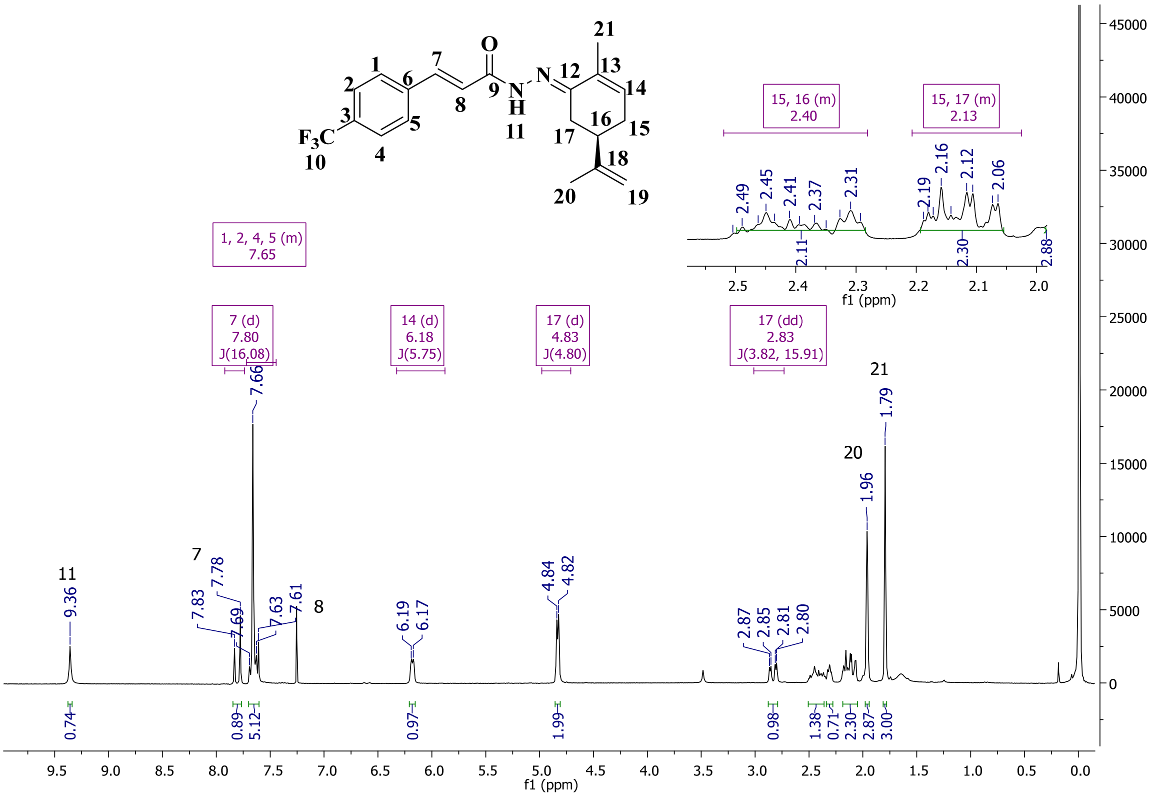

Figure S94. ^{1}H NMR spectrum (300 MHz, $CDCl_3$) of compound **PQM301**.

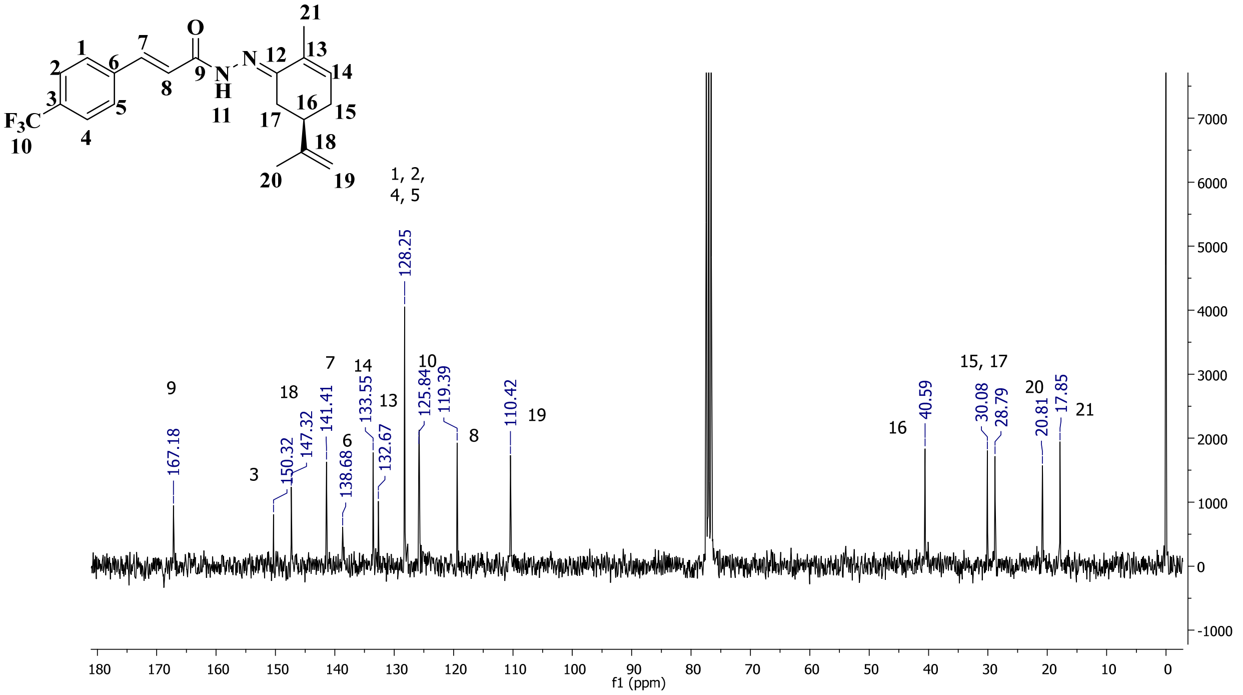

Figure S95. ^{13}C NMR spectrum (75 MHz, $CDCl_3$) of compound **PQM301**.

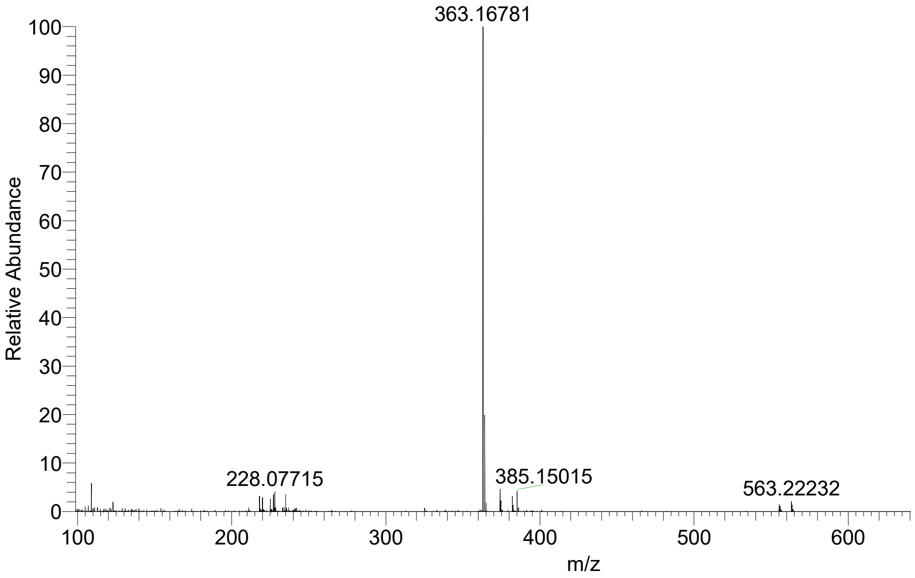

Figure S96. Mass spectrum (ESI-MS) of compound **PQM301**.

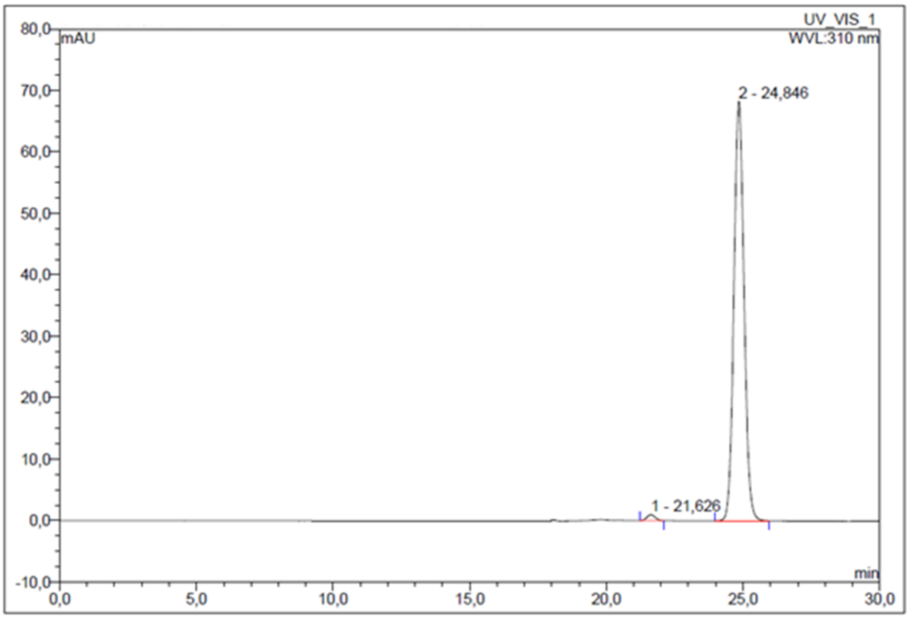

No.	Ret.Time min	Peak Name	Height mAU	Area mAU*min	Rel.Area %	Amount	Type
1	21,63	n.a.	0,955	0,334	1,12	n.a.	BMB
2	24,85	n.a.	68,311	29,388	98,88	n.a.	BMB
Total:			69,266	29,722	100,00	0,000	

Figure S97. HPLC chromatogram of compound **PQM301**.

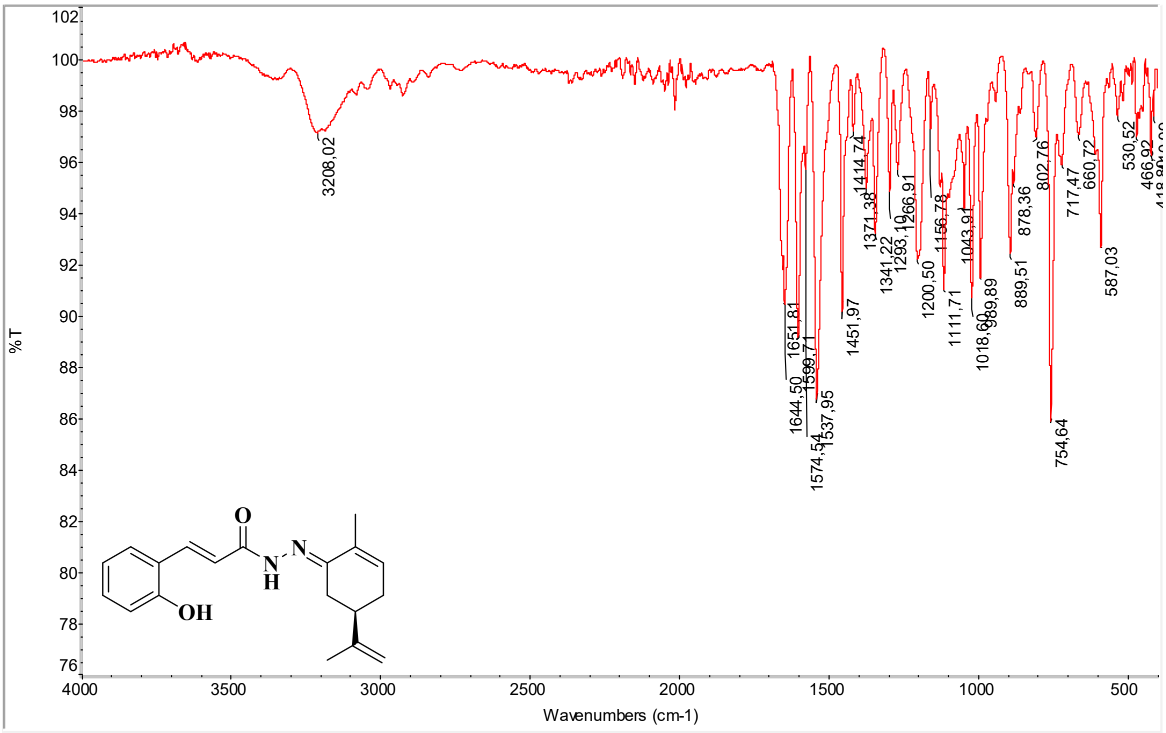

Figure S98. Figure 87. Absorption spectrum in the IR region (ATR) of compound **PQM303**.

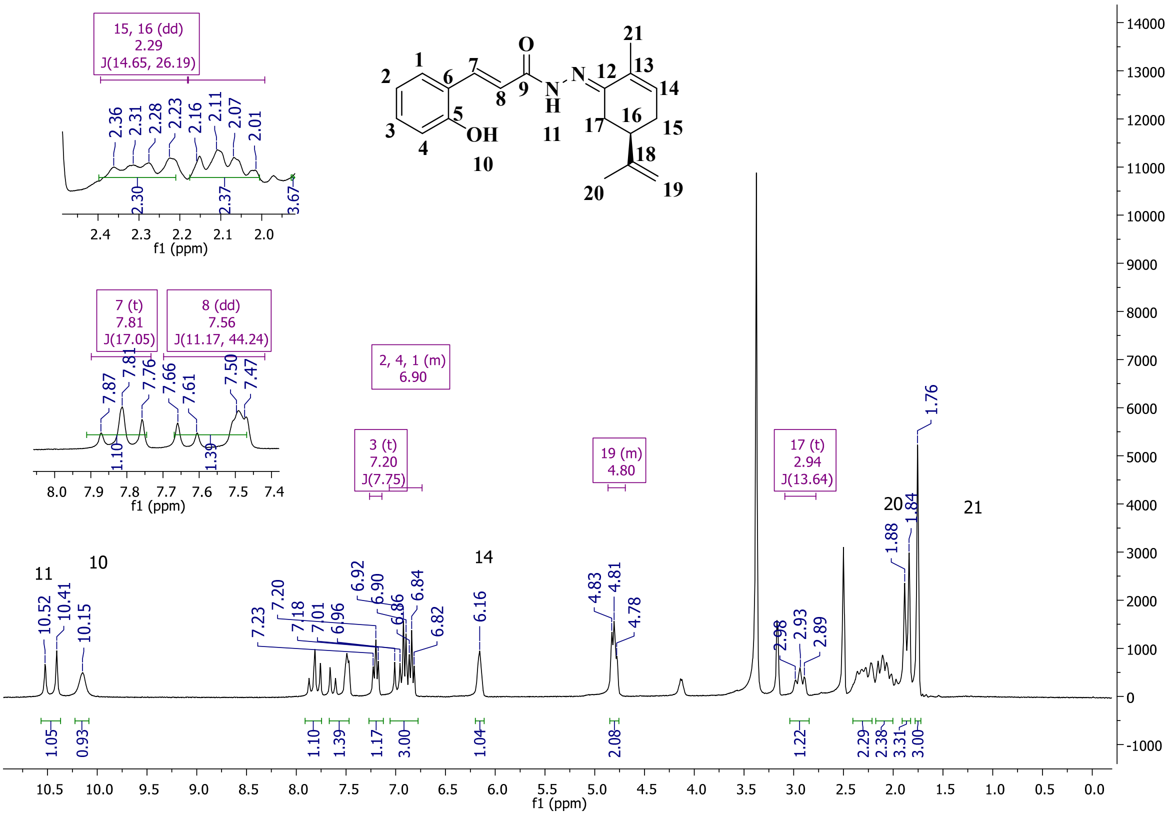

Figure S99. ^{1}H NMR spectrum (300 MHz, DMSO-d_6) of compound **PQM303**.

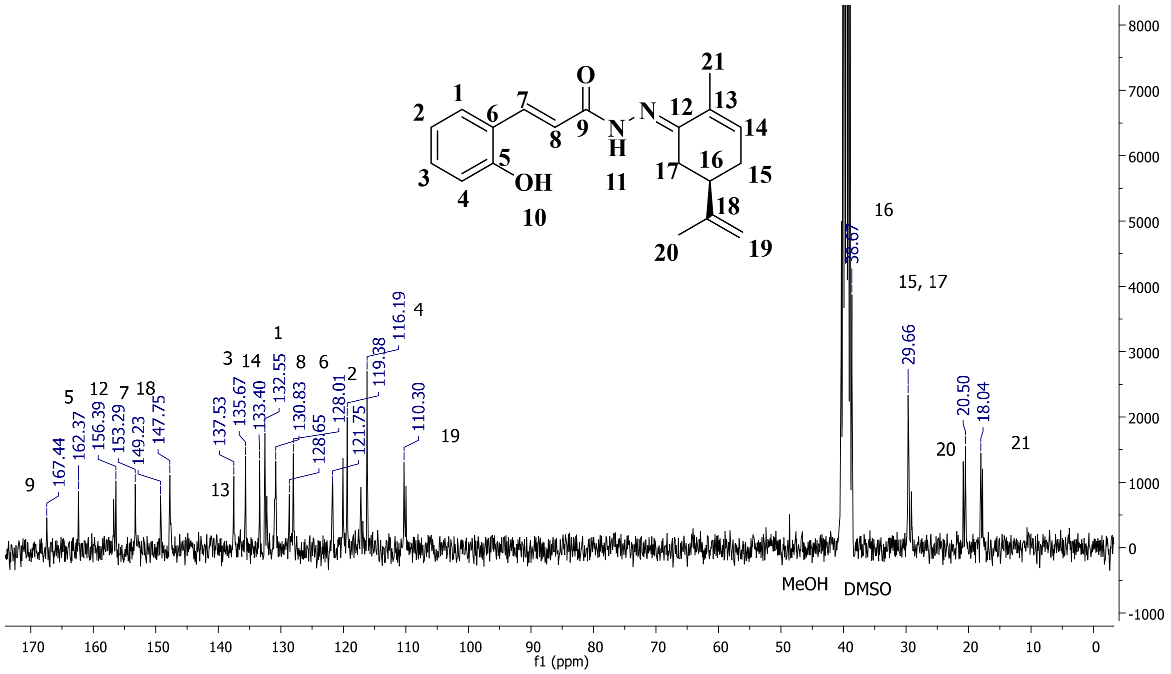

Figure S100. ^{13}C NMR spectrum (75 MHz, DMSO-d_6) of compound **PQM303**.

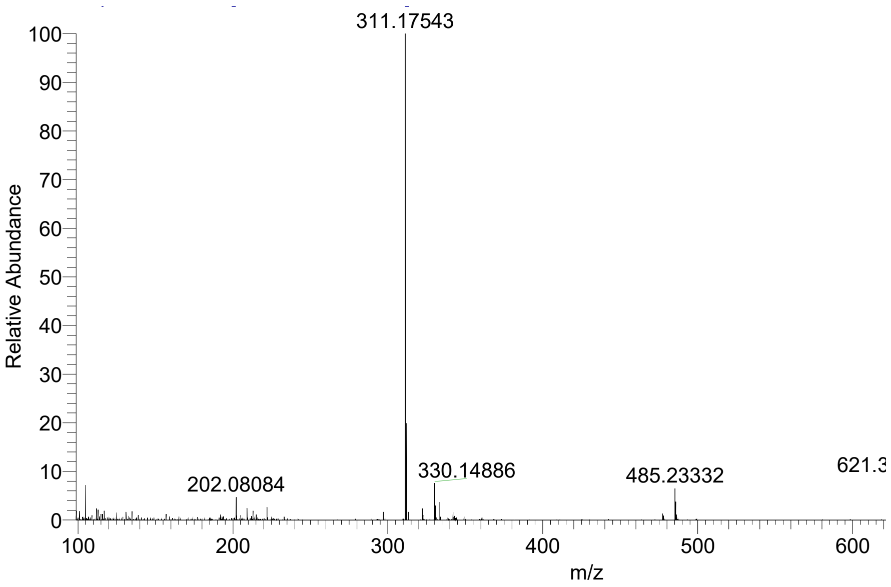

Figure S101. Mass spectrum (ESI-MS) of compound **PQM303**.

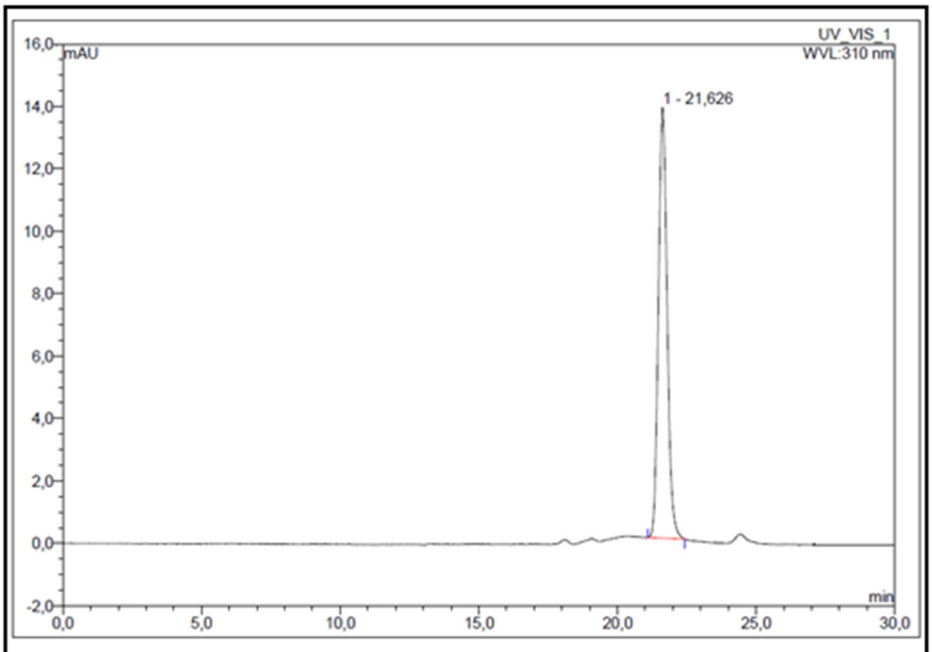

No.	Ret.Time min	Peak Name	Height mAU	Area mAU*min	Rel.Area %	Amount	Type
1	21,63	n.a.	13,800	5,036	100,00	n.a.	BMB
Total:			13,800	5,036	100,00	0,000	

Figure S102. HPLC chromatogram of compound **PQM303**.

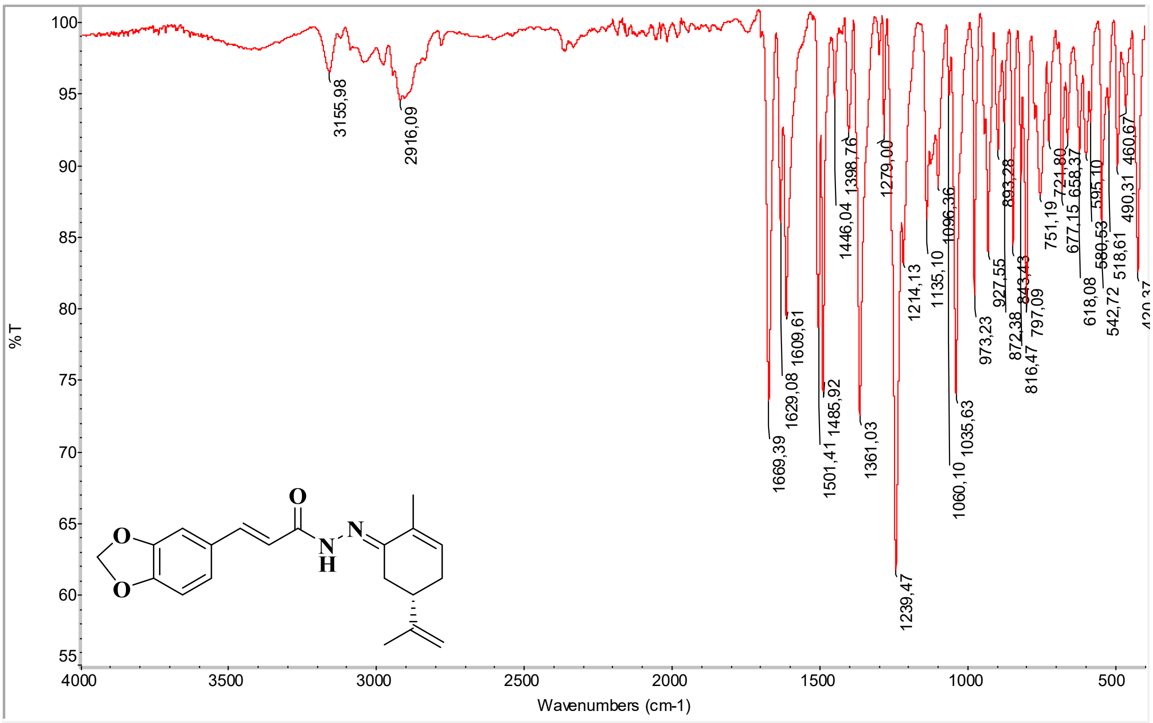

Figure S103. Absorption spectrum in the IR region (ATR) of compound **PQM304**.

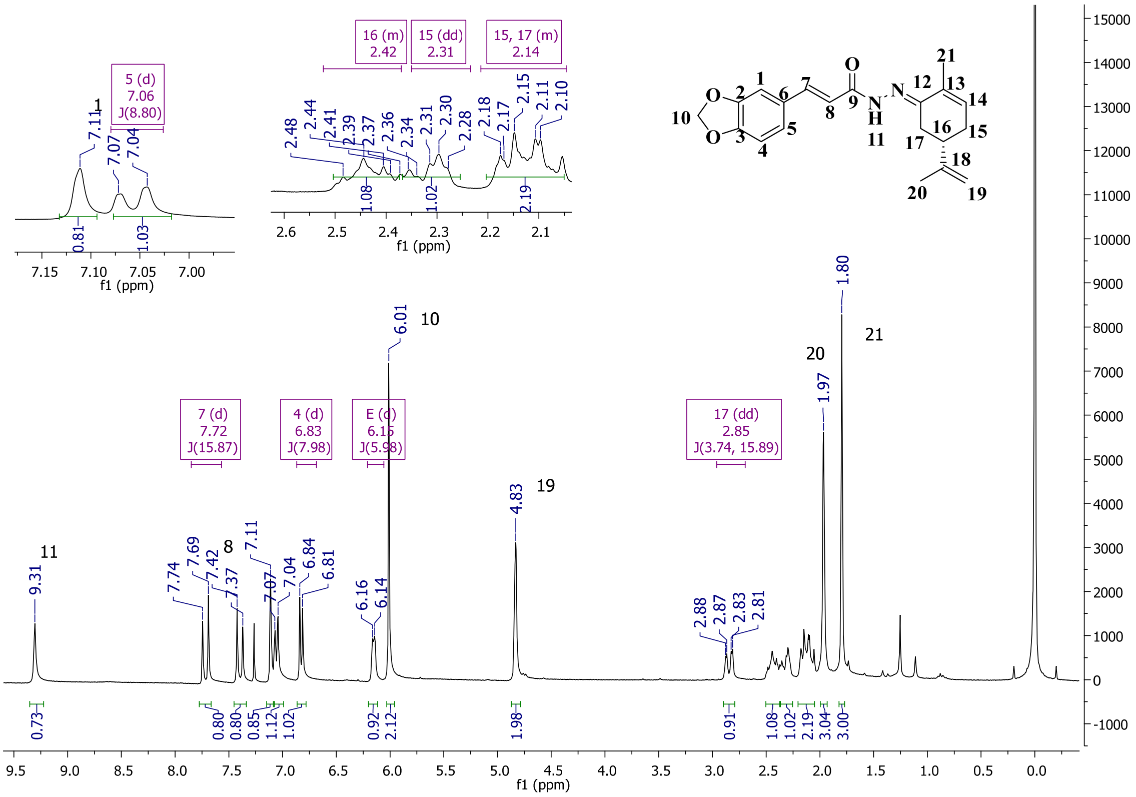

Figure S104. ^{1}H NMR spectrum (300 MHz, $CDCl_3$) of compound **PQM304**.

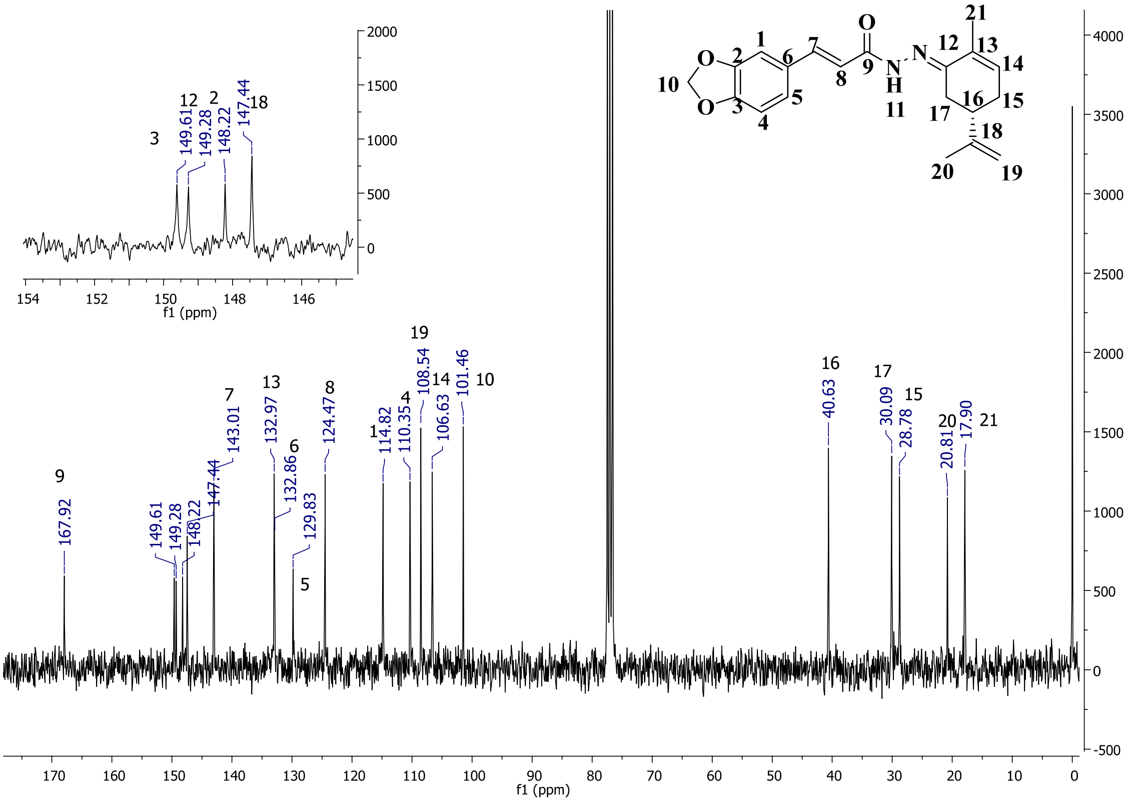

Figure S105. ^{13}C NMR spectrum (75 MHz, $CDCl_3$) of compound **PQM304**.

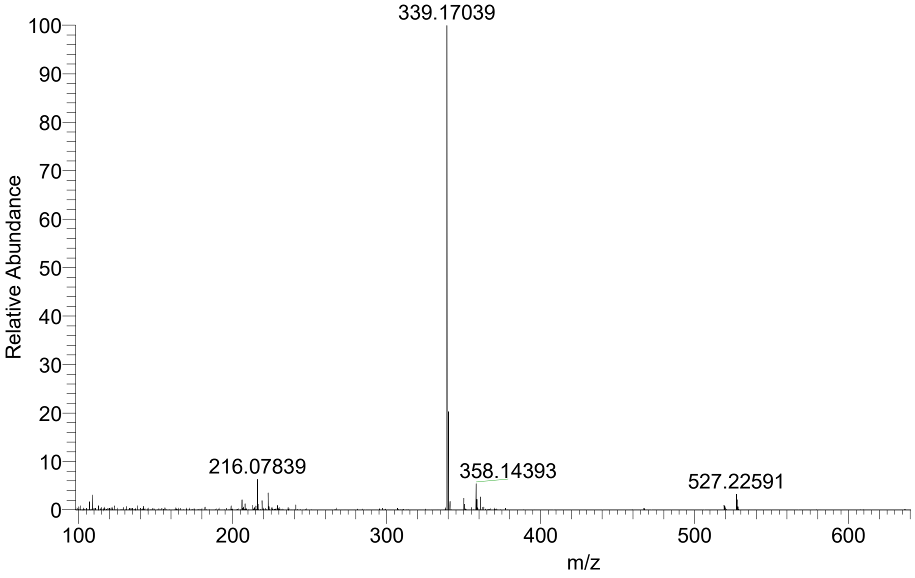

Figure S106. Mass spectrum (ESI-MS) of compound **PQM304**.

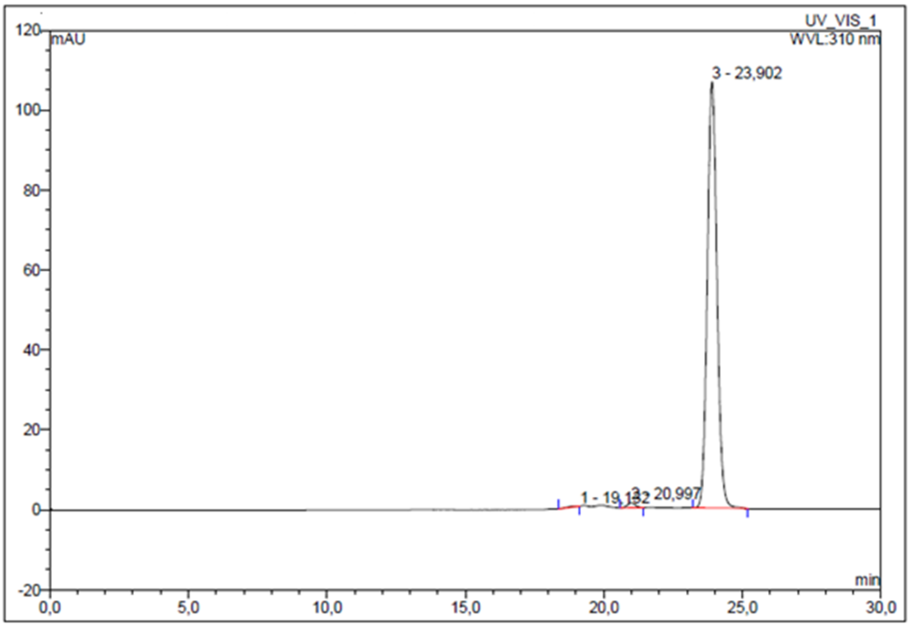

No.	Ret.Time min	Peak Name	Height mAU	Area mAU*min	Rel.Area %	Amount	Type
1	19,13	n.a.	0,000	0,089	0,20	n.a.	BMB
2	21,00	n.a.	1,220	0,422	0,97	n.a.	BMB
3	23,90	n.a.	106,607	43,174	98,83	n.a.	BMB
Total:			107,828	43,684	100,00	0,000	

Figure S107. HPLC chromatogram of compound **PQM304**.

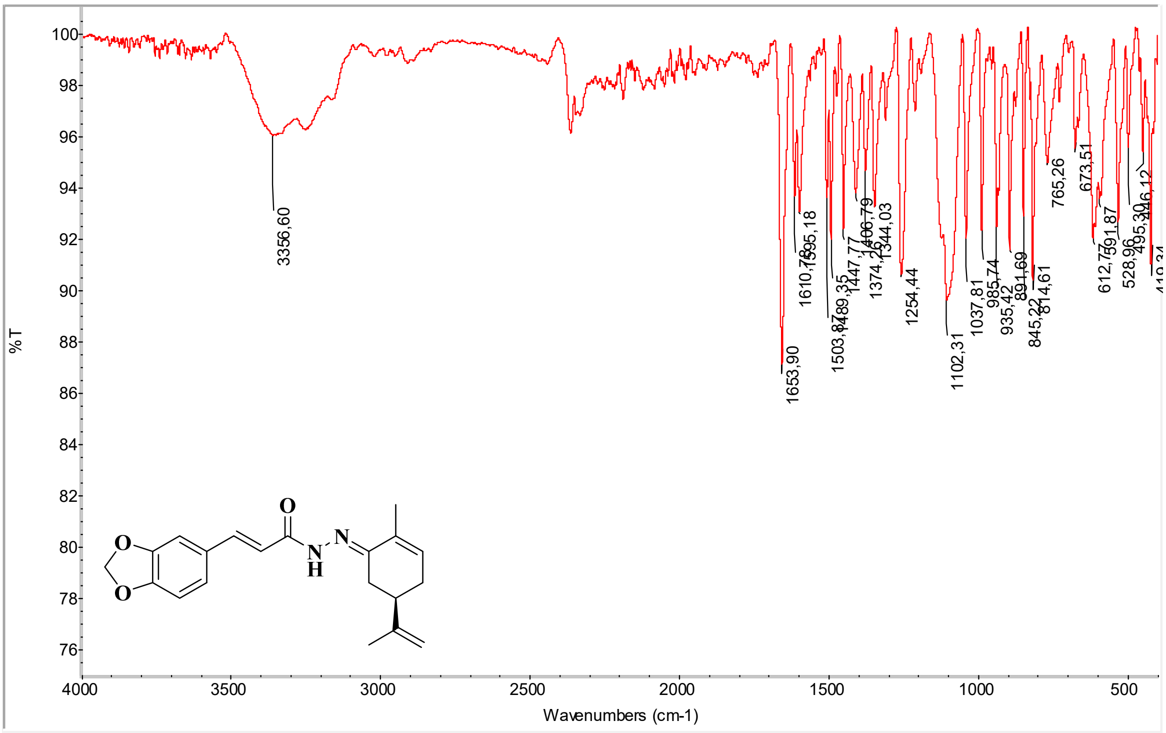

Figure S108. Absorption spectrum in the IR region (ATR) of compound **PQM305**.

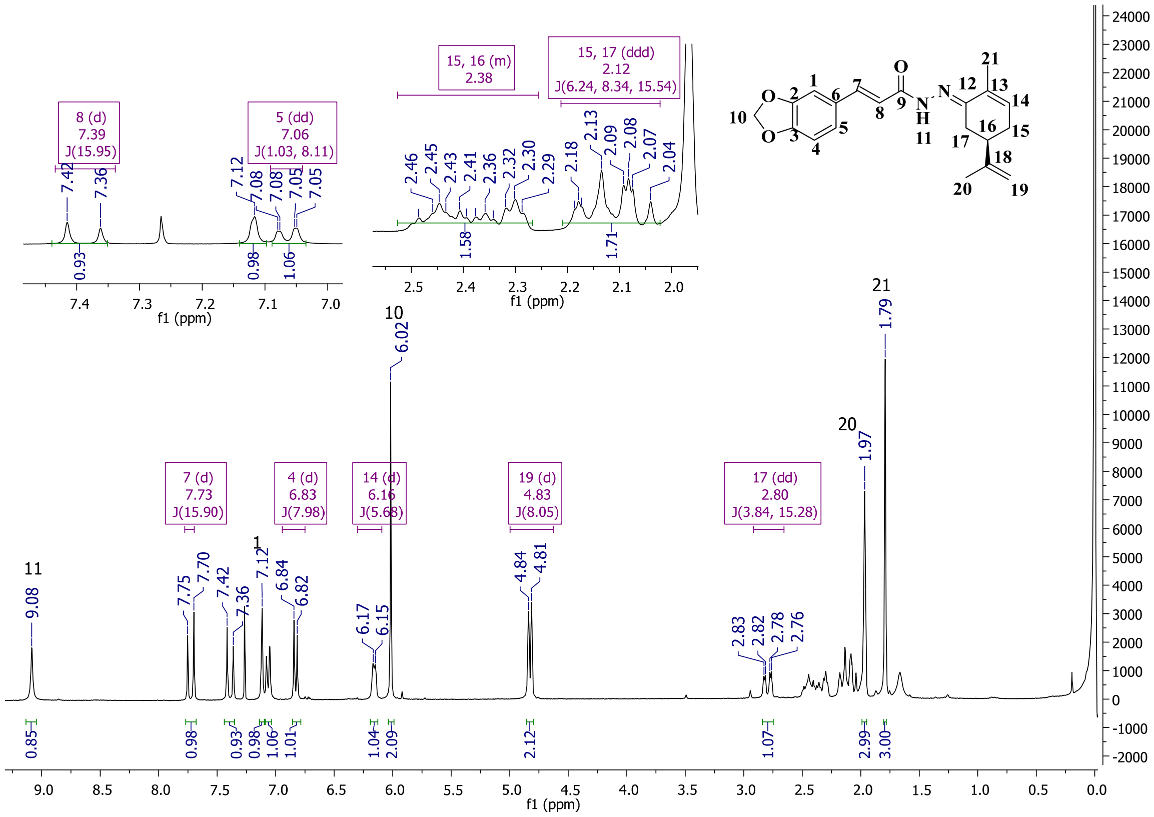

Figure S109. ^{1}H NMR spectrum (300 MHz, $CDCl_3$) of compound **PQM305**.

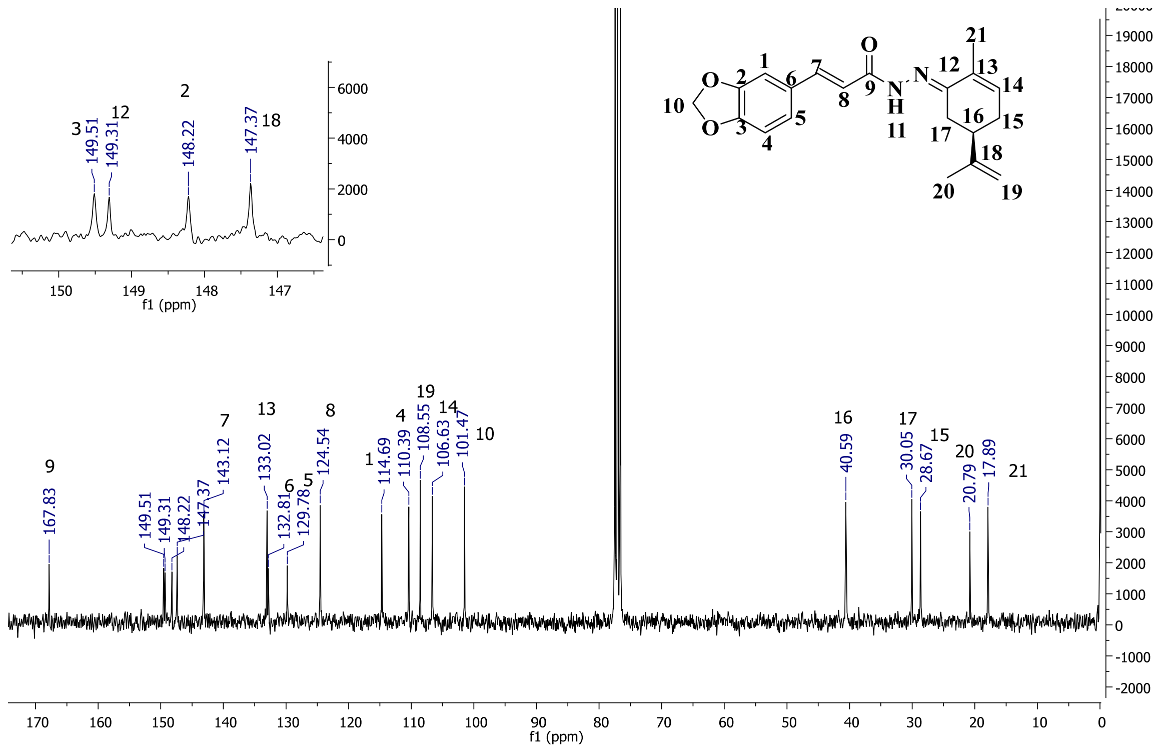

Figure S110. ^{13}C NMR spectrum (75 MHz, $CDCl_3$) of compound **PQM305**.

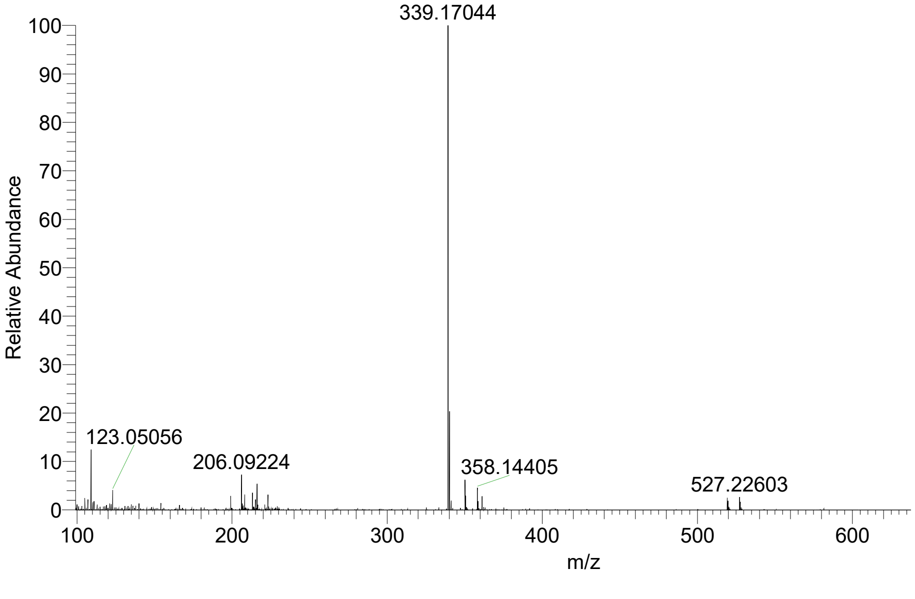

Figure S111. Mass spectrum (ESI-MS) of compound **PQM305**.

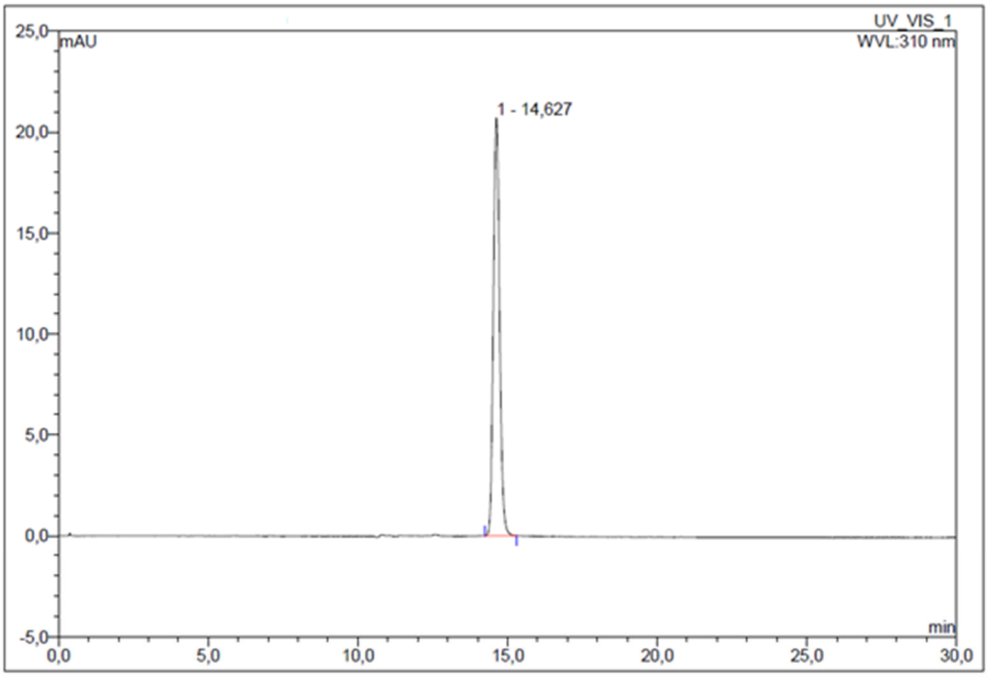

No.	Ret.Time min	Peak Name	Height mAU	Area mAU*min	Rel.Area %	Amount	Type
1	14,63	n.a.	20,700	5,008	100,00	n.a.	BMB
Total:			20,700	5,008	100,00	0,000	

Figure S112. HPLC chromatogram of compound **PQM305**.

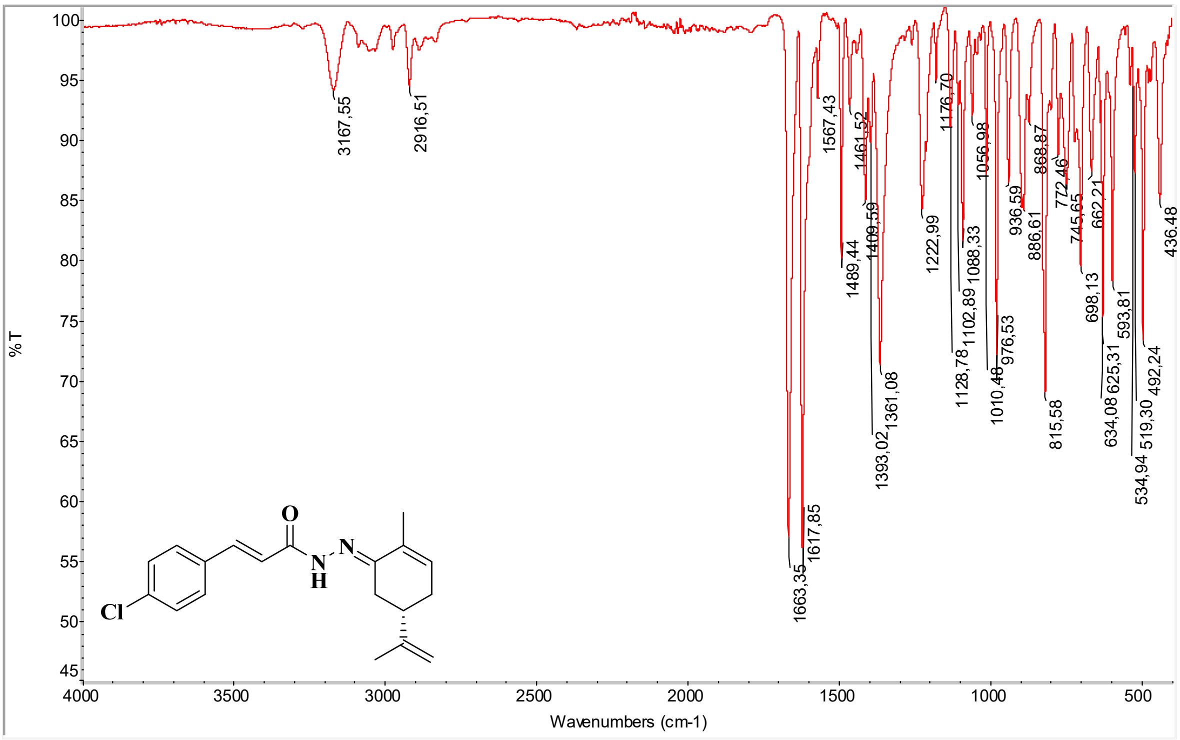

Figure S113. Absorption spectrum in the IR region (ATR) of compound **PQM306**.

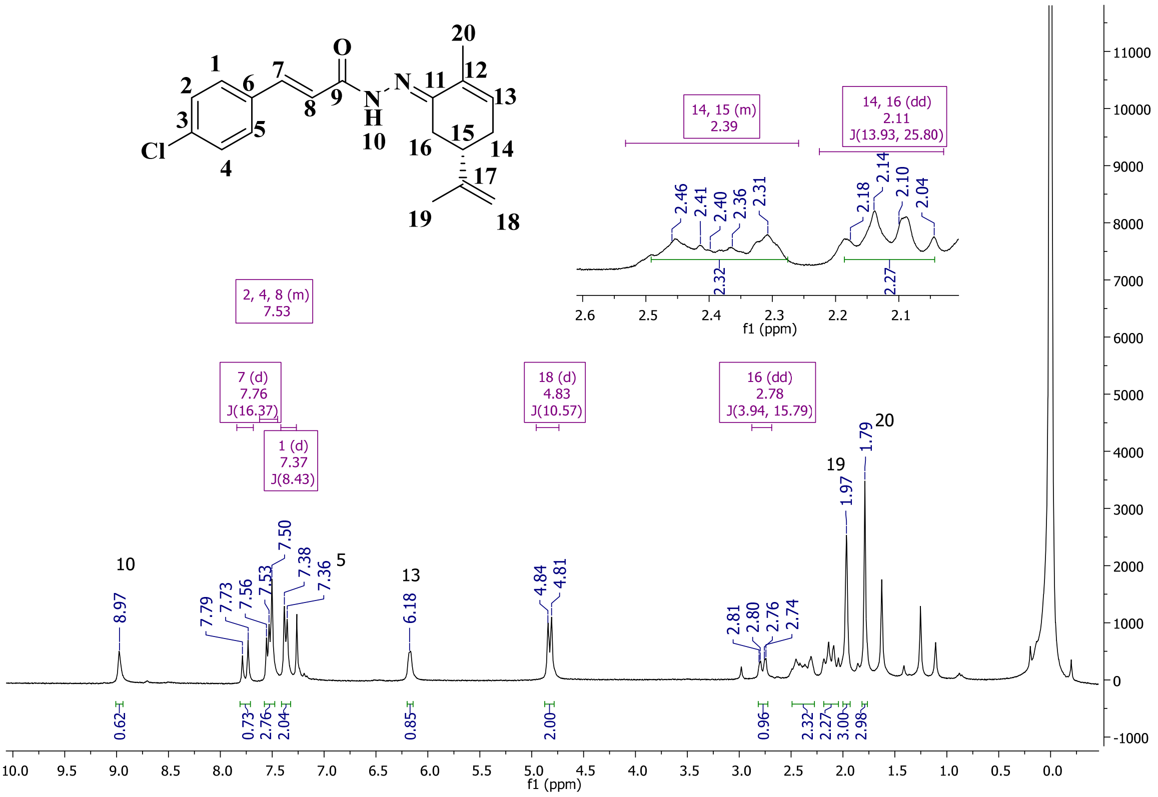

Figure S114. ^{1}H NMR spectrum (300 MHz, $CDCl_3$) of compound **PQM306.**

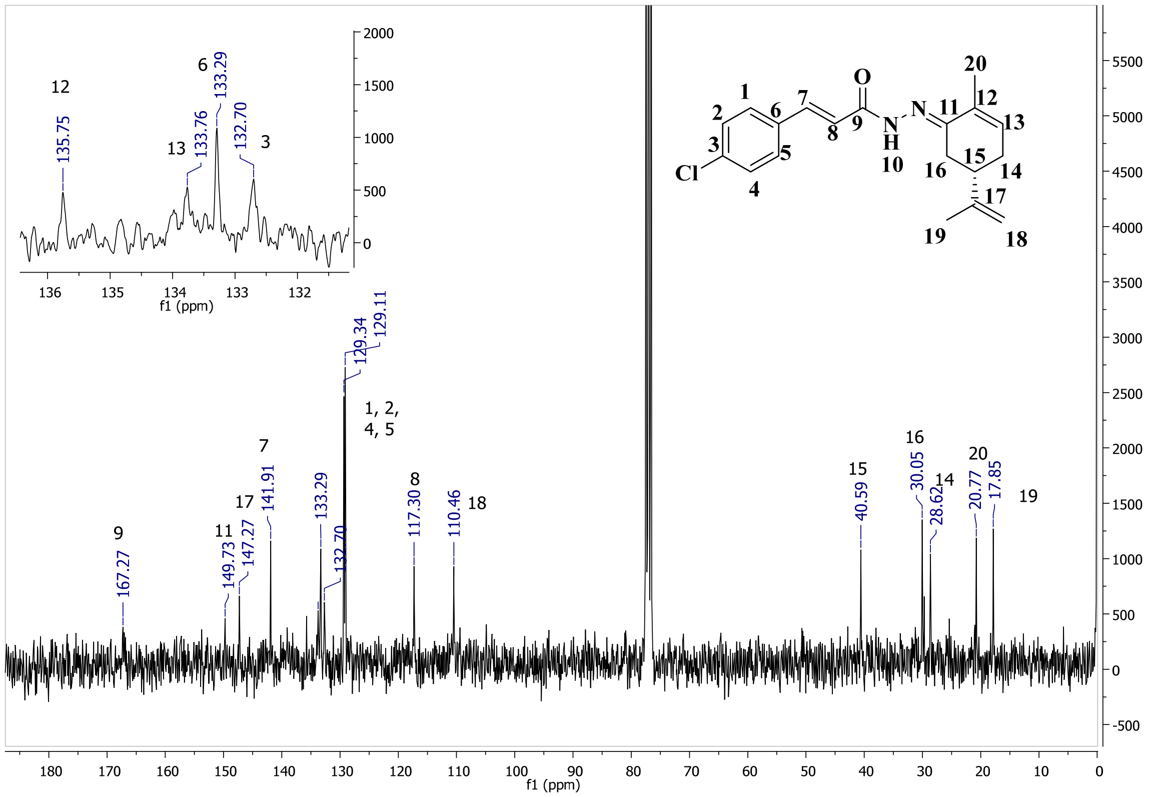

Figure S115. ^{13}C NMR spectrum (75 MHz, $CDCl_3$) of compound **PQM306**.

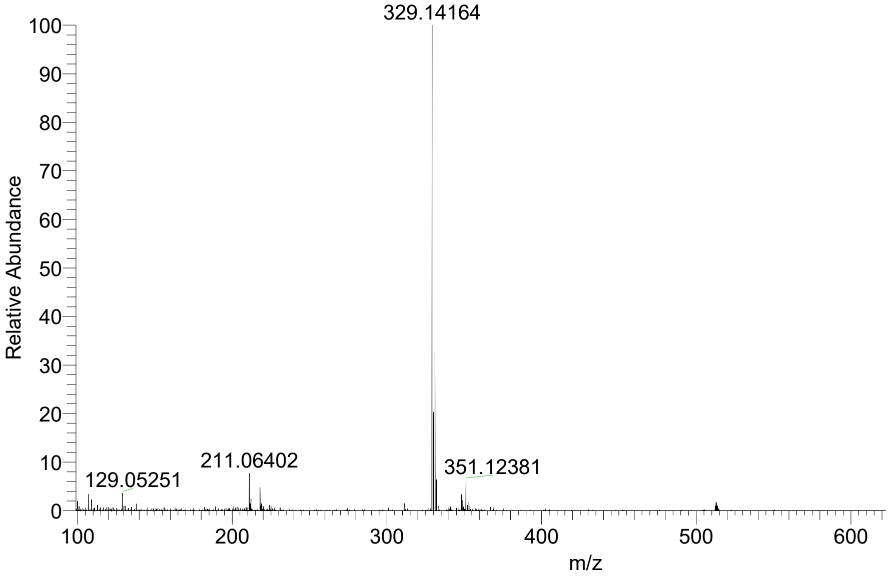

Figure S116. Mass spectrum (ESI-MS) of compound **PQM306**.

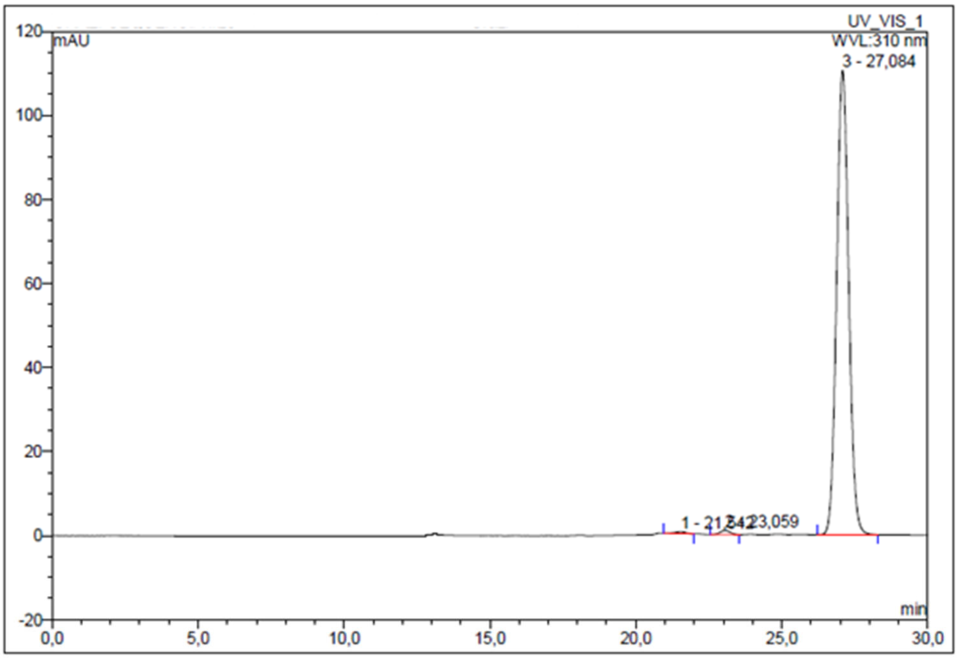

No.	Ret.Time min	Peak Name	Height mAU	Area mAU*min	Rel.Area %	Amount	Type
1	21,54	n.a.	0,445	0,192	0,36	n.a.	BMB
2	23,06	n.a.	1,005	0,394	0,74	n.a.	BMB
3	27,08	n.a.	110,471	52,900	98,90	n.a.	BMB
Total:			111,920	53,486	100,00	0,000	

Figure S117. HPLC chromatogram of compound **PQM306**.

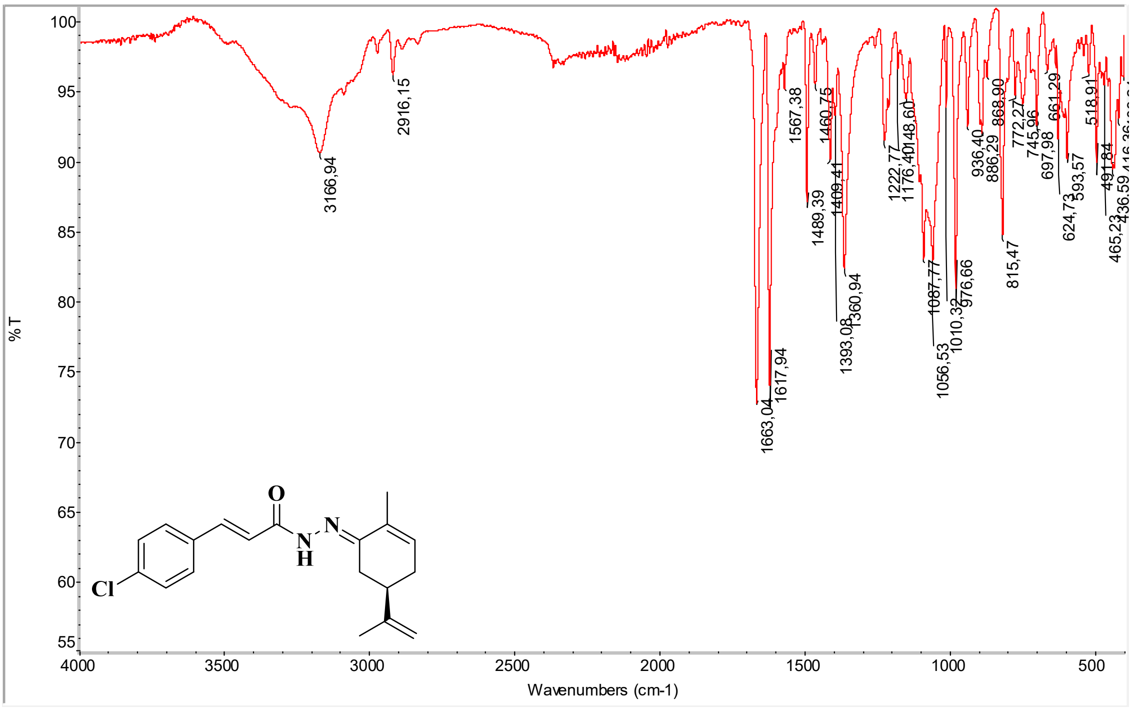

Figure S118. Absorption spectrum in the IR region (ATR) of compound **PQM307**.

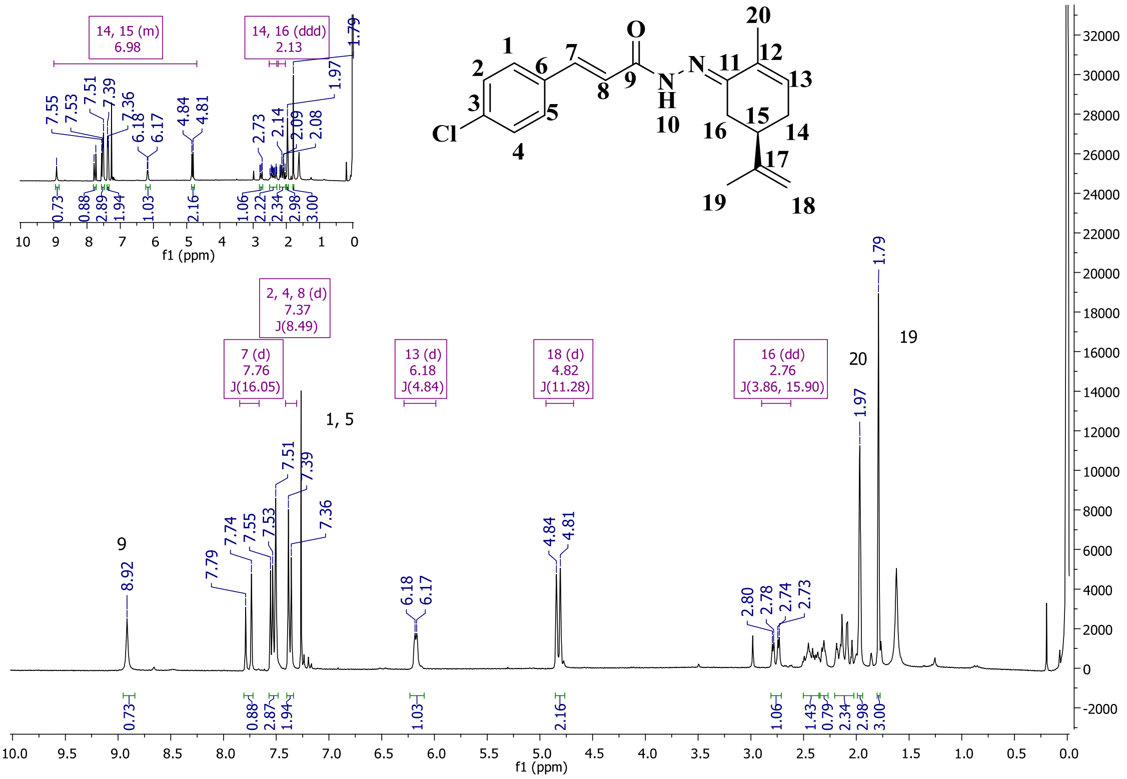

Figure S119. ^{1}H NMR spectrum (300 MHz, $CDCl_3$) of compound **PQM307**.

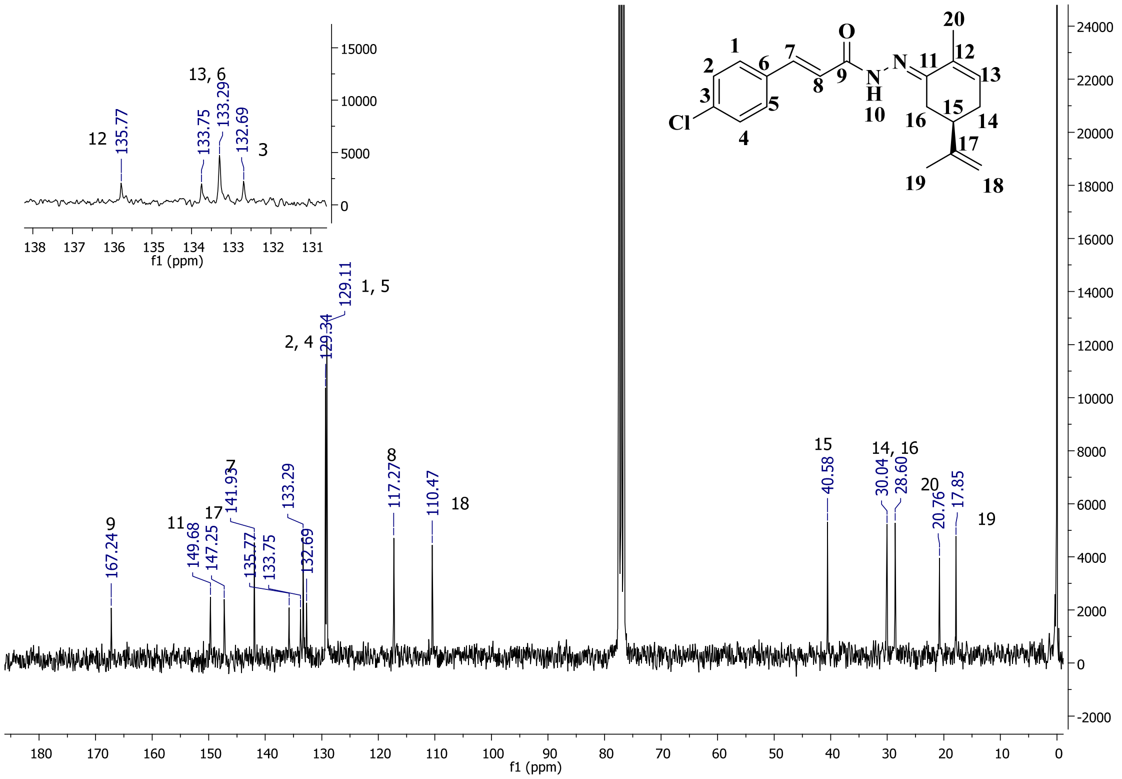

Figure S120. ^{13}C NMR spectrum (75 MHz, $CDCl_3$) of compound **PQM307.**

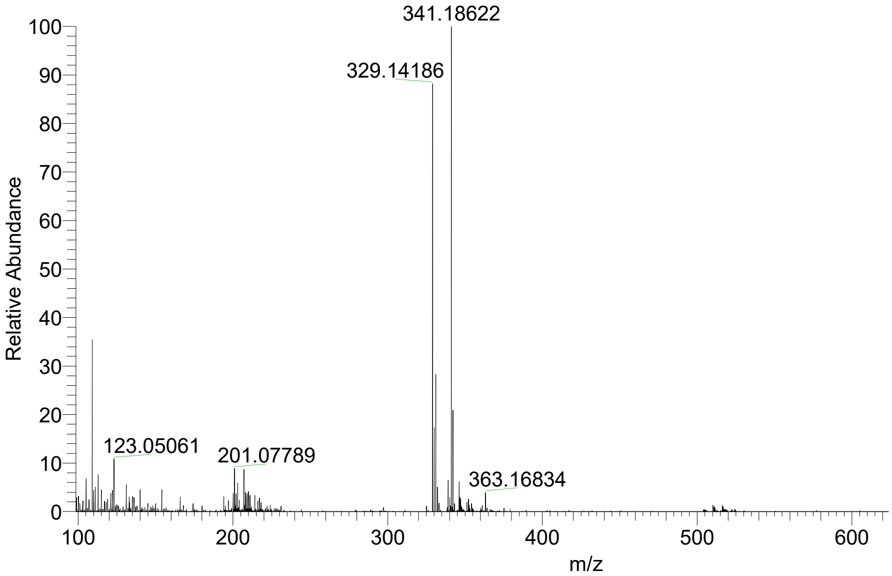

Figure S121. Mass spectrum (ESI-MS) of compound **PQM307**.

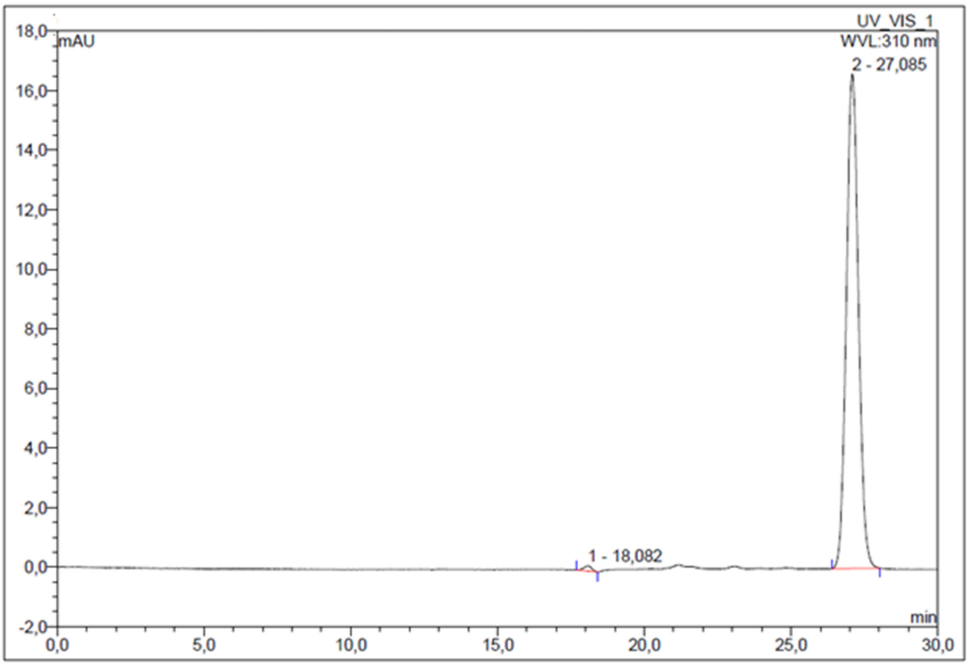

No.	Ret.Time min	Peak Name	Height mAU	Area mAU*min	Rel.Area %	Amount	Type
1	18,08	n.a.	0,179	0,055	0,69	n.a.	BMB
2	27,08	n.a.	16,615	7,926	99,31	n.a.	BMB
Total:			16,793	7,981	100,00	0,000	

Figure S122. HPLC chromatogram of compound **PQM307**.

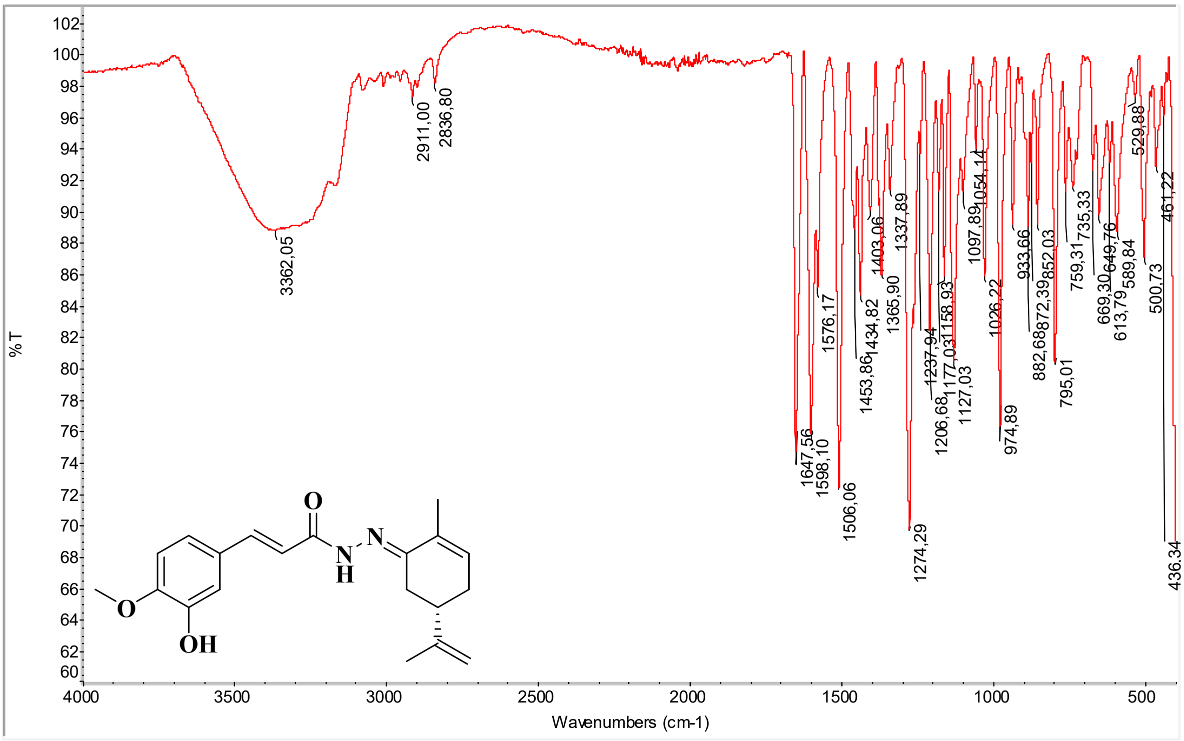

Figure S123. Absorption spectrum in the IR region (ATR) of compound **PQM308**.

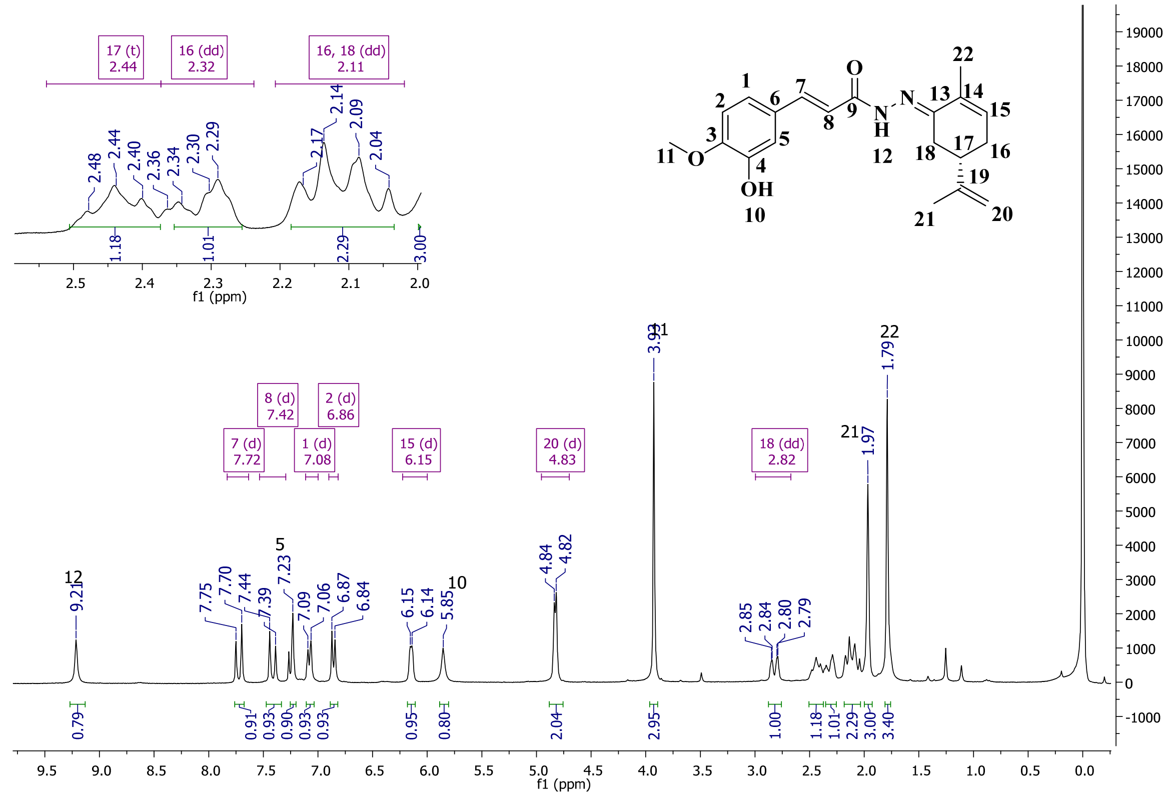

Figure S124. ^{1}H NMR spectrum (300 MHz, $CDCl_3$) of compound **PQM308**.

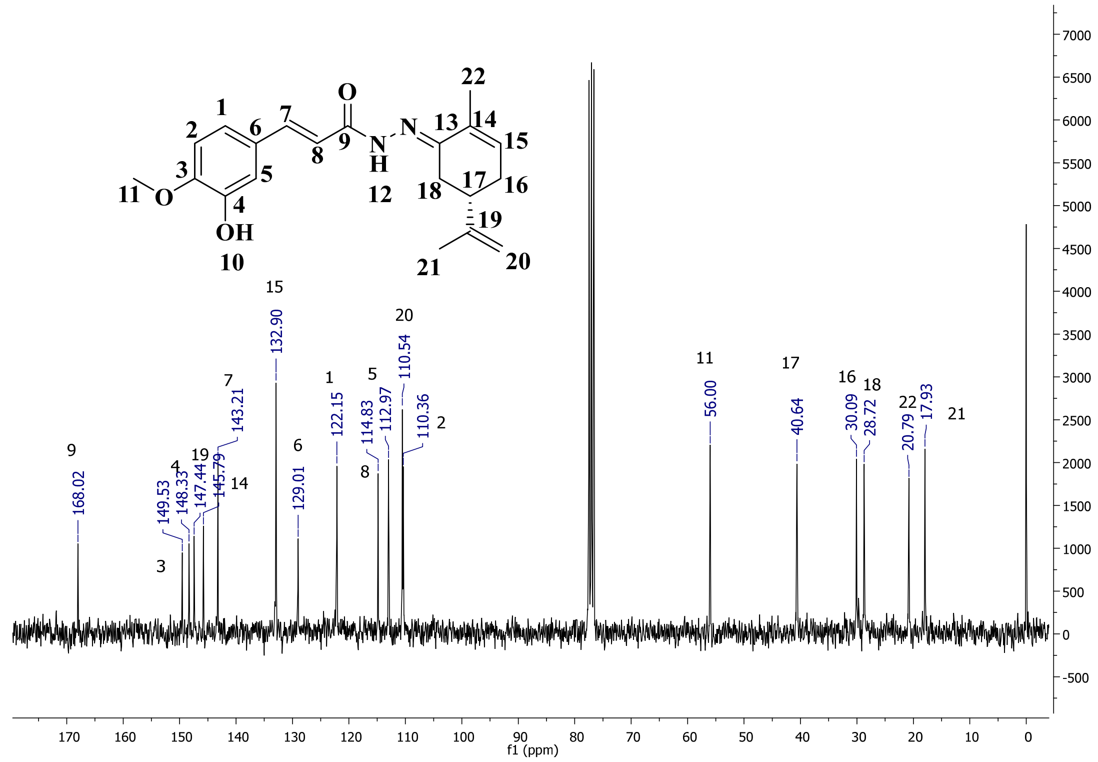

Figure S125. ^{13}C NMR spectrum (75 MHz, $CDCl_3$) of compound **PQM308**.

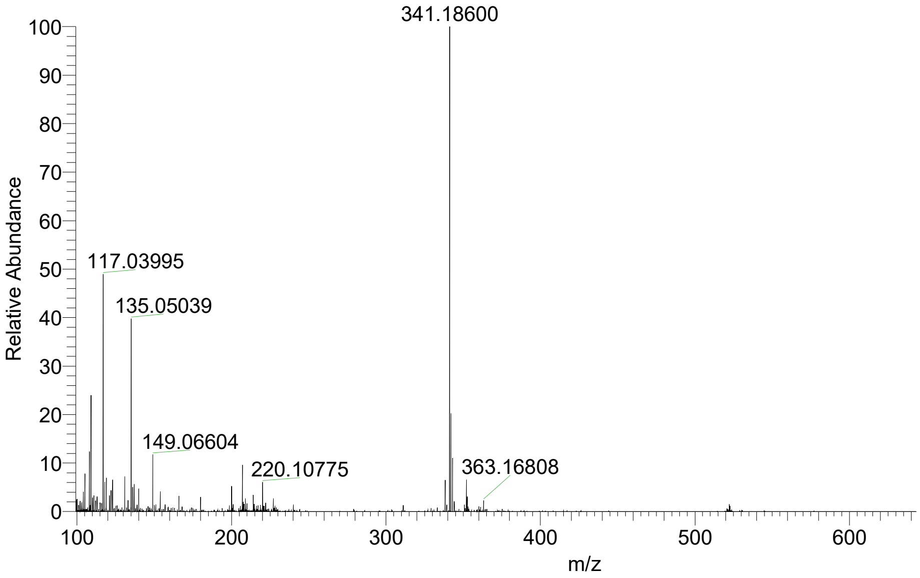

Figure S126. Mass spectrum (ESI-MS) of compound **PQM308**.

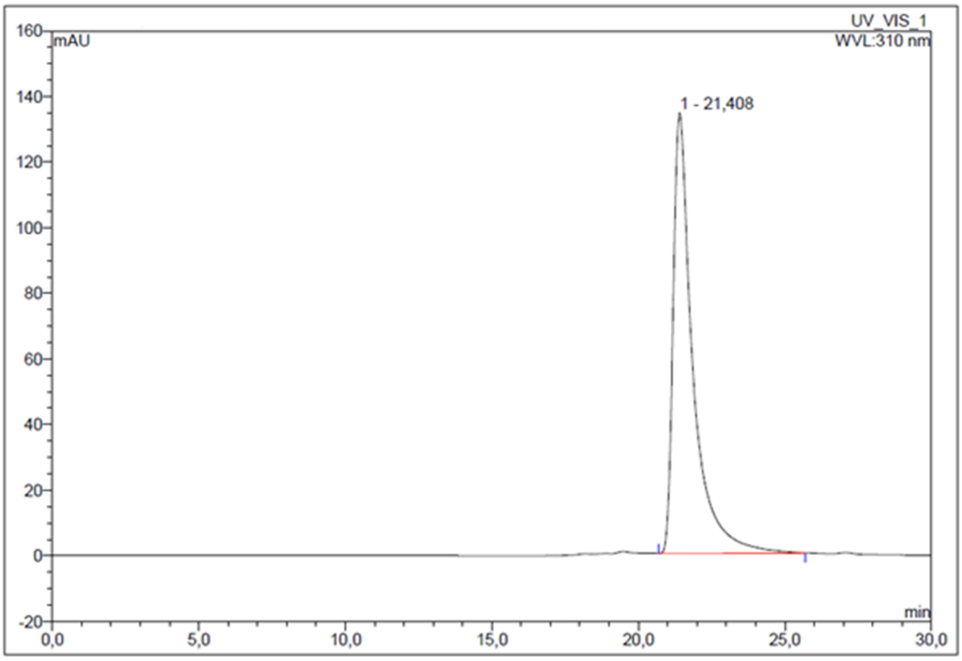

No.	Ret.Time min	Peak Name	Height mAU	Area mAU*min	Rel.Area %	Amount	Type
1	21,41	n.a.	134,310	104,657	100,00	n.a.	BMB
Total:			134,310	104,657	100,00	0,000	

Figure S127. HPLC chromatogram of compound **PQM308**.

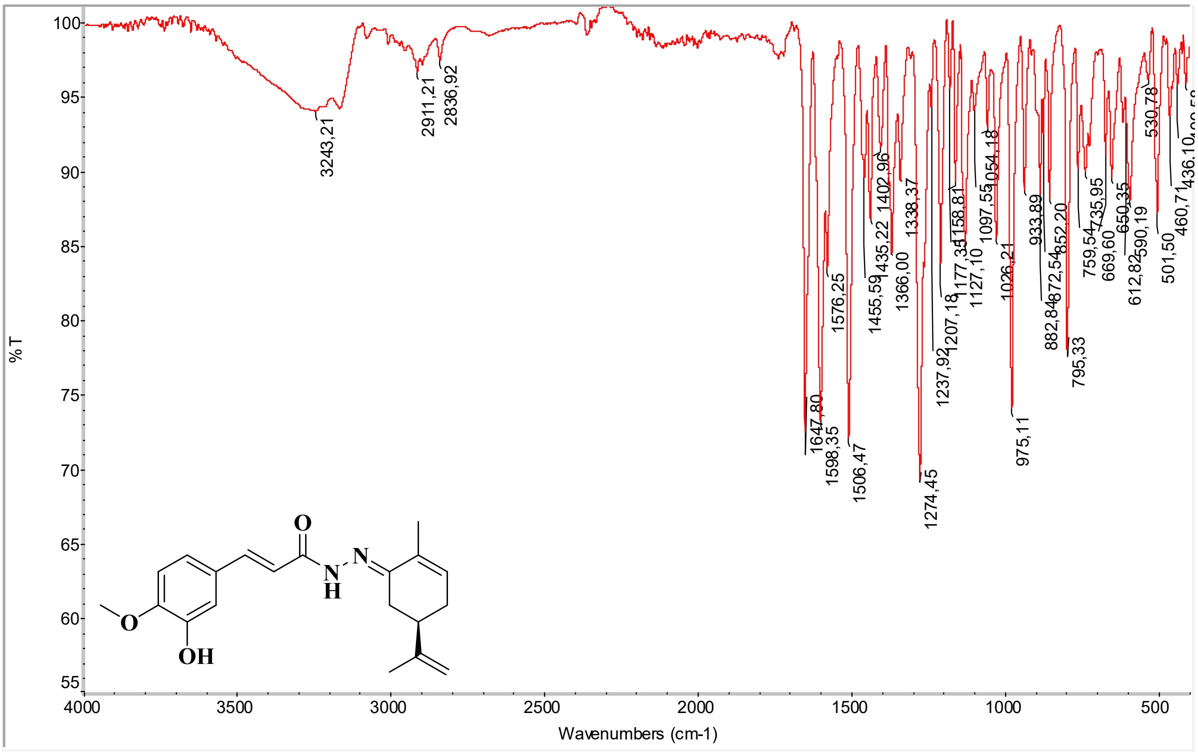

Figure S128. Absorption spectrum in the IR region (ATR) of compound **PQM309**.

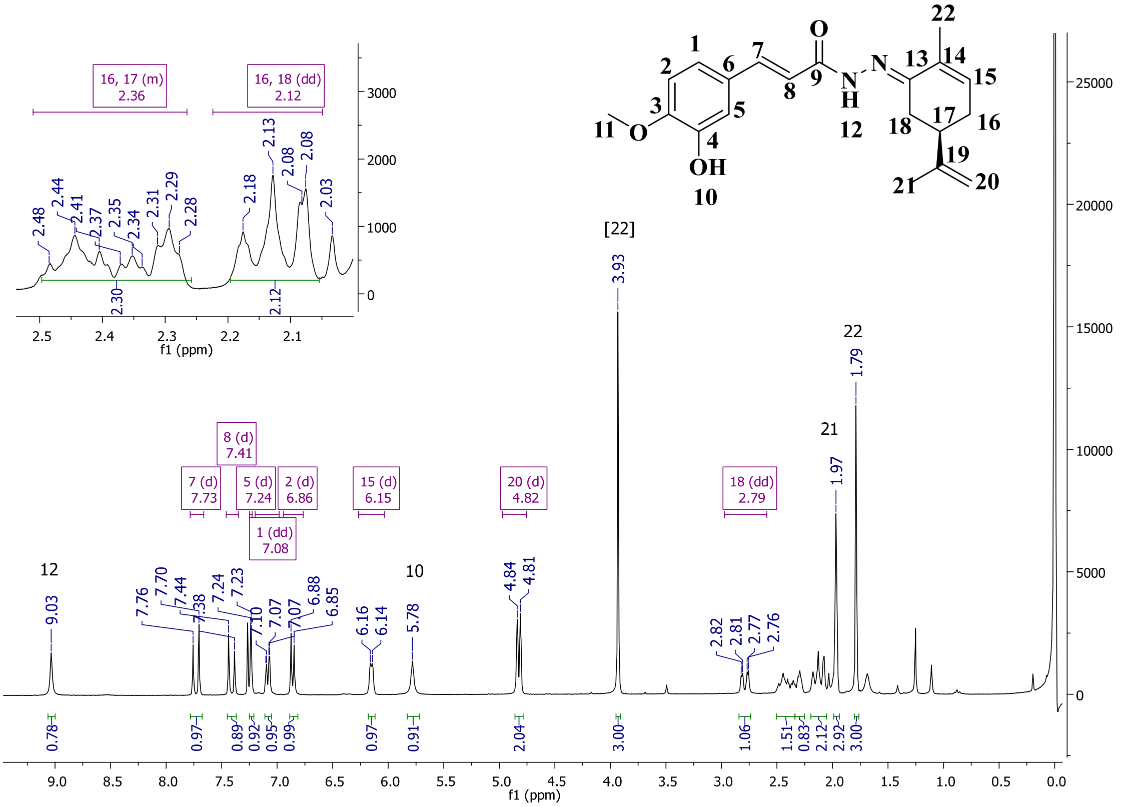

Figure S129. ^{1}H NMR spectrum (300 MHz, $CDCl_3$) of compound **PQM309**.

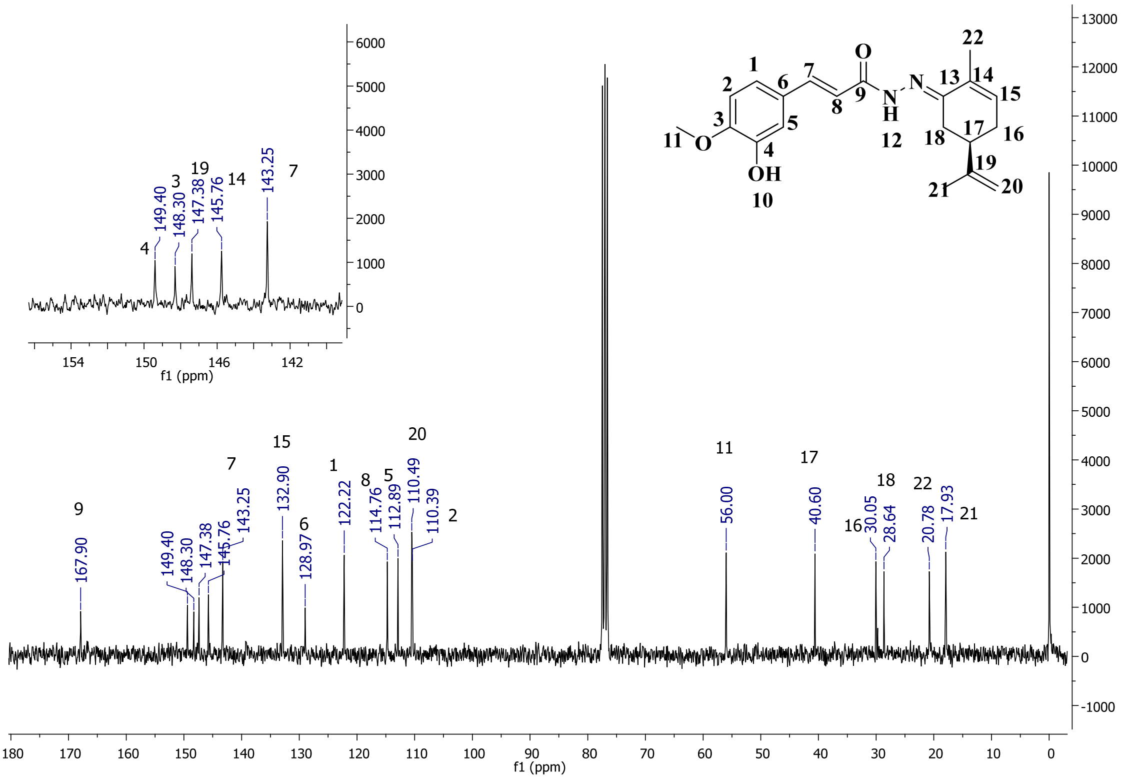

Figure S130. ^{13}C NMR spectrum (75 MHz, $CDCl_3$) of compound **PQM309.**

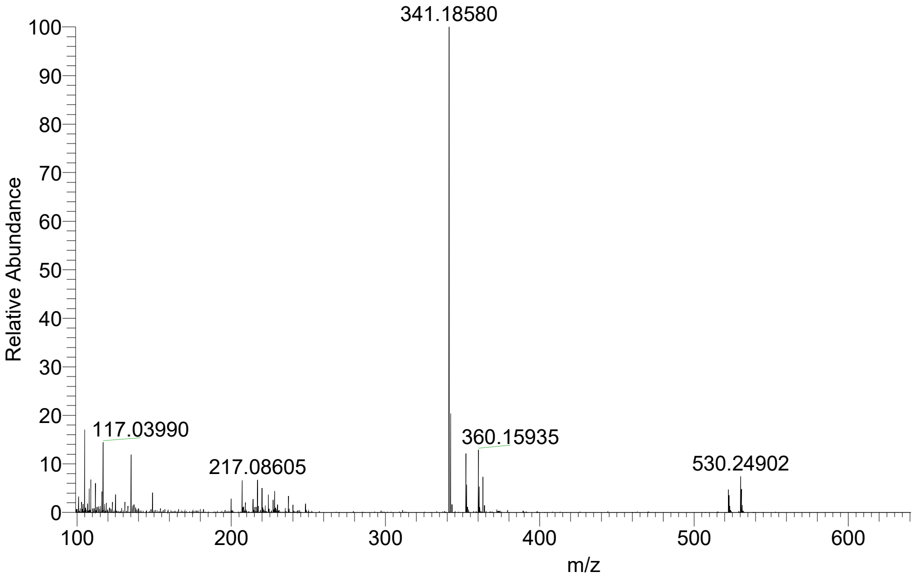

Figure S131. Mass spectrum (ESI-MS) of compound **PQM309.**

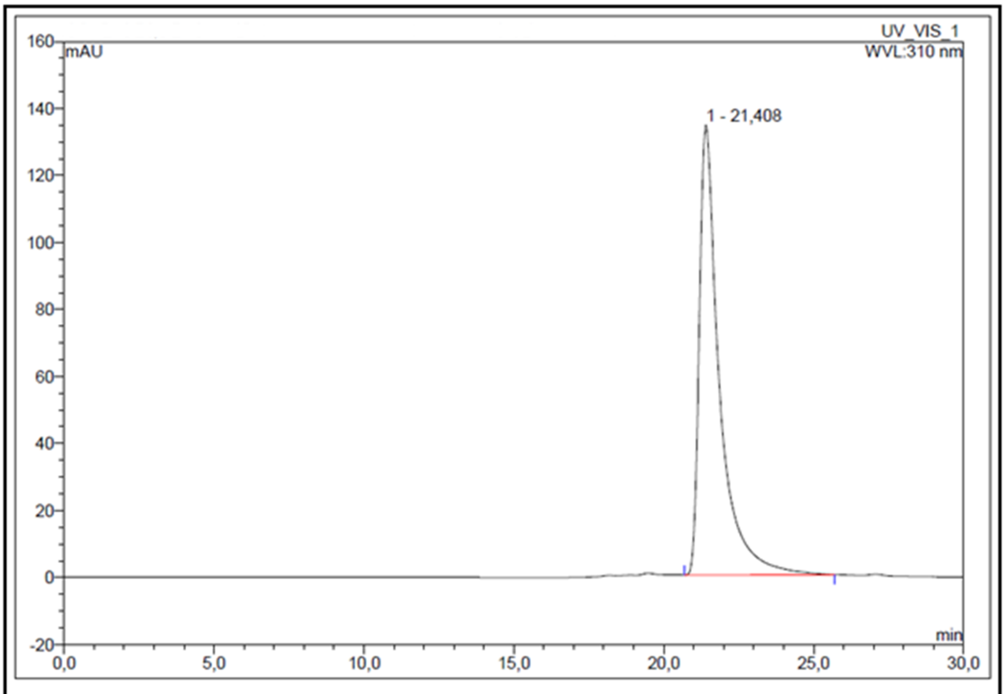

No.	Ret.Time min	Peak Name	Height mAU	Area mAU*min	Rel.Area %	Amount	Type
1	21,41	n.a.	134,310	104,657	100,00	n.a.	BMB
Total:			134,310	104,657	100,00	0,000	

Figure S132. HPLC chromatogram of compound **PQM309.**

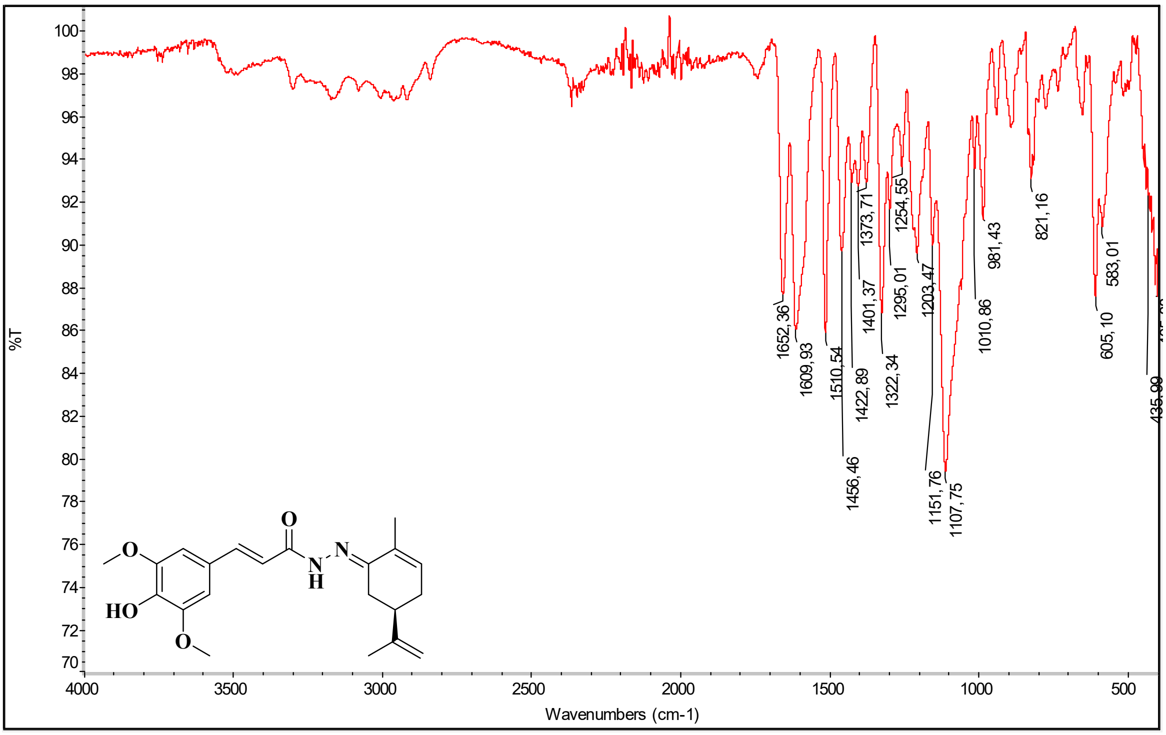

Figure S133. Absorption spectrum in the IR region (ATR) of compound **PQM375**.

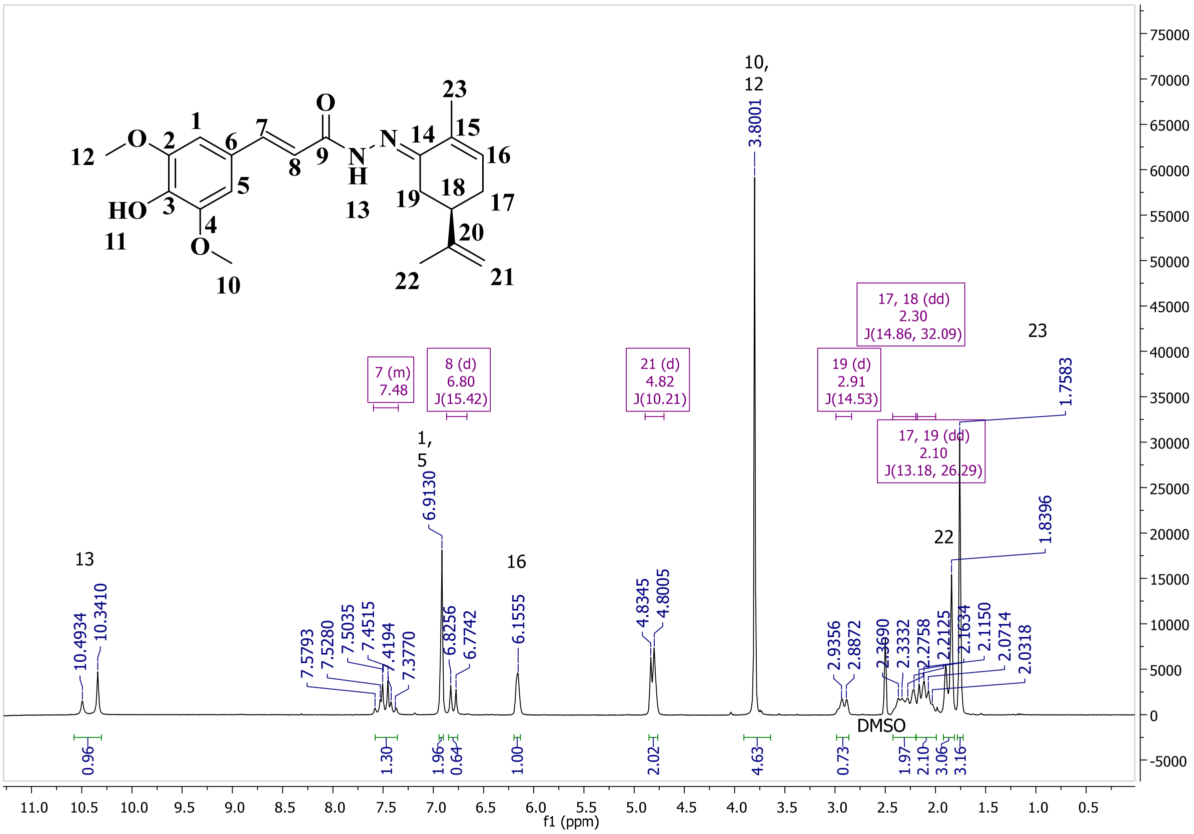

Figure S134. ^{1}H NMR spectrum (300 MHz, DMSO-d_6) of compound **PQM375.**

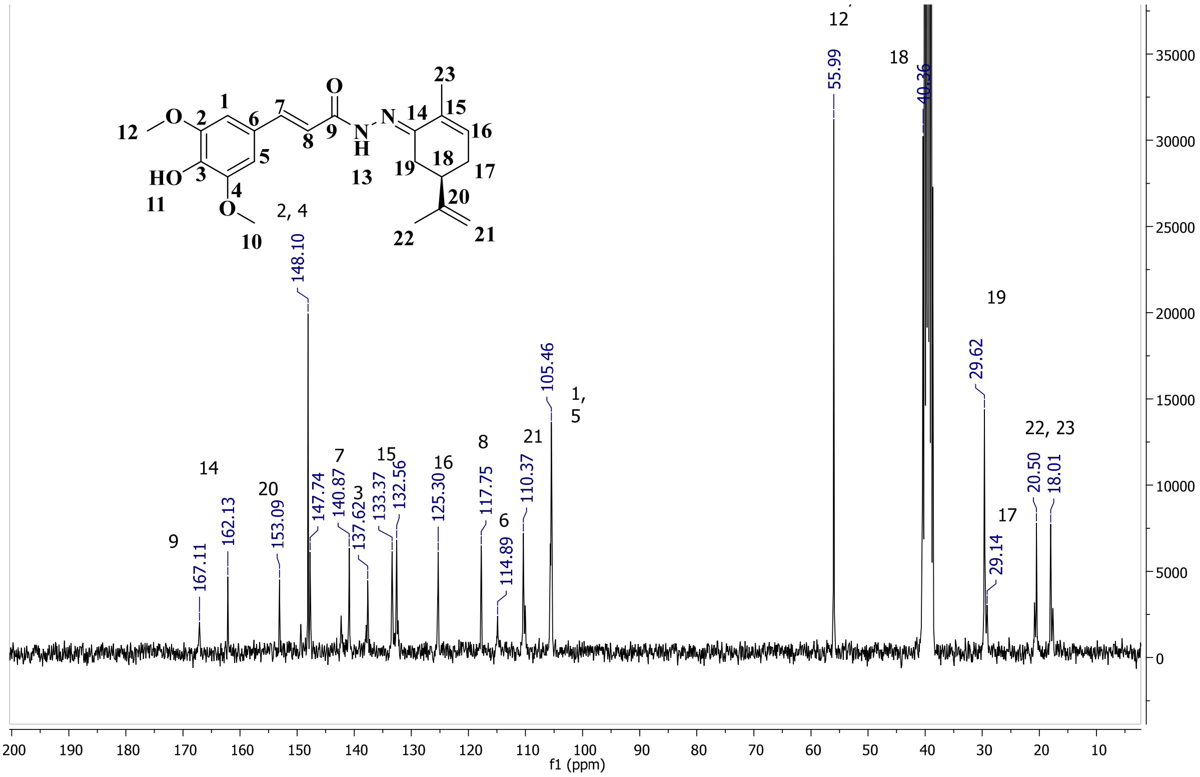

Figure S135. ^{13}C NMR spectrum (300 MHz, DMSO-d_6) of compound **PQM375**.

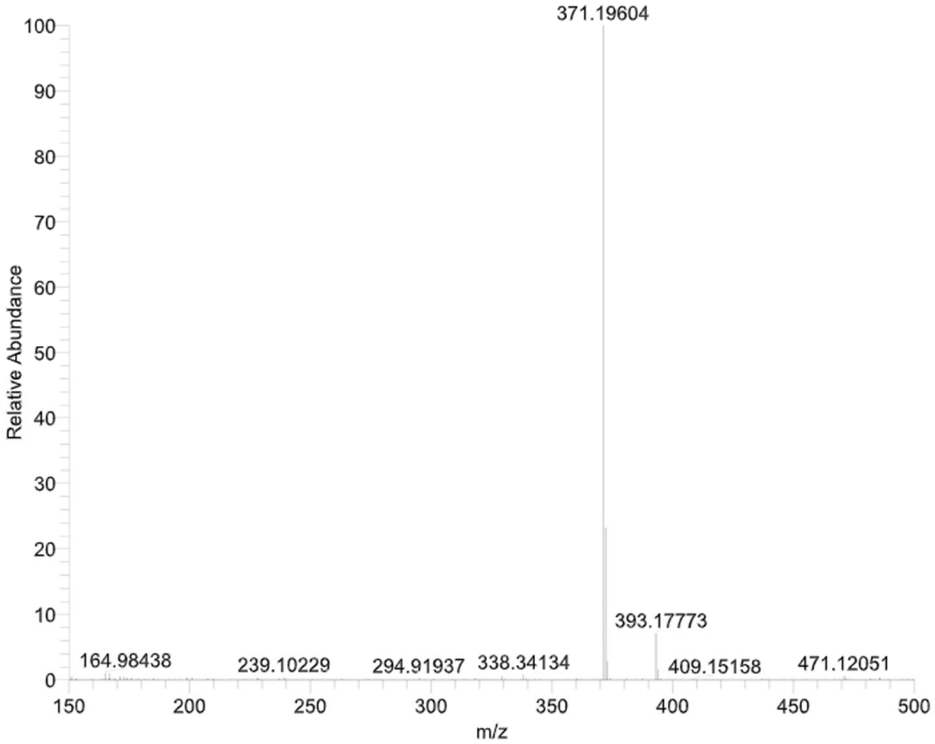

Figure S136. Mass spectrum (ESI-MS) of compound **PQM375.**

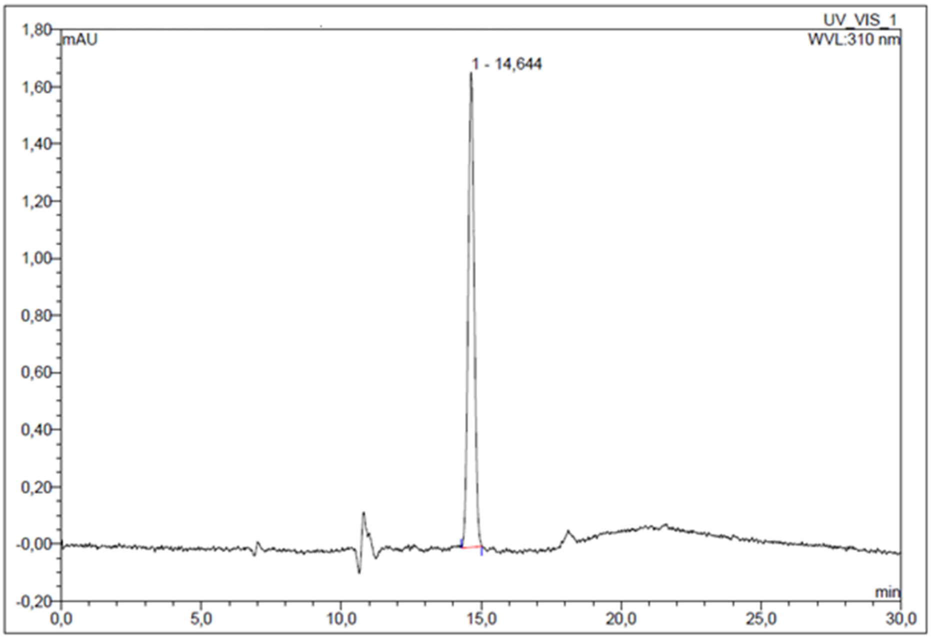

No.	Ret.Time min	Peak Name	Height mAU	Area mAU*min	Rel.Area %	Amount	Type
1	14,64	n.a.	1,664	0,419	100,00	n.a.	BMB
Total:			1,664	0,419	100,00	0,000	

Figure S137. HPLC chromatogram of compound **PQM375.**

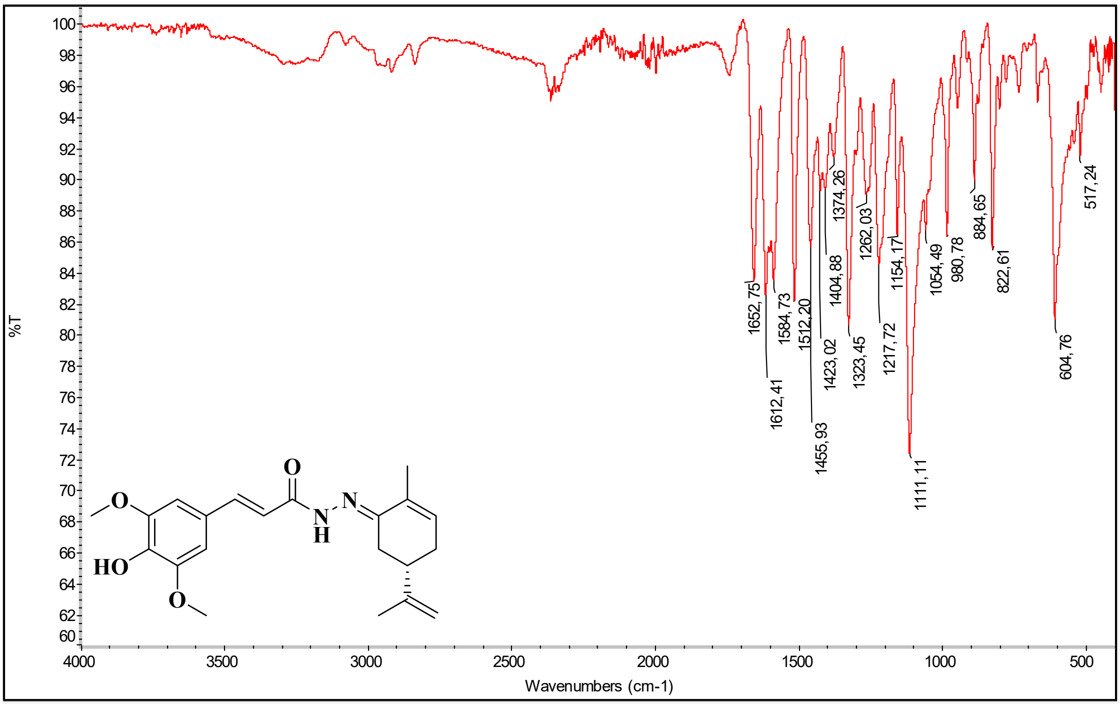

Figure S138. Absorption spectrum in the IR region (ATR) of compound **PQM376**.

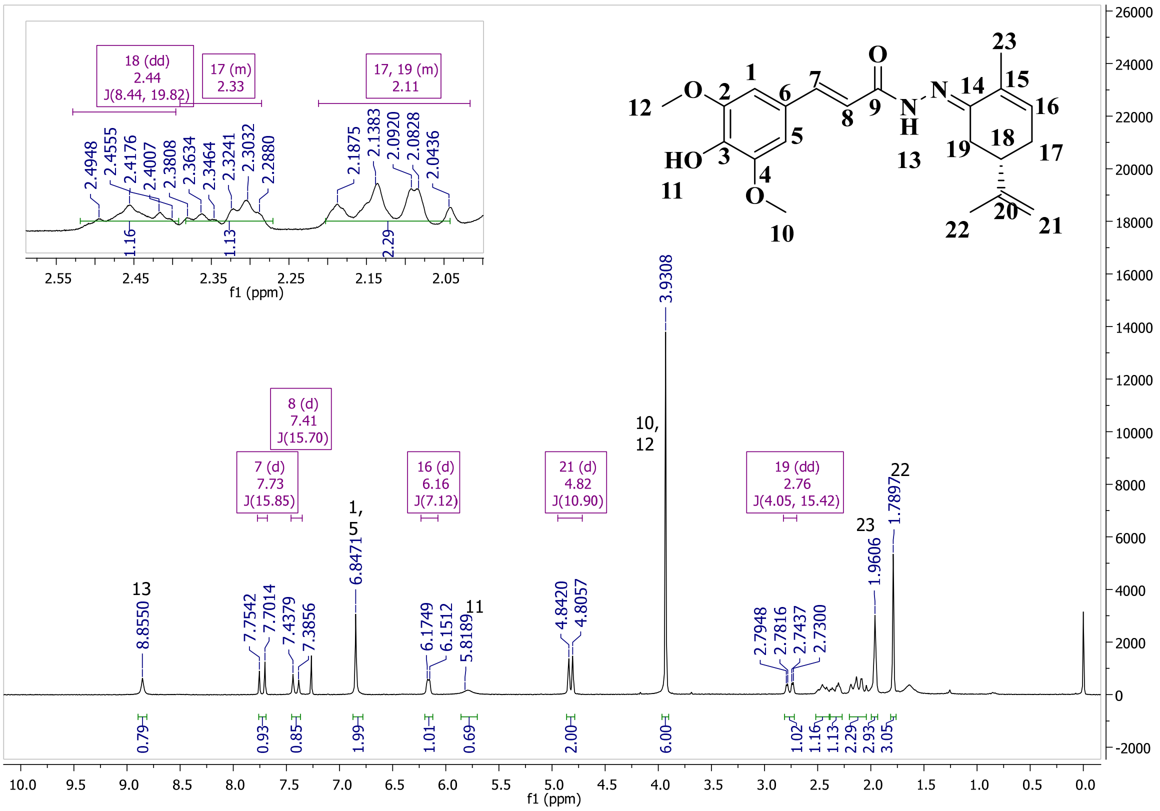

Figure S139. ^{1}H NMR spectrum (300 MHz, $CDCl_3$) of compound **PQM376**.

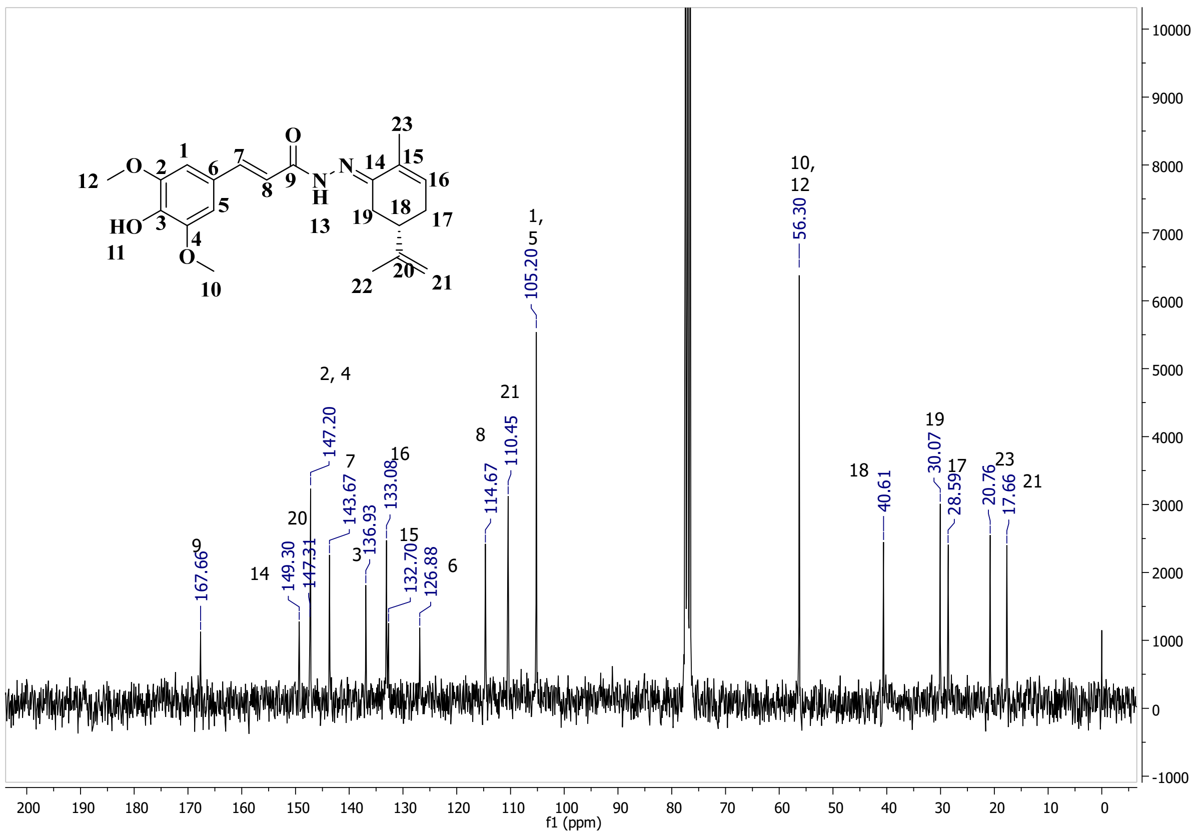

Figure S140. ^{13}C NMR spectrum (75 MHz, $CDCl_3$) of compound **PQM376**.

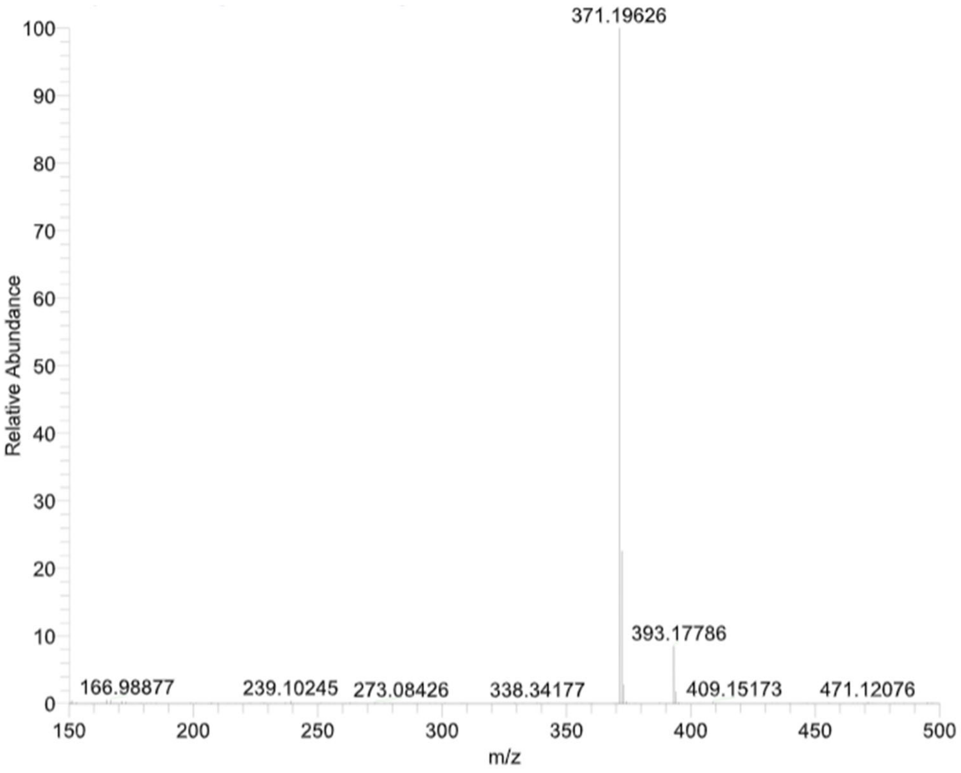

Figure S141. Mass spectrum (ESI-MS) of compound **PQM376.**

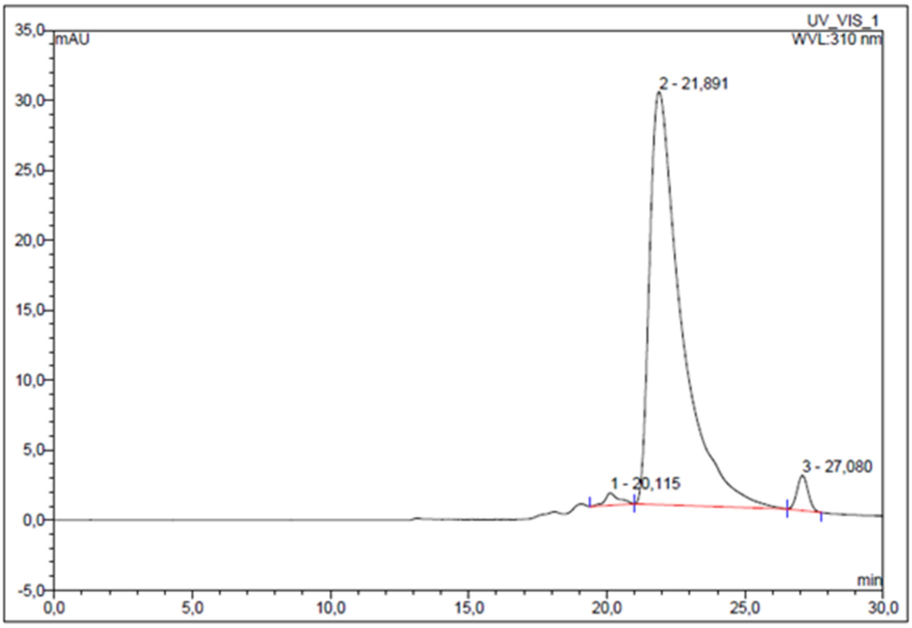

No.	Ret.Time min	Peak Name	Height mAU	Area mAU*min	Rel.Area %	Amount	Type
1	20,11	n.a.	0,892	0,526	1,28	n.a.	BMB
2	21,89	n.a.	29,513	39,293	95,87	n.a.	BMB
3	27,08	n.a.	2,505	1,165	2,84	n.a.	BMB
Total:			32,910	40,984	100,00	0,000	

Figure S142. HPLC chromatogram of compound **PQM376**.

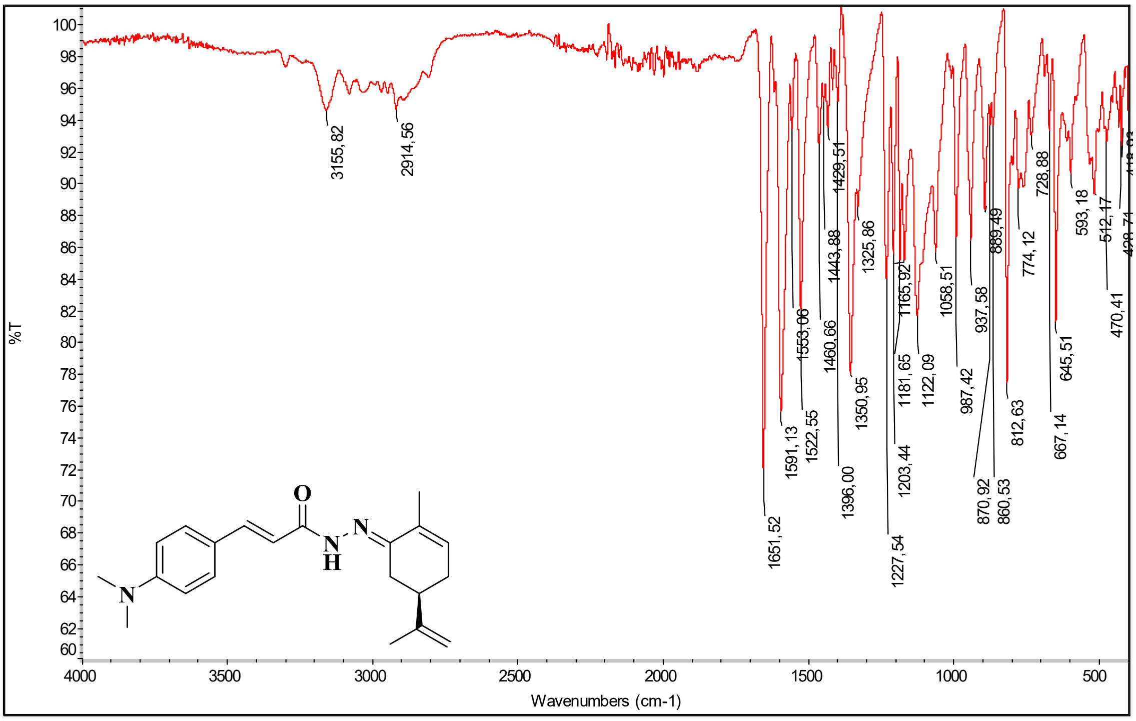

Figure S143. Absorption spectrum in the IR region (ATR) of compound **PQM377.**

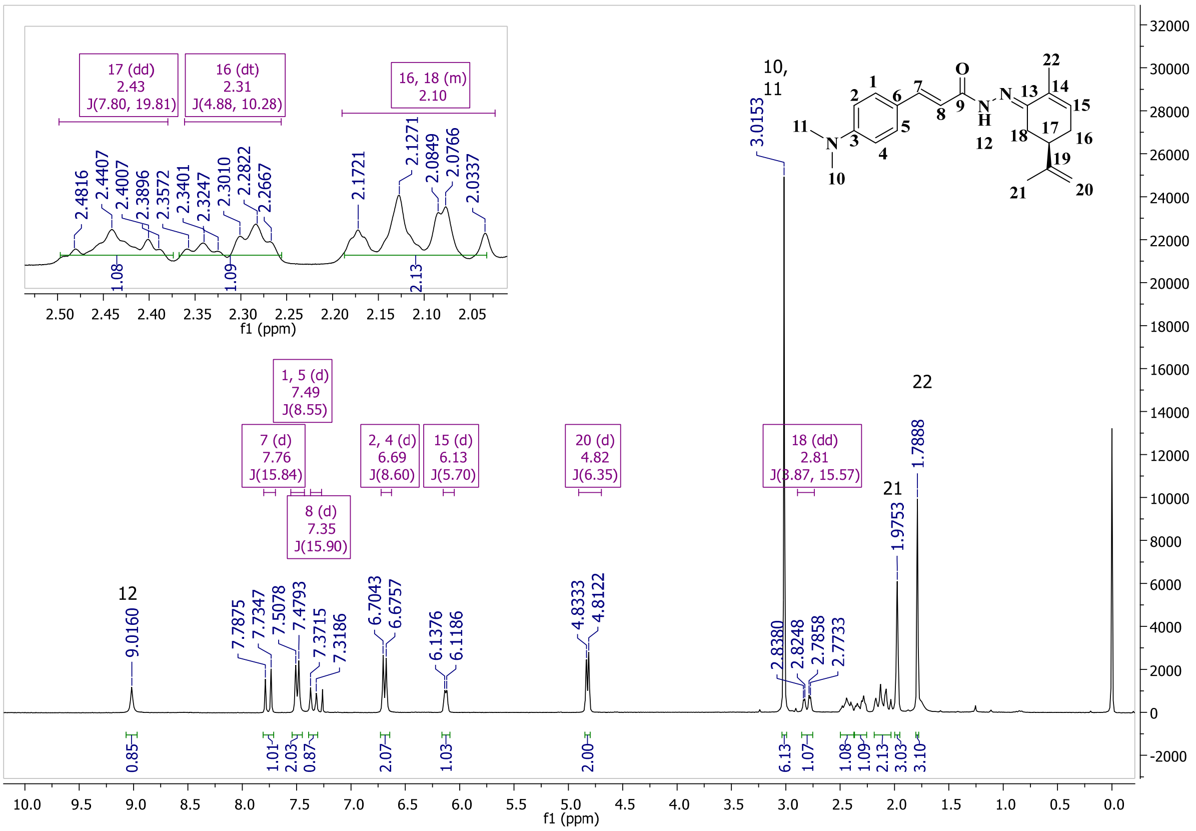

Figure S144. ^{1}H NMR spectrum (300 MHz, $CDCl_3$) of compound **PQM377**.

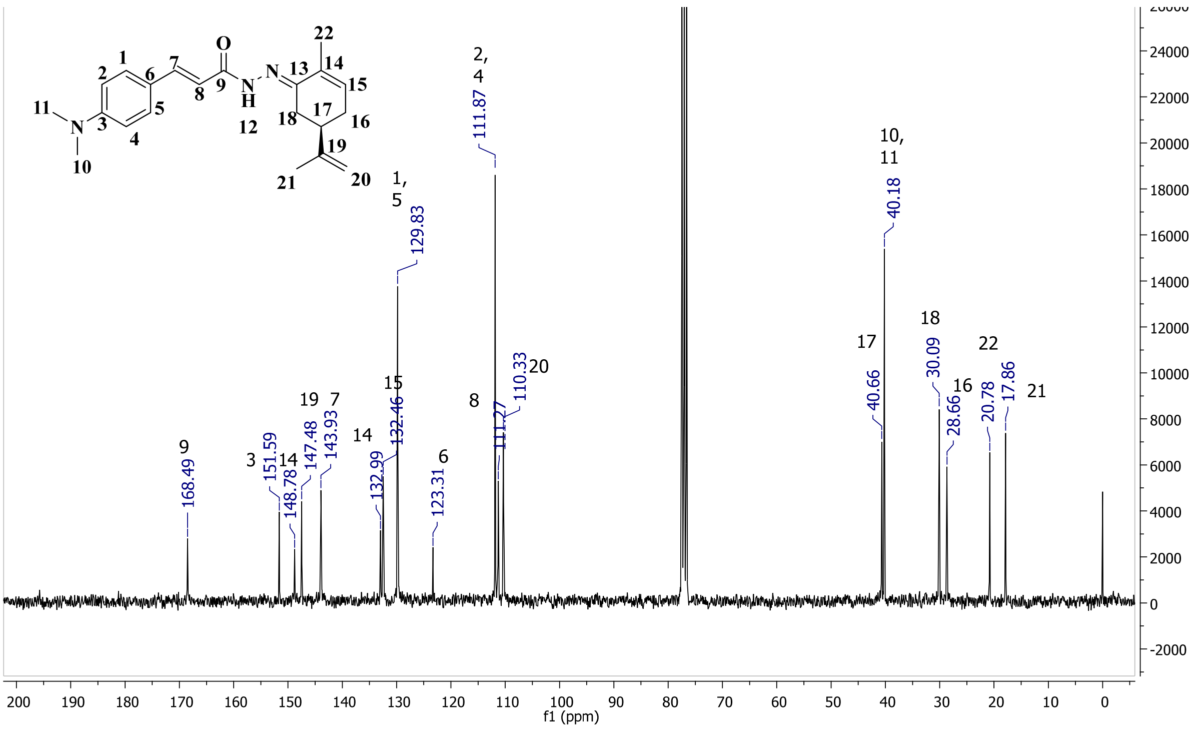

Figure S145. ^{13}C NMR spectrum (75 MHz, $CDCl_3$) of compound **PQM377.**

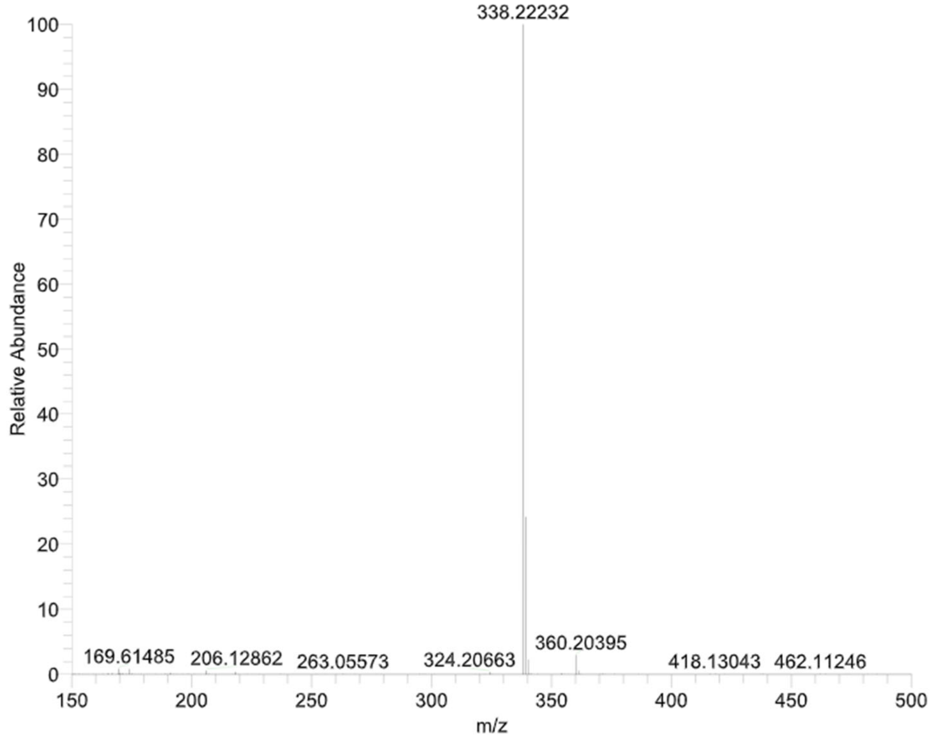

Figure S146. Mass spectrum (ESI-MS) of compound **PQM377.**

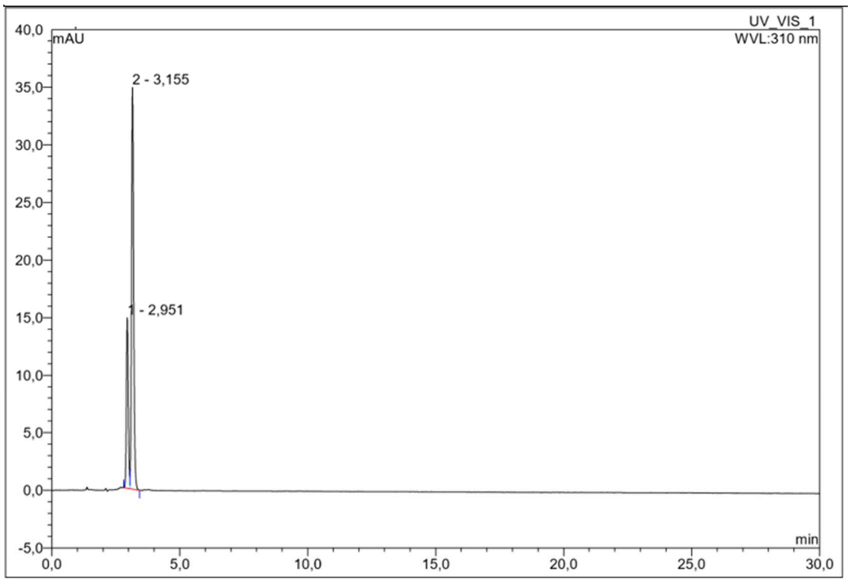

No.	Ret.Time min	Peak Name	Height mAU	Area mAU*min	Rel.Area %	Amount	Type
1	2,95	n.a.	14,843	1,271	27,74	n.a.	BM
2	3,16	n.a.	34,899	3,312	72,26	n.a.	MB

Figure S147. HPLC chromatogram of compound **PQM377.**

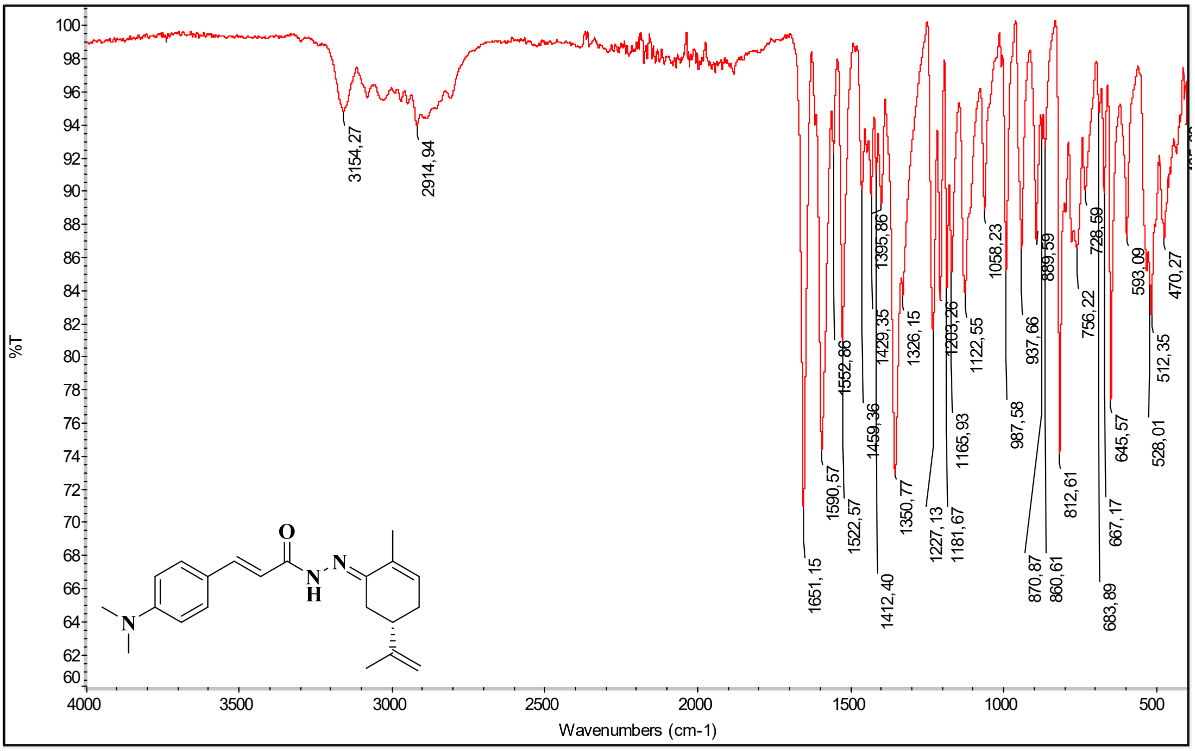

Figure S148. Absorption spectrum in the IR region (ATR) of compound **PQM378**.

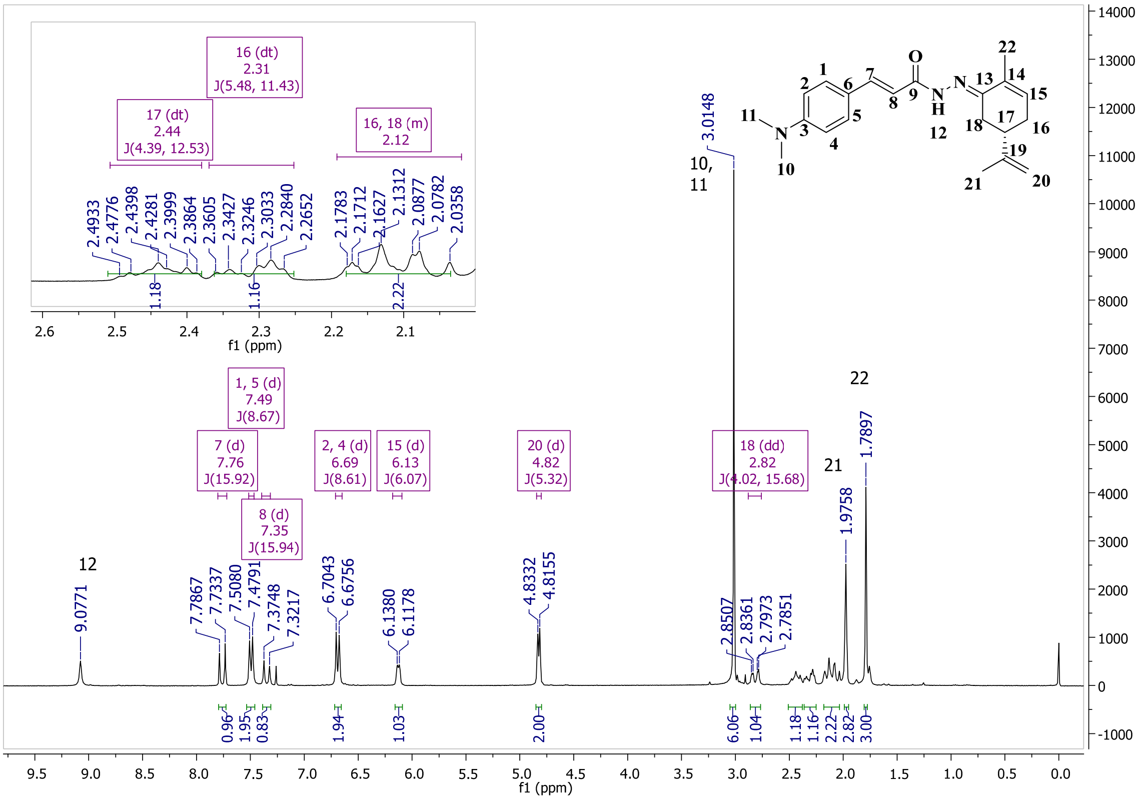

Figure S149. ^{1}H NMR spectrum (300 MHz, $CDCl_3$) of compounds **PQM378**.

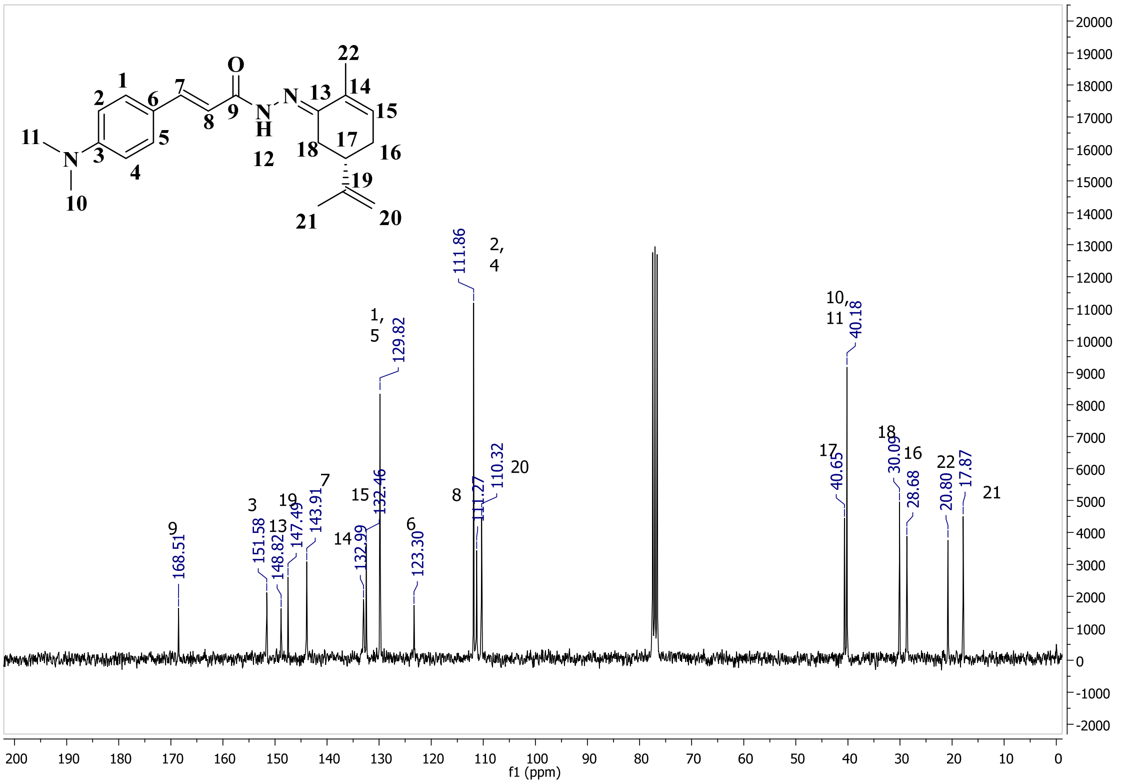

Figure S150. ^{13}C NMR spectrum (75 MHz, $CDCl_3$) of compound **PQM378.**

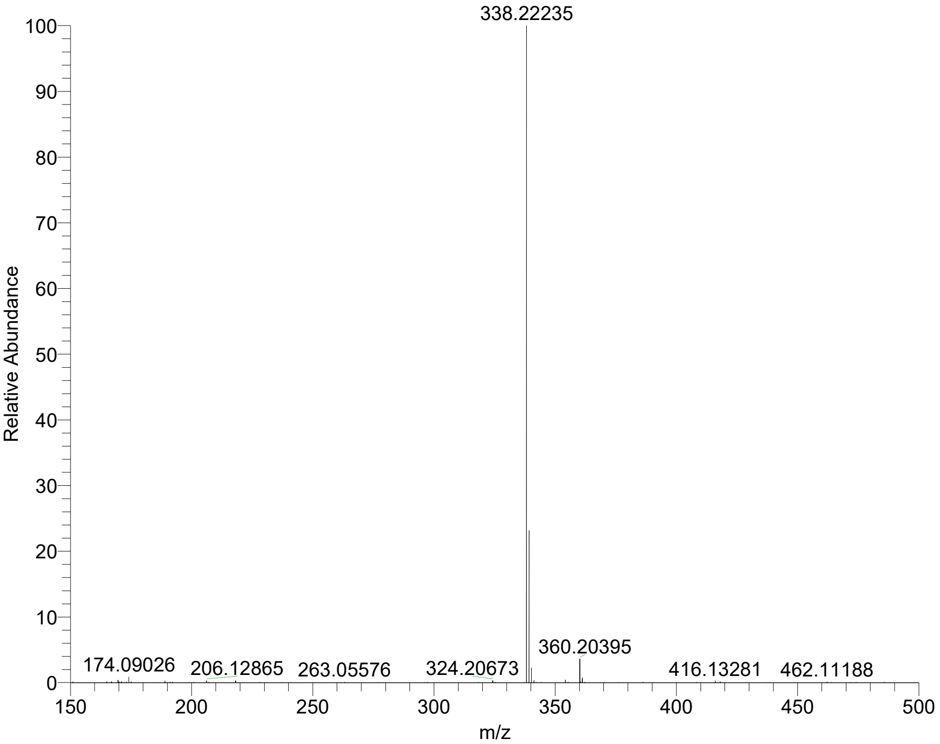

Figure S151. Mass spectrum (ESI-MS) of compound **PQM378.**

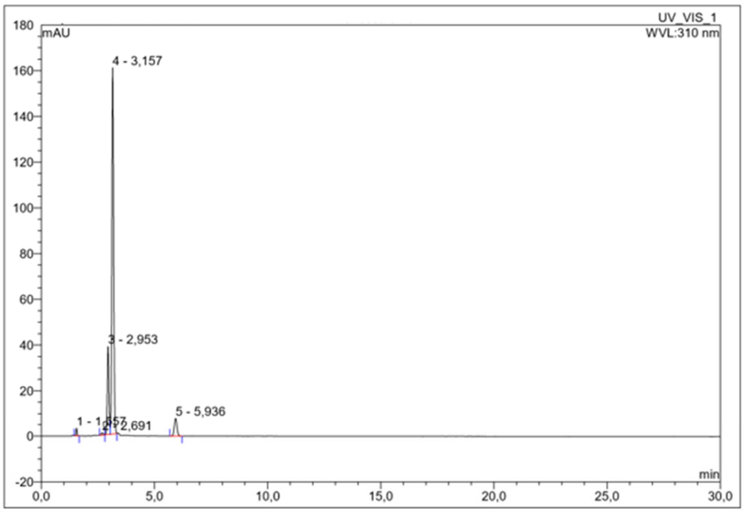

No.	Ret.Time min	Peak Name	Height mAU	Area mAU*min	Rel.Area %	Amount	Type
1	1,56	n.a.	3,330	0,177	0,92	n.a.	BMB
2	2,69	n.a.	1,030	0,092	0,48	n.a.	BM
3	2,95	n.a.	38,720	3,306	17,15	n.a.	M
4	3,16	n.a.	160,403	14,624	75,86	n.a.	MB
5	5,94	n.a.	7,923	1,078	5,59	n.a.	BMB
Total:			211,407	19,276	100,00	0,000	

Figure S152. HPLC chromatogram of compound **PQM378.**

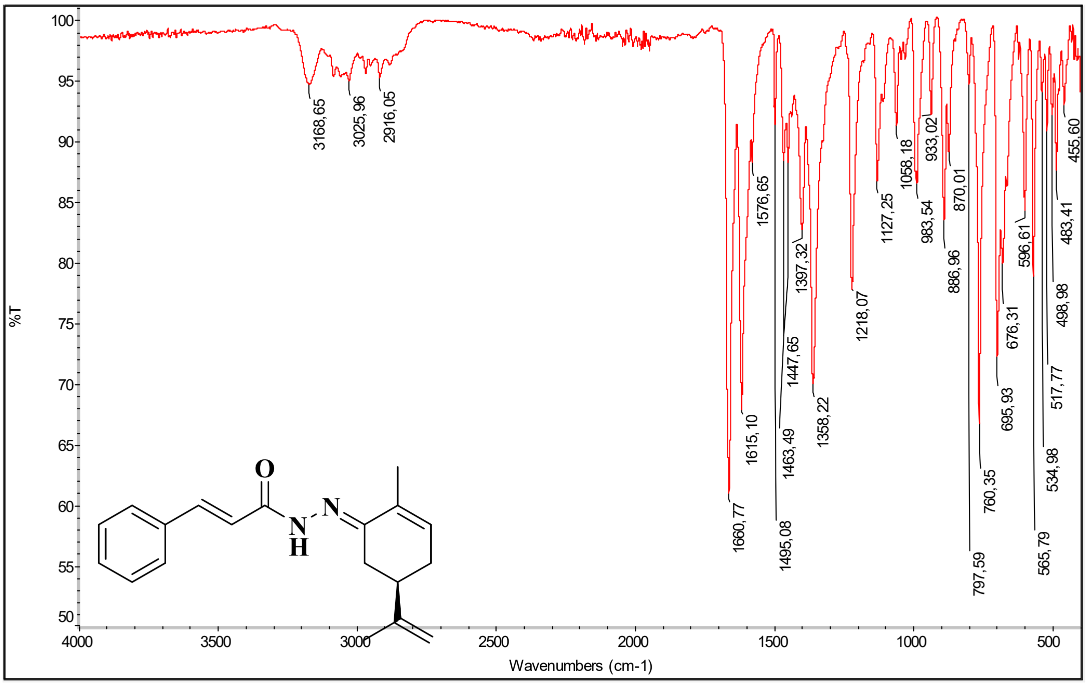

Figure S153. Absorption spectrum in the IR region (ATR) of compound **PQM379**.

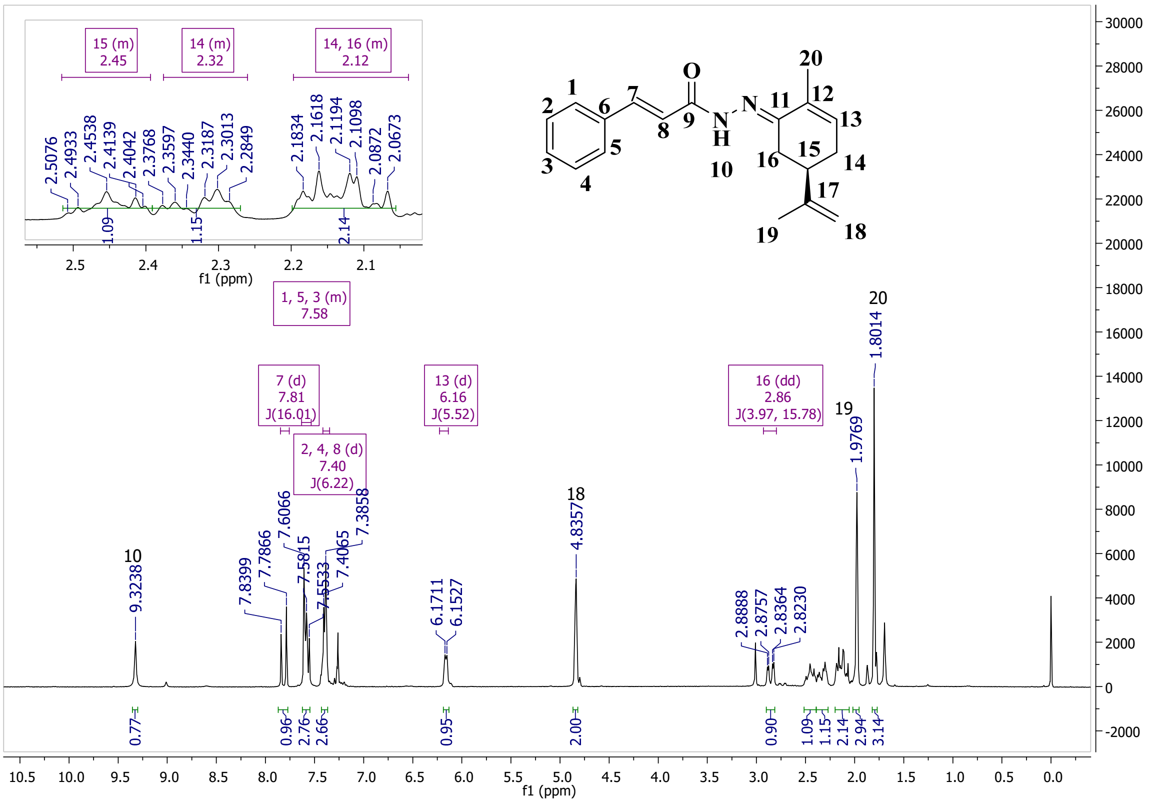

Figure S154. ^{1}H NMR spectrum (300 MHz, $CDCl_3$) of compound **PQM379**.

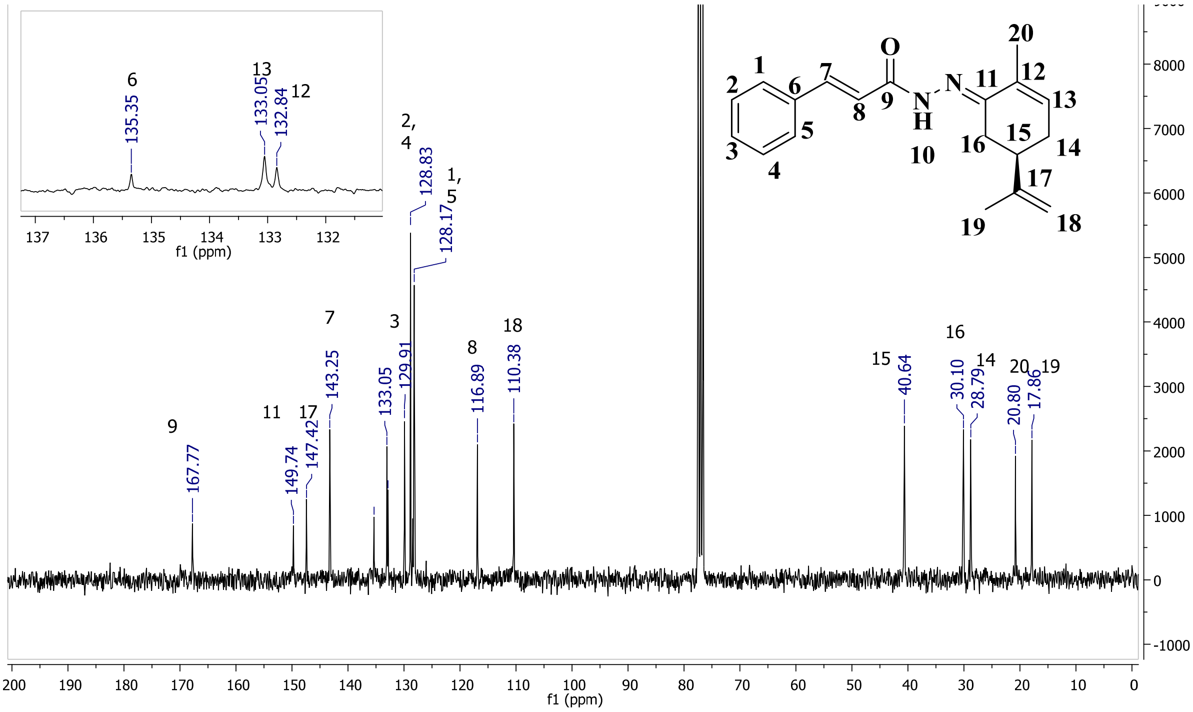

Figure S155. ^{13}C NMR spectrum (75 MHz, $CDCl_3$) of compound **PQM379**.

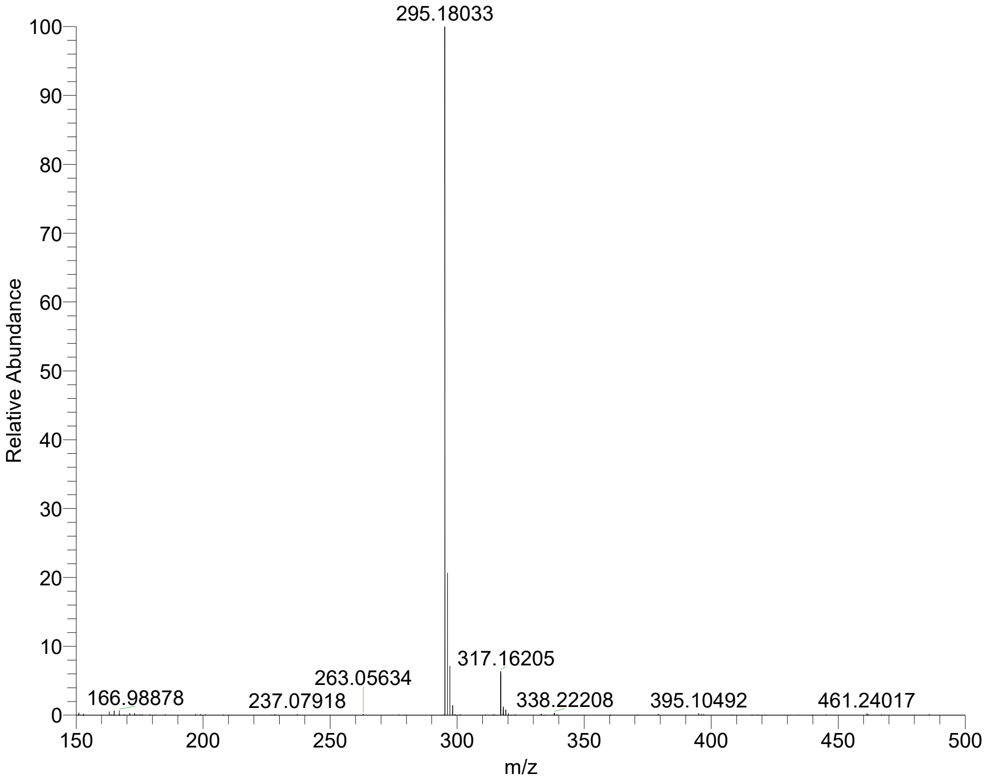

Figure S156. Mass spectrum (ESI-MS) of compound **PQM379**.

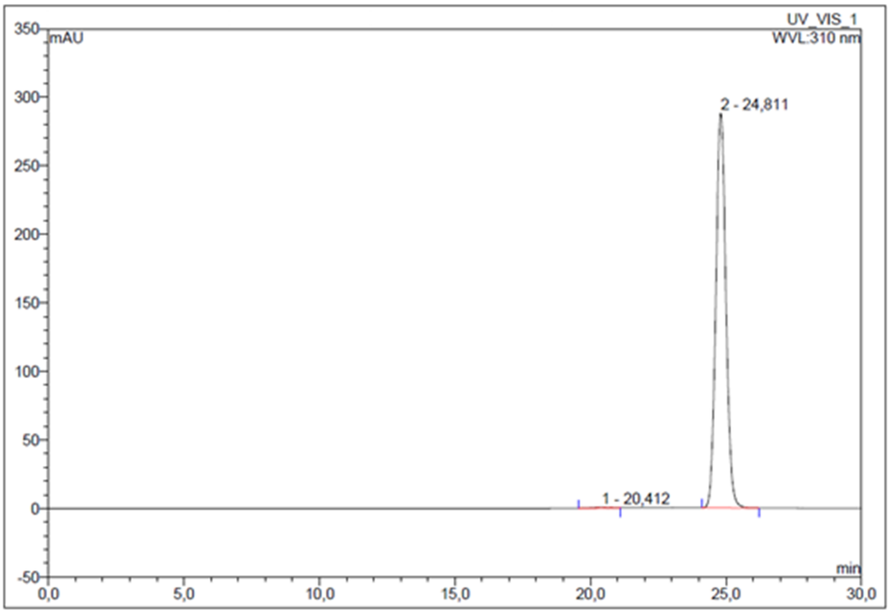

No.	Ret.Time min		Peak Name	Height mAU	Area mAU*min	Rel.Area %	Amount	Type
1	20,41	n.a.		0,629	0,433	0,35	n.a.	BMB
2	24,81	n.a.		287,920	123,925	99,65	n.a.	BMB
Total:				288,549	124,359	100,00	0,000	

Figure S157. HPLC chromatogram of compound **PQM379.**

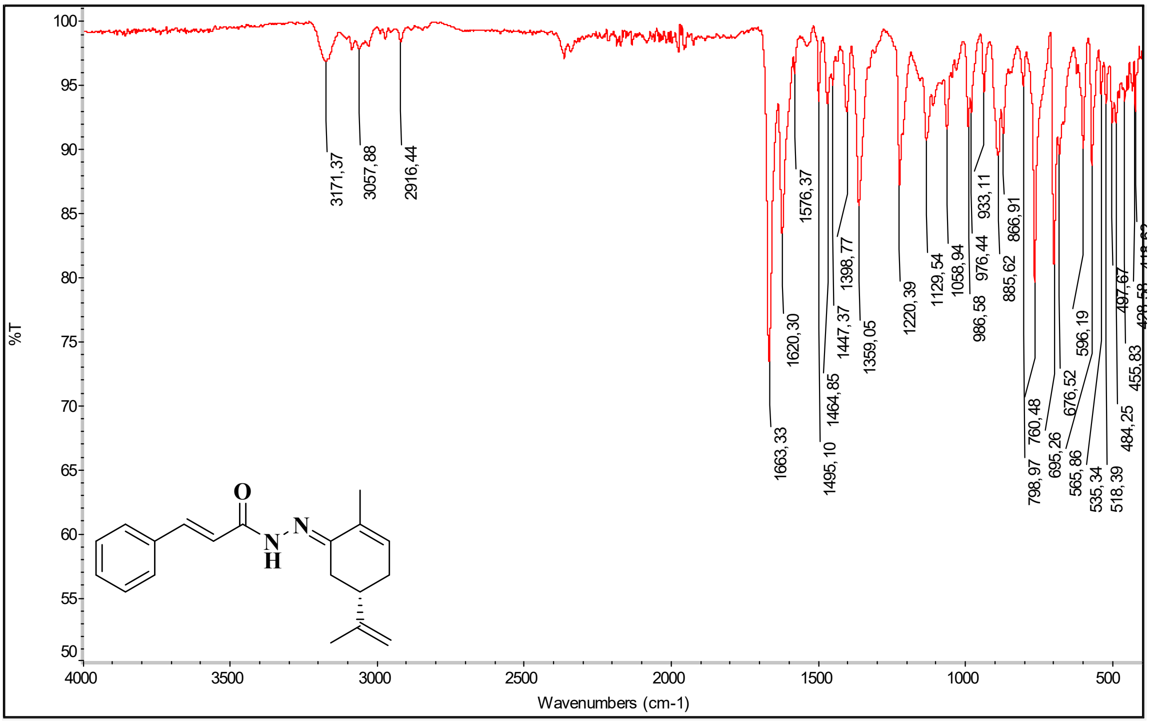

Figure S158. Absorption spectrum in the IR region (ATR) of compound **PQM380.**

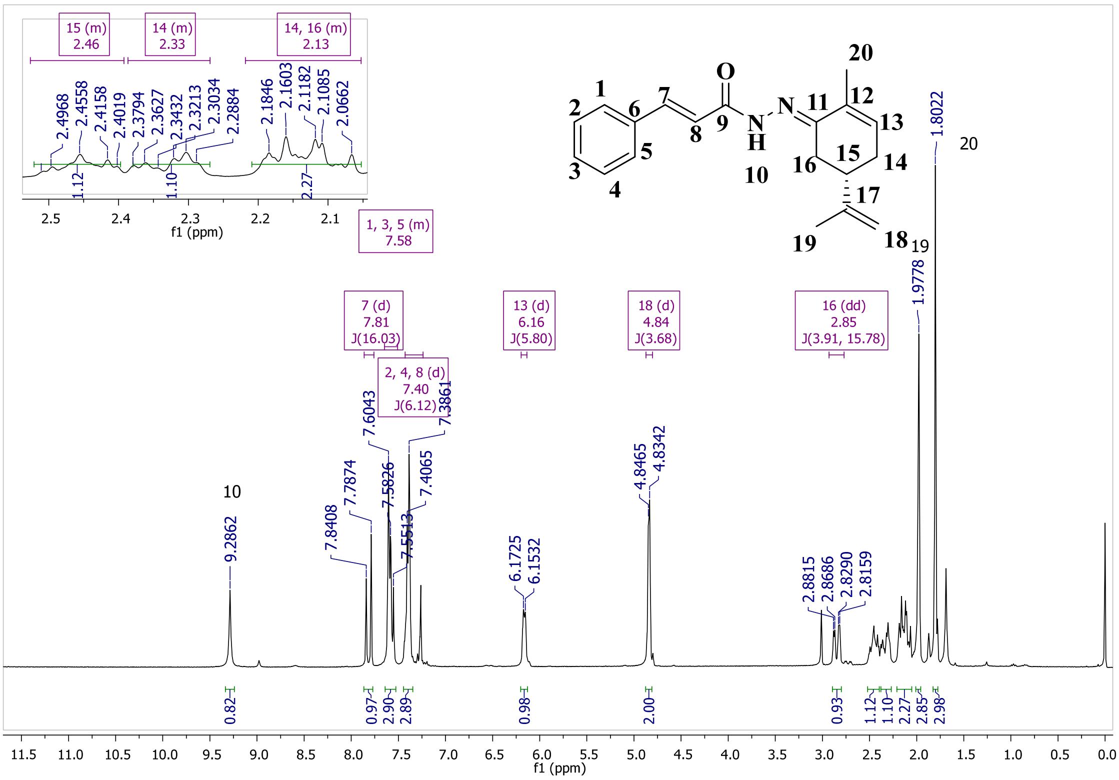

Figure S159. ^{1}H NMR spectrum (300 MHz, $CDCl_3$) of compound **PQM380**.

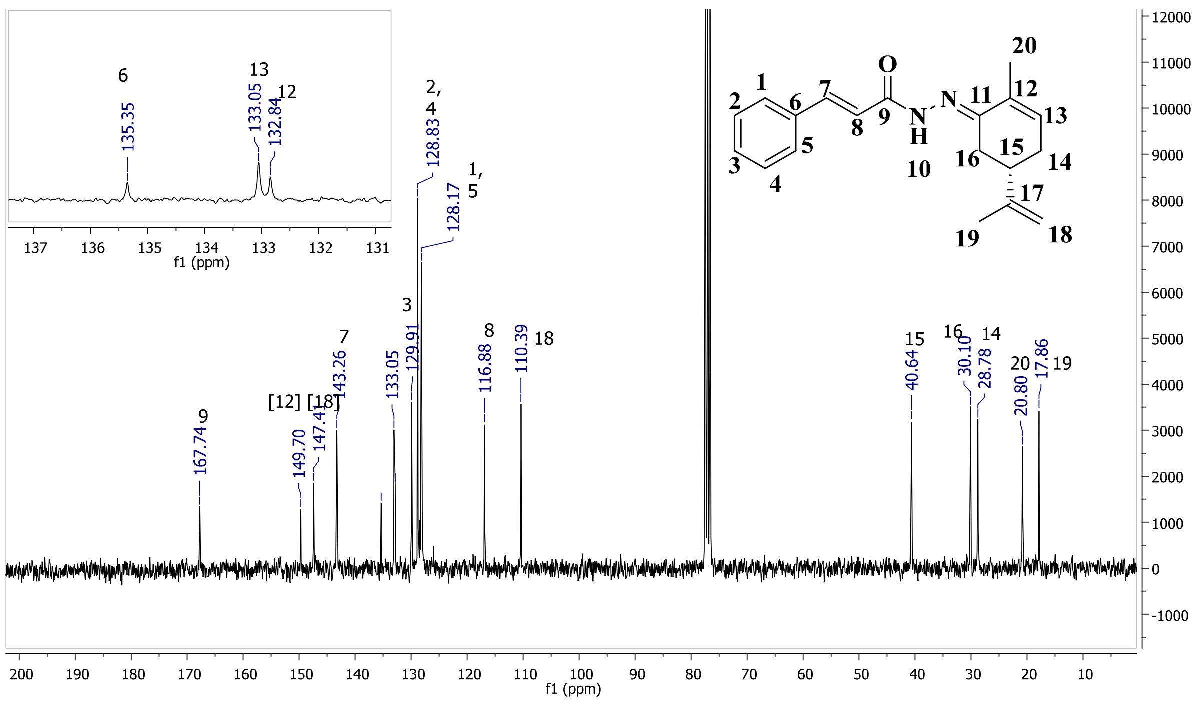

Figure S160. ^{13}C NMR spectrum (75 MHz, $CDCl_3$) of compound **PQM380**.

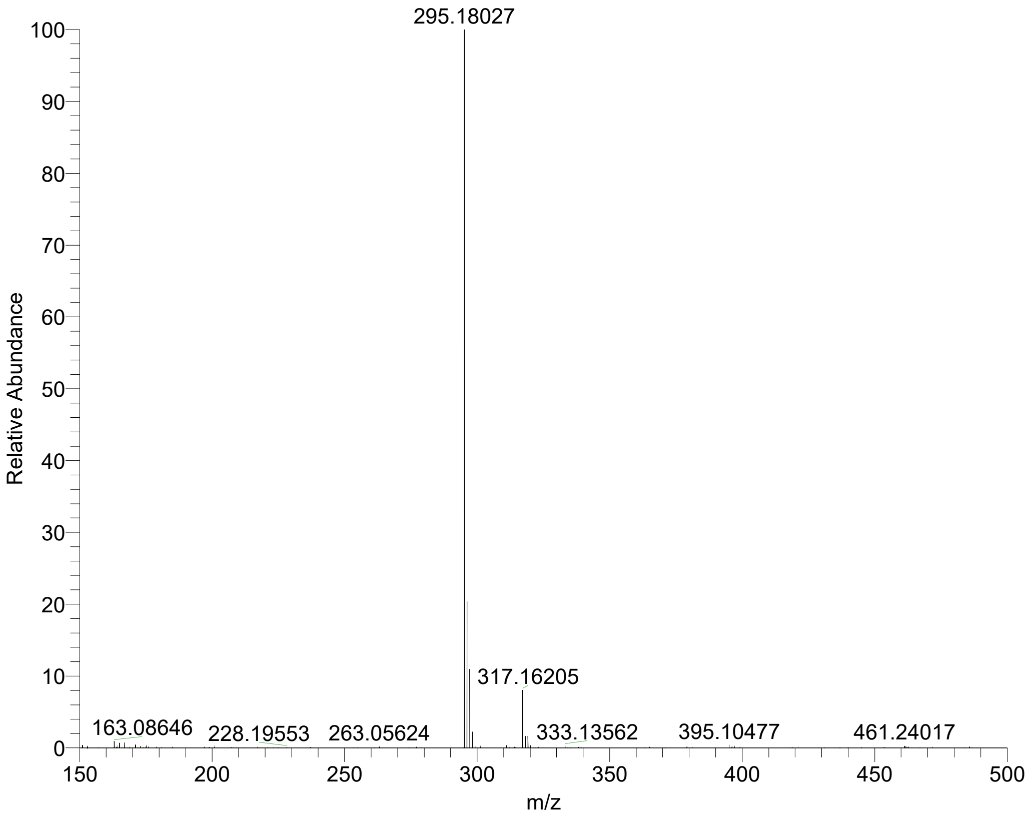

Figure S161. Mass spectrum (ESI-MS) of compound **PQM380**.

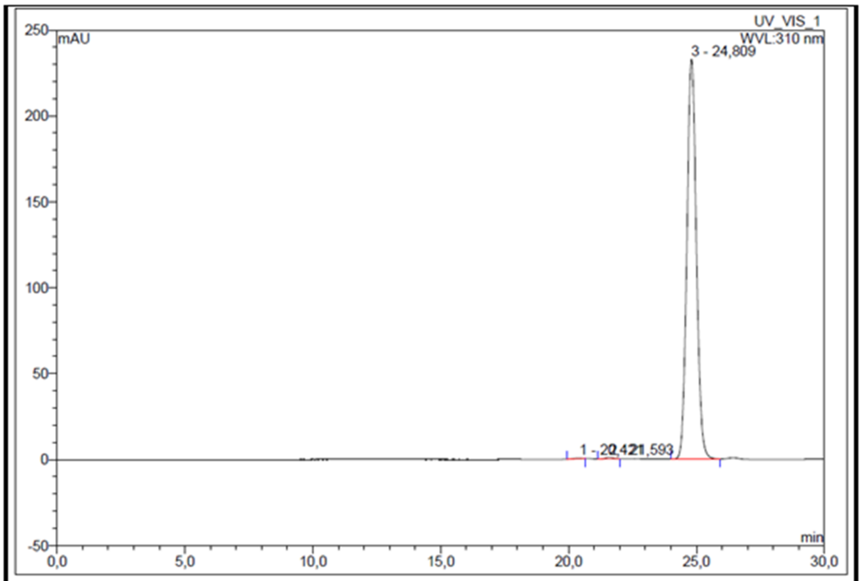

No.	Ret.Time min	Peak Name	Height mAU	Area mAU*min	Rel.Area %	Amount	Type
1	20,42	n.a.	0,292	0,122	0,12	n.a.	BMB
2	21,59	n.a.	0,733	0,257	0,26	n.a.	BMB
3	24,81	n.a.	232,666	99,998	99,62	n.a.	BMB
Total:			233,691	100,376	100,00	0,000	

Figure S162. HPLC chromatogram of compound **PQM380**.
